# A General Method to Improve Fluorophores Using Deuterated Auxochromes

**DOI:** 10.1021/jacsau.1c00006

**Published:** 2021-04-23

**Authors:** Jonathan B. Grimm, Liangqi Xie, Jason C. Casler, Ronak Patel, Ariana N. Tkachuk, Natalie Falco, Heejun Choi, Jennifer Lippincott-Schwartz, Timothy A. Brown, Benjamin S. Glick, Zhe Liu, Luke D. Lavis

**Affiliations:** † Janelia Research Campus, Howard Hughes Medical Institute, 19700 Helix Drive, Ashburn, Virginia 20147, United States; ‡ Department of Molecular Genetics and Cell Biology, University of Chicago, 920 East 58th Street, Chicago, Illinois 60637, United States

**Keywords:** fluorescence, isotope effect, microscopy, organic chemistry, photochemistry, photobleaching, rhodamine, single-molecule imaging

## Abstract

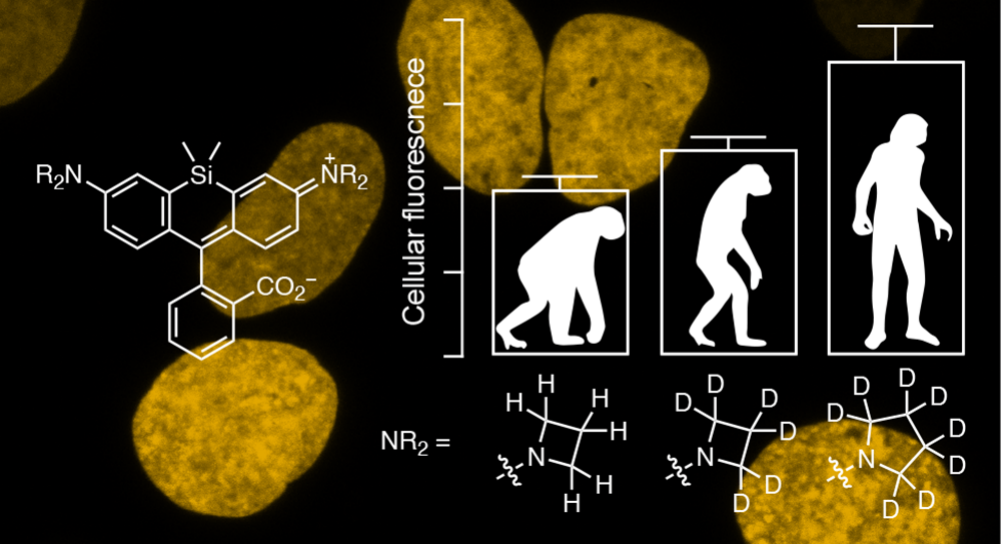

Fluorescence microscopy relies on dyes that absorb and then emit photons. In addition to fluorescence, fluorophores can undergo photochemical processes that decrease quantum yield or result in spectral shifts and irreversible photobleaching. Chemical strategies that suppress these undesirable pathways—thereby increasing the brightness and photostability of fluorophores—are crucial for advancing the frontier of bioimaging. Here, we describe a general method to improve small-molecule fluorophores by incorporating deuterium into the alkylamino auxochromes of rhodamines and other dyes. This strategy increases fluorescence quantum yield, inhibits photochemically induced spectral shifts, and slows irreparable photobleaching, yielding next-generation labels with improved performance in cellular imaging experiments.

## Introduction

Fluorescence microscopy is a powerful tool to visualize the location and dynamics of biomolecules in living systems. The development of advanced microscopy techniques such as live-cell single-molecule imaging promises the visualization of proteins and other cellular components with high spatial and temporal precision. These new microscopy methods are photon-intensive, however, placing increased demands on the fluorescent labels. The unrelenting need for more photons is driving a renaissance in the field of small-molecule fluorophores, which exhibit the requisite brightness and photostability for advanced microscopy techniques and can be adapted to disparate imaging modalities and labeling strategies.^1^

There are several avenues for enhancing small-molecule fluorescent dyes.^2^ Increasing the fluorescence quantum yield (Φ_f_) is an obvious way to improve imaging because brighter dyes translate more excitation light into emitted photons. Fluorophores can undergo various photochemical reactions that yield nonfluorescent products (i.e., photobleaching). Designing fluorophores with improved photostability enables longer duration imaging. Small-molecule dyes can undergo other photochemistry that elicits undesirable shifts in the absorption maximum (λ_abs_) and fluorescence emission maximum (λ_em_), which effectively broadens spectra and decreases excitation efficiency. Thus, improving fluorophore “chromostability” is also advantageous for imaging. Here, we describe a general method to improve small-molecule fluorophores through installation of deuterated auxochromes. This straightforward modification of dyes—the net addition of a few neutrons—enhances Φ_f_, photostability, and chromostability, resulting in fluorophores with improved performance in cellular imaging experiments.

## Results and Discussion

Rational optimization of small-molecule dyes requires an understanding of dye photophysics and the ability to easily modulate fluorophore structure using chemistry. We considered the rhodamine dyes, which persist in modern biological imaging due to their excellent brightness, superb photostability, and tunable spectral and chemical properties.^3−8^ The photophysics of rhodamines are well-understood due to their importance as laser dyes and biological probes.^2^ Rhodamines are also amenable to structural modification using a variety of synthetic organic chemistry strategies.^4,9−11^

**Figure 1:**
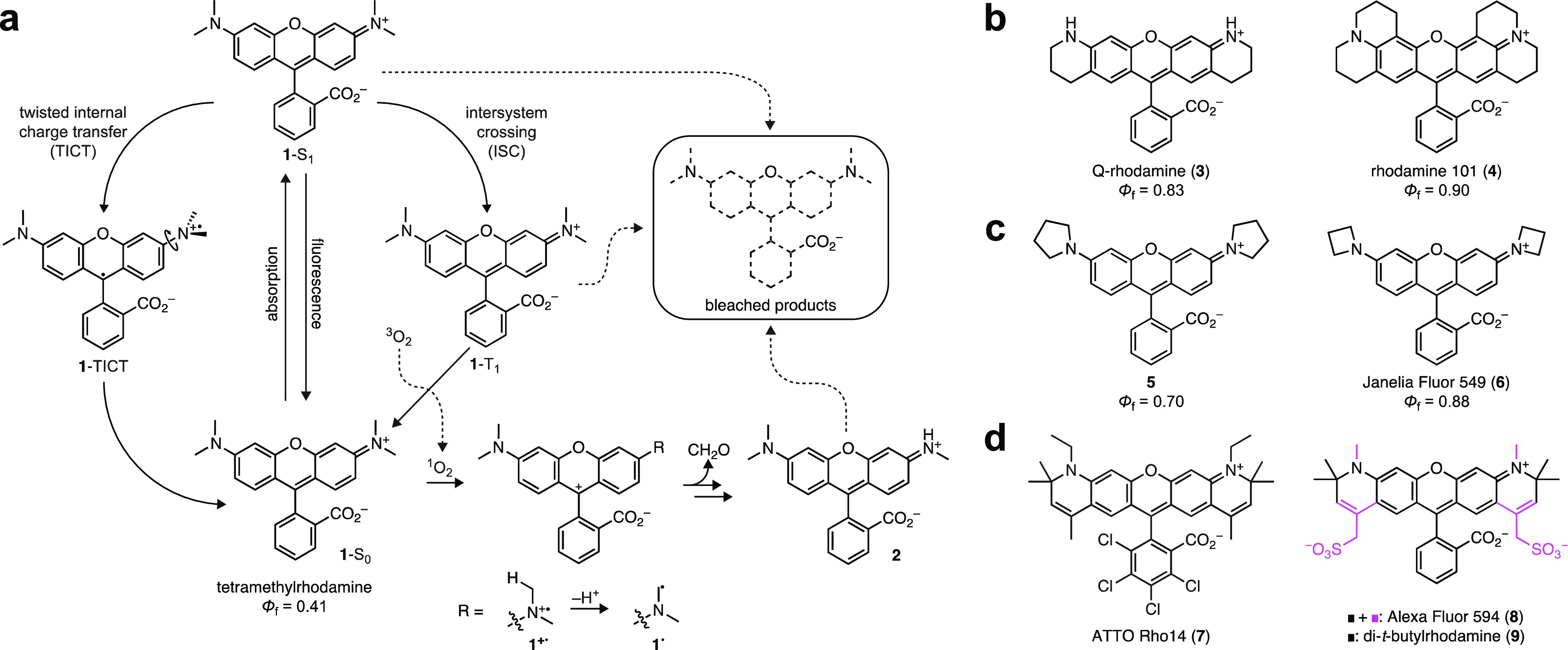
Photophysics of rhodamines and methods to improve rhodamine properties. (a) Photophysics of tetramethylrhodamine (TMR, **1**). (b) Structures of rigidified rhodamines **3**–**4**. (c) Structures of cyclic amine-containing rhodamines **5**–**6**. (c) Structures of α-quaternary rhodamines **7**–**9**.

The classic fluorophore tetramethylrhodamine (TMR, **1**, Figure 1a) illustrates the intricate photophysics of rhodamine dyes. Absorption of a photon excites the dye from the ground state (**1**-S_0_) ultimately to the first excited state (**1**-S_1_). After excitation, the molecule can relax back to **1**-S_0_ through different processes. Emission of a photon (fluorescence) competes with nonradiative decay pathways such as twisted internal charge transfer (TICT),^12^ where electron transfer from the aniline nitrogen to the xanthene system gives a charge-separated species with a twisted C–N bond (**1**-TICT); this decays back to **1**-S_0_ without emitting a photon. TMR is susceptible to nonradiative decay via TICT, leading to a modest quantum yield (Φ_f_ = 0.41).^13^ Alternatively, the excited dye can undergo intersystem crossing (ISC) to the first triplet excited state (**1**-T_1_), from where it can sensitize singlet oxygen (^1^O_2_) and return to **1**-S_0_. The ^1^O_2_ can oxidize the aniline nitrogen to the radical cation (**1**^**+•**^), which can undergo deprotonation to a carbon-centered radical (**1**^**•**^). Reaction with reactive oxygen species eventually results in loss of formaldehyde, giving the dealkylated trimethylrhodamine (**2**).^2^ This leads to a shift in λ_abs_ and λ_em_. The distinct photobleaching reactions of rhodamines remain mysterious with multiple possible pathways (Figure 1a). Nevertheless, dealkylated rhodamines typically exhibit lower photostability, making this photochemical reaction a key initial step in photobleaching.

Both of these undesirable processes—TICT and dealkylation—can be mitigated through modifications in chemical structure. Rigidification of the rhodamine prevents rotation of the C–N bond and improves Φ_f_,^13^ as evidenced by Q-rhodamine (**3**; Φ_f_ = 0.83)^14^ and rhodamine 101 (**4**; Φ_*f*_ = 0.90; Figure 1b).^15^ Decreasing the electron donor strength and minimizing homoallylic interactions can also decrease TICT and improve brightness. This was first demonstrated by Drexhage, a pioneer in fluorophore chemistry, who found that replacing the *N*,*N*-dimethylamino auxochromes in **1** with five-membered pyrrolidine rings afforded a brighter dye (**5**; Φ_f_ = 0.70; Figure 1c).^13^ We discovered that incorporation of smaller, four-membered azetidine rings further improved the brightness of rhodamines and other fluorophores, yielding the “Janelia Fluor” (JF) dyes.^6−8^ The azetidinyl-rhodamine **6** (JF_549_; Φ_f_ = 0.88) shows comparable Φ_f_ to the fully rigidified **4** (Figure 1b, c). The increased ionization potential of azetidine^16^ likely underlies the improved photostability of **6** because it suppresses formation of a radical cation (e.g., **1**^**+•**^, Figure 1a). Finally, the dealkylation process can be inhibited by installing α-quaternary centers on the aniline nitrogens, thereby precluding deprotonation to form radicals such as **1**^**•**^ (Figure 1a). This structural motif was also introduced by Drexhage;^17^ it is found in a number of commercial fluorophores (e.g., **7**–**8**),^18^ and this concept was revisited in the simplified di-*t*-butylrhodamine (**9**, Figure 1d).^19^

We envisioned an alternative strategy to increase brightness and photostability of small-molecule fluorophores such as **1** by replacing the hydrogen (H) atoms in the *N*-alkyl groups with deuterium (D). Oxidation of alkylamines can show remarkably large secondary isotope effects,^20^ suggesting that deuteration could reduce the electron donor strength of the auxochrome. This would decrease the efficiency of the TICT process and increase Φ_f_. This effect would also slow ^1^O_2_-mediated oxidation (e.g., **1** → **1**^**+•**^), and the stronger C–D bond could lower the rate of deprotonation (e.g., **1**^**+•**^ → **1**^**•**^, Figure 1a). Together, these effects would likely decrease undesired dealkylation and improve both chromostability and photostability.

**Figure 2:**
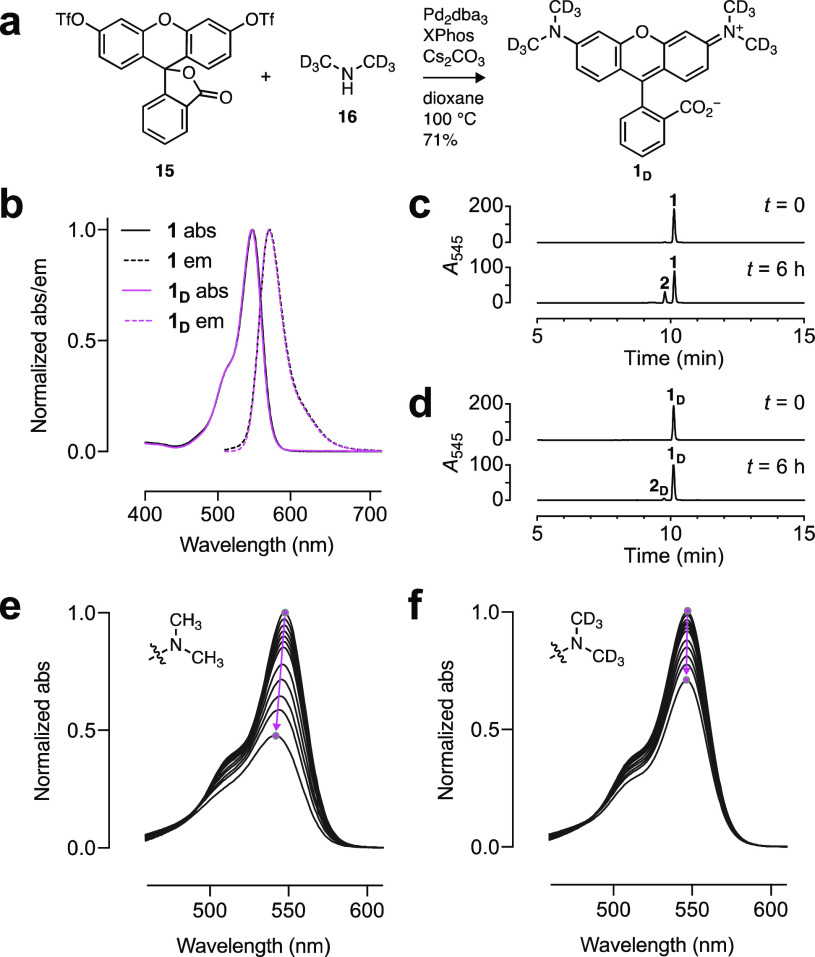
Deuterated tetramethylrhodamine. (a) Synthesis of **1**_**D**_. (b) Normalized absorption (abs) and fluorescence emission (em) spectra of **1** and **1**_**D**_. (c, d) LC–MS traces of **1** (c) and **1**_**D**_ (d) before and after photobleaching using 560 nm (1.02 W/cm^2^, 6 h). (e, f) Sequential absorption spectra of **1** (e) and **1**_**D**_ (f) during photobleaching using 560 nm (1.02 W/cm^2^). The magenta arrows highlight the shift in λ_abs_ and absorption intensity over time.

Deuterium substitution has been suggested as a strategy to improve fluorophores by altering vibrational modes.^21^ This idea is bolstered by the higher Φ_f_ and photostability observed for many fluorophores in deuterated solvents.^22−24^ Prior examples of deuterated dyes are limited, however, and are largely focused on direct attachment of D atoms to the aromatic system of the fluorophore. This substitution typically yields a negative or neutral effect on Φ_f_ as demonstrated for compounds **10**–**14** (Figure S1).^25−27^ The use of deuterated *N*-alkyl auxochromes to control electron and proton transfer represents a new hypothesis, which was initially tested with the classic fluorophore TMR (**1**). The deuterated analogue **1**_**D**_ was synthesized using a cross-coupling approach with fluorescein ditriflate (**15**) and dimethylamine-*d*_6_ (**16**; Figure 2a).^9^ Comparison of dyes **1** and **1**_**D**_ revealed quite similar λ_abs_ and λ_em_, high extinction coefficients at λ_abs_ (ε; Table 1), and no change in the shape of the absorption or fluorescence emission spectra (Figure 1b). Deuteration did affect the brightness of the dye, however, with **1**_**D**_ showing a 22% increase in Φ_f_ compared to **1** (Table 1).^28^

**Table 1:**
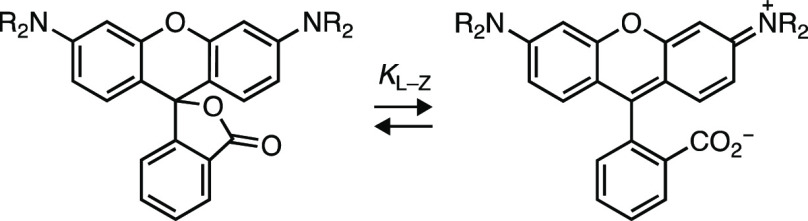

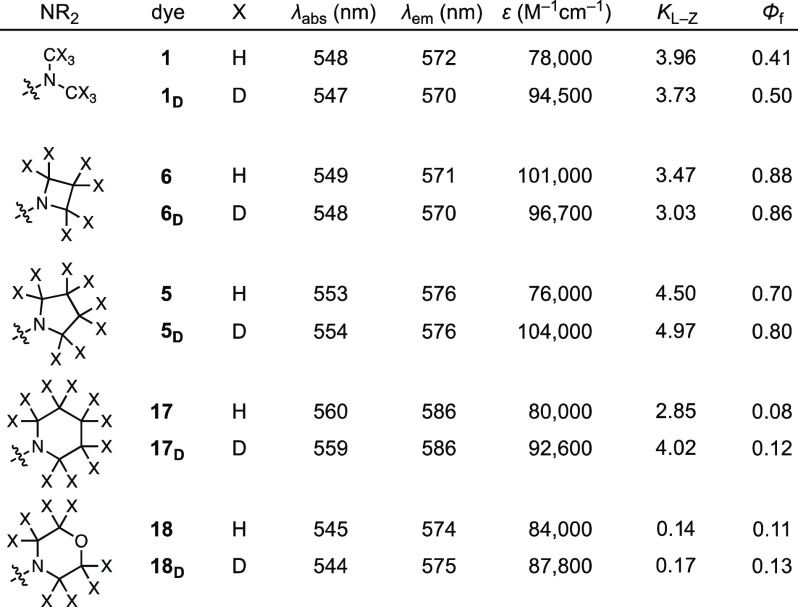
Spectral Properties of Rhodamines^a^

a All values are in 10 mM HEPES, pH 7.3 except for *K*_L–Z_, which was measured in 1:1 v/v dioxane:H_2_O.

The photostabilities of **1** and **1**_**D**_ were then compared *in vitro*. Solutions of both dyes were illuminated with 560 nm light and measured intermittently using tandem liquid chromatography–mass spectrometry (LC–MS) and absorption spectroscopy. For the parent TMR (**1**), LC–MS revealed clean demethylation to trimethylrhodamine (**2**; Figure 2c). This was also observed for the deuterated compound **1**_**D**_ but at a slower rate (Figure 2d, Figure S2a, b). The different degrees of demethylation were reflected in the absorption spectra, where **1** showed faster photobleaching and a more pronounced blue-shift in λ_abs_ compared to the deuterated **1**_**D**_ (Figure 2e, f, Figure S2c, d).

Based on this result with TMR (**1**), a series of matched pairs of rhodamine dyes with H- or D-substituted cyclic *N*-alkyl groups were synthesized (Table 1, Figure S3a). Like the TMR compounds **1** and **1**_**D**_, deuteration did not significantly change λ_abs_, λ_em_, or spectral shape (Table 1, Figure S3b–e). The lactone–zwitterion equilibrium constant (*K*_L–Z_), a key determinant for rhodamine performance in biological environments,^8^ showed only minor changes with deuteration. The pyrrolidine dyes **5** and **5**_**D**_ exhibited the highest values with *K*_L–Z_ > 4. The morpholino derivatives **18** and **18**_**D**_ showed a substantial shift to lower values (*K*_L–Z_ < 0.2), due in part to the inductive electron-withdrawing properties of the oxygen atom.^29^ Like the TMR scaffold, where the deuterated **1**_**D**_ showed a higher Φ_f_ value compared to the parent dye **1** (Φ_f,D_/Φ_f,H_ = 1.22), other deuterated rhodamines showed an increase in Φ_f_, including dyes containing pyrrolidine (**5**/**5**_**D**_; Φ_f,D_/Φ_f,H_ = 1.14), piperidine (**17**/**17**_**D**_; Φ_f,D_/Φ_f,H_ = 1.50), and morpholine (**18**/**18**_**D**_; Φ_f,D_/Φ_f,H_ = 1.18; Table 1). Notably, the azetidine-*d*_6_ compound (**6**_**D**_) showed no improvement in Φ_f_ over **6** (Table 1). This result is consistent with the hypothesis that the azetidine and deuterium substitutions both suppress TICT. Because the azetidine modification largely rescues quantum yield (cf. **4** and **6**, Figure 1b, c), addition of deuterium does not offer further improvement to Φ_f_.

**Figure 3:**
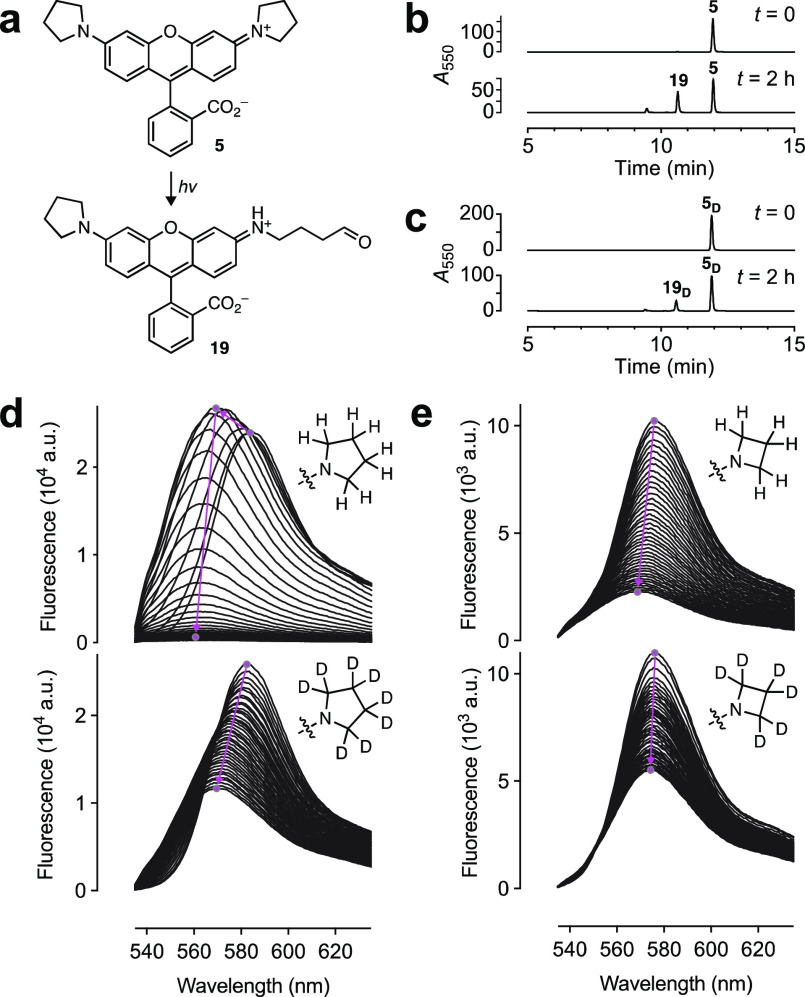
Photostability and chromostability of **5**, **5_D_**, **6**, and **6_D_**. (a) Photochemical dealkylation of **5** to form aldehyde **19**. (b, c) LC–MS traces of **5** (b) and **5**_**D**_ (c) before and after photobleaching. (d, e) Sequential fluorescence emission spectra of (d) **5** and **5**_**D**_ or (e) **6** and **6**_**D**_ during photobleaching using a 532 nm laser (0.96 W/cm^2^; 40 spectra taken over 40 min). The magenta arrows highlight the shift in λ_em_ and intensity over time.

The photostability and chromostability of the brightest variants—those containing azetidine and pyrrolidine substituents—were then evaluated. Based on the photophysics of **1** (Figure 1a), dealkylation of pyrrolidinyl **5** should yield aldehyde **19** (Figure 3a). This was confirmed by LC–MS, which also revealed slower dealkylation of the deuterated **5**_**D**_ (Figure 3b, c, Figure S4a). Monitoring the bleaching of **5** by fluorescence emission gave a complicated set of spectra with an initial increase in intensity along with a hypsochromic spectral shift followed by rapid bleaching (Figure 3d). This result can be explained by the relatively low Φ_f_ of **5**; dealkylation of this unoptimized dye yields the brighter trialkyl species **19**, which then rapidly bleaches. Monitoring bleaching by absorption spectroscopy circumvents this Φ_f_ confound and shows a steady decrease in ε and blue-shift in λ_abs_ (Figure S4b). The bright JF_549_ (**6**) displayed a constant rate of bleaching with a concomitant shift in λ_em_ (Figure 3e). In comparison, the deuterated rhodamine congeners **5**_**D**_ and **6**_**D**_ bleached slower and exhibited higher resistance to undesirable dealkylation evidenced by the reduced shifts in λ_abs_ and λ_em_ (Figure 3d, e, Figure S4c, d). Based on these results, dyes **6**_**D**_ and **5**_**D**_ were given the monikers “JFX_549_” and “JFX_554_”, respectively, to denote the extra stability afforded by the deuterated auxochromes.

**Figure 4:**
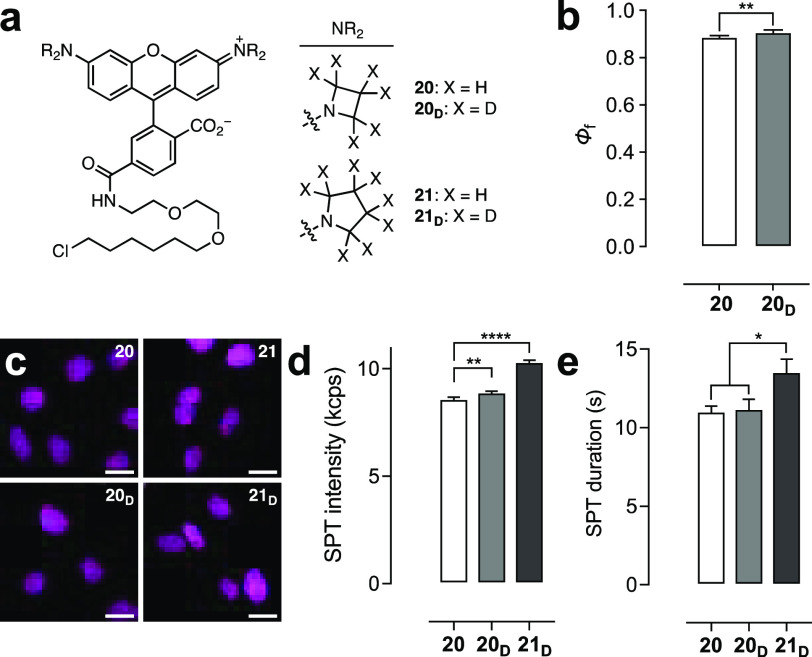
Performance of rhodamine ligands. (a) Structures of HaloTag ligands **20**, **20**_**D**_, **21**, and **21**_**D**_. (b) Φ_f_ of HaloTag protein conjugates of **20** and **20**_**D**_. (c) Confocal microscopy images of live U2OS cells expressing HaloTag–histone H2B incubated with HaloTag ligands **20**, **20**_**D**_, **21**, and **21**_**D**_ (200 nM, 2 h); ex/em = 561 nm/565–632 nm; scale bars: 21 μm. (d, e) SPT intensity (kilocounts per second, kcps) (d) or duration (s) (e) from cells labeled with **20**, **20**_**D**_, or **21**_**D**_. All error bars: SEM.

The effect of deuteration on the properties of fluorophore:protein conjugates was then evaluated to determine if the improvements observed for the free dyes would translate to superior performance as fluorescent labels. The HaloTag^30^ ligand of **6** (JF_549_–HaloTag ligand, **20**) was previously synthesized starting from a 6-carboxyfluorescein derivative.^6^ This approach was used to prepare the JFX_549_–HaloTag ligand (**20**_**D**_) and JFX_554_–HaloTag ligand (**21**_**D**_), along with the HaloTag ligand of **5** (**21**; Figure 4a, Scheme S1). Comparison of the HaloTag conjugates of **20** and **20**_**D**_*in vitro* revealed a small but significant increase in Φ_f_ for the **20**_**D**_:HaloTag conjugate (Φ_f_ = 0.89) compared to the nondeuterated **20**-labeled protein (Φ_f_ = 0.87; Figure 4b). For the free dyes, **6**_**D**_ shows a slightly smaller Φ_f_ compared to **6** (Table 1), so this result suggests that deuteration suppresses a protein-bound-specific mode of nonradiative decay. Photobleaching experiments using the HaloTag protein labeled with ligands **20**, **20**_**D**_, **21**, and **21**_**D**_ revealed that the HaloTag conjugates were more photostable and chromostable than the free dyes, demonstrating that the local environment around the fluorophore can substantially affect photophysics. Still, the HaloTag conjugates of deuterated dyes **20**_**D**_ and **21**_**D**_ exhibited slower bleaching compared to the HaloTag-bound **20** and **21** (Figure S5a, b).

The rhodamine-based HaloTag ligands **20**, **20**_**D**_, **21**, and **21**_**D**_ were then evaluated in cellular experiments. All four ligands could label HaloTag–histone H2B fusions in living cells (Figure 4c) with no substantial differences in loading kinetics (Figure S5c). **20**_**D**_ gave a significantly longer fluorescence lifetime (τ) than **20** in live cells (Figure S5d), in line with the *in vitro* Φ_f_ measurements (Figure 4b). Photobleaching experiments in fixed cells demonstrated slightly slower bleaching for the deuterated **20**_**D**_ and **21**_**D**_ compared to cells labeled with **20** and **21** (Figure S5f, g). The utility of these dyes in live-cell single-particle tracking (SPT) was then assessed. In initial experiments, pyrrolidine compound **21** showed significantly poorer performance compared to **20** with substantially shorter average duration of individual molecules (Figure S5e). We therefore focused on the JF_549_–HaloTag ligand (**20**), JFX_549_–HaloTag ligand (**20**_**D**_), and JFX_554_–HaloTag ligand (**21**_**D**_) because the free fluorophores **5**_**D**_, **6**, and **6**_**D**_ exhibit the highest molecular brightness (ε × Φ; Table 1). Deuteration elicited modestly higher fluorescence intensity in cells for the azetidine **20**_**D**_ (8.91 ± 0.05 kcps; mean ± SEM) compared to **20** (8.60 ± 0.07 kcps) under equivalent imaging conditions (Figure 4d). Compound **20**_**D**_ showed no significant improvement in mean duration (11.2 ± 0.6 s; Figure 4e) over **20** (11.0 ± 0.4 s), indicating that in this case the higher photostability observed *in vitro* and in fixed cells does not translate to the live-cell environment. Remarkably, the deuterated pyrrolidine rhodamine ligand **21**_**D**_ exhibited significantly higher intensity (10.33 ± 0.07 kcps) and track length (13.6 ± 0.8 s) compared to azetidines **20** and **20**_**D**_, making JFX_554_ an attractive new label for cellular imaging.^31^

This deuteration strategy was then applied to other fluorophores, including coumarins (**22**–**22**_**D**_), phenoxazines (**23**–**23**_**D**_), and fluorinated rhodamines (**24**–**24**_**D**_, **25**–**25**_**D**_; Table 2, Scheme S2).^6,11^ Deuteration increased Φ_f_ for both the pyrrolidinyl coumarin (**22**/**22**_**D**_; Φ_f,D_/Φ_f,H_ = 1.22) and pyrrolidinyl phenoxazine (**23**/**23**_**D**_; Φ_f,D_/Φ_f,H_ = 1.38). The fluorinated rhodamines exhibited more nuanced behavior with the azetidinyl dyes **24** and **24**_**D**_ showing high Φ_f_ values of 0.83 and 0.88, respectively; **24**_**D**_ displayed similar properties to JF_549_ (**6**). The pyrrolidinyl dyes **25** and **25**_**D**_ exhibited considerably lower quantum yields, however, with Φ_f_ values of 0.30 and 0.37, respectively. This result is consistent with the TICT mechanism (Figure 1a). The higher electron donor strength of the pyrrolidine substituent, combined with the higher electron acceptor strength of the fluorinated xanthene system, promotes electron transfer and decreases Φ_f_; the deuterated pyrrolidine in **25**_**D**_ partially rescues Φ_f_.

**Table 2:**
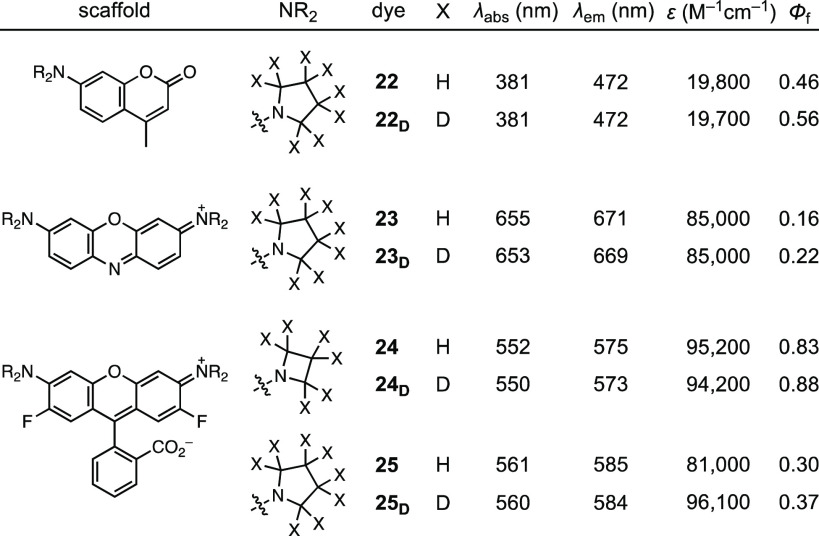
Spectral Properties of Other Deuterated Dyes^a^

a All values in 10 mM HEPES, pH 7.3.

Deuteration of red-shifted rhodamine variants was then explored through the synthesis of matched pairs of azetidinyl and pyrrolidinyl carborhodamines^10^ (**26**–**26**_**D**_, **27**–**27**_**D**_) and Si–rhodamines^11,32^ (**28**–**28**_**D**_, **29**–**29**_**D**_; Table 3, Scheme S2). For carborhodamines, deuteration increased Φ_f_ for both the azetidine-containing dyes (**26**/**26**_**D**_; Φ_f,D_/Φ_f,H_ = 1.10) and the pyrrolidinyl fluorophores (**27**/**27**_**D**_; Φ_f,D_/Φ_f,H_ = 1.30). The Si–rhodamine dyes were similar to the rhodamine series, however, with azetidines **28** (JF_646_) and **28**_**D**_ giving the same Φ_f_. The deuterated pyrrolidine **29**_**D**_ exhibited a higher Φ_f_ than the parent dye (**29**/**29**_**D**_; Φ_f,D_/Φ_f,H_ = 1.10). Like the rhodamine series (Table 1), deuteration had only minor effects on the lactone–zwitterion equilibrium, although the pyrrolidinyl dyes showed substantially higher *K*_L–Z_ values compared to the azetidinyl dyes. The brightest new fluorophores, **28**_**D**_ and **29**_**D**_, were named “JFX_646_” and “JFX_650_”, respectively.

**Table 3:**
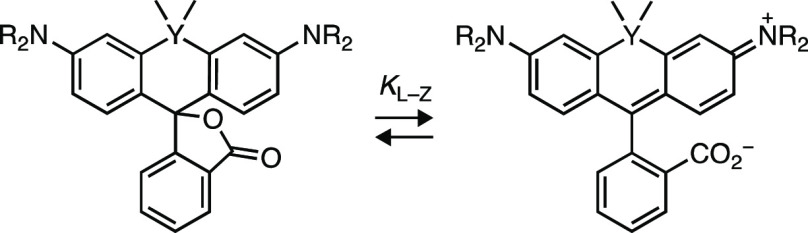

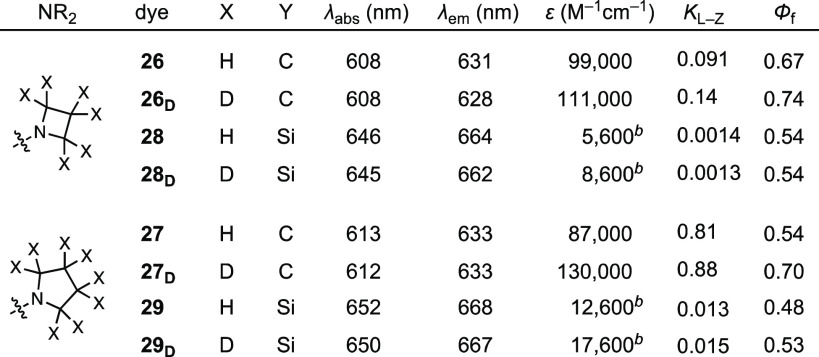
Spectral Properties of Red-Shifted Rhodamine Variants^a^

a All values in 10 mM HEPES, pH 7.3 except for *K*_L–Z_, which was measured in 1:1 v/v dioxane:H_2_O.

b ε > 150 000 M^–1^cm^–1^ in EtOH or TFE with 1% v/v TFA.

**Figure 5:**
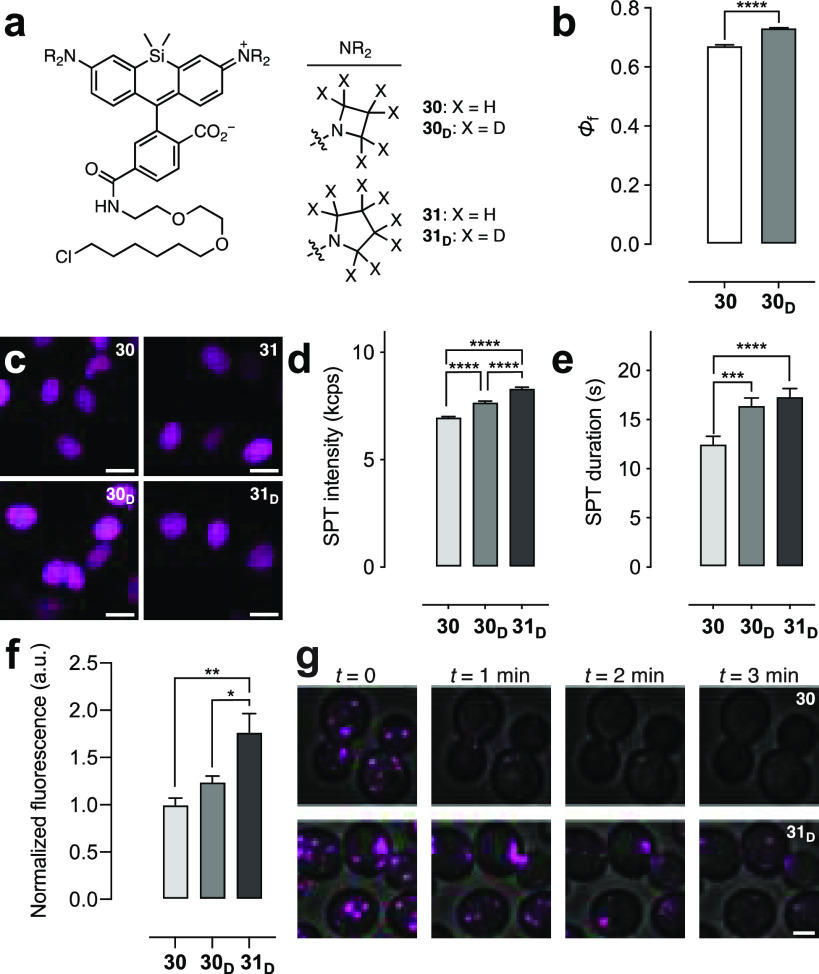
Performance of Si–rhodamine ligands. (a) Structures of HaloTag ligands **30**, **30**_**D**_, **31**, and **31**_**D**_. (b) Φ of HaloTag protein conjugates of **30** or **30**_**D**_. (c) Confocal microscopy images of live U2OS cells expressing HaloTag–histone H2B incubated with HaloTag ligands **30**, **30**_**D**_, **31**, and **31**_**D**_ (200 nM, 2 h); ex/em = 640 nm/656–700 nm; scale bars: 21 μm. (d, e) SPT intensity (kcps) (d) or duration (s) (e) from cells labeled with **30**, **30**_**D**_, or **31**_**D**_. (f) Intensity from *S. cerevisiae* labeled with **30**, **30**_**D**_, or **31**_**D**_ (1 μM, 30 min). (g) Image montage of *S. cerevisiae* labeled with **30** or **31**_**D**_; ex/em = 633 nm/650–795 nm; scale bar: 2 μm. All error bars: SEM.

The HaloTag ligands of the new Si–rhodamine compounds were then prepared (**30**_**D**_, **31**–**31**_**D**_) based on the previous synthesis of JF_646_–HaloTag ligand^6^ (**30**; Figure 5a, Scheme S3). Like the analogous rhodamine ligands, the Φ_f_ of the deuterated azetidine JFX_646_–HaloTag ligand (**30**_**D**_; Φ_f_ = 0.73) was significantly higher than the parent ligand **30** (Φ_f_ = 0.65) when conjugated to the HaloTag protein (Figure 5b) despite the equivalent Φ_f_ values of the free fluorophores **28** and **28**_**D**_ (Table 3); **30**_**D**_ also showed longer τ in cells (Figure S6a). All four ligands could label HaloTag fusions in live cells (Figure 5c) with similar loading profiles (Figure S6b). Photobleaching experiments in fixed cells showed higher stability for **30**_**D**_ and **31**_**D**_ compared with the nondeuterated molecules **30** and **31** (Figure S6c, d). Mirroring the rhodamine series (Figure 4d,e), compounds **30**, **30**_**D**_, and **31**_**D**_ were evaluated in live-cell SPT experiments because the free dyes **28**, **28**_**D**_, and **29**_**D**_ exhibited the highest Φ_f_ values among the Si–rhodamines (Table 3). The deuterated Si–rhodamine HaloTag ligands showed superior performance with ligand **28**_**D**_ showing higher mean intensity (7.69 ± 0.05 kcps; Figure 5d) and longer average track length (16.4 ± 0.8 s; Figure 5e) compared to compound **28** (6.98 ± 0.04 kcps, 12.5 ± 0.8 s). The JFX_650_–HaloTag ligand (**31**_**D**_) exhibited the best overall performance (8.33 ± 0.04 kcps, 17.5 ± 0.3 s). Moving beyond SPT, these Si–rhodamine ligands were evaluated in confocal imaging experiments using a strain of *S. cerevisiae* expressing a Sec7-GFP–HaloTag fusion to visualize the late Golgi apparatus. The results were consistent with the SPT experiments, with the deuterated JFX ligands **30**_**D**_ and **31**_**D**_ exhibiting significantly higher brightness (Figure 5f) and photostability (Figure 5g, Figure S6e, f, Movie S1) compared to the JF_646_ ligand **30**. The deuterated pyrrolidine JFX_650_–HaloTag ligand (**31**_**D**_) showed the best performance in the ensemble imaging in yeast.

## Conclusion

Taking into account the known photophysical processes of rhodamines, we hypothesized that deuteration of the *N*-alkyl auxochromes of rhodamine dyes would improve brightness, chromostability, and photostability (Figure 1a). We based this idea on the isotope effects that deuterium exerts on electron and proton transfer processes and not the alteration of vibrational modes as previously proposed.^21^ This hypothesis was tested first using the classic fluorophore TMR (**1**; Figure 2) and then by synthesizing other deuterated rhodamine dyes. We discovered that deuteration maintained or improved Φ_f_ (Table 1) and enhanced chromo- and photostability (Figure 3). Synthesis of HaloTag ligands showed that deuterated rhodamines were superior labels for live-cell SPT experiments (Figure 4). This deuteration strategy was generalizable to other fluorophore classes (Table 2, Table 3), and the deuterated Si–rhodamines also showed improved performance in cellular imaging experiments (Figure 5).

Overall, this work establishes deuteration of *N*-alkyl auxochromes as a general strategy for improving the properties of small-molecule fluorophores. This work also demonstrates the predictive value and caveats of *in vitro* photobleaching experiments for the evaluation of probes intended for living cells. For the azetidine- and pyrrolidine-containing fluorophores, we observed variation in the relative performance of the free dyes **5**, **5**_**D**_, **6**, and **6**_**D**_ in solution (Figure 3d, e), the corresponding ligands **20**, **20**_**D**_, **21**, and **21**_**D**_ on the HaloTag protein (Figure S5a,b), the ligands in fixed cells (Figure S5f, g), and the ligands in living cells (Figure 4d, e). Although the general trend of deuteration improving brightness and photostability held throughout these experiments, evaluation in living cells was needed to reveal the superior performance of pyrrolidine-containing compounds JFX_554_ and JFX_650_. These new dyes can be used as direct replacements of the original Janelia Fluor 549 and Janelia Fluor 646 in bioimaging experiments.^6^ Future work will focus on utilizing these improved labels in other advanced imaging techniques, combining this deuteration strategy with complementary fluorophore tuning methods,^7,8^ and extending this straightforward isotope approach to improve other chromophores containing *N*-alkyl auxochromes.

## Supporting Information Available

Synthetic schemes, supplementary figures, experimental details, and characterization for all new compounds (PDF)
Movie S1 (MP4)
