# Supplementary material for: A General Method to Improve Fluorophores Using Deuterated Auxochromes
Source: JACS Au. 2021 Apr 23;1(5):690–6. doi: 10.1021/jacsau.1c00006 (PMC8154212; doi:10.1021/jacsau.1c00006)
Supplement: Supplementary file 1 — au1c00006_si_001.pdf [file au1c00006_si_001.pdf]

SUPPORTING INFORMATION

A general method to improve fluorophores using deuterated auxochromes

Jonathan B. Grimm[1], Liangqi Xie[1], Jason C. Casler[2], Ronak Patel[1], Ariana N. Tkachuk[1], Natalie Falco[1], Heejun Choi[1], Jennifer Lippincott-Schwartz[1], Timothy A. Brown[1], Benjamin S. Glick[2], Zhe Liu[1], and Luke D. Lavis[1,]*

[1]*Janelia Research Campus, The Howard Hughes Medical Institute, 19700 Helix Drive, Ashburn, VA 20147, USA*

[2]*Department of Molecular Genetics and Cell Biology, University of Chicago, 920 East 58th Street, Chicago, IL 60637, USA*

*Email: lavisl@janelia.hhmi.org

EXPERIMENTAL INFORMATION

Page	Contents
S2	Figures S1–S6
S7	Schemes S1–S3
S10	Experimental Details for Spectroscopy and Imaging
S15	Experimental Details and Characterization Data for All Compounds
S34	References
S35	NMR and HPLC–MS

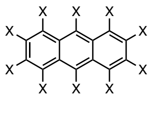

10, anthracene
$\Phi_{f,X=D}/\Phi_{f,X=H}$ = 0.89 (ethanol)

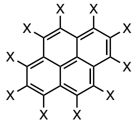

11, pyrene
$\Phi_{f,X=D}/\Phi_{f,X=H}$ = 0.83 (ethanol)

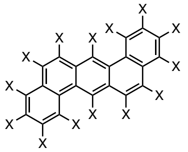

12, benzo[*k*]tetraphene
$\Phi_{f,X=D}/\Phi_{f,X=H}$ = 0.87 (ethanol)

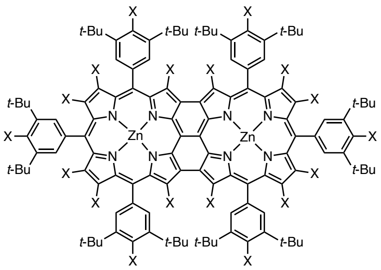

13
$\Phi_{f,X=D}/\Phi_{f,X=H}$ = 1.00 (CD_2Cl_2)

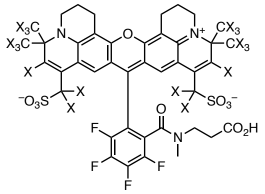

14
$\Phi_{f,X=D}/\Phi_{f,X=H}$ = 0.98 (H_2O)

Figure S1. Chemical structures and fluorescence quantum yields (Φ_f) of matched pairs of nondeuterated (X = H) and deuterated (X = D) fluorophores, including polycyclic aromatic compounds (**10**–**12**), a porphyrin (**13**), and a rhodamine (**14**).

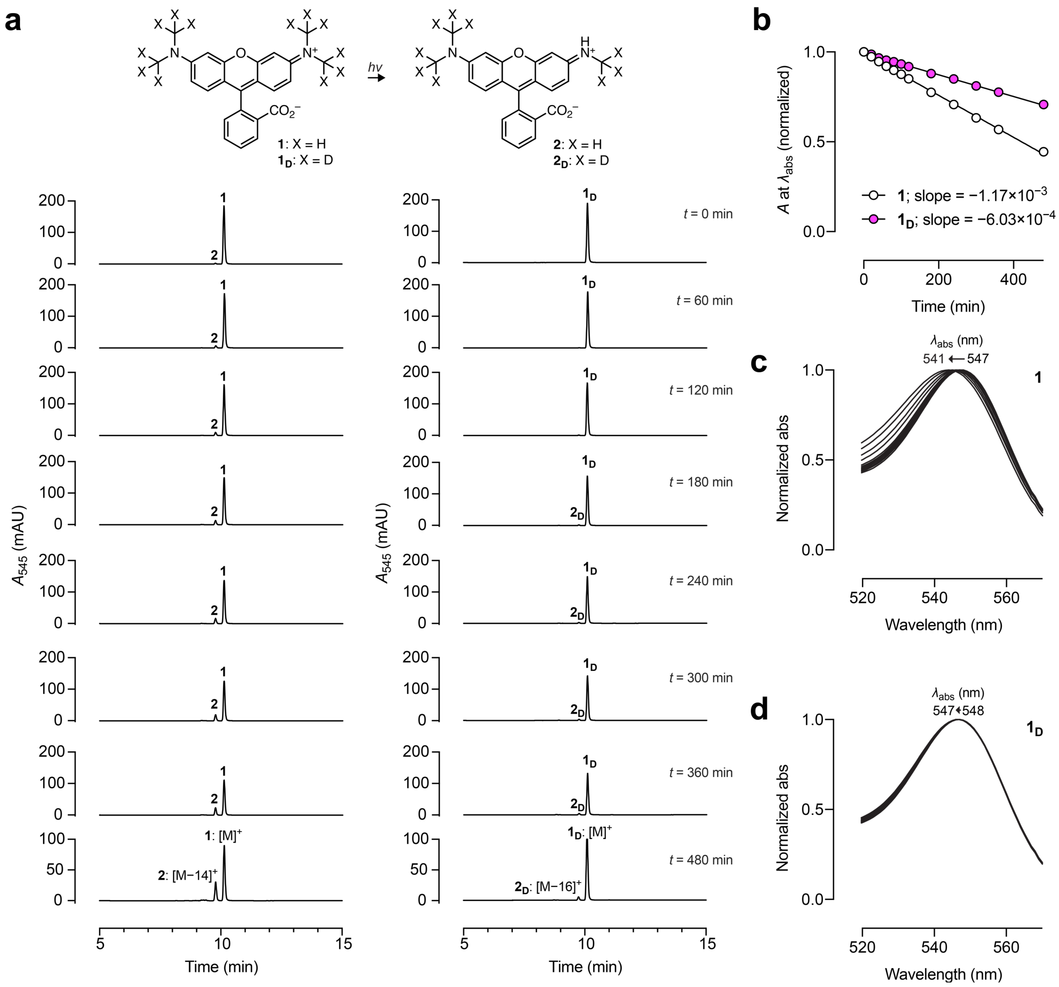

Figure S2. Photobleaching of TMR (1) and TMR-*d*$_{12}$ (1_D). (**a**) Photoinduced dealkylation of **1** or **1_D** to form **2** or **2_D**, respectively, and reaction monitoring by tandem liquid chromatography–mass spectrometry (LC–MS) confirming loss of methyl groups. (**b**) Relative absorption *vs.* time and initial slopes of **1** at λ_{abs} = 547 nm or **1_D** at λ_{abs} = 548 nm during photobleaching (560 nm; 1.02 W/cm^2); full spectra are given in Figure 2e,f. (**c,d**) Independently normalized absorption spectra over time for **1** (**c**) or **1_D** (**d**) during photobleaching (560 nm; 1.02 W/cm^2) to show improvement in chromostability upon deuteration of the *N*-methyl groups in **1_D**.

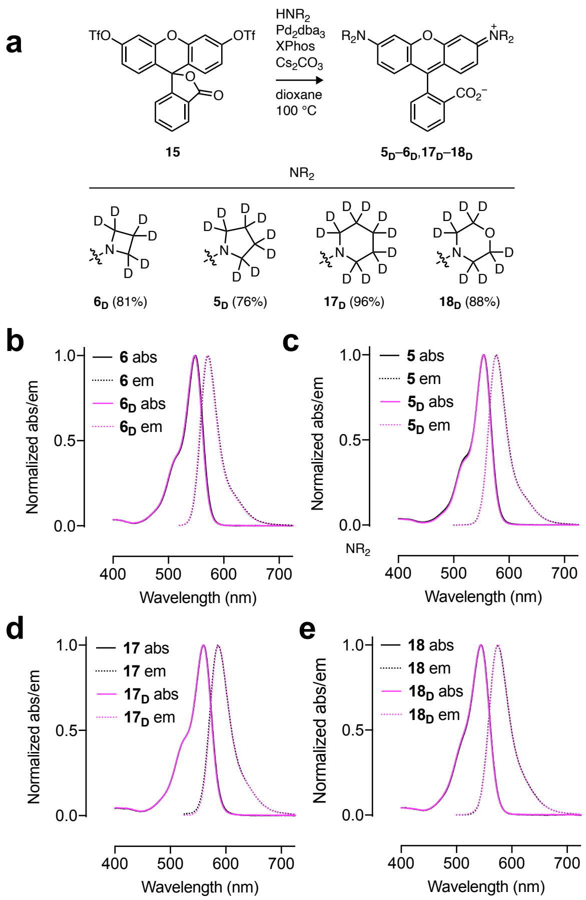

Figure S3. (**a**) Synthesis $\mathbf{5_D}$–$\mathbf{6_D}$ and $\mathbf{17_D}$–$\mathbf{18_D}$ from fluorescein ditriflate (**15**). (**b**–**e**) Normalized absorption (abs) and fluorescence emission (em) spectra for matched pairs of nondeuterated and deuterated rhodamine dyes: (**b**) **6** and $\mathbf{6_D}$; (**c**) **5** and $\mathbf{5_D}$; (**d**) **17** and $\mathbf{17_D}$; (**e**) **18** and $\mathbf{18_D}$.

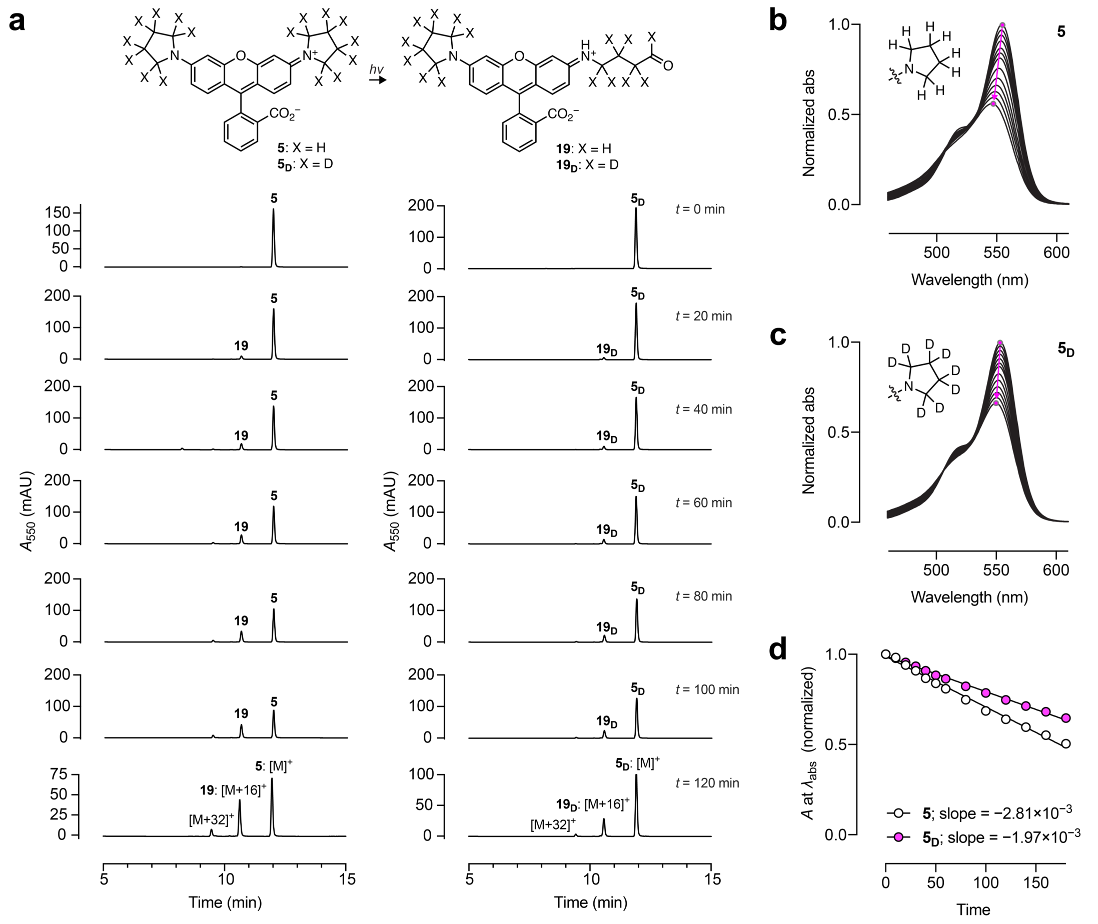

Figure S4. Photobleaching of 5 and 5_D. (**a**) Photoinduced dealkylation of **5** or **5_D** to form **19** or **19_D**, respectively, and reaction monitoring by tandem liquid chromatography–mass spectrometry (LC–MS) confirming addition of oxygen. (**b,c**) Sequential absorption spectra of **5** (**b**) and **5_D** (**c**) during photobleaching at 560 nm (1.02 W/cm^2). The magenta arrows highlight the shift in λ_{abs} and intensity over time. (**d**) Relative absorption *vs.* time and initial slopes of **5** at λ_{abs} = 553 nm or **5_D** at λ_{abs} = 554 nm during photobleaching (560 nm; 1.02 W/cm^2); full spectra are given in (**b,c**).

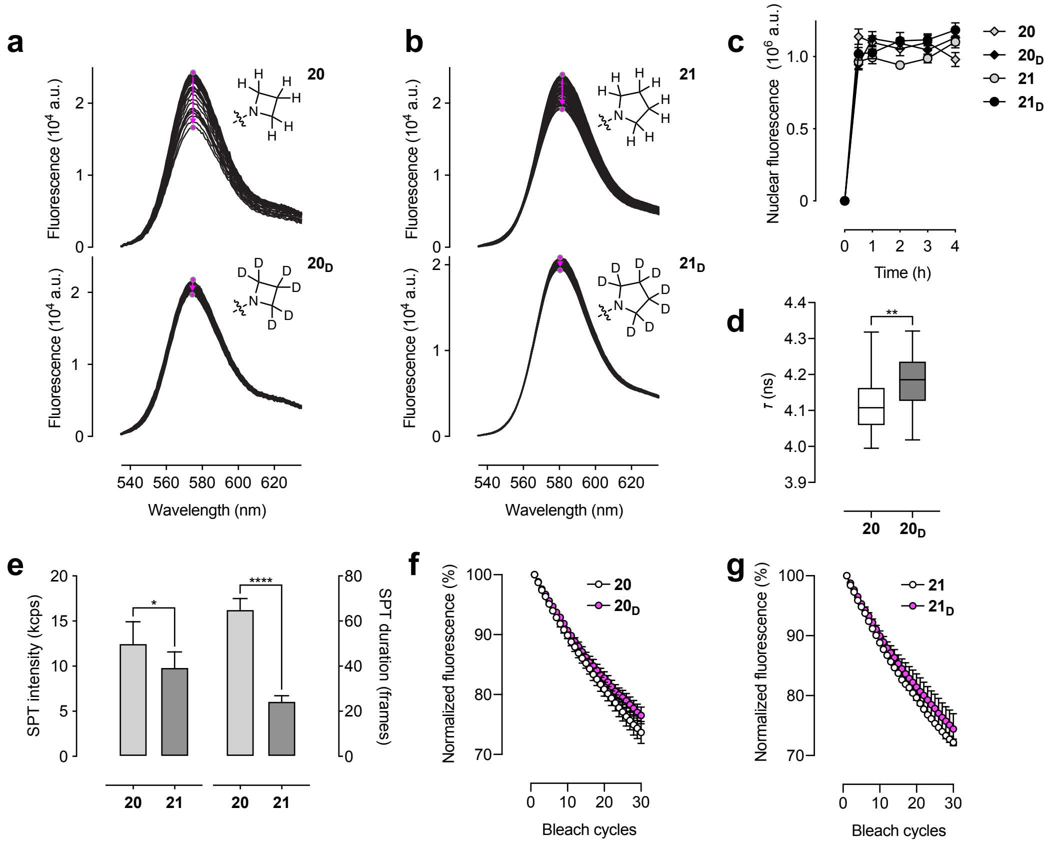

Figure S5. (**a**,**b**) Sequential fluorescence emission spectra of (**a**) **20**:HaloTag protein conjugate and **20**$_\mathbf{D}$:HaloTag protein conjugate or (**b**) **21**:HaloTag protein conjugate and **21**$_\mathbf{D}$:HaloTag protein conjugate during illumination; the magenta arrows show the shift in λ_{em} over time. (**c**) Nuclear fluorescence *vs.* time upon addition of HaloTag ligands **20**, **20**$_\mathbf{D}$, **21**, and **21**$_\mathbf{D}$ (200 nM) to live U2OS cells expressing HaloTag–histone H2B; error bars indicate SEM. (**d**) Plot of fluorescence lifetime (τ) of U2OS cells expressing HaloTag–histone H2B and labeled with HaloTag ligands **20** or **20**$_\mathbf{D}$; center line indicates median; box limits indicate upper and lower quartiles; whiskers indicate min–max. (**e**) Plot of average single-molecule intensity and trajectory length per cell in single-particle tracking experiments using JF_{549}-HaloTag ligand (**20**) or pyrrolidine-containing HaloTag ligand **21**; error bars indicate SEM. (**f,g**) Nuclear fluorescence during photobleaching in fixed U2OS cells expressing HaloTag–histone H2B and labeled with **20** and **20**$_\mathbf{D}$ (**f**) or with **21** and **21**$_\mathbf{D}$ (**g**).

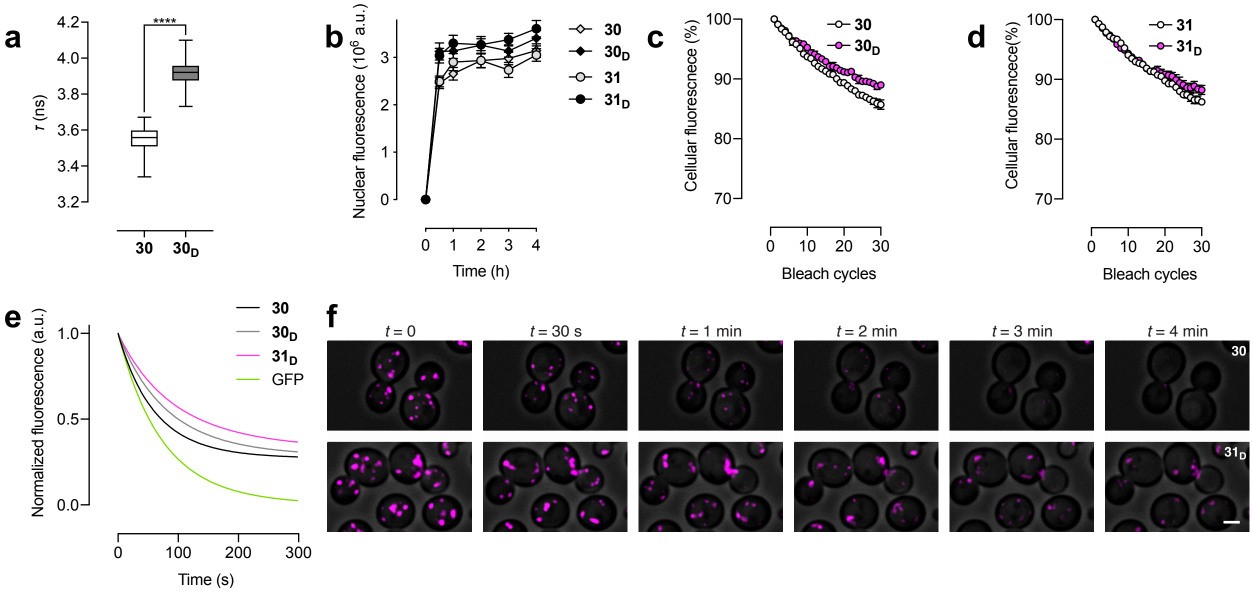

Figure S6. (**a**) Plot of fluorescence lifetime (τ) of U2OS cells expressing HaloTag–histone H2B and labeled with HaloTag ligands **30** or **30_D**; center line indicates median; box limits indicate upper and lower quartiles; whiskers indicate min–max. (**b**) Nuclear fluorescence *vs.* time upon addition of ligands **30**, **30_D**, **31**, or **31_D** (200 nM) to live U2OS cells expressing HaloTag–histone H2B; error bars indicate SEM. (**c,d**) Cellular fluorescence during photobleaching in fixed cells expressing HaloTag–histone H2B and labeled with (**c**) **30** and **30_D** or with (**d**) **31** and **31_D**. (**e**) Photobleaching plot of normalized fluorescence intensity *vs.* time of *S. cerevisiae* cells expressing Sec7–iGFP–HaloTag fusions and labeled with ligands **30**, **30_D**, or **31_D**. (**f**) Expanded image montage from Figure 5g/Movie S1 showing *S. cerevisiae* labeled with **30** or **31_D**; scale bar: 2 µm.

Scheme S1. Synthesis of HaloTag ligands **20_D**, **21**, and **21_D**.

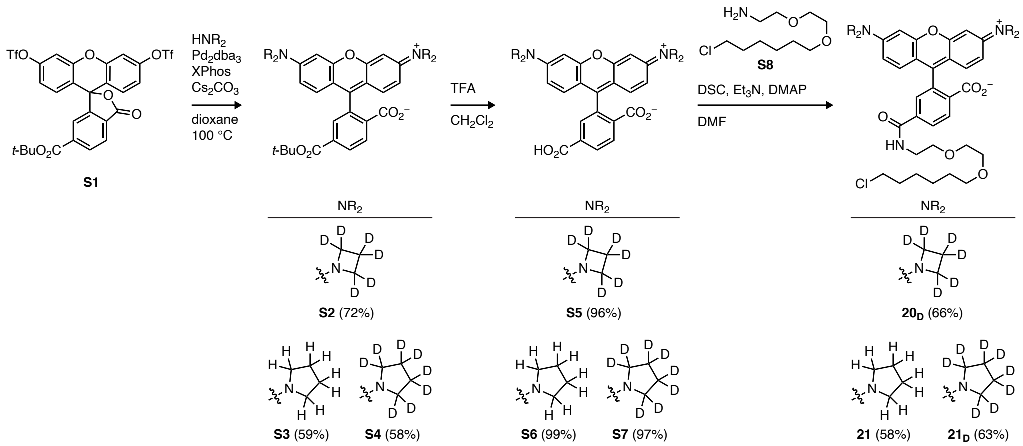

Scheme S2. Synthesis of nondeuterated and deuterated dyes: (**a**) coumarins, (**b**) phenoxazines, (**c**) fluorinated rhodamines, (**d**) carborhodamines, and (**e**) Si-rhodamines.

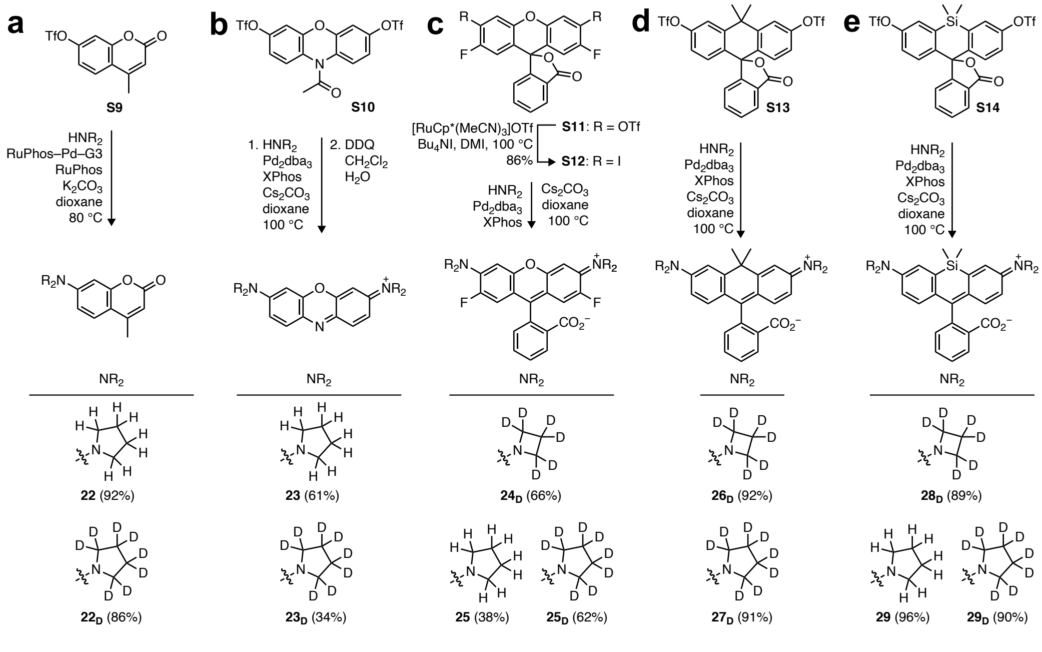

Scheme S3. Synthesis of HaloTag ligands $\mathbf{30_D}$, **31**, and $\mathbf{31_D}$.

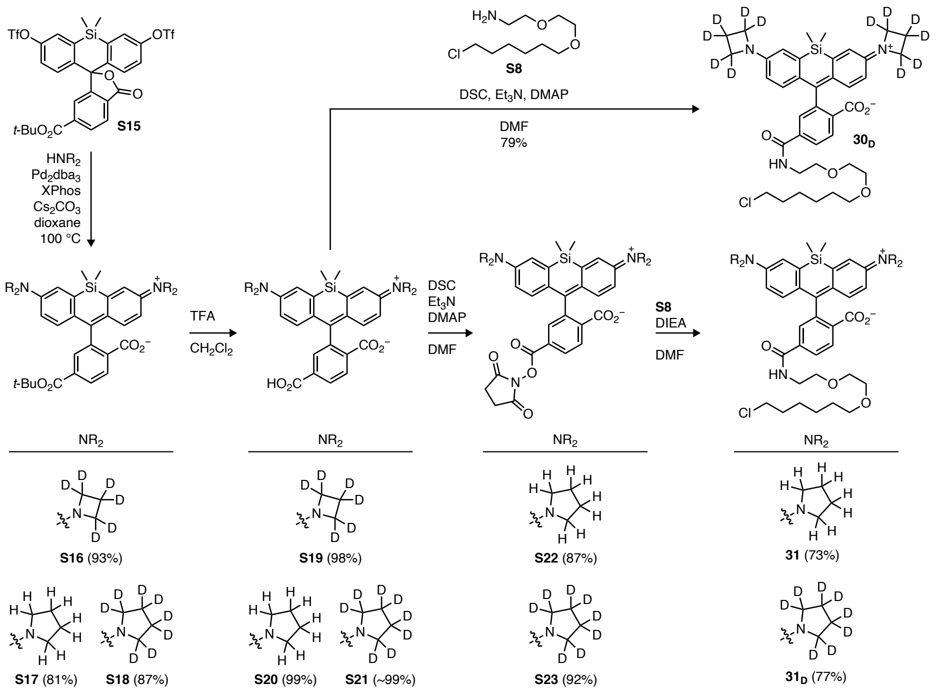

General Experimental Information for Spectroscopy and Imaging

Compound sources. Tetramethylrhodamine (**1**; Reference Standard Grade) was purchased from AAT BioQuest. Compounds **5**, **6**, **17**, **18**, **20**, **24**, **26**–**28**, and **30** were available from previous work.[1-4]

UV–vis and fluorescence spectroscopy (Figure 2b, Table 1, Table 2, Table 3, Figure S3b–e). Fluorescent molecules for spectroscopy were prepared as stock solutions in DMSO and diluted such that the DMSO concentration did not exceed 1% v/v. Spectroscopy was performed using 1-cm path length, 3.5-mL quartz cuvettes or 1-cm path length, 1.0-mL quartz microcuvettes from Starna Cells. All measurements were taken at ambient temperature (22 ± 2 °C). Absorption spectra were recorded on a Cary Model 100 spectrometer (Agilent). Fluorescence spectra were recorded on a Cary Eclipse fluorometer (Varian). Maximum absorption wavelength (λ_{abs}), extinction coefficient (ε), and maximum emission wavelength (λ_{em}) were measured in 10 mM HEPES, pH 7.3 buffer. Normalized spectra are shown for clarity.

Quantum yield determination (Table 1, Table 2, Table 3). All reported absolute fluorescence quantum yield values (Φ) were measured in our laboratory under identical conditions using a Quantaurus-QY spectrometer (model C11374, Hamamatsu). This instrument uses an integrating sphere to determine photons absorbed and emitted by a sample. Measurements were carried out using dilute samples ($A < 0.1$) and self-absorption corrections[5] were performed using the instrument software. Reported values are averages ($n = 3$).

Determination of $K_{L\text{–}Z}$ (Table 1, Table 3). We calculated $K_{L\text{–}Z}$ using the following equation[6]: $K_{L\text{–}Z} = (\varepsilon_{dw}/\varepsilon_{max})/(1 - \varepsilon_{dw}/\varepsilon_{max})$. ε_{dw} is the extinction coefficient of the dyes in a 1:1 v/v dioxane:water solvent mixture; this dioxane–water mixture was chosen to give the maximum spread of $K_{L\text{–}Z}$ values[7]. ε_{max} refers to the maximal extinction coefficients measured in different solvent mixtures empirically determined depending on dye type: 0.1% v/v TFA in ethanol for the Si-rhodamines **28** and **28_D**; 0.1% v/v trifluoroacetic acid (TFA) in 2,2,2-trifluoroethanol (TFE) for all the other rhodamine variants.

Photostability and chromostability measurements of dyes by absorption spectroscopy and LC–MS (Figure 2c–f, Figure 3b,c, Figure S2, Figure S4). To test the stability of the free dyes, solutions of **1**, **1$_{\mathbf{D}}$**, **5**, and **5$_{\mathbf{D}}$** (10 μM) were prepared in 10 mM HEPES, pH 7.3. For each dye, two 3 mL samples were prepared in 1-cm path length, 3.5-mL quartz cuvettes (Starna Cells). Samples were placed under a Mightex super high-powered LED collimator source (560 nm broadband, 110 nm FWHM, 22 mm aperture, 3500 mW typical output power), which was vertically mounted 7 cm above a mirrored disc (d = 2 cm) on a black acrylic base. The LED was driven by a ThorLabs DC2200 high-power 1-channel LED driver (10 A, 100% LED brightness, pulse mode, 1000 ms on-time, 0.001 ms off-time). Samples were bleached for the indicated amount of time with a measured irradiance of 1.02 W/cm^2 (ThorLabs PM100A), pausing at regular time intervals (10–20 min) to record the absorption spectrum on a Cary Model 100 spectrometer and remove a small aliquot for LC–MS analysis (Agilent Eclipse XDB 4.6 × 150 mm 5 μm C18 column; 20 μL injection; 10–95%

MeCN/H_2O, linear gradient, with constant 0.1% v/v TFA additive; 20 min run; 1 mL/min flow; ESI; positive ion mode; detection at 545 or 550 nm).

Photostability and chromostability measurements of dyes and HaloTag conjugates by fluorescence emission (Figure 3d,e, Figure S5a,b). To test the stability of the free dyes, solutions of **5**, **5_D**, **6**, and **6_D** (1 μM) were prepared in 10 mM HEPES, pH 7.3. To test the stability of the HaloTag conjugates, HaloTag protein was used as a 200 μM solution in PBS, pH 7.4. Solutions of HaloTag ligands **20**, **20_D**, **21**, and **21_D** (5 μM) were prepared in 10 mM HEPES, pH 7.3 containing 0.1 mg·mL^{-1} CHAPS. An aliquot of HaloTag protein (2.0 equiv, 10 μM final [HaloTag]) was added to each sample, and the resulting mixture was incubated at 4 °C overnight. Stability measurements were performed after the HaloTag conjugate solutions were diluted 5× (1 μM final [ligand]) into 10 mM HEPES, pH 7.3 buffer solution. An aliquot of these solutions was added to each well of 10-well Teflon printed glass slide with 1.5 mm well diameter (Tekdon, Inc.) and sealed with a coverslip using vacuum grease. The hydrophobic coating of the slide resulted in formation of macrodroplets of aqueous dye solution in each well. Fluorophore bleaching was measured by illuminating an entire droplet using an upright microscope (Zeiss Axio Observer Z2) and a 5×/0.25 NA objective. The fluorophore and HaloTag conjugate solutions were bleached using an upright microscope (Zeiss Axio Observer Z2) 5×/0.25 NA objective. Light illumination was provided by a 532 nm laser (Compass 215M-75; Coherent) at 18 mW power (intensity 0.96 W/cm^2). Fluorescence emission was collected using a 590/104 filter and emission spectra were measured using a fiber coupled spectrometer (QE 65000; Ocean Optics). Each sample was continuously bleached for 40 min with fluorescence spectra acquired every 1 min. Spectra are averages (n = 2).

Quantum yield determination of HaloTag protein conjugates (Figure 4b, Figure 5b). HaloTag protein was used as a 100 μM solution in 75 mM NaCl, 50 mM TRIS·HCl, pH 7.4 with 50% v/v glycerol (TBS–glycerol). A solution of HaloTag ligand **20**, **20_D**, **30**, or **30_D** (5 μM) was prepared in 10 mM HEPES, pH 7.3 containing 0.1 mg·mL^{-1} CHAPS. An aliquot of HaloTag protein (1.5 equiv, 7.5 μM final [HaloTag]) was added and the resulting mixture was incubated until consistent absorption signal was observed (60–120 min). After incubation, this solution was diluted 5–10-fold in 10 mM HEPES, pH 7.3 containing 0.1 mg·mL^{-1} CHAPS to give 500 nM dye concentration, which is optimal for quantum yield determination (n = 14).

Confocal fluorescence microscopy (Figure 4c, Figure 5c). U2OS cells (ATCC) with an integrated histone H2B–HaloTag fusion protein expressing plasmid via the piggyBac transposon system were cultured in Dulbecco's modified Eagle medium (DMEM, phenol red-free; Life Technologies) supplemented with 10% v/v fetal bovine serum (FBS, Life Technologies), 1 mM GlutaMAX (Life Technologies) and maintained at 37 °C in a humidified 5% (v/v) CO_2 environment. These cell lines were kept under the selection of 500 μg/mL Geneticin (Life Technologies) and undergo regular mycoplasma testing by the Janelia Cell Culture Facility. Cells were incubated with HaloTag ligands **20**, **20_D**, **21**, **21_D**, **30**, **30_D**, **31**, or **31_D** (200 nM, 2 h) and washed 3× with dye-free media. Cells were imaged on a Zeiss LSM 800 confocal microscope with a Plan APO 63×/1.4 NA oil DIC M27 objective. Ligands **20**, **20_D**, **21**, and **21_D** were excited with a 561nm laser and emission light was collected at 565–632 nm. Ligands **30**, **30_D**, **31**, and **31_D** were excited

with a 640 nm laser, and emission light was collected at 656–700 nm. The confocal images were processed using FIJI[8] and displayed as maximum intensity image projections.

Dye loading kinetics (Figure S5c, Figure S6b). Live U2OS cells stably expressing histone H2B–HaloTag fusion proteins were labeled over a time course of 0–4 h with 200 nM of HaloTag ligands **20**, **20_D**, **21**, **21_D**, **30**, **30_D**, **31**, or **31_D**. Cells were briefly washed 3× with dye-free media and immediately imaged live using widefield microscopy on a Nikon Eclipse Ti, Plan APO λ 20×/0.75 air objective. Fluorescence was quantified from the average of the summed intensity of nuclear signals in single-plane widefield images analyzed using Nikon NIS-Elements AR software. Fields of view were chosen randomly; n = 100 nuclear signals per compound.

In-cell fluorescence lifetime (τ) measurements (Figure S5d, Figure S6a). U2OS cells stably expressing histone H2B–HaloTag protein fusions were plated on a coverslip pre-coated with human fibronectin (Millipore). After 24 h, the cells were incubated with 100 nM of dyes **20**, **20_D**, **30**, or **30_D** for 30 min in DMEM (Corning), supplemented with 10% v/v FBS (Corning), 2 mM L-Glutamine, 100 U/mL penicillin and 100 µg/mL streptomycin at 37 °C and 5% CO_2. Prior to imaging the cells were briefly washed 3× with dye-free media. These cells were imaged using a Zeiss 880 laser scanning confocal microscope equipped with Ti:Sapphire laser (Coherent Chameleon). The labeled U2OS cell samples were excited with 850 nm (**20** and **20_D**) or 830 nm (**30** and **30_D**). The excitation laser was filtered through a short-pass filter (675LP). The emitted light passed through a dichroic (DIC560) and collected by the PMA hybrid detector (PicoQuant) with bandpass filters (BPF600/50 for **20** and **20_D**; no filter for **30** and **30_D**) and fed to the single-photon counting board (TimeHarp 260, PicoQuant) and monitored at 80 MHz. The resulting histogram was fit to a single exponential decay function using SymPhoTime 64 (PicoQuant); n = 30 for **20** and **20_D**, n = 27 for **30**, and n = 29 for **30_D**.

Initial comparison of HaloTag ligands 20 and 21 in single-particle tracking (SPT) experiments (Figure S5e). U2OS cells stably expressing histone H2B–HaloTag protein fusions were plated on a coverslip pre-coated with human fibronectin (Millipore). After 24 h, the cells were incubated with 1 nM of either **20** or **21** for 30 min in (?) DMEM (Corning) supplemented with 10% v/v FBS (Corning), 2 mM L-Glutamine, 100 U/mL penicillin and 100 µg/mL streptomycin at 37 °C and 5% CO_2. Prior to imaging the cells were briefly washed 3× with dye-free media. These cells were imaged using a Zeiss Elyra microscope equipped with 561 nm laser excitation and a 100× Plan-Apochromat 1.45 NA objective (Zeiss) and EMCCD (Andor). The cells were illuminated using oblique illumination and spt-*d*STORM was performed as previously described.[9] The emitted light was collected with a multi-bandpass filter (BP420-480 + BP570-640 + LP740). The single particles were imaged at 20 Hz and trajectories were mapped using TrackMate. The average trajectory length (*i.e.*, duration time) per cell was quantified from all the trajectories with the maximum displacement r < 400 nm within a single step. SPT intensity was quantified using Zeiss Zen Black for the average intensity of all single-particle emitters inside the cells. For SPT intensity, n = 7 cells using **20** and n = 8 cells using **21**. For SPT duration time, n = 7 cells using **20** and n = 16 cells using **21**.

Single-particle tracking (SPT) experiments (Figure 4d,e, Figure 5d,e). SPT experiments were carried out as previously described.[10] JM8.N4 mouse embryonic stem cells (ESCs) stably expressing histone H2B–HaloTag fusion proteins were generated by coexpressing the PiggyBac EF1a-H2B-HaloTag-IRES-Neo vector with the super piggyBac transposase as described previously,[11] followed by G418 selection (500 µg/mL) for 2 weeks and verified by FACS sorting/confocal imaging staining with JF_{549}-HaloTag ligand (**20**; 100 nM, 30 min). Cells were seeded on 25 mm #1.5 coverglass pre-cleaned with KOH and ethanol and coated with Matrigel according to the manufacturer's instruction. All live cell imaging experiments were conducted using an ESC imaging medium composed of FluoroBrite DMEM (Thermo Fisher Scientific) plus 15% v/v ESC-qualified FBS, 1× GlutaMax, 1× NEAA, 0.1 mM 2-mercaptoethanol, and LIF. To achieve sparse labeling, the histone H2B–HaloTag ESCs were stained with 2 nM of HaloTag ligands **20**, **20_D**, **21_D**, **30**, **30_D**, or **31_D** for 15 min at 37 °C with 5% v/v CO_2 and then washed with dye-free media 3× for 30 min each wash. To quantify the brightness and photostability of the HaloTag ligands, labeled cells were mounted onto a high speed motorized Nikon Eclipse Ti-E inverted microscope equipped with the following: 100× Apo TIRF 1.49 NA objective with a correction collar; four excitation laser lines (405/488/561/642 nm) and matching TIRF quad cube (405/488/561/640 nm reflection bands); automatic TIRF illuminator with motorized X axis and manual Y axis for beam positioning and focus: perfect focus 3 system; Triple DU-897 iXon Ultra EMCCD cameras on a Cairn Tri-cam emission splitter with filters 525/50 (GFP) 600/50 (RFP) and 705/72 (Cy5); humidified incubation chamber maintained at 37 °C with 5% v/v CO_2 (Tokai Hit). SPT was performed at 2 Hz for ~500 frames using `10% of maximal excitation power. The excitation laser was controlled by an acousto-optic tunable filter (AOTF) and reflected into the objective by a multi-band dichroic (405/488/561/633 BrightLine quad-band bandpass filter; Semrock). HaloTag ligands **20**, **20_D**, and **21_D** were illuminated with 561 nm laser, whereas **30**, **30_D**, and **31_D** were excited with a 641 nm laser. To minimize drift during imaging, the incubation chamber was fully thermally equilibrated and all imaging was conducted in an isolated ultra-clean room with minimal mechanical vibrations. The microscope, laser lines, and camera integration were controlled using the Nikon NIS-Elements software. We performed two independent biological replicates each with at least eight cells. Cells with obvious movement during acquisition were removed from further analysis. SMT analysis was based on multiple target tracing[12] (MTT) with tracking parameters similar to previous work.[10] For SPT brightness (kilocounts per second, kcps), intensity values from the first frame of the imaging experiment were analyzed: n = 3351 single-molecule events using **20**; n = 3838 single-molecule events using **20_D**; n = 4724 single-molecule events using **21_D**; n = 5523 single-molecule events using **30**; n = 3295 single-molecule events using **30_D**; n = 7125 single-molecule events using **31_D**. For SPT duration (s), the single-molecule trajectories of each cell were combined and a survival curve was generated based on the 1-cumulative distribution function (1-CDF) histogram and mathematical fitting (MATLAB) as previously described.[10] Histone H2B molecules were considered immobile under the imaging conditions and disappearance from the detection plane occurs primarily by photobleaching. Thus, the dissociation rate from this analysis equals the inverse of the fluorophore duration time, allowing comparison of photobleaching; n = 15 cells using **20**; n = 10 cells using **20_D**; n = 11 cells using **21_D**; n = 14 cells using **30**; n = 13 cells using **30_D**; n = 12 cells using **31_D**.

Fixed cell photobleaching experiments (Figure S5f,g, Figure S6c,d). U2OS cells expressing HaloTag–histone H2B fusion protein were co-fixed and labeled by incubation of 4% w/v PFA and 200 nM of ligands **20**, **20_D**, **21**, **21_D**, **30**, **30_D**, **31**, or **31_D** in 0.1 M phosphate buffer. Cells were washed 3× in phosphate-buffered saline and then imaged using a Leica SP8 Falcon confocal microscope with an HC PL-APO 86×/1.20 water objective. To measure photobleaching, we used the tunable white light laser (WLL) excitation on the Leica SP8 Falcon confocal microscope to excite dyes at their λ_{abs} in constant power mode; $n = 3$.

Imaging in yeast (Figure 5f,g, Figure S6e,f). A *Saccharomyces cerevisiae* strain expressing the late Golgi marker Sec7-iGFP-HaloTag fusion from the native locus was grown shaking at room temperature to mid-log phase in NSD. Cells were incubated with HaloTag ligands **30**, **30_D**, or **31_D** (1 µM diluted from a 1 mM stock in DMSO; 30 min), filter washed to remove excess dye, and adhered to a concanavalin A coated confocal dish. The cells were then imaged on a Leica SP8 laser scanning confocal microscope. GFP was excited at 488 nm and emission light was collected from 495–550 nm. Fluorophores **30**, **30_D**, or **31_D** were excited at 633 nm and collected from 650–795 nm under identical conditions. Movie S1 is a combination of representative average projected z-stacks of cells labeled with the indicated dye. Deconvolution using Huygens Essential X11 software was performed to smooth the noisy confocal data. Average brightness per cell was calculated by measuring the total fluorescence intensity of the first frame of the movies and dividing by the total number of cells. The GFP fluorescence intensity was used as an internal control to correct for variation in expression between cells; $n = 25$ for **30**; $n = 35$ for **30_D**; $n = 37$ for **31_D**. Photobleaching curves were generated via the EMBL bleach correction plug-in on ImageJ. The graph consists of the average photobleaching traces of movies ($n = 5$) representing 25–40 cells per condition.

Statistics and Reproducibility. For spectroscopy measurements, photobleaching experiments, or photo- and chromo-stability evaluations, reported *n* values represent measurements of different samples prepared from the same dye DMSO stock solution or HaloTag conjugate stock solution. For cell loading experiments, *n* represents the number of intensity values from individual nuclei extracted from three random fields of view at the indicated time points. For initial comparison of HaloTag ligands **20** and **21** in SPT, *n* indicates separate cells. For the comparison of the SPT intensity of HaloTag ligands **20**, **20_D**, and **21_D**, or **30**, **30_D**, and **31_D**, *n* indicates separate events extracted by the MTT algorithm. For full comparison of the SPT duration time of HaloTag ligands **20**, **20_D**, and **21_D**, or **30**, **30_D**, and **31_D**, *n* indicates values extracted from separate cells. For cell-loading experiments, *n* represents the number of intensity values from individual cells extracted from three fields of view at the indicated time points. For fluorescence microscopy experiments, all procedures were repeated at least once on a separate biological sample to ensure results were similar. Statistical analyses were performed using GraphPad Prism. P values were indicated as follows: 0.1234 (ns), 0.0332 (*), 0.0021 (**), 0.0002 (***), <0.0001 (****).

General Experimental Information for Synthesis

Commercial reagents were obtained from reputable suppliers and used as received. All solvents were purchased in septum-sealed bottles stored under an inert atmosphere. Fluorescein ditriflate[1] (**15**), Oregon Green ditriflate[1] (**S11**), 6-*tert*-butoxycarbonylfluorescein ditriflate[3] (**S1**), carbofluorescein ditriflate[2] (**S13**), Si-fluorescein ditriflate[3] (**S14**), 4-methylumbelliferone triflate[13] (**S9**), 10-acetyl-10*H*-phenoxazine-3,7-diyl bis(trifluoromethanesulfonate)[3] (**S10**), and 6-*tert*-butoxycarbonyl-Si-fluorescein ditriflate[3] (**S15**) were synthesized as previously described. Azetidine-2,2,3,3,4,4-d_6 was used as either the free base (Cambridge Isotopes) or the hydrochloride salt (synthesized as previously described[14]). All reactions were sealed with septa through which a nitrogen atmosphere was introduced unless otherwise noted. Reactions were conducted in round-bottomed flasks or septum-capped crimp-top vials containing Teflon-coated magnetic stir bars. Heating of reactions was accomplished with a silicon oil bath or an aluminum reaction block on top of a stirring hotplate equipped with an electronic contact thermometer to maintain the indicated temperatures.

Reactions were monitored by thin layer chromatography (TLC) on precoated TLC glass plates (silica gel 60 F_{254}, 250 μm thickness) or by LC–MS (Phenomenex Kinetex 2.1 mm × 30 mm 2.6 μm C18 column; 5 μL injection; 5–98% MeCN/H_2O, linear gradient, with constant 0.1% v/v HCO_2H additive; 6 min run; 0.5 mL/min flow; ESI; positive ion mode). TLC chromatograms were visualized by UV illumination or developed with *p*-anisaldehyde, ceric ammonium molybdate, or $KMnO_4$ stain. Reaction products were purified by flash chromatography on an automated purification system using pre-packed silica gel columns or by preparative HPLC (Phenomenex Gemini–NX 30 × 150 mm 5 μm C18 column). Analytical HPLC analysis was performed with an Agilent Eclipse XDB 4.6 × 150 mm 5 μm C18 column under the indicated conditions. High-resolution mass spectrometry was performed by the High Resolution Mass Spectrometry Facility at the University of Iowa.

NMR spectra were recorded on a 400 MHz spectrometer. ^{1}H and ^{13}C chemical shifts were referenced to TMS or residual solvent peaks, and ^{19}F chemical shifts were referenced to $CFCl_3$. Data for ^{1}H NMR spectra are reported as follows: chemical shift (δ ppm), multiplicity (s = singlet, d = doublet, t = triplet, q = quartet, dd = doublet of doublets, m = multiplet), coupling constant (Hz), integration. Data for ^{13}C NMR spectra are reported by chemical shift (δ ppm) with hydrogen multiplicity (C, CH, CH_2, CH_3) information obtained from DEPT spectra.

EXPERIMENTALS

Pd-Catalyzed Cross-Coupling Reactions

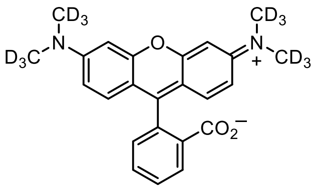

TMR-d_{12} (1_D). A vial was charged with fluorescein ditriflate (**15**, 150 mg, 0.251 mmol), dimethyl-d_6-amine hydrochloride (**16**, 52.9 mg, 0.604 mmol, 2.4 eq), Pd_2dba_3 (23.0 mg, 25.1 μmol, 0.1 eq), XPhos (36.0 mg, 75.4 μmol, 0.3 eq), and Cs_2CO_3 (393 mg, 1.21 mmol, 4.8 eq). The vial was sealed and evacuated/backfilled with nitrogen (3×). Dioxane (1.5 mL) was added, and the reaction was flushed again with nitrogen (3×). The reaction was then stirred at 100 °C for 4 h. It was subsequently cooled to room temperature, diluted with MeOH, deposited onto Celite, and concentrated to dryness. Purification by silica gel chromatography (0–10% MeOH (2 M NH_3)/CH_2Cl_2, linear gradient; dry load on Celite) followed by reverse phase HPLC (10–50% MeCN/H_2O, linear gradient, with constant 0.1% v/v TFA additive) afforded $\mathbf{1_D}$ (92 mg, 71%, TFA salt) as a dark red solid. ^1H NMR (CD_3OD, 400 MHz) δ 8.37 – 8.32 (m, 1H), 7.86 (td, J = 7.5, 1.5 Hz, 1H), 7.80 (td, J = 7.6, 1.5 Hz, 1H), 7.44 – 7.38 (m, 1H), 7.15 (d, J = 9.5 Hz, 2H), 7.05 (dd, J = 9.5, 2.5 Hz, 2H), 6.97 (d, J = 2.5 Hz, 2H); ^{13}C NMR (CD_3OD, 101 MHz) δ 168.0 (C), 161.8 (C), 159.1 (C), 159.0 (C), 135.3 (C), 133.8 (CH), 132.5 (CH), 132.3 (C), 132.1 (CH), 131.5 (CH), 131.4 (CH), 115.4 (CH), 115.0 (C), 97.3 (CH); Analytical HPLC: t_R = 10.5 min, >99% purity (10–95% MeCN/H_2O, linear gradient, with constant 0.1% v/v TFA additive; 20 min run; 1 mL/min flow; ESI; positive ion mode; detection at 550 nm); HRMS (ESI) calcd for $C_{24}H_{11}D_{12}N_2O_3$ $[M+H]^+$ 399.2456, found 399.2454.

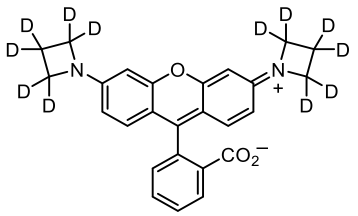

JFX$_{549}$ ($\mathbf{6_D}$). A vial was charged with fluorescein ditriflate (**15**, 200 mg, 0.335 mmol), azetidine-2,2,3,3,4,4-d_6 hydrochloride[14] (167 mg, 1.68 mmol, 5 eq), Pd_2dba_3 (30.7 mg, 33.5 μmol, 0.1 eq), XPhos (48.0 mg, 0.101 mmol, 0.3 eq), and Cs_2CO_3 (874 mg, 2.68 mmol, 8 eq). The vial was sealed and evacuated/backfilled with nitrogen (3×). Dioxane (2 mL) was added, and the reaction was flushed again with nitrogen (3×). The reaction was then stirred at 100 °C for 2 h. It was subsequently cooled to room temperature, diluted with MeOH, deposited onto Celite, and concentrated to dryness. Purification by silica gel chromatography (0–10% MeOH (2 M NH_3)/CH_2Cl_2, linear gradient; dry load on Celite) afforded $\mathbf{6_D}$ (115 mg, 81%) as a dark red solid. ^1H NMR ($CDCl_3$, 400 MHz) δ 8.02 – 7.95 (m, 1H), 7.63 (td, J = 7.4, 1.3 Hz, 1H), 7.57 (td, J = 7.4, 1.1 Hz, 1H), 7.17 (dt, J = 7.5, 1.0 Hz, 1H), 6.56 (d, J = 8.6 Hz, 2H), 6.20 (d, J = 2.3 Hz, 2H), 6.08 (dd, J = 8.6, 2.3 Hz, 2H); ^{13}C NMR ($CDCl_3$, 101 MHz) δ 169.9 (C), 153.8 (C), 153.1 (C), 153.0 (C), 134.6 (CH), 129.4 (CH), 129.0 (CH), 127.8 (C), 125.0 (CH), 124.3 (CH), 107.9 (C), 107.8 (CH), 97.7 (CH); Analytical

HPLC: t_R = 11.3 min, >99% purity (10–95% MeCN/H_2O, linear gradient, with constant 0.1% v/v TFA additive; 20 min run; 1 mL/min flow; ESI; positive ion mode; detection at 550 nm); HRMS (ESI) calcd for $C_{26}H_{11}D_{12}N_2O_3$ $[M+H]^+$ 423.2456, found 423.2454.

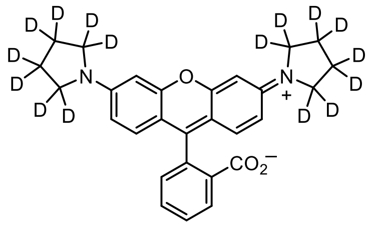

JFX$_{554}$ (5$_D$). A vial was charged with fluorescein ditriflate (**15**, 200 mg, 0.335 mmol), Pd_2dba_3 (30.7 mg, 33.5 µmol, 0.1 eq), XPhos (48.0 mg, 0.101 mmol, 0.3 eq), and Cs_2CO_3 (306 mg, 0.939 mmol, 2.8 eq). The vial was sealed and evacuated/backfilled with nitrogen (3×). Dioxane (2 mL) was added, and the reaction was flushed again with nitrogen (3×). Following the addition of pyrrolidine-2,2,3,3,4,4,5,5-d_8 (64 mg, 0.805 mmol, 2.4 eq), the reaction was stirred at 100 °C for 3 h. It was subsequently cooled to room temperature, diluted with MeOH, deposited onto Celite, and concentrated to dryness. Purification by silica gel chromatography (0–10% MeOH (2 M NH_3)/CH_2Cl_2, linear gradient; dry load on Celite) afforded **5$_D$** (116 mg, 76%) as a dark red-purple solid. ^1H NMR (CD_3OD, 400 MHz) δ 8.11 – 8.07 (m, 1H), 7.65 (td, J = 7.5, 1.6 Hz, 1H), 7.60 (td, J = 7.4, 1.6 Hz, 1H), 7.26 (d, J = 9.3 Hz, 2H), 7.25 – 7.22 (m, 1H), 6.85 (dd, J = 9.3, 2.4 Hz, 2H), 6.75 (d, J = 2.3 Hz, 2H); ^{13}C NMR (CD_3OD, 101 MHz) δ 163.4 (C), 158.9 (C), 156.1 (C), 141.8 (C), 133.9 (C), 132.9 (CH), 131.0 (CH), 130.7 (CH), 130.5 (CH), 130.4 (CH), 115.6 (CH), 115.1 (C), 97.5 (CH); Analytical HPLC: t_R = 12.4 min, >99% purity (10–95% MeCN/H_2O, linear gradient, with constant 0.1% v/v TFA additive; 20 min run; 1 mL/min flow; ESI; positive ion mode; detection at 550 nm); HRMS (ESI) calcd for $C_{28}H_{11}D_{16}N_2O_3$ $[M+H]^+$ 455.3020, found 455.3018.

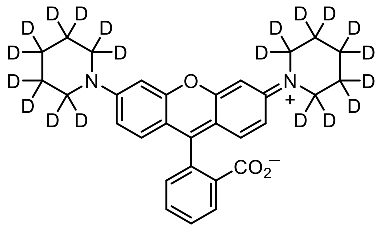

2-(3,6-Bis(piperidin-1-yl-*d*$_{10}$)xanthylium-9-yl)benzoate (17$_D$). The title compound (96%, dark red-purple solid) was prepared from fluorescein ditriflate (**15**) and piperidine-2,2,3,3,4,4,5,5,6,6-d_{10} according to the procedure described for **5$_D$**. ^1H NMR (CD_3OD, 400 MHz) δ 8.11 – 8.05 (m, 1H), 7.66 (td, J = 7.4, 1.8 Hz, 1H), 7.62 (td, J = 7.3, 1.7 Hz, 1H), 7.26 – 7.21 (m, 1H), 7.17 (d, J = 9.4 Hz, 2H), 7.06 (dd, J = 9.4, 2.6 Hz, 2H), 7.01 (d, J = 2.5 Hz, 2H); ^{13}C NMR (CD_3OD, 101 MHz) δ 158.9 (C), 157.6 (C), 152.1 (C), 139.7 (C), 136.7 (C), 132.6 (CH), 131.4 (CH), 130.8 (CH), 130.3 (CH), 129.7 (CH), 115.1 (CH), 114.5 (C), 98.6 (CH); Analytical HPLC: t_R = 12.7 min, >99% purity (10–95% MeCN/H_2O, linear gradient, with constant 0.1% v/v TFA additive; 20 min run; 1 mL/min flow; ESI; positive ion mode; detection at 550 nm); HRMS (ESI) calcd for $C_{30}H_{11}D_{20}N_2O_3$ $[M+H]^+$ 487.3585, found 487.3588.

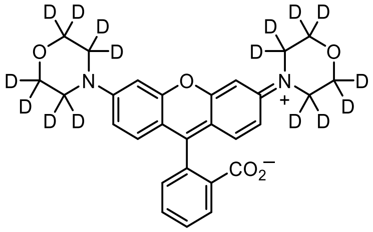

2-(3,6-Bis(morpholino-d_8)xanthylium-9-yl)benzoate (18_D). The title compound (88%, pink solid) was prepared from fluorescein ditriflate (**15**) and morpholine-2,2,3,3,5,5,6,6-d_8 according to the procedure described for $\mathbf{5_D}$. ^{1}H NMR ($CDCl_3$, 400 MHz) δ 8.04 – 7.98 (m, 1H), 7.64 (td, J = 7.4, 1.4 Hz, 1H), 7.59 (td, J = 7.4, 1.2 Hz, 1H), 7.18 – 7.13 (m, 1H), 6.67 (d, J = 2.5 Hz, 2H), 6.65 (d, J = 8.8 Hz, 2H), 6.57 (dd, J = 8.8, 2.5 Hz, 2H); ^{13}C NMR ($CDCl_3$, 101 MHz) δ 169.8 (C), 153.4 (C), 153.1 (C), 152.8 (C), 134.9 (CH), 129.6 (CH), 128.9 (CH), 127.2 (C), 125.0 (CH), 124.1 (CH), 111.4 (CH), 109.9 (C), 101.8 (CH), 83.9 (C); Analytical HPLC: t_R = 10.2 min, >99% purity (10–95% MeCN/H_2O, linear gradient, with constant 0.1% v/v TFA additive; 20 min run; 1 mL/min flow; ESI; positive ion mode; detection at 550 nm); HRMS (ESI) calcd for $C_{28}H_{11}D_{16}N_2O_5$ [M+H]$^+$ 487.2919, found 487.2926.

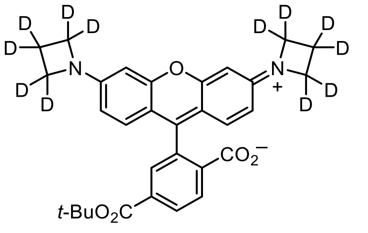

6-*tert*-Butoxycarbonyl-JFX$_{549}$ (S2). The title compound (72%, dark red-purple solid) was prepared from 6-*tert*-butoxycarbonylfluorescein ditriflate (**S1**) and azetidine-2,2,3,3,4,4-d_6 hydrochloride[14] according to the procedure described for $\mathbf{6_D}$. ^{1}H NMR ($CDCl_3$, 400 MHz) δ 8.19 (dd, J = 8.0, 1.3 Hz, 1H), 8.01 (dd, J = 8.0, 0.5 Hz, 1H), 7.77 – 7.70 (m, 1H), 6.53 (d, J = 8.6 Hz, 2H), 6.21 (d, J = 2.3 Hz, 2H), 6.09 (dd, J = 8.6, 2.3 Hz, 2H), 1.54 (s, 9H); ^{13}C NMR ($CDCl_3$, 101 MHz) δ 169.1 (C), 164.5 (C), 153.8 (C), 153.3 (C), 152.9 (C), 138.1 (C), 130.8 (C), 130.6 (CH), 128.9 (CH), 125.3 (CH), 124.8 (CH), 107.8 (CH), 107.2 (C), 97.7 (CH), 86.5 (C), 82.5 (C), 28.2 (CH_3); Analytical HPLC: t_R = 12.9 min, >99% purity (10–95% MeCN/H_2O, linear gradient, with constant 0.1% v/v TFA additive; 20 min run; 1 mL/min flow; ESI; positive ion mode; detection at 550 nm); HRMS (ESI) calcd for $C_{31}H_{19}D_{12}N_2O_5$ [M+H]$^+$ 523.2981, found 523.2982.

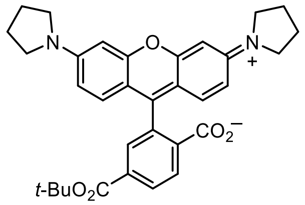

6-*tert*-Butoxycarbonyl-Rh$_P$ (S3). The title compound (59%, dark red-purple solid) was prepared from 6-*tert*-butoxycarbonylfluorescein ditriflate (**S1**) and pyrrolidine according to the procedure described for $\mathbf{5_D}$. ^{1}H NMR (CD_3OD, 400 MHz) δ 8.20 (dd, J = 8.1, 1.7 Hz, 1H), 8.11 (d, J = 8.1 Hz, 1H), 7.78 (d, J = 1.7 Hz, 1H), 7.22 (d, J =

9.4 Hz, 2H), 6.87 (dd, J = 9.4, 2.3 Hz, 2H), 6.77 (d, J = 2.3 Hz, 2H), 3.65 – 3.52 (m, 8H), 2.20 – 2.06 (m, 8H), 1.59 (s, 9H); ^{13}C NMR (CD_3OD, 101 MHz) δ 172.4 (C), 166.1 (C), 162.2 (C), 158.9 (C), 156.1 (C), 145.9 (C), 133.8 (C), 133.6 (C), 132.7 (CH), 131.5 (CH), 131.2 (CH), 131.0 (CH), 115.9 (CH), 115.1 (C), 97.6 (CH), 83.1 (C), 49.9 (CH_2), 28.4 (CH_3), 26.2 (CH_2); Analytical HPLC: t_R = 13.7 min, >99% purity (10–95% MeCN/H_2O, linear gradient, with constant 0.1% v/v TFA additive; 20 min run; 1 mL/min flow; ESI; positive ion mode; detection at 550 nm); HRMS (ESI) calcd for $C_{33}H_{35}N_2O_5$ $[M+H]^+$ 539.2540, found 539.2544.

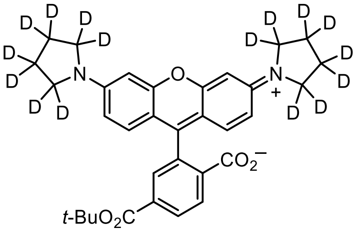

6-*tert*-Butoxycarbonyl-JFX$_{554}$ (S4). The title compound (58%, dark red-purple solid) was prepared from 6-*tert*-butoxycarbonylfluorescein ditriflate (**S1**) and pyrrolidine-2,2,3,3,4,4,5,5-d_8 according to the procedure described for **5$_D$**. ^{1}H NMR (CD_3OD, 400 MHz) δ 8.20 (dd, J = 8.2, 1.7 Hz, 1H), 8.11 (d, J = 8.1 Hz, 1H), 7.78 (d, J = 1.6 Hz, 1H), 7.21 (d, J = 9.3 Hz, 2H), 6.87 (dd, J = 9.3, 2.4 Hz, 2H), 6.76 (d, J = 2.3 Hz, 2H), 1.59 (s, 9H); ^{13}C NMR (CD_3OD, 101 MHz) δ 172.5 (C), 166.1 (C), 162.2 (C), 158.9 (C), 156.2 (C), 145.9 (C), 133.7 (C), 133.6 (C), 132.7 (CH), 131.5 (CH), 131.2 (CH), 131.0 (CH), 115.9 (CH), 115.0 (C), 97.6 (CH), 83.1 (C), 28.4 (CH_3); Analytical HPLC: t_R = 13.6 min, >99% purity (10–95% MeCN/H_2O, linear gradient, with constant 0.1% v/v TFA additive; 20 min run; 1 mL/min flow; ESI; positive ion mode; detection at 550 nm); HRMS (ESI) calcd for $C_{33}H_{19}D_{16}N_2O_5$ $[M+H]^+$ 555.3545, found 555.3544.

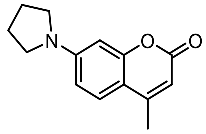

4-Methyl-7-(pyrrolidin-1-yl)-2*H*-chromen-2-one (22). A vial was charged with 4-methylumbelliferone triflate (**S9**; 250 mg, 0.811 mmol), RuPhos-G3-palladacycle (33.9 mg, 40.6 μmol, 0.05 eq), RuPhos (18.9 mg, 40.6 μmol, 0.05 eq), and K_2CO_3 (157 mg, 1.14 mmol, 1.4 eq). The vial was sealed and evacuated/backfilled with nitrogen (3×). Dioxane (4 mL) was added, and the reaction was flushed again with nitrogen (3×). Following the addition of pyrrolidine (81.2 μL, 0.973 mmol, 1.2 eq), the reaction was stirred at 80 °C for 6 h. It was subsequently cooled to room temperature, filtered through Celite with CH_2Cl_2, and concentrated *in vacuo*. Purification by silica gel chromatography (0–50% EtOAc/hexanes, linear gradient) afforded 171 mg (92%) of **22** as a yellow solid. ^{1}H NMR ($CDCl_3$, 400 MHz) δ 7.39 (d, J = 8.8 Hz, 1H), 6.48 (dd, J = 8.8, 2.4 Hz, 1H), 6.38 (d, J = 2.4 Hz, 1H), 5.94 (q, J = 1.1 Hz, 1H), 3.40 – 3.30 (m, 4H), 2.34 (d, J = 1.1 Hz, 3H), 2.10 – 1.99 (m, 4H); ^{13}C NMR ($CDCl_3$, 101 MHz) δ 162.3 (C), 155.9 (C), 153.2 (C), 150.5 (C), 125.5 (CH), 109.4 (C), 109.1 (CH), 108.8 (CH), 98.0 (CH), 47.8 (CH_2), 25.6 (CH_2), 18.6 (CH_3); Analytical HPLC: t_R = 13.2 min, >99% purity (10–95% MeCN/H_2O, linear gradient, with constant

0.1% v/v TFA additive; 20 min run; 1 mL/min flow; ESI; positive ion mode; detection at 375 nm); HRMS (ESI) calcd for $C_{14}H_{16}NO_2$ $[M+H]^+$ 230.1176, found 230.1180.

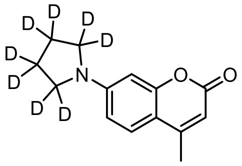

4-Methyl-7-(pyrrolidin-1-yl-*d*$_8$)-2*H*-chromen-2-one (22$_D$). The title compound (86%, yellow solid) was prepared from 4-methylumbelliferone triflate (**S9**) and pyrrolidine-2,2,3,3,4,4,5,5-*d*$_8$ according to the procedure described for **22**. ^{1}H NMR ($CDCl_3$, 400 MHz) δ 7.38 (d, *J* = 8.8 Hz, 1H), 6.48 (dd, *J* = 8.8, 2.4 Hz, 1H), 6.38 (d, *J* = 2.4 Hz, 1H), 5.94 (q, *J* = 1.1 Hz, 1H), 2.34 (d, *J* = 1.1 Hz, 3H); ^{13}C NMR ($CDCl_3$, 101 MHz) δ 162.3 (C), 155.9 (C), 153.2 (C), 150.6 (C), 125.5 (CH), 109.3 (C), 109.1 (CH), 108.7 (CH), 98.0 (CH), 18.6 (CH_3); Analytical HPLC: t_R = 13.1 min, >99% purity (10–95% $MeCN/H_2O$, linear gradient, with constant 0.1% v/v TFA additive; 20 min run; 1 mL/min flow; ESI; positive ion mode; detection at 375 nm); HRMS (ESI) calcd for $C_{14}H_8D_8NO_2$ $[M+H]^+$ 238.1678, found 238.1682.

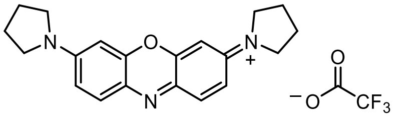

3,7-Di(pyrrolidin-1-yl)phenoxazin-5-ium trifluoroacetate (23). A vial was charged with 10-acetyl-10*H*-phenoxazine-3,7-diyl bis(trifluoromethanesulfonate) (**S10**; 250 mg, 0.480 mmol), Pd_2dba_3 (43.9 mg, 48.0 μmol, 0.1 eq), XPhos (68.6 mg, 0.144 mmol, 0.3 eq), and Cs_2CO_3 (437 mg, 1.34 mmol, 2.8 eq). The vial was sealed and evacuated/backfilled with nitrogen (3×). Dioxane (2.5 mL) was added, and the reaction was flushed again with nitrogen (3×). Following the addition of pyrrolidine (96.1 μL, 1.15 mmol, 2.4 eq), the reaction was stirred at 80 °C for 4 h. It was then cooled to room temperature, filtered through Celite with CH_2Cl_2, and concentrated *in vacuo*. Purification by silica gel chromatography (0–40% EtOAc/toluene, linear gradient) afforded the *N*-acetyl leuco-dye (112 mg, 64%) as an off-white solid.

The intermediate leuco-dye (112 mg, 0.308 mmol) was taken up in a mixture of CH_2Cl_2 (9 mL) and water (1 mL). DDQ (105 mg, 0.462 mmol, 1.5 eq) was added, and the reaction was stirred at room temperature for 2 h. The crude reaction mixture was then deposited onto Celite and concentrated to dryness. Silica gel chromatography (0–20% $MeOH/CH_2Cl_2$, linear gradient, with constant 1% v/v AcOH additive; dry load with Celite) followed by reverse phase HPLC (10–50% $MeCN/H_2O$, linear gradient, with constant 0.1% v/v TFA additive) afforded 127 mg (95%) of **23** as a dark blue solid. ^{1}H NMR (CD_3OD, 400 MHz) δ 7.77 (d, *J* = 9.5 Hz, 2H), 7.25 (dd, *J* = 9.4, 2.5 Hz, 2H), 6.81 (d, *J* = 2.5 Hz, 2H), 3.82 – 3.66 (m, 8H), 2.22 – 2.13 (m, 8H); ^{13}C NMR (CD_3OD, 101 MHz) δ 156.6 (C), 150.4 (C), 135.5 (C), 135.3 (CH), 119.4 (CH), 98.0 (CH), 50.9 (bs, CH_2), 26.2 (bs, CH_2); Analytical HPLC: t_R = 10.4 min, >99% purity (10–95% $MeCN/H_2O$, linear gradient, with constant 0.1% v/v TFA additive; 20 min run; 1 mL/min flow; ESI; positive ion mode; detection at 650 nm); HRMS (ESI) calcd for $C_{20}H_{22}N_3O$ $[M]^+$ 320.1757, found 320.1763.

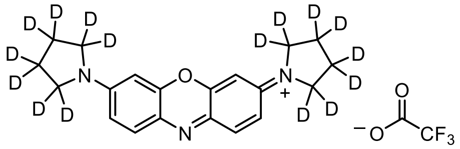

3,7-Bis(pyrrolidin-1-yl-d_8)phenoxazin-5-ium trifluoroacetate (23_D). The title compound (34% 2-step yield, dark blue solid) was prepared from 10-acetyl-10*H*-phenoxazine-3,7-diyl bis(trifluoromethanesulfonate) (**S10**) and pyrrolidine-2,2,3,3,4,4,5,5-d_8 according to the procedure described for **23**. ^{1}H NMR (CD_3OD, 400 MHz) δ 7.77 (d, J = 9.4 Hz, 2H), 7.25 (dd, J = 9.5, 2.5 Hz, 2H), 6.80 (d, J = 2.5 Hz, 2H); ^{13}C NMR (CD_3OD, 101 MHz) δ 156.7 (C), 150.4 (C), 135.5 (C), 135.3 (CH), 119.4 (CH), 97.9 (CH); Analytical HPLC: t_R = 10.3 min, >99% purity (10–95% MeCN/H_2O, linear gradient, with constant 0.1% v/v TFA additive; 20 min run; 1 mL/min flow; ESI; positive ion mode; detection at 650 nm); HRMS (ESI) calcd for $C_{20}H_6D_{16}N_3O$ $[M]^+$ 336.2762, found 336.2765.

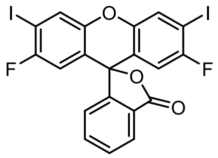

2',7'-Difluoro-3',6'-diiodofluoran (S12). A vial was charged with Oregon Green ditriflate **S11** (5.00 g, 7.91 mmol), tetrabutylammonium iodide (17.52 g, 47.44 mmol, 6 eq), and [RuCp*$(MeCN)_3$]OTf (201 mg, 0.395 mmol, 0.05 eq). The vial was sealed and evacuated/backfilled with nitrogen (3×). DMI (40 mL) was added, and the reaction was flushed again with nitrogen (3×). The reaction was stirred at 100 °C for 18 h. It was then cooled to room temperature, diluted with water, and extracted with 3:1 $CHCl_3$/hexanes (3×). The combined organics were washed with water and brine, dried over anhydrous $MgSO_4$, filtered, and concentrated *in vacuo*. The crude material was purified twice by silica gel chromatography (0–10% EtOAc/hexanes, linear gradient, with constant 40% v/v CH_2Cl_2 additive; then, 100% CH_2Cl_2) to yield 4.00 g of **S12** (86%) as a white solid. ^{1}H NMR (DMSO-d_6, 400 MHz) δ 8.06 – 8.01 (m, 1H), 7.94 (d, $^4J_{HF}$ = 5.2 Hz, 2H), 7.80 (td, J = 7.4, 1.4 Hz, 1H), 7.75 (td, J = 7.4, 1.2 Hz, 1H), 7.38 – 7.33 (m, 1H), 6.77 (d, $^3J_{HF}$ = 8.0 Hz, 2H); ^{19}F NMR (DMSO-d_6, 376 MHz) δ -100.10 (dd, J_{FH} = 8.0, 5.2 Hz); ^{13}C NMR (DMSO-d_6, 101 MHz) δ 168.0 (C), 157.5 (d, $^1J_{CF}$ = 239.5 Hz, CF), 151.4 (C), 146.71 (d, $^4J_{CF}$ = 2.4 Hz, C), 136.0 (CH), 130.7 (CH), 127.4 (d, $^3J_{CF}$ = 2.4 Hz, CH), 125.5 (CH), 125.0 (C), 123.7 (CH), 119.44 (d, $^3J_{CF}$ = 6.9 Hz, C), 113.2 (d, $^2J_{CF}$ = 27.5 Hz, CH), 86.0 (d, $^2J_{CF}$ = 29.0 Hz, C), 80.2 (C); HRMS (ESI) calcd for $C_{20}H_9F_2I_2O_3$ $[M+H]^+$ 588.8604, found 588.8613.

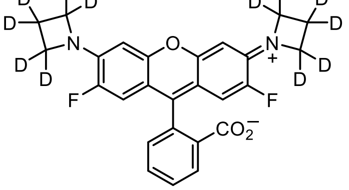

JFX_{552} (24_D). The title compound (66%, dark red-purple solid, TFA salt) was prepared from 2',7'-difluoro-3',6'-diiodofluoran (**S12**) and azetidine-2,2,3,3,4,4-d_6 according to the procedure described for **5_D**. ^{1}H NMR (CD_3OD, 400 MHz) δ 8.37 – 8.33 (m, 1H), 7.86 (td, J = 7.5, 1.6 Hz, 1H), 7.81 (td, J = 7.6, 1.6 Hz, 1H), 7.40 – 7.36 (m, 1H), 6.73

(d, ${}^{3}J_{HF}$ = 12.7 Hz, 2H), 6.65 (d, ${}^{4}J_{HF}$ = 7.3 Hz, 2H); ^{19}F NMR (CD_3OD, 376 MHz) δ -75.25 (s, 3F), -130.91 (dd, J_{FH} = 12.8, 7.4 Hz, 2F); ^{13}C NMR (CD_3OD, 101 MHz) δ 168.0 (C), 160.6 (C), 155.9 (C), 151.7 (d, ${}^{1}J_{CF}$ = 250.5 Hz, CF), 148.9 (d, ${}^{2}J_{CF}$ = 15.2 Hz, C), 134.9 (C), 134.2 (CH), 132.7 (CH), 131.93 (C), 131.86 (CH), 131.2 (CH), 114.59 (d, ${}^{3}J_{CF}$ = 8.5 Hz, C), 113.7 (d, ${}^{2}J_{CF}$ = 21.6 Hz, CH), 97.8 (d, ${}^{3}J_{CF}$ = 5.7 Hz, CH); Analytical HPLC: t_R = 11.4 min, >99% purity (10–95% MeCN/H_2O, linear gradient, with constant 0.1% v/v TFA additive; 20 min run; 1 mL/min flow; ESI; positive ion mode; detection at 550 nm); HRMS (ESI) calcd for $C_{26}H_9D_{12}F_2N_2O_3$ $[M+H]^+$ 459.2268, found 459.2272.

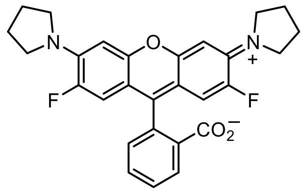

2',7'-Difluoro-Rh$_P$ (25). The title compound (38%, purple solid) was prepared from 2',7'-difluoro-3',6'-diiodofluoran (**S12**) and pyrrolidine according to the procedure described for **5$_D$**. ^{1}H NMR ($CDCl_3$, 400 MHz) δ 8.01 (dt, J = 7.4, 1.1 Hz, 1H), 7.67 (td, J = 7.4, 1.3 Hz, 1H), 7.61 (td, J = 7.4, 1.1 Hz, 1H), 7.17 (dt, J = 7.5, 1.1 Hz, 1H), 6.40 (d, ${}^{4}J_{HF}$ = 7.7 Hz, 2H), 6.28 (d, ${}^{3}J_{HF}$ = 14.0 Hz, 2H), 3.51 – 3.35 (m, 8H), 2.01 – 1.88 (m, 8H); ^{19}F NMR ($CDCl_3$, 376 MHz) δ -133.06 – -133.17 (m); ^{13}C NMR ($CDCl_3$, 101 MHz) δ 169.5 (C), 152.7 (C), 148.8 (d, ${}^{4}J_{CF}$ = 1.0 Hz, C), 148.2 (d, ${}^{1}J_{CF}$ = 238.5 Hz, CF), 139.5 (d, ${}^{2}J_{CF}$ = 11.3 Hz, C), 135.0 (CH), 129.8 (CH), 127.3 (C), 125.2 (CH), 124.1 (CH), 114.0 (d, ${}^{2}J_{CF}$ = 24.0 Hz, CH), 105.2 (d, ${}^{3}J_{CF}$ = 6.7 Hz, C), 101.6 (d, ${}^{3}J_{CF}$ = 5.5 Hz, CH), 84.8 (C), 49.8 (d, ${}^{4}J_{CF}$ = 5.4 Hz, CH_2), 25.5 (d, ${}^{5}J_{CF}$ = 2.1 Hz, CH_2); Analytical HPLC: t_R = 12.3 min, >99% purity (10–95% MeCN/H_2O, linear gradient, with constant 0.1% v/v TFA additive; 20 min run; 1 mL/min flow; ESI; positive ion mode; detection at 550 nm); HRMS (ESI) calcd for $C_{28}H_{25}F_2N_2O_3$ $[M+H]^+$ 475.1828, found 475.1840.

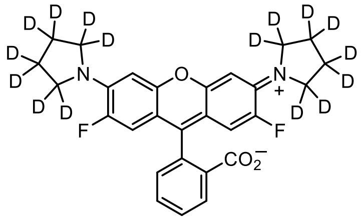

JFX$_{560}$ (25$_D$). The title compound (62%, purple solid) was prepared from 2',7'-difluoro-3',6'-diiodofluoran (**S12**) and pyrrolidine-2,2,3,3,4,4,5,5-d_8 according to the procedure described for **5$_D$**. ^{1}H NMR ($CDCl_3$, 400 MHz) δ 8.04 – 7.97 (m, 1H), 7.67 (td, J = 7.4, 1.3 Hz, 1H), 7.61 (td, J = 7.4, 1.1 Hz, 1H), 7.17 (dt, J = 7.5, 0.9 Hz, 1H), 6.40 (d, ${}^{4}J_{HF}$ = 7.8 Hz, 2H), 6.28 (d, ${}^{3}J_{HF}$ = 14.0 Hz, 2H); ^{19}F NMR ($CDCl_3$, 376 MHz) δ -133.24 (dd, J_{FH} = 14.1, 7.7 Hz); ^{13}C NMR ($CDCl_3$, 101 MHz) δ 169.5 (C), 152.6 (C), 148.9 (d, ${}^{4}J_{CF}$ = 0.8 Hz, C), 148.2 (d, ${}^{1}J_{CF}$ = 238.5 Hz, CF), 139.6 (d, ${}^{2}J_{CF}$ = 11.4 Hz, C), 135.0 (CH), 129.8 (CH), 127.4 (C), 125.2 (CH), 124.1 (CH), 114.0 (d, ${}^{2}J_{CF}$ = 23.9 Hz, CH), 105.1 (d, ${}^{3}J_{CF}$ = 6.7 Hz, C), 101.5 (d, ${}^{3}J_{CF}$ = 5.4 Hz, CH), 85.0 (C); Analytical HPLC: t_R = 12.3 min, >99% purity (10–95% MeCN/H_2O, linear gradient, with constant 0.1% v/v TFA additive; 20 min run; 1 mL/min flow; ESI; positive ion mode; detection at 550 nm); HRMS (ESI) calcd for $C_{28}H_9D_{16}F_2N_2O_3$ $[M+H]^+$ 491.2832, found 491.2839.

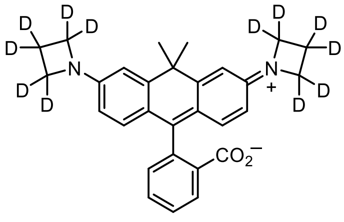

JFX$_{608}$ (26$_D$). The title compound (92%, pale blue solid) was prepared from carbofluorescein ditriflate (**S13**) and azetidine-2,2,3,3,4,4-d_6 according to the procedure described for **5$_D$**. ^{1}H NMR ($CDCl_3$, 400 MHz) δ 8.00 – 7.95 (m, 1H), 7.58 (td, J = 7.4, 1.4 Hz, 1H), 7.53 (td, J = 7.4, 1.2 Hz, 1H), 7.08 – 7.04 (m, 1H), 6.58 (d, J = 2.4 Hz, 2H), 6.55 (d, J = 8.6 Hz, 2H), 6.20 (dd, J = 8.6, 2.4 Hz, 2H), 1.82 (s, 3H), 1.72 (s, 3H); ^{13}C NMR ($CDCl_3$, 101 MHz) δ 170.9 (C), 155.5 (C), 152.5 (C), 146.9 (C), 134.5 (CH), 129.0 (CH), 128.9 (CH), 127.4 (C), 125.0 (CH), 124.1 (CH), 120.6 (C), 110.4 (CH), 107.9 (CH), 88.5 (C), 38.5 (C), 35.7 (CH_3), 32.3 (CH_3); Analytical HPLC: t_R = 11.9 min, >99% purity (10–95% MeCN/H_2O, linear gradient, with constant 0.1% v/v TFA additive; 20 min run; 1 mL/min flow; ESI; positive ion mode; detection at 600 nm); HRMS (ESI) calcd for $C_{29}H_{17}D_{12}N_2O_2$ [M+H]$^+$ 449.2977, found 449.2980.

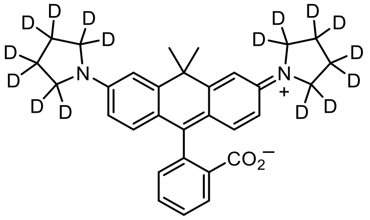

JFX$_{612}$ (27$_D$). The title compound (91%, pale blue solid) was prepared from carbofluorescein ditriflate (**S13**) and pyrrolidine-2,2,3,3,4,4,5,5-d_8 according to the procedure described for **5$_D$**. ^{1}H NMR ($CDCl_3$, 400 MHz) δ 8.00 – 7.96 (m, 1H), 7.57 (td, J = 7.3, 1.5 Hz, 1H), 7.52 (td, J = 7.4, 1.3 Hz, 1H), 7.08 – 7.04 (m, 1H), 6.72 (d, J = 2.5 Hz, 2H), 6.58 (d, J = 8.7 Hz, 2H), 6.35 (dd, J = 8.7, 2.5 Hz, 2H), 1.88 (s, 3H), 1.77 (s, 3H); ^{13}C NMR ($CDCl_3$, 101 MHz) δ 171.1 (C), 155.7 (C), 148.3 (C), 147.2 (C), 134.4 (CH), 129.2 (CH), 128.7 (CH), 127.6 (C), 124.9 (CH), 124.1 (CH), 118.8 (C), 111.0 (CH), 108.3 (CH), 38.6 (C), 36.0 (CH_3), 32.5 (CH_3); Analytical HPLC: t_R = 9.8 min, >99% purity (30–95% MeCN/H_2O, linear gradient, with constant 0.1% v/v TFA additive; 20 min run; 1 mL/min flow; ESI; positive ion mode; detection at 600 nm); HRMS (ESI) calcd for $C_{31}H_{17}D_{16}N_2O_2$ [M+H]$^+$ 481.3541, found 481.3545.

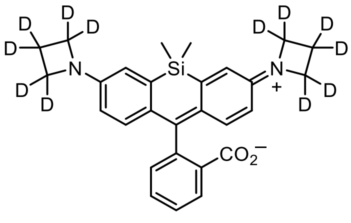

JFX$_{646}$ (28$_D$). The title compound (89%, light blue solid) was prepared from Si-fluorescein ditriflate (**S14**) and azetidine-2,2,3,3,4,4-d_6 according to the procedure described for **5$_D$**. ^{1}H NMR ($CDCl_3$, 400 MHz) δ 7.96 (dt, J = 7.6, 1.0 Hz, 1H), 7.64 (td, J = 7.5, 1.1 Hz, 1H), 7.54 (td, J = 7.5, 0.8 Hz, 1H), 7.31 (dt, J = 7.7, 0.9 Hz, 1H), 6.75 (d, J = 8.7 Hz, 2H), 6.67 (d, J = 2.6 Hz, 2H), 6.25 (dd, J = 8.6, 2.7 Hz, 2H), 0.61 (s, 3H), 0.59 (s, 3H); ^{13}C NMR ($CDCl_3$, 101

MHz) δ 170.8 (C), 154.3 (C), 151.1 (C), 137.1 (C), 133.7 (CH), 132.9 (C), 128.8 (CH), 128.0 (CH), 127.2 (C), 125.8 (CH), 124.8 (CH), 115.7 (CH), 112.3 (CH), 92.1 (C), 0.5 (CH_3), -1.5 (CH_3); Analytical HPLC: t_R = 12.5 min, >99% purity (10–95% MeCN/H_2O, linear gradient, with constant 0.1% v/v TFA additive; 20 min run; 1 mL/min flow; ESI; positive ion mode; detection at 650 nm); HRMS (ESI) calcd for $C_{28}H_{17}D_{12}N_2O_2Si$ $[M+H]^+$ 465.2746, found 465.2749.

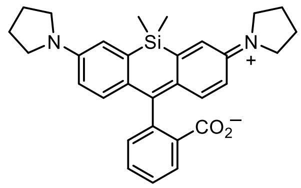

SiRh$_P$ (29). The title compound (96%, pale blue solid) was prepared from Si-fluorescein ditriflate (**S14**) and pyrrolidine according to the procedure described for **5$_D$**. ^{1}H NMR ($CDCl_3$, 400 MHz) δ 7.95 (dt, *J* = 7.6, 0.9 Hz, 1H), 7.62 (td, *J* = 7.5, 1.1 Hz, 1H), 7.52 (td, *J* = 7.5, 0.9 Hz, 1H), 7.31 – 7.27 (m, 1H), 6.79 (d, *J* = 2.7 Hz, 2H), 6.76 (d, *J* = 8.8 Hz, 2H), 6.38 (dd, *J* = 8.8, 2.8 Hz, 2H), 3.34 – 3.24 (m, 8H), 2.05 – 1.93 (m, 8H), 0.63 (s, 3H), 0.60 (s, 3H); ^{13}C NMR ($CDCl_3$, 101 MHz) δ 171.0 (C), 154.9 (C), 146.8 (C), 137.2 (C), 133.7 (CH), 131.0 (C), 128.6 (CH), 128.4 (CH), 127.2 (C), 125.6 (CH), 124.7 (CH), 115.9 (CH), 112.7 (CH), 92.5 (C), 47.6 (CH_2), 25.6 (CH_2), 0.6 (CH_3), -1.3 (CH_3); Analytical HPLC: t_R = 13.0 min, >99% purity (10–95% MeCN/H_2O, linear gradient, with constant 0.1% v/v TFA additive; 20 min run; 1 mL/min flow; ESI; positive ion mode; detection at 650 nm); HRMS (ESI) calcd for $C_{30}H_{33}N_2O_2Si$ $[M+H]^+$ 481.2306, found 481.2317.

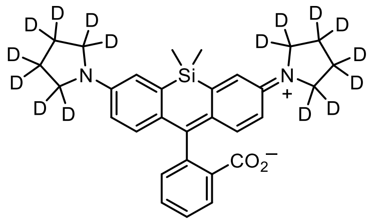

JFX$_{650}$ (**29$_D$**). The title compound (90%, off-white solid) was prepared from Si-fluorescein ditriflate (**S14**) and pyrrolidine-2,2,3,3,4,4,5,5-d_8 according to the procedure described for **5$_D$**. ^{1}H NMR ($CDCl_3$, 400 MHz) δ 7.95 (dt, *J* = 7.7, 1.0 Hz, 1H), 7.61 (td, *J* = 7.5, 1.1 Hz, 1H), 7.52 (td, *J* = 7.5, 0.9 Hz, 1H), 7.28 (dt, *J* = 7.8, 1.0 Hz, 1H), 6.79 (d, *J* = 2.8 Hz, 2H), 6.76 (d, *J* = 8.8 Hz, 2H), 6.38 (dd, *J* = 8.8, 2.8 Hz, 2H), 0.63 (s, 3H), 0.59 (s, 3H); ^{13}C NMR ($CDCl_3$, 101 MHz) δ 171.0 (C), 154.9 (C), 147.0 (C), 137.2 (C), 133.7 (CH), 131.0 (C), 128.6 (CH), 128.5 (CH), 127.2 (C), 125.7 (CH), 124.7 (CH), 115.9 (CH), 112.8 (CH), 92.5 (C), 0.6 (CH_3), -1.3 (CH_3); Analytical HPLC: t_R = 13.0 min, >99% purity (10–95% MeCN/H_2O, linear gradient, with constant 0.1% v/v TFA additive; 20 min run; 1 mL/min flow; ESI; positive ion mode; detection at 650 nm); HRMS (ESI) calcd for $C_{30}H_{17}D_{16}N_2O_2Si$ $[M+H]^+$ 497.3310, found 497.3312.

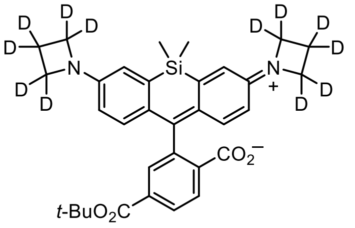

6-*tert*-Butoxycarbonyl-JFX$_{646}$ (S16). The title compound (93%, off-white solid) was prepared from 6-*tert*-butoxycarbonyl-Si-fluorescein ditriflate (**S15**) and azetidine-2,2,3,3,4,4-d_6 according to the procedure described for **5$_D$**. ^{1}H NMR ($CDCl_3$, 400 MHz) δ 8.11 (dd, J = 8.0, 1.3 Hz, 1H), 7.95 (dd, J = 8.0, 0.6 Hz, 1H), 7.83 – 7.80 (m, 1H), 6.82 (d, J = 8.7 Hz, 2H), 6.66 (d, J = 2.7 Hz, 2H), 6.29 (dd, J = 8.7, 2.7 Hz, 2H), 1.54 (s, 9H), 0.64 (s, 3H), 0.58 (s, 3H); ^{13}C NMR ($CDCl_3$, 101 MHz) δ 170.3 (C), 164.5 (C), 155.4 (C), 151.1 (C), 137.2 (C), 136.2 (C), 132.4 (C), 129.9 (CH), 129.2 (C), 127.7 (CH), 125.6 (CH), 125.2 (CH), 115.7 (CH), 112.6 (CH), 91.9 (C), 82.3 (C), 28.2 (CH_3), 0.2 (CH_3), -0.7 (CH_3); Analytical HPLC: t_R = 14.1 min, 98.8% purity (10–95% MeCN/H_2O, linear gradient, with constant 0.1% v/v TFA additive; 20 min run; 1 mL/min flow; ESI; positive ion mode; detection at 650 nm); HRMS (ESI) calcd for $C_{33}H_{25}D_{12}N_2O_4Si$ $[M+H]^+$ 565.3270, found 565.3277.

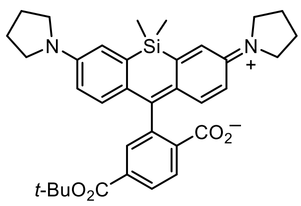

6-*tert*-Butoxycarbonyl-SiRh$_P$ (S17). The title compound (81%, off-white solid) was prepared from 6-*tert*-butoxycarbonyl-Si-fluorescein ditriflate (**S15**) and pyrrolidine according to the procedure described for **5$_D$**. ^{1}H NMR ($CDCl_3$, 400 MHz) δ 8.10 (dd, J = 8.0, 1.3 Hz, 1H), 7.96 (dd, J = 8.0, 0.5 Hz, 1H), 7.83 – 7.79 (m, 1H), 6.84 (d, J = 8.8 Hz, 2H), 6.79 (d, J = 2.7 Hz, 2H), 6.44 (dd, J = 8.8, 2.8 Hz, 2H), 3.35 – 3.25 (m, 8H), 2.04 – 1.95 (m, 8H), 1.53 (s, 9H), 0.67 (s, 3H), 0.60 (s, 3H); ^{13}C NMR ($CDCl_3$, 101 MHz) δ 170.6 (C), 164.5 (C), 155.9 (C), 146.8 (C), 137.2 (C), 136.3 (C), 130.5 (C), 129.8 (CH), 129.3 (C), 128.1 (CH), 125.5 (CH), 125.1 (CH), 115.9 (CH), 113.2 (CH), 92.2 (C), 82.2 (C), 47.6 (CH_2), 28.2 (CH_3), 25.6 (CH_2), 0.2 (CH_3), -0.5 (CH_3); Analytical HPLC: t_R = 14.5 min, >99% purity (10–95% MeCN/H_2O, linear gradient, with constant 0.1% v/v TFA additive; 20 min run; 1 mL/min flow; ESI; positive ion mode; detection at 650 nm); HRMS (ESI) calcd for $C_{35}H_{41}N_2O_4Si$ $[M+H]^+$ 581.2830, found 581.2839.

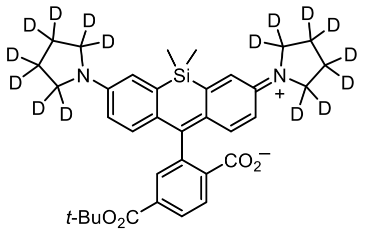

6-*tert*-Butoxycarbonyl-JFX$_{650}$ (S18). The title compound (87%, off-white solid) was prepared from 6-*tert*-butoxycarbonyl-Si-fluorescein ditriflate (**S15**) and pyrrolidine-2,2,3,3,4,4,5,5-d_8 according to the procedure described

for **5_D**. ^1H NMR ($CDCl_3$, 400 MHz) δ 8.10 (dd, *J* = 8.0, 1.3 Hz, 1H), 7.95 (dd, *J* = 8.0, 0.6 Hz, 1H), 7.83 – 7.78 (m, 1H), 6.84 (d, *J* = 8.8 Hz, 2H), 6.79 (d, *J* = 2.7 Hz, 2H), 6.43 (dd, *J* = 8.8, 2.8 Hz, 2H), 1.53 (s, 9H), 0.67 (s, 3H), 0.59 (s, 3H); ^{13}C NMR ($CDCl_3$, 101 MHz) δ 170.6 (C), 164.5 (C), 155.9 (C), 146.9 (C), 137.2 (C), 136.3 (C), 130.5 (C), 129.8 (CH), 129.3 (C), 128.1 (CH), 125.5 (CH), 125.1 (CH), 115.9 (CH), 113.1 (CH), 92.3 (C), 82.2 (C), 28.2 (CH_3), 0.2 (CH_3), -0.5 (CH_3); Analytical HPLC: t_R = 14.4 min, >99% purity (10–95% MeCN/H_2O, linear gradient, with constant 0.1% v/v TFA additive; 20 min run; 1 mL/min flow; ESI; positive ion mode; detection at 650 nm); HRMS (ESI) calcd for $C_{35}H_{25}D_{16}N_2O_4Si$ $[M+H]^+$ 597.3834, found 597.3835.

t-Butyl Ester Deprotection

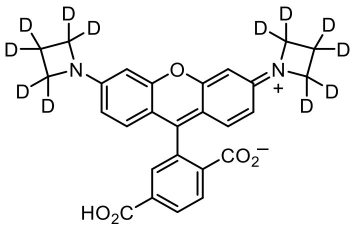

6-Carboxy-JFX$_{549}$ (S5). 6-*tert*-Butoxycarbonyl-JFX$_{549}$ (**S2**; 102 mg, 0.195 mmol) was taken up in CH_2Cl_2 (2.5 mL), and trifluoroacetic acid (0.5 mL) was added. The reaction was stirred at room temperature for 6 h. Toluene (3 mL) was added; the reaction mixture was concentrated to dryness and then azeotroped with MeOH three times to provide **S5** as a red-brown solid (109 mg, 96%, TFA salt). Analytical HPLC and NMR indicated that the material was >95% pure and did not require further purification prior to amide coupling. ^{1}H NMR (CD_3OD, 400 MHz) δ 8.40 (d, *J* = 8.1 Hz, 1H), 8.37 (dd, *J* = 8.2, 1.5 Hz, 1H), 7.95 – 7.93 (m, 1H), 7.06 (d, *J* = 9.2 Hz, 2H), 6.60 (dd, *J* = 9.2, 2.2 Hz, 2H), 6.54 (d, *J* = 2.2 Hz, 2H); ^{13}C NMR (CD_3OD, 101 MHz) δ 167.7 (C), 167.3 (C), 160.0 (C), 158.8 (C), 158.2 (C), 136.0 (C), 135.9 (C), 135.5 (C), 132.8 (CH), 132.3 (CH), 132.2 (CH), 114.8 (C), 113.6 (CH), 95.2 (CH); Analytical HPLC: t_R = 9.9 min, >99% purity (10–95% MeCN/H_2O, linear gradient, with constant 0.1% v/v TFA additive; 20 min run; 1 mL/min flow; ESI; positive ion mode; detection at 550 nm); HRMS (ESI) calcd for $C_{27}H_{11}D_{12}N_2O_5$ $[M+H]^+$ 467.2355, found 467.2354.

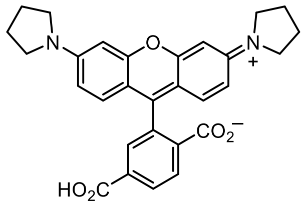

6-Carboxy-Rh$_P$ (S6). The title compound (99%, dark red-purple solid, TFA salt) was prepared from 6-*tert*-butoxycarbonyl-Rh$_P$ (**S3**) according to the procedure described for **S5**. ^{1}H NMR (CD_3OD, 400 MHz) δ 8.42 (d, *J* = 8.2 Hz, 1H), 8.39 (dd, *J* = 8.2, 1.5 Hz, 1H), 7.99 – 7.96 (m, 1H), 7.11 (d, *J* = 9.4 Hz, 2H), 6.92 (dd, *J* = 9.4, 2.3 Hz, 2H), 6.84 (d, *J* = 2.3 Hz, 2H), 3.68 – 3.56 (m, 8H), 2.21 – 2.07 (m, 8H); ^{13}C NMR (CD_3OD, 101 MHz) δ 167.7 (C), 167.4 (C), 160.0 (C), 158.9 (C), 156.2 (C), 136.1 (C), 135.9 (C), 135.5 (C), 132.8 (CH), 132.31 (CH), 132.28 (CH), 132.0 (CH), 116.4 (CH), 114.9 (C), 97.8 (CH), 50.0 (CH_2), 26.2 (CH_2); Analytical HPLC: t_R = 10.8 min, >99% purity (5 μL injection; 10–95% MeCN/H_2O, linear gradient, with constant 0.1% v/v TFA additive; 20 min run; 1 mL/min flow; ESI; positive ion mode; detection at 550 nm); HRMS (ESI) calcd for $C_{29}H_{27}N_2O_5$ $[M+H]^+$ 483.1914, found 483.1919.

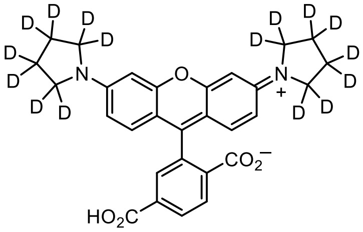

6-Carboxy-JFX$_{554}$ (S7). The title compound (97%, dark red-purple solid, TFA salt) was prepared from 6-*tert*-butoxycarbonyl-JFX$_{554}$ (**S4**) according to the procedure described for **S5**. ^{1}H NMR (CD_3OD, 400 MHz) δ 8.41 (d, *J* = 8.2 Hz, 1H), 8.38 (dd, *J* = 8.2, 1.5 Hz, 1H), 7.99 – 7.96 (m, 1H), 7.11 (d, *J* = 9.4 Hz, 2H), 6.91 (dd, *J* = 9.3, 2.3 Hz, 2H), 6.83 (d, *J* = 2.3 Hz, 2H); ^{13}C NMR (CD_3OD, 101 MHz) δ 167.7 (C), 167.4 (C), 159.9 (C), 158.9 (C), 156.4 (C), 136.1 (C), 135.9 (C), 135.5 (C), 132.8 (CH), 132.32 (CH), 132.27 (CH), 132.0 (CH), 116.4 (CH), 114.8 (C), 97.8 (CH); Analytical HPLC: t_R = 10.7 min, >99% purity (10–95% MeCN/H_2O, linear gradient, with constant 0.1% v/v TFA additive; 20 min run; 1 mL/min flow; ESI; positive ion mode; detection at 550 nm); HRMS (ESI) calcd for $C_{29}H_{11}D_{16}N_2O_5$ $[M+H]^+$ 499.2919, found 499.2922.

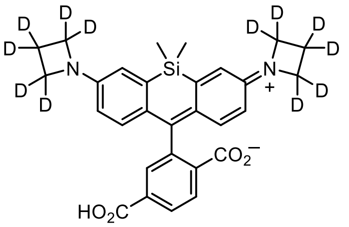

6-Carboxy-JFX$_{646}$ (S19). The title compound (98%, green solid, TFA salt) was prepared from 6-*tert*-butoxycarbonyl-JFX$_{646}$ (**S16**) according to the procedure described for **S5**. ^{1}H NMR (CD_3OD, 400 MHz) δ 8.29 – 8.25 (m, 2H), 7.80 (t, *J* = 1.0 Hz, 1H), 6.90 (d, *J* = 2.6 Hz, 2H), 6.87 (d, *J* = 9.2 Hz, 2H), 6.33 (dd, *J* = 9.2, 2.6 Hz, 2H), 0.60 (s, 3H), 0.53 (s, 3H); Analytical HPLC: t_R = 10.9 min, 97.8% purity (10–95% MeCN/H_2O, linear gradient, with constant 0.1% v/v TFA additive; 20 min run; 1 mL/min flow; ESI; positive ion mode; detection at 650 nm); HRMS (ESI) calcd for $C_{29}H_{17}D_{12}N_2O_4Si$ $[M+H]^+$ 509.2644, found 509.2648.

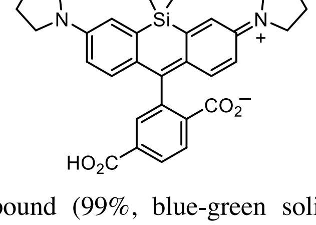

6-Carboxy-SiRh$_P$ (S20). The title compound (99%, blue-green solid, TFA salt) was prepared from 6-*tert*-butoxycarbonyl-SiRh$_P$ (**S17**) according to the procedure described for **S5**. ^{1}H NMR (CD_3OD, 400 MHz) δ 8.34 (d, *J* = 8.2 Hz, 1H), 8.29 (dd, *J* = 8.2, 1.7 Hz, 1H), 7.82 (d, *J* = 1.6 Hz, 1H), 7.20 (d, *J* = 2.7 Hz, 2H), 6.94 (d, *J* = 9.4 Hz, 2H), 6.61 (dd, *J* = 9.5, 2.7 Hz, 2H), 3.73 – 3.58 (m, 8H), 2.16 – 2.05 (m, 8H), 0.64 (s, 3H), 0.57 (s, 3H); ^{13}C NMR (CD_3OD, 101 MHz) δ 170.3 (C), 168.0 (C), 167.5 (C), 152.9 (C), 149.3 (C), 142.5 (C), 141.6 (CH), 136.1 (C), 135.2 (C), 132.5 (CH), 132.4 (CH), 131.0 (CH), 129.1 (C), 122.6 (CH), 115.7 (CH), 49.9 (CH_2), 26.1 (CH_2), -0.8 (CH_3), -1.8

(CH_3); Analytical HPLC: t_R = 11.5 min, >99% purity (10–95% $MeCN/H_2O$, linear gradient, with constant 0.1% v/v TFA additive; 20 min run; 1 mL/min flow; ESI; positive ion mode; detection at 650 nm); HRMS (ESI) calcd for $C_{31}H_{33}N_2O_4Si$ $[M+H]^+$ 525.2204, found 525.2214.

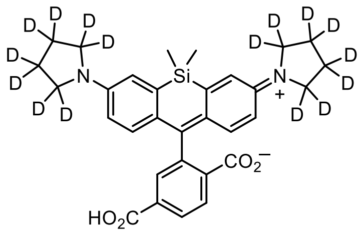

6-Carboxy-JFX$_{650}$ (S21). The title compound (~99%, blue-green solid, TFA salt) was prepared from 6-*tert*-butoxycarbonyl-JFX$_{650}$ (**S18**) according to the procedure described for **S5**. ^1H NMR (CD_3OD, 400 MHz) δ 8.34 (d, *J* = 8.1 Hz, 1H), 8.29 (dd, *J* = 8.2, 1.6 Hz, 1H), 7.83 – 7.81 (m, 1H), 7.19 (d, *J* = 2.7 Hz, 2H), 6.94 (d, *J* = 9.5 Hz, 2H), 6.60 (dd, *J* = 9.5, 2.7 Hz, 2H), 0.64 (s, 3H), 0.57 (s, 3H); ^{13}C NMR (CD_3OD, 101 MHz) δ 168.0 (C), 167.5 (C), 153.0 (C), 149.3 (C), 142.5 (C), 141.6 (CH), 136.1 (C), 135.2 (C), 132.5 (CH), 132.4 (CH), 131.0 (CH), 129.0 (C), 122.6 (CH), 115.7 (CH), -0.8 (CH_3), -1.8 (CH_3); Analytical HPLC: t_R = 11.5 min, >99% purity (10–95% $MeCN/H_2O$, linear gradient, with constant 0.1% v/v TFA additive; 20 min run; 1 mL/min flow; ESI; positive ion mode; detection at 650 nm); HRMS (ESI) calcd for $C_{31}H_{17}D_{16}N_2O_4Si$ $[M+H]^+$ 541.3208, found 541.3213.

N-Hydroxysuccinimidyl Ester Formation

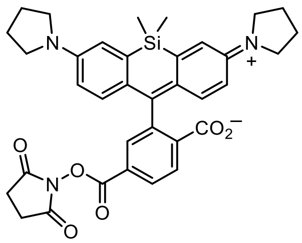

SiRh$_P$-NHS (S22). 6-Carboxy-SiRh$_P$ (**S20**; 244 mg, 0.382 mmol, TFA salt) was combined with DSC (235 mg, 0.917 mmol, 2.4 eq) in DMF (5 mL). After adding Et_3N (319 µL, 2.29 mmol, 6 eq) and DMAP (4.7 mg, 38.2 µmol, 0.1 eq), the reaction was stirred at room temperature for 30 min. It was subsequently diluted with 10% w/v citric acid and extracted with EtOAc (2×). The combined organic extracts were washed with brine, dried over anhydrous $MgSO_4$, filtered, and concentrated *in vacuo*. Silica gel chromatography (0–100% EtOAc/toluene, linear gradient) yielded 206 mg (87%) of **S22** as a blue-green solid. ^1H NMR ($CDCl_3$, 400 MHz) δ 8.27 (dd, *J* = 8.0, 1.4 Hz, 1H), 8.07 (dd, *J* = 8.0, 0.8 Hz, 1H), 8.00 (dd, *J* = 1.3, 0.8 Hz, 1H), 6.79 (d, *J* = 2.7 Hz, 2H), 6.74 (d, *J* = 8.8 Hz, 2H), 6.43 (dd, *J* = 8.9, 2.7 Hz, 2H), 3.35 – 3.25 (m, 8H), 2.88 (s, 4H), 2.04 – 1.94 (m, 8H), 0.64 (s, 3H), 0.58 (s, 3H); ^{13}C NMR ($CDCl_3$, 101 MHz) δ 169.6 (C), 168.9 (C), 161.2 (C), 155.8 (C), 147.0 (C), 136.9 (C), 132.0 (C), 130.6 (CH), 130.1 (C), 129.7 (C), 128.3 (CH), 126.8 (CH), 126.2 (CH), 116.1 (CH), 113.2 (CH), 92.9 (C), 47.6 (CH_2), 25.8 (CH_2), 25.6 (CH_2), 0.4 (CH_3), -1.1 (CH_3); Analytical HPLC: t_R = 12.2 min, 98.6% purity (10–95% MeCN/H_2O, linear gradient, with constant 0.1% v/v TFA additive; 20 min run; 1 mL/min flow; ESI; positive ion mode; detection at 650 nm); HRMS (ESI) calcd for $C_{35}H_{36}N_3O_6Si$ $[M+H]^+$ 622.2368, found 622.2369.

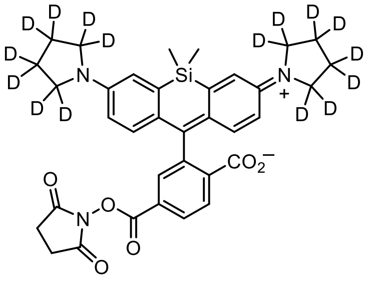

JFX$_{650}$-NHS (S23). The title compound (92%, blue-green solid) was prepared from 6-carboxy-JFX$_{650}$ (**S21**) according to the procedure described for **S22**. ^1H NMR ($CDCl_3$, 400 MHz) δ 8.27 (dd, *J* = 8.0, 1.4 Hz, 1H), 8.07 (dd, *J* = 8.0, 0.6 Hz, 1H), 8.01 (dd, *J* = 1.4, 0.8 Hz, 1H), 6.78 (d, *J* = 2.8 Hz, 2H), 6.73 (d, *J* = 8.8 Hz, 2H), 6.42 (dd, *J* = 8.8, 2.8 Hz, 2H), 2.89 (s, 4H), 0.64 (s, 3H), 0.58 (s, 3H); Analytical HPLC: t_R = 12.1 min, 98.8% purity (10–95% MeCN/H_2O, linear gradient, with constant 0.1% v/v TFA additive; 20 min run; 1 mL/min flow; ESI; positive ion mode; detection at 650 nm); HRMS (ESI) calcd for $C_{35}H_{20}D_{16}N_3O_6Si$ $[M+H]^+$ 638.3372, found 638.3380.

HaloTag Ligand Formation

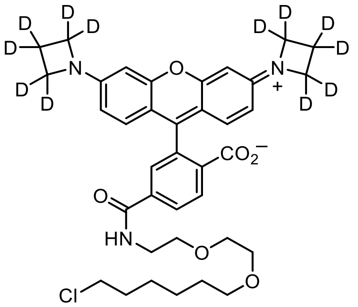

JFX_{549}–HaloTag ligand (20_D). 6-Carboxy-JFX_{549} (**S5**; 25 mg, 43.1 µmol, TFA salt) was combined with DSC (26.5 mg, 0.103 mmol, 2.4 eq) in DMF (1 mL). After adding Et_3N (36.0 µL, 0.258 mmol, 6 eq) and DMAP (0.5 mg, 4.3 µmol, 0.1 eq), the reaction was stirred at room temperature for 30 min. A solution of 2-(2-((6-chlorohexyl)oxy)ethoxy)-ethanamine (**S8**, 'HaloTag(O2)amine'; 43.6 mg, 0.129 mmol, 3 eq) in DMF (500 µL) was then added. The reaction was stirred an additional 2 h at room temperature. Purification of the crude reaction mixture by reverse phase HPLC (20–60% MeCN/H_2O, linear gradient, with constant 0.1% v/v TFA additive) afforded 22.4 mg (66%, TFA salt) of **20_D** as a dark red solid. ^{1}H NMR (CD_3OD, 400 MHz) δ 8.78 (t, J = 5.5 Hz, 1H), 8.39 (d, J = 8.2 Hz, 1H), 8.20 (dd, J = 8.2, 1.8 Hz, 1H), 7.80 (d, J = 1.7 Hz, 1H), 7.06 (d, J = 9.2 Hz, 2H), 6.60 (dd, J = 9.2, 2.2 Hz, 2H), 6.55 (d, J = 2.2 Hz, 2H), 3.69 – 3.55 (m, 8H), 3.53 (t, J = 6.6 Hz, 2H), 3.43 (t, J = 6.5 Hz, 2H), 1.76 – 1.67 (m, 2H), 1.55 – 1.46 (m, 2H), 1.45 – 1.28 (m, 4H); Analytical HPLC: t_R = 12.3 min, >99% purity (10–95% MeCN/H_2O, linear gradient, with constant 0.1% v/v TFA additive; 20 min run; 1 mL/min flow; ESI; positive ion mode; detection at 550 nm); HRMS (ESI) calcd for $C_{37}H_{31}D_{12}ClN_3O_6$ $[M+H]^+$ 672.3588, found 672.3590.

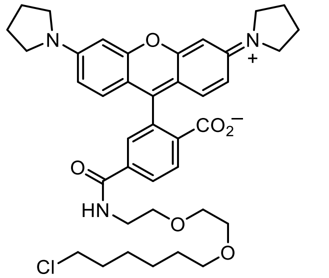

Rh_P–HaloTag ligand (21). The title compound (58%, dark red-purple solid, TFA salt) was prepared from 6-carboxy-Rh_P (**S6**) and 2-(2-((6-chlorohexyl)oxy)ethoxy)ethanamine (**S8**) according to the procedure described for **20_D**. ^{1}H NMR (CD_3OD, 400 MHz) δ 8.76 (t, J = 5.3 Hz, 1H), 8.40 (d, J = 8.2 Hz, 1H), 8.21 (dd, J = 8.3, 1.8 Hz, 1H), 7.83 (d, J = 1.7 Hz, 1H), 7.12 (d, J = 9.3 Hz, 2H), 6.91 (dd, J = 9.3, 2.3 Hz, 2H), 6.84 (d, J = 2.3 Hz, 2H), 3.71 – 3.54 (m, 16H), 3.51 (d, J = 6.6 Hz, 2H), 3.43 (t, J = 6.5 Hz, 2H), 2.20 – 2.05 (m, 8H), 1.76 – 1.66 (m, 2H), 1.54 – 1.45 (m, 2H), 1.45 – 1.26 (m, 4H); Analytical HPLC: t_R = 13.2 min, 98.4% purity (10–95% MeCN/H_2O, linear gradient, with constant 0.1% v/v TFA additive; 20 min run; 1 mL/min flow; ESI; positive ion mode; detection at 550 nm); HRMS (ESI) calcd for $C_{39}H_{47}ClN_3O_6$ $[M+H]^+$ 688.3148, found 688.3156.

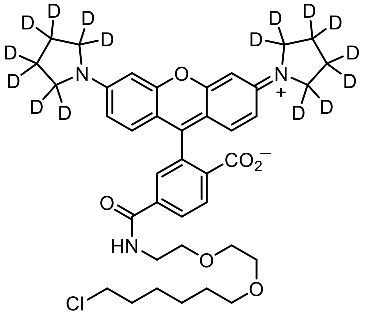

JFX_{554}–HaloTag ligand (21_D). The title compound (63%, dark red-purple solid, TFA salt) was prepared from 6-carboxy-JFX_{554} (**S7**) and 2-(2-((6-chlorohexyl)oxy)ethoxy)ethanamine (**S8**) according to the procedure described for **20_D**. ^1H NMR (CD_3OD, 400 MHz) δ 8.76 (t, *J* = 5.2 Hz, 1H), 8.40 (d, *J* = 8.2 Hz, 1H), 8.21 (dd, *J* = 8.3, 1.8 Hz, 1H), 7.83 (d, *J* = 1.7 Hz, 1H), 7.12 (d, *J* = 9.3 Hz, 2H), 6.91 (dd, *J* = 9.3, 2.3 Hz, 2H), 6.84 (d, *J* = 2.3 Hz, 2H), 3.70 – 3.54 (m, 8H), 3.52 (t, *J* = 6.6 Hz, 2H), 3.43 (t, *J* = 6.5 Hz, 2H), 1.76 – 1.66 (m, 2H), 1.55 – 1.45 (m, 2H), 1.45 – 1.27 (m, 4H); Analytical HPLC: t_R = 13.1 min, 98.4% purity (10–95% $MeCN/H_2O$, linear gradient, with constant 0.1% v/v TFA additive; 20 min run; 1 mL/min flow; ESI; positive ion mode; detection at 550 nm); HRMS (ESI) calcd for $C_{39}H_{31}D_{16}ClN_3O_6$ $[M+H]^+$ 704.4152, found 704.4155.

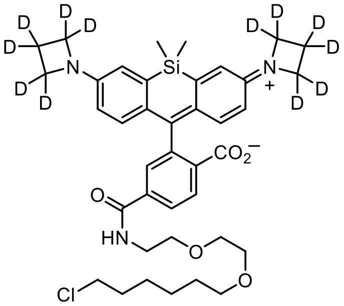

JFX_{646}–HaloTag ligand (30_D). The title compound (79%, pale blue-green solid) was prepared from 6-carboxy-JFX_{646} (**S19**) and 2-(2-((6-chlorohexyl)oxy)ethoxy)ethanamine (**S8**) according to the procedure described for **20_D**. ^1H NMR ($CDCl_3$, 400 MHz) δ 7.98 (d, *J* = 7.9 Hz, 1H), 7.90 (dd, *J* = 8.0, 1.4 Hz, 1H), 7.70 – 7.66 (m, 1H), 6.76 (s, 1H), 6.75 (d, *J* = 8.7 Hz, 2H), 6.66 (d, *J* = 2.7 Hz, 2H), 6.26 (dd, *J* = 8.6, 2.7 Hz, 2H), 3.67 – 3.59 (m, 6H), 3.56 – 3.53 (m, 2H), 3.50 (t, *J* = 6.6 Hz, 2H), 3.39 (t, *J* = 6.7 Hz, 2H), 1.76 – 1.69 (m, 2H), 1.54 – 1.48 (m, 2H), 1.44 – 1.26 (m, 4H), 0.63 (s, 3H), 0.57 (s, 3H); Analytical HPLC: t_R = 13.2 min, 98.7% purity (10–95% $MeCN/H_2O$, linear gradient, with constant 0.1% v/v TFA additive; 20 min run; 1 mL/min flow; ESI; positive ion mode; detection at 650 nm); HRMS (ESI) calcd for $C_{39}H_{37}D_{12}ClN_3O_5Si$ $[M+H]^+$ 714.3878, found 714.3885.

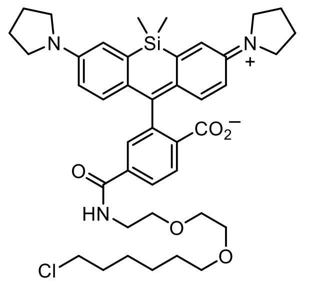

SiRh$_P$–HaloTag ligand (31). SiRh$_P$-NHS (**S22**; 75 mg, 0.121 mmol) and 2-(2-((6-chlorohexyl)oxy)ethoxy)ethanamine (**S8**; 61.1 mg, 0.181 mmol, 1.5 eq) were combined in DMF (3 mL), and DIEA (63.0 μL, 0.362 mmol, 3 eq) was added. After stirring the reaction at room temperature for 1 h, it was diluted with saturated $NaHCO_3$ and extracted with EtOAc (2×). The combined organic extracts were washed with water and brine, dried over anhydrous $MgSO_4$, filtered, and evaporated. Purification of the crude product by silica gel chromatography (10–100% EtOAc/toluene, linear gradient) provided **31** as a pale blue-green solid (64.4 mg, 73%). ^1H NMR ($CDCl_3$, 400 MHz) δ 7.99 (d, *J* = 7.9 Hz, 1H), 7.91 (dd, *J* = 7.9, 1.4 Hz, 1H), 7.67 – 7.64 (m, 1H), 6.79 (d, *J* = 2.7 Hz, 2H), 6.75 (s, 1H), 6.75 (d, *J* = 8.8 Hz, 2H), 6.39 (dd, *J* = 8.9, 2.7 Hz, 2H), 3.67 – 3.58 (m, 6H), 3.57 – 3.52 (m, 2H), 3.50 (t, *J* = 6.7 Hz, 2H), 3.39 (t, *J* = 6.7 Hz, 2H), 3.35 – 3.24 (m, 8H), 2.05 – 1.93 (m, 8H), 1.77 – 1.68 (m, 2H), 1.55 – 1.47 (m, 2H), 1.44 – 1.26 (m, 4H), 0.65 (s, 3H), 0.59 (s, 3H); Analytical HPLC: t_R = 13.5 min, >99% purity (10–95% $MeCN/H_2O$, linear gradient, with constant 0.1% v/v TFA additive; 20 min run; 1 mL/min flow; ESI; positive ion mode; detection at 650 nm); HRMS (ESI) calcd for $C_{41}H_{53}ClN_3O_5Si$ $[M+H]^+$ 730.3438, found 730.3442.

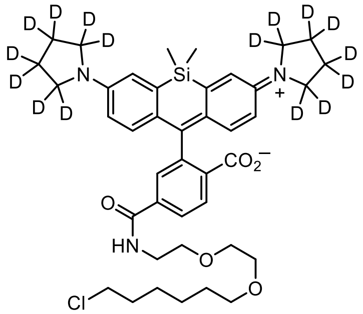

JFX$_{650}$–HaloTag ligand (31$_D$). The title compound (77%, pale blue-green solid) was prepared from JFX$_{650}$-NHS (**S23**) and 2-(2-((6-chlorohexyl)oxy)ethoxy)ethanamine (**S8**) according to the procedure described for **31**. ^1H NMR ($CDCl_3$, 400 MHz) δ 7.99 (dd, *J* = 8.0, 0.5 Hz, 1H), 7.91 (dd, *J* = 8.0, 1.4 Hz, 1H), 7.67 – 7.63 (m, 1H), 6.78 (d, *J* = 2.8 Hz, 2H), 6.747 (d, *J* = 8.8 Hz, 2H), 6.744 (s, 1H), 6.39 (dd, *J* = 8.8, 2.8 Hz, 2H), 3.66 – 3.58 (m, 6H), 3.57 – 3.52 (m, 2H), 3.50 (t, *J* = 6.7 Hz, 2H), 3.39 (t, *J* = 6.7 Hz, 2H), 1.77 – 1.68 (m, 2H), 1.55 – 1.47 (m, 2H), 1.44 – 1.35 (m, 2H), 1.34 – 1.25 (m, 2H), 0.65 (s, 3H), 0.58 (s, 3H); Analytical HPLC: t_R = 13.4 min, >99% purity (10–95% $MeCN/H_2O$, linear gradient, with constant 0.1% v/v TFA additive; 20 min run; 1 mL/min flow; ESI; positive ion mode; detection at 650 nm); HRMS (ESI) calcd for $C_{41}H_{37}D_{16}ClN_3O_5Si$ $[M+H]^+$ 746.4442, found 746.4449.

References

1. Grimm, J. B.; Lavis, L. D., Synthesis of rhodamines from fluoresceins using Pd-catalyzed C-N cross-coupling. *Org. Lett.* **2011,** *13*, 6354–6357.
2. Grimm, J. B.; Sung, A. J.; Legant, W. R.; Hulamm, P.; Matlosz, S. M.; Betzig, E.; Lavis, L. D., Carbofluoresceins and carborhodamines as scaffolds for high-contrast fluorogenic probes. *ACS Chem. Biol.* **2013,** *8*, 1303–1310.
3. Grimm, J. B.; English, B. P.; Chen, J.; Slaughter, J. P.; Zhang, Z.; Revyakin, A.; Patel, R.; Macklin, J. J.; Normanno, D.; Singer, R. H.; Lionnet, T.; Lavis, L. D., A general method to improve fluorophores for live-cell and single-molecule microscopy. *Nat. Methods* **2015,** *12*, 244–250.
4. Grimm, J. B.; Brown, T. A.; Tkachuk, A. N.; Lavis, L. D., General synthetic method for Si-fluoresceins and Si-rhodamines. *ACS Cent. Sci.* **2017,** *3*, 975–985.
5. Suzuki, K.; Kobayashi, A.; Kaneko, S.; Takehira, K.; Yoshihara, T.; Ishida, H.; Shiina, Y.; Oishi, S.; Tobita, S., Reevaluation of absolute luminescence quantum yields of standard solutions using a spectrometer with an integrating sphere and a back-thinned CCD detector. *Phys. Chem. Chem. Phys.* **2009,** *11*, 9850–9860.
6. Hinckley, D. A.; Seybold, P. G., A spectroscopic/thermodynamic study of the rhodamine B lactone ⇌ zwitterion equilibrium. *Spectrochim. Acta, Part A* **1988,** *44*, 1053–1059.
7. Grimm, J. B.; Muthusamy, A. K.; Liang, Y.; Brown, T. A.; Lemon, W. C.; Patel, R.; Lu, R.; Macklin, J. J.; Keller, P. J.; Ji, N.; Lavis, L. D., A general method to fine-tune fluorophores for live-cell and in vivo imaging. *Nat. Methods* **2017,** *14*, 987–994.
8. Schindelin, J.; Arganda-Carreras, I.; Frise, E.; Kaynig, V.; Longair, M.; Pietzsch, T.; Preibisch, S.; Rueden, C.; Saalfeld, S.; Schmid, B.; Tinevez, J. Y.; White, D. J.; Hartenstein, V.; Eliceiri, K.; Tomancak, P.; Cardona, A., Fiji: An open-source platform for biological-image analysis. *Nat. Methods* **2012,** *9*, 676–682.
9. Grimm, J. B.; English, B. P.; Choi, H.; Muthusamy, A. K.; Mehl, B. P.; Dong, P.; Brown, T. A.; Lippincott-Schwartz, J.; Liu, Z.; Lionnet, T.; Lavis, L. D., Bright photoactivatable fluorophores for single-molecule imaging. *Nat. Methods* **2016,** *13*, 985–988.
10. Xie, L.; Torigoe, S. E.; Xiao, J.; Mai, D. H.; Li, L.; Davis, F. P.; Dong, P.; Marie-Nelly, H.; Grimm, J.; Lavis, L.; Darzacq, X.; Cattoglio, C.; Liu, Z.; Tjian, R., A dynamic interplay of enhancer elements regulates Klf4 expression in naïve pluripotency. *Genes Dev.* **2017,** *31*, 1795–1808.
11. Chen, J.; Zhang, Z.; Li, L.; Chen, B.-C.; Revyakin, A.; Hajj, B.; Legant, W.; Dahan, M.; Lionnet, T.; Betzig, E.; Tjian, R.; Liu, Z., Single-molecule dynamics of enhanceosome assembly in embryonic stem cells. *Cell* **2014,** *156*, 1274–1285.
12. Sergé, A.; Bertaux, N.; Rigneault, H.; Marguet, D., Dynamic multiple-target tracing to probe spatiotemporal cartography of cell membranes. *Nat. Methods* **2008,** *5*, 687–694.
13. Kövér, J.; Antus, S., Facile deoxygenation of hydroxylated flavonoids by palladium-catalysed reduction of its triflate derivatives. *Z. Naturforsch. B* **2005,** *60*, 792–796.
14. Helal, C. J.; Chappie, T. A.; Humphrey, J. M. Pyrazolo[3,4-d]pyrimidine compounds and their use as pde2 inhibitors and/or cyp3a4 inhibitors. Int. Pat. Appl. WO 2012/168817 A1, Dec 13, 2012, 2012.

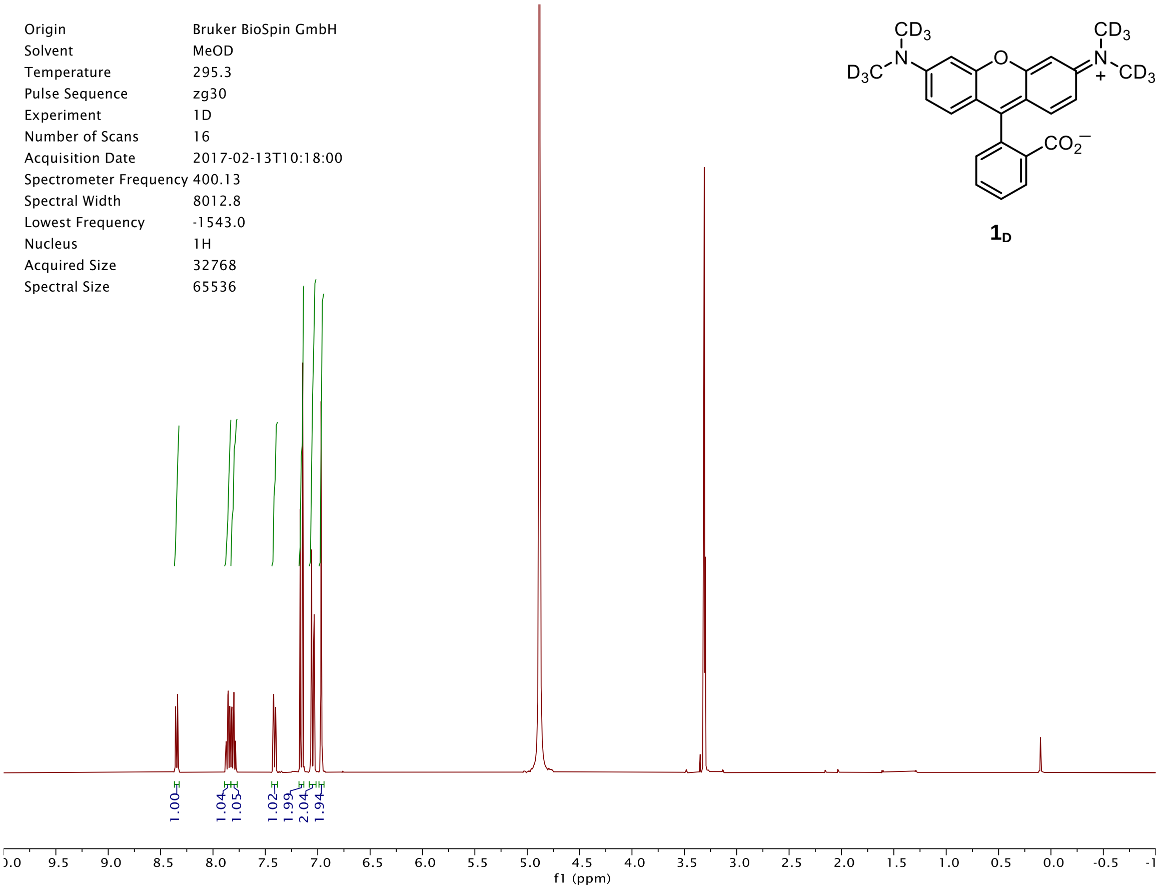
Origin Bruker BioSpin GmbH
Solvent MeOD
Temperature 295.3
Pulse Sequence zg30
Experiment 1D
Number of Scans 16
Acquisition Date 2017-02-13T10:18:00
Spectrometer Frequency 400.13
Spectral Width 8012.8
Lowest Frequency -1543.0
Nucleus 1H
Acquired Size 32768
Spectral Size 65536
1.00
1.04
1.05
1.02
1.99
2.04
1.94
f1 (ppm)

1$_D$

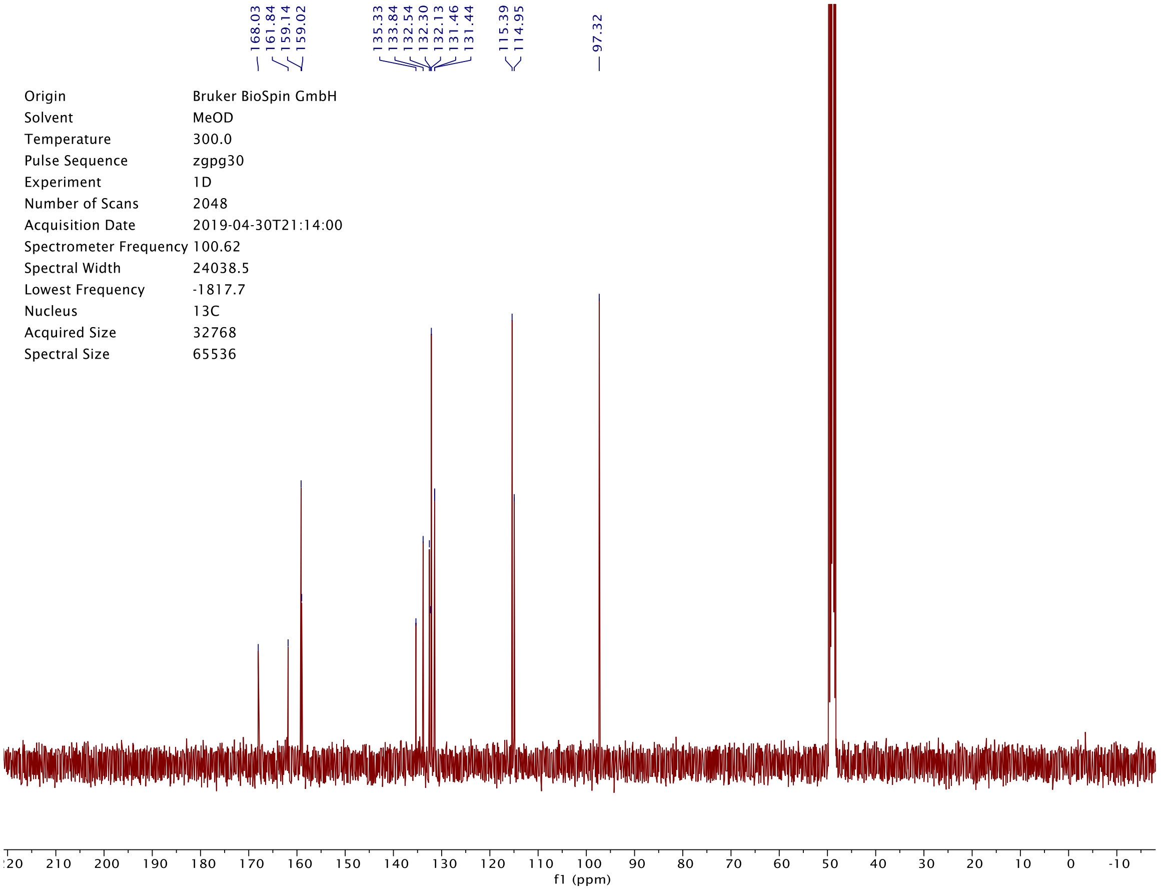
168.03
161.84
159.14
159.02
135.33
133.84
132.54
132.30
132.13
131.46
131.44
115.39
114.95
97.32
Origin Bruker BioSpin GmbH
Solvent MeOD
Temperature 300.0
Pulse Sequence zgpg30
Experiment 1D
Number of Scans 2048
Acquisition Date 2019-04-30T21:14:00
Spectrometer Frequency 100.62
Spectral Width 24038.5
Lowest Frequency -1817.7
Nucleus 13C
Acquired Size 32768
Spectral Size 65536
f1 (ppm)

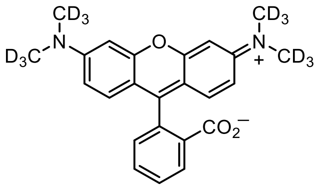
CD3
D3C
N
O
CD3
N
+
CD3
CO2−

1$_D$

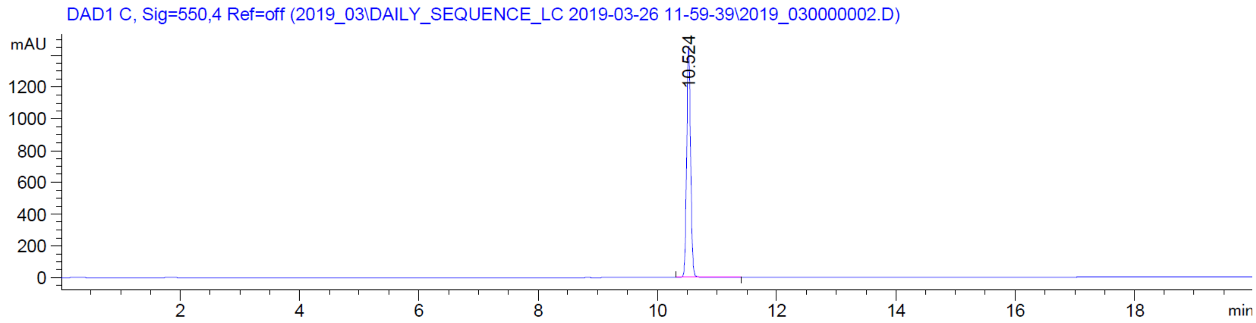
DAD1 C, Sig=550,4 Ref=off (2019_03\DAILY_SEQUENCE_LC 2019-03-26 11-59-39\2019_030000002.D)
mAU
10.524
min

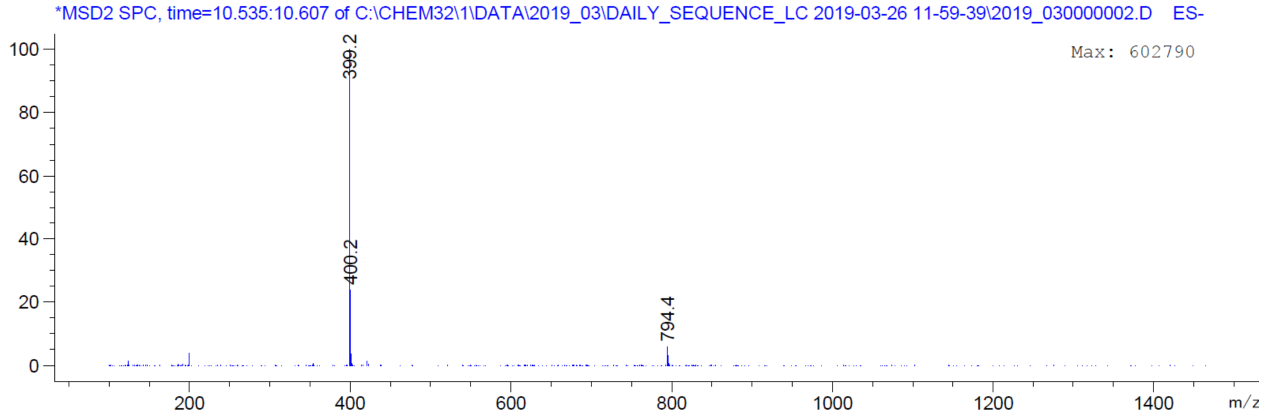
*MSD2 SPC, time=10.535:10.607 of C:\CHEM32\1\DATA\2019_03\DAILY_SEQUENCE_LC 2019-03-26 11-59-39\2019_030000002.D ES-
Max: 602790
399.2
400.2
794.4
m/z

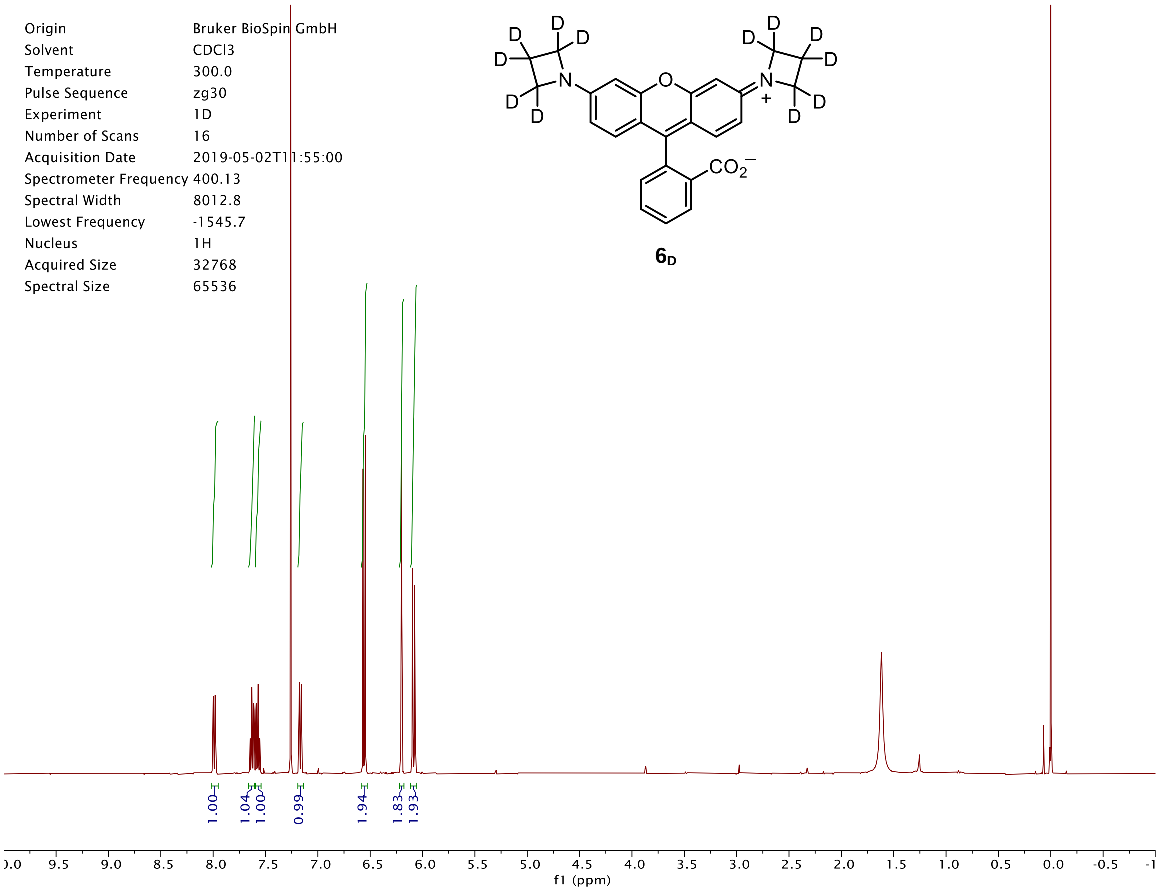
Origin Bruker BioSpin GmbH
Solvent CDCl3
Temperature 300.0
Pulse Sequence zg30
Experiment 1D
Number of Scans 16
Acquisition Date 2019-05-02T11:55:00
Spectrometer Frequency 400.13
Spectral Width 8012.8
Lowest Frequency -1545.7
Nucleus 1H
Acquired Size 32768
Spectral Size 65536
6_D
1.00
1.04
1.00
0.99
1.94
1.83
1.93
f1 (ppm)

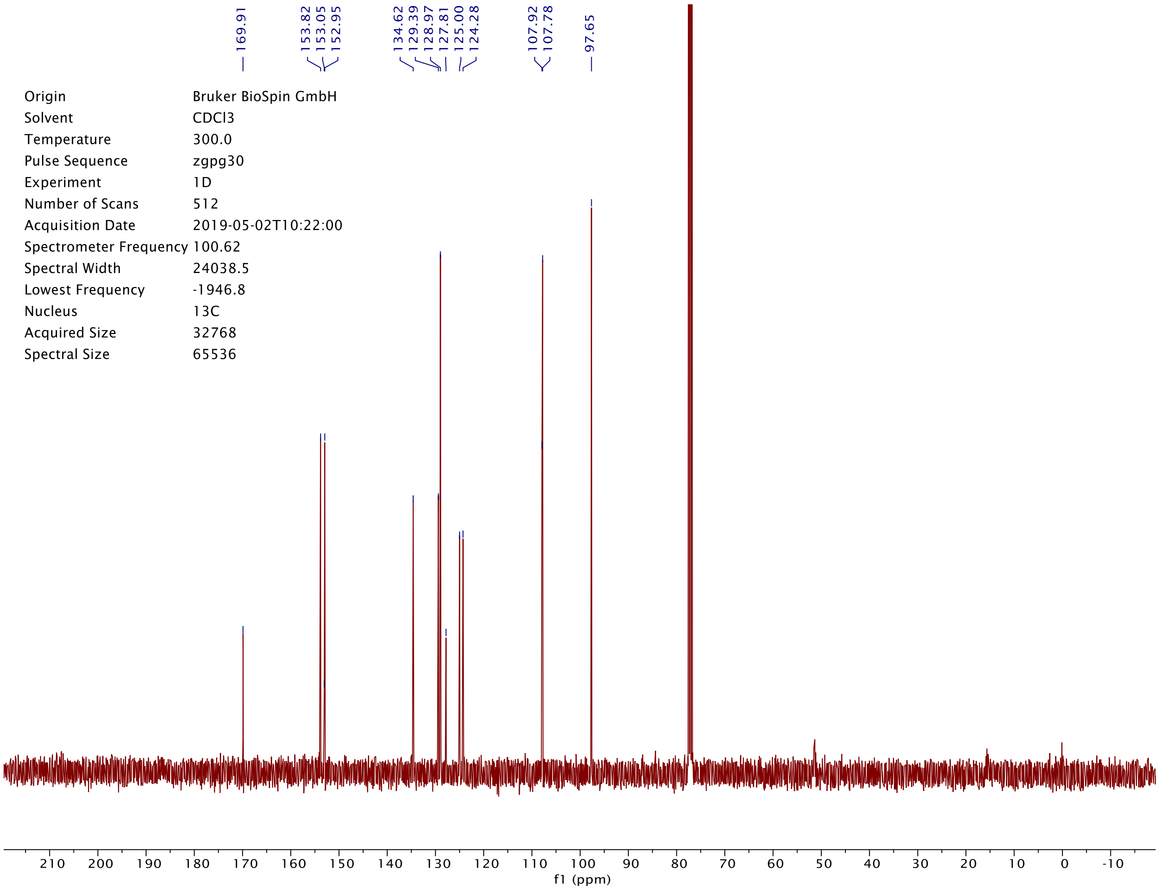
169.91
153.82
153.05
152.95
134.62
129.39
128.97
127.81
125.00
124.28
107.92
107.78
97.65
Origin Bruker BioSpin GmbH
Solvent CDCl3
Temperature 300.0
Pulse Sequence zgpg30
Experiment 1D
Number of Scans 512
Acquisition Date 2019-05-02T10:22:00
Spectrometer Frequency 100.62
Spectral Width 24038.5
Lowest Frequency -1946.8
Nucleus 13C
Acquired Size 32768
Spectral Size 65536
f1 (ppm)

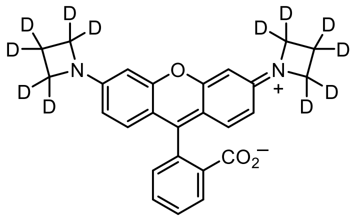

6$_D$

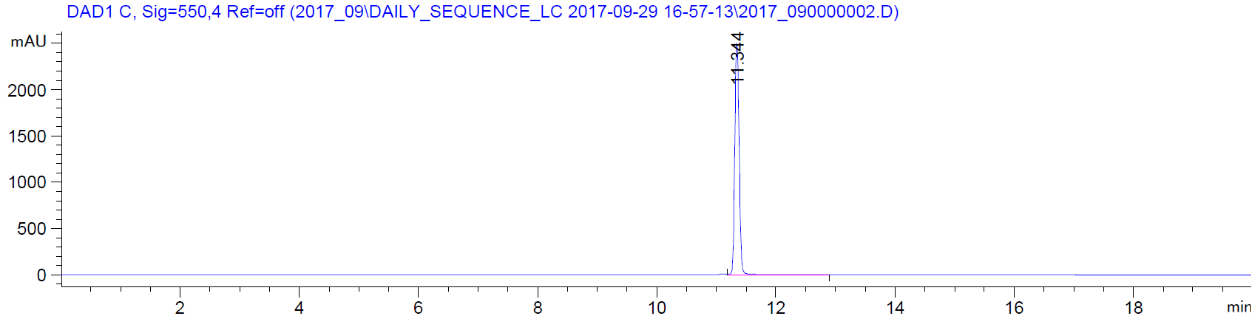
DAD1 C, Sig=550,4 Ref=off (2017_09\DAILY_SEQUENCE_LC 2017-09-29 16-57-13\2017_090000002.D)
mAU
2000
1500
1000
500
0
11.344
2
4
6
8
10
12
14
16
18
min

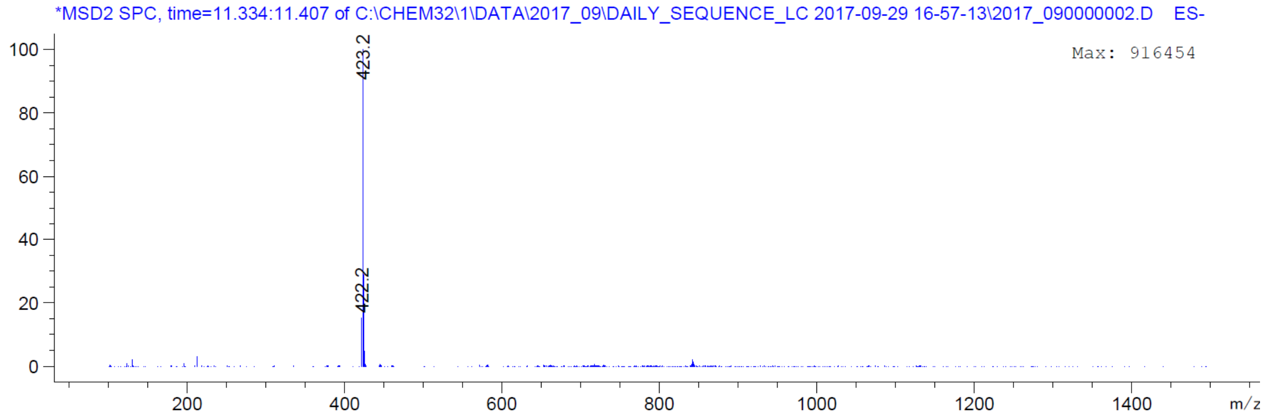
*MSD2 SPC, time=11.334:11.407 of C:\CHEM32\1\DATA\2017_09\DAILY_SEQUENCE_LC 2017-09-29 16-57-13\2017_090000002.D ES-
Max: 916454
100
80
60
40
20
0
423.2
422.2
200
400
600
800
1000
1200
1400
m/z

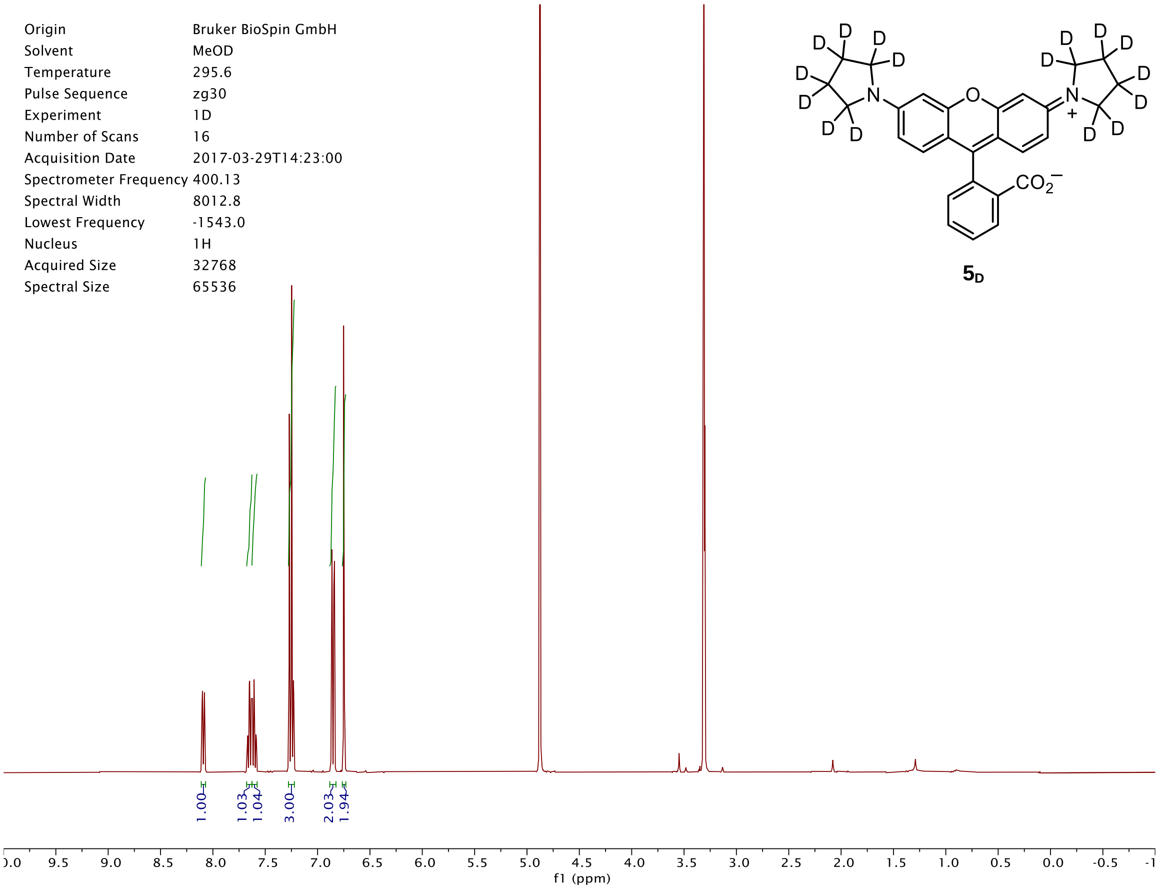
Origin Bruker BioSpin GmbH
Solvent MeOD
Temperature 295.6
Pulse Sequence zg30
Experiment 1D
Number of Scans 16
Acquisition Date 2017-03-29T14:23:00
Spectrometer Frequency 400.13
Spectral Width 8012.8
Lowest Frequency -1543.0
Nucleus 1H
Acquired Size 32768
Spectral Size 65536
5_D
1.00
1.03
1.04
3.00
2.03
1.94
f1 (ppm)

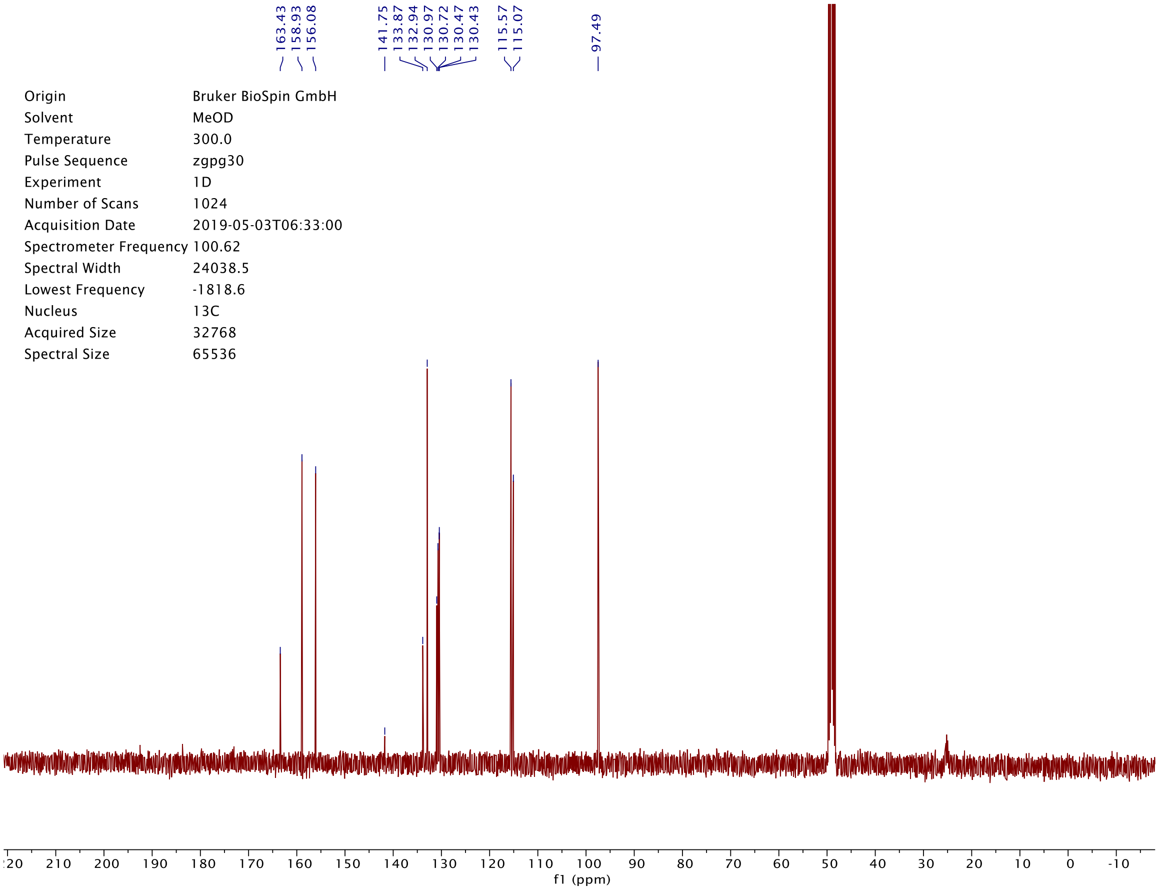
163.43
158.93
156.08
141.75
133.87
132.94
130.97
130.72
130.47
130.43
115.57
115.07
97.49
Origin Bruker BioSpin GmbH
Solvent MeOD
Temperature 300.0
Pulse Sequence zgpg30
Experiment 1D
Number of Scans 1024
Acquisition Date 2019-05-03T06:33:00
Spectrometer Frequency 100.62
Spectral Width 24038.5
Lowest Frequency -1818.6
Nucleus 13C
Acquired Size 32768
Spectral Size 65536
f1 (ppm)

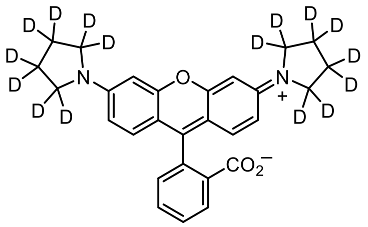

5$_D$

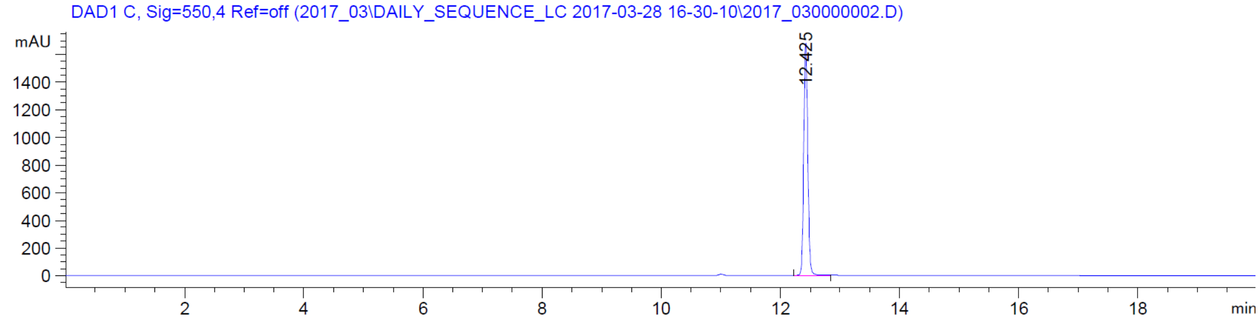

DAD1 C, Sig=550,4 Ref=off (2017_03\DAILY_SEQUENCE_LC 2017-03-28 16-30-10\2017_030000002.D)
mAU
12.425
min

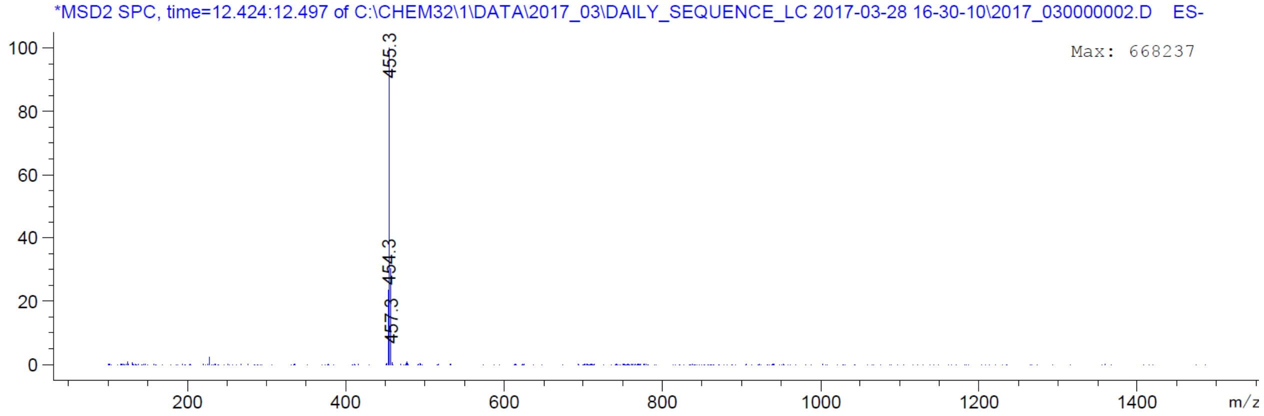

*MSD2 SPC, time=12.424:12.497 of C:\CHEM32\1\DATA\2017_03\DAILY_SEQUENCE_LC 2017-03-28 16-30-10\2017_030000002.D ES-
Max: 668237
455.3
454.3
457.3
m/z

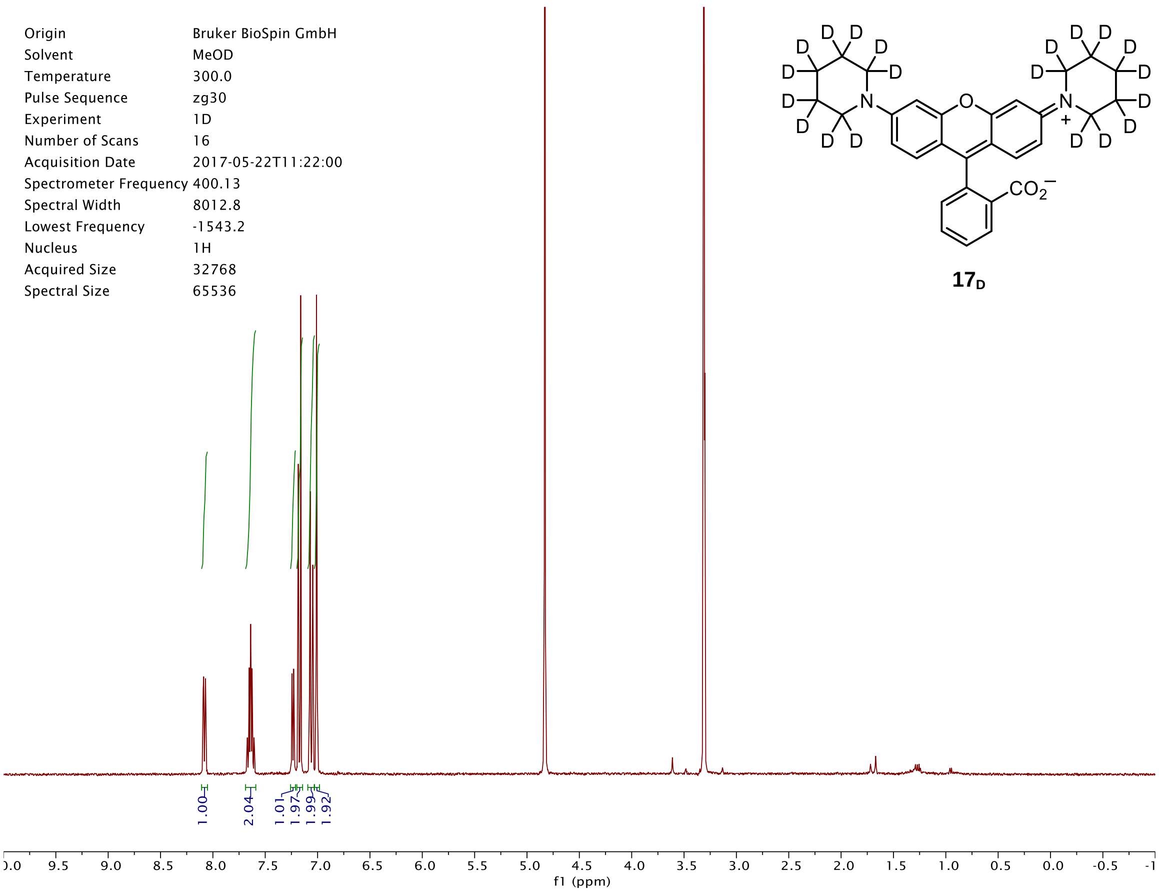

Origin Bruker BioSpin GmbH
Solvent MeOD
Temperature 300.0
Pulse Sequence zg30
Experiment 1D
Number of Scans 16
Acquisition Date 2017-05-22T11:22:00
Spectrometer Frequency 400.13
Spectral Width 8012.8
Lowest Frequency -1543.2
Nucleus 1H
Acquired Size 32768
Spectral Size 65536
1.00
2.04
1.01
1.97
1.99
1.92
f1 (ppm)

17_D

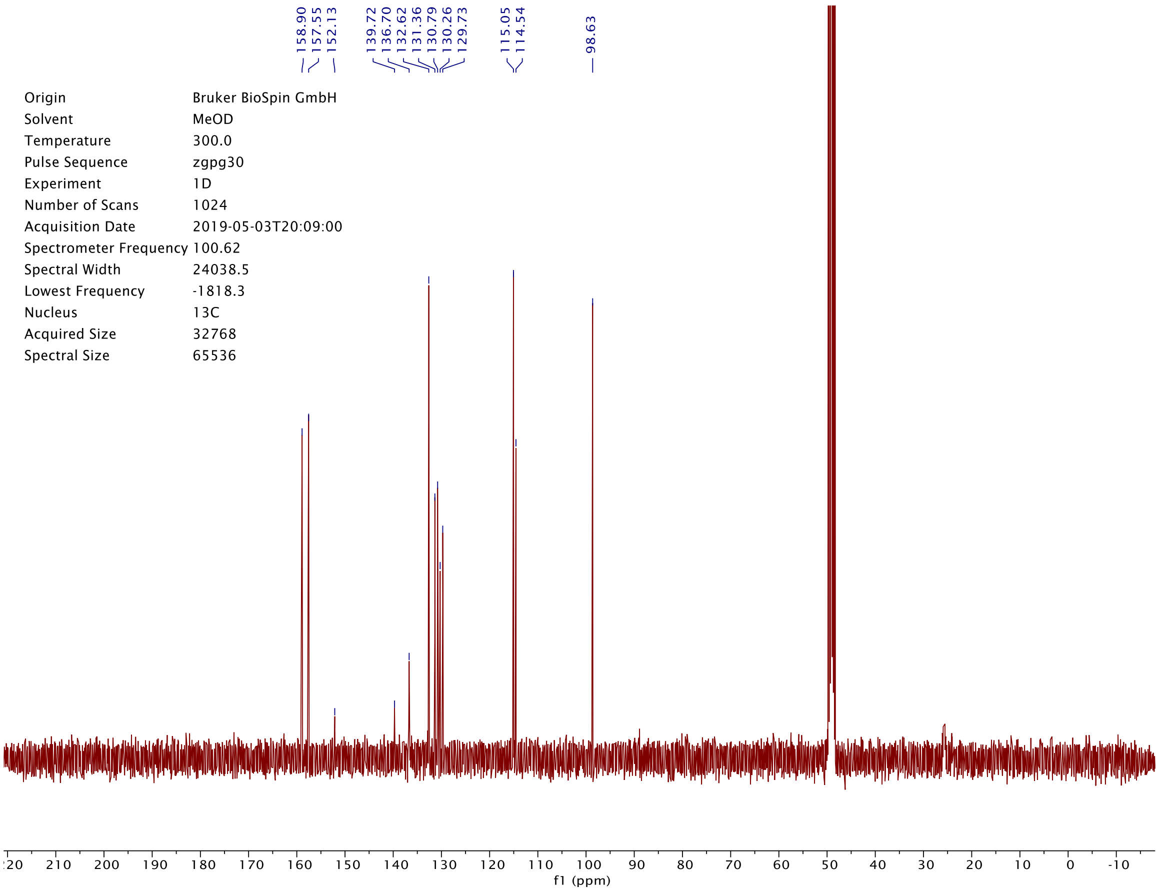

158.90
157.55
152.13
139.72
136.70
132.62
131.36
130.79
130.26
129.73
115.05
114.54
98.63
Origin Bruker BioSpin GmbH
Solvent MeOD
Temperature 300.0
Pulse Sequence zgpg30
Experiment 1D
Number of Scans 1024
Acquisition Date 2019-05-03T20:09:00
Spectrometer Frequency 100.62
Spectral Width 24038.5
Lowest Frequency -1818.3
Nucleus 13C
Acquired Size 32768
Spectral Size 65536
f1 (ppm)

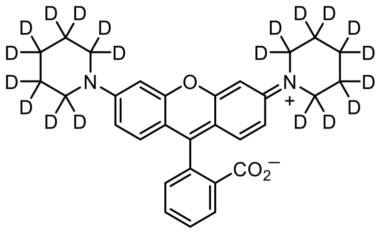

17_D

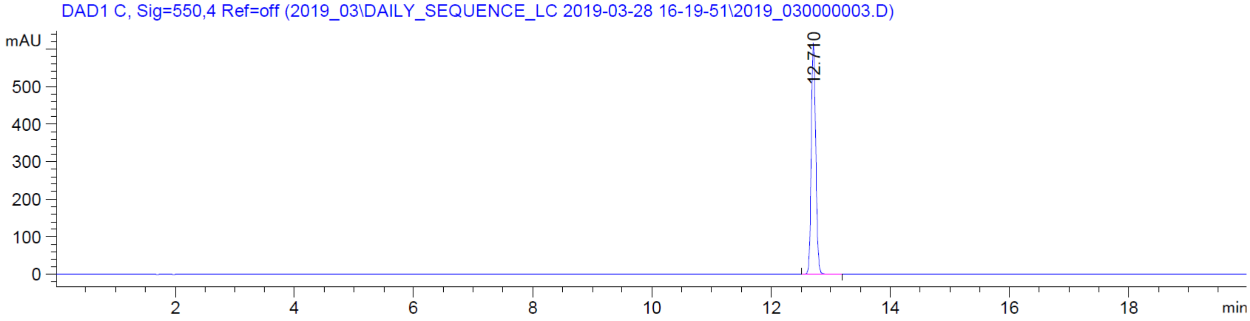
DAD1 C, Sig=550,4 Ref=off (2019_03\DAILY_SEQUENCE_LC 2019-03-28 16-19-51\2019_030000003.D)
mAU
12.710
min

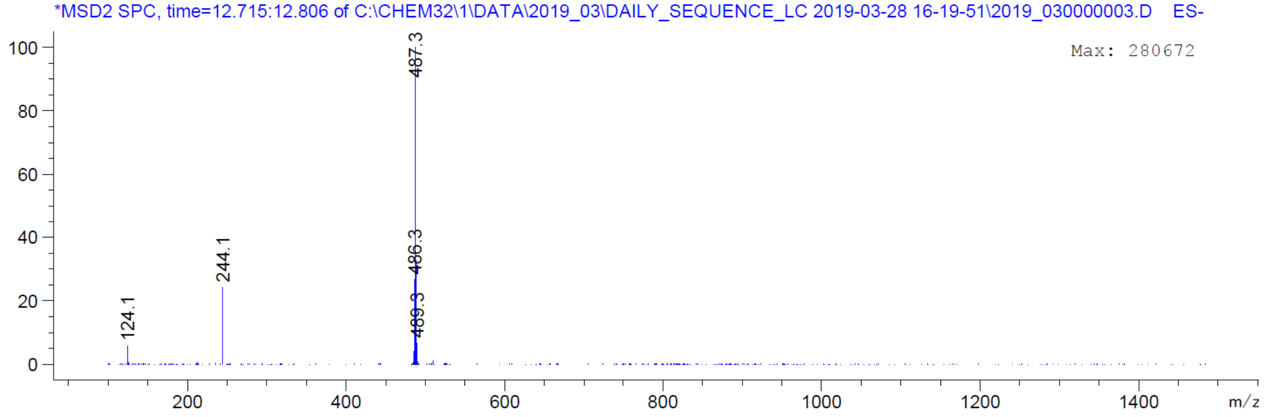
*MSD2 SPC, time=12.715:12.806 of C:\CHEM32\1\DATA\2019_03\DAILY_SEQUENCE_LC 2019-03-28 16-19-51\2019_030000003.D ES-
Max: 280672
487.3
486.3
489.3
244.1
124.1
m/z

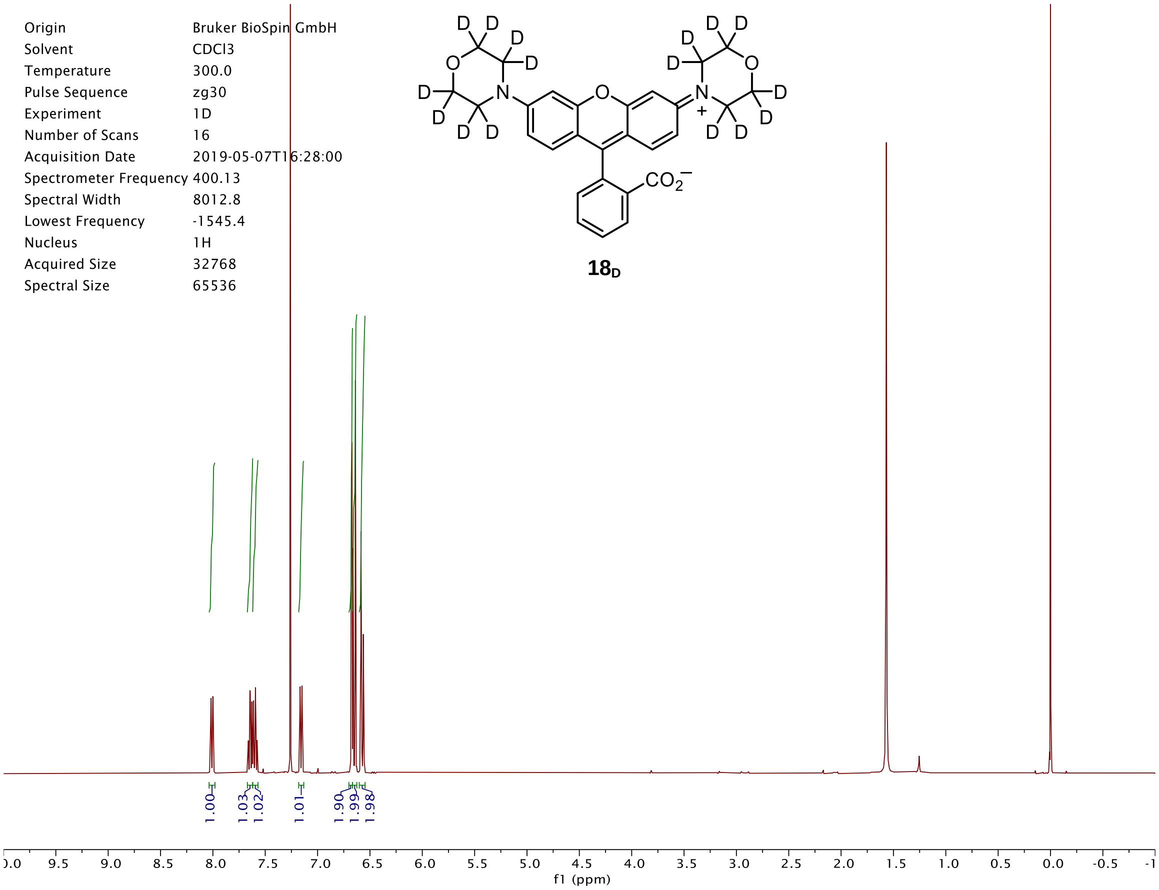
Origin Bruker BioSpin GmbH
Solvent CDCl3
Temperature 300.0
Pulse Sequence zg30
Experiment 1D
Number of Scans 16
Acquisition Date 2019-05-07T16:28:00
Spectrometer Frequency 400.13
Spectral Width 8012.8
Lowest Frequency -1545.4
Nucleus 1H
Acquired Size 32768
Spectral Size 65536
1.00
1.03
1.02
1.01
1.90
1.99
1.98
9.5 9.0 8.5 8.0 7.5 7.0 6.5 6.0 5.5 5.0 4.5 4.0 3.5 3.0 2.5 2.0 1.5 1.0 0.5 0.0 -0.5
f1 (ppm)
D D D D D D D D D D D D D D D D
O N O N
+
CO_2^-

18_D

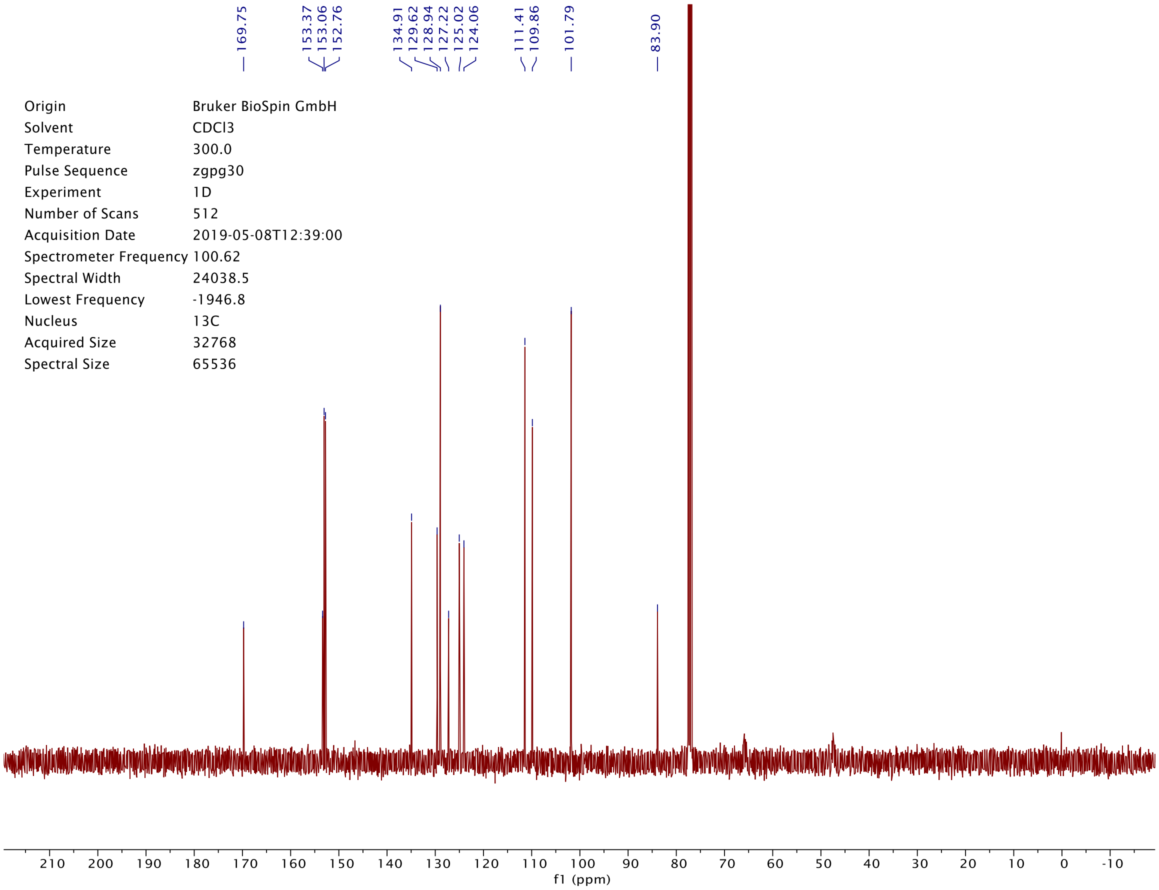
169.75
153.37
153.06
152.76
134.91
129.62
128.94
127.22
125.02
124.06
111.41
109.86
101.79
83.90
Origin Bruker BioSpin GmbH
Solvent CDCl3
Temperature 300.0
Pulse Sequence zgpg30
Experiment 1D
Number of Scans 512
Acquisition Date 2019-05-08T12:39:00
Spectrometer Frequency 100.62
Spectral Width 24038.5
Lowest Frequency -1946.8
Nucleus 13C
Acquired Size 32768
Spectral Size 65536
210 200 190 180 170 160 150 140 130 120 110 100 90 80 70 60 50 40 30 20 10 0 -10
f1 (ppm)

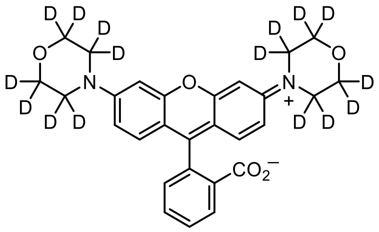

18_D

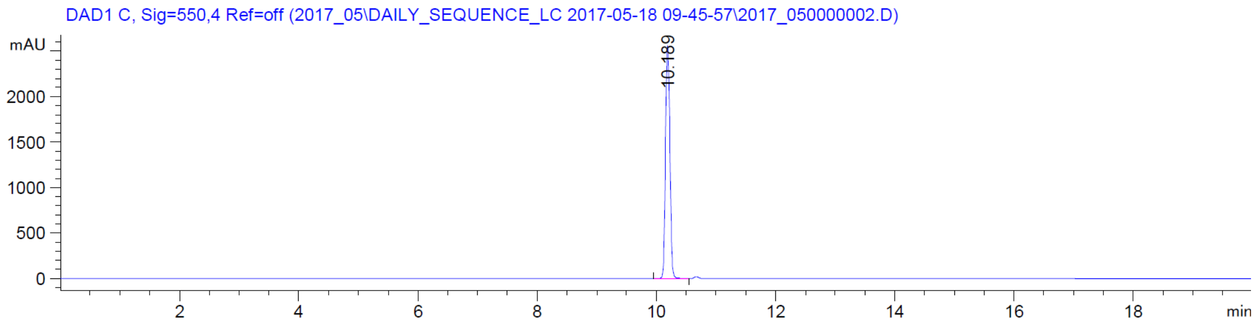

DAD1 C, Sig=550,4 Ref=off (2017_05\DAILY_SEQUENCE_LC 2017-05-18 09-45-57\2017_050000002.D)
mAU
2000
1500
1000
500
0
2
4
6
8
10
12
14
16
18
min
10.189

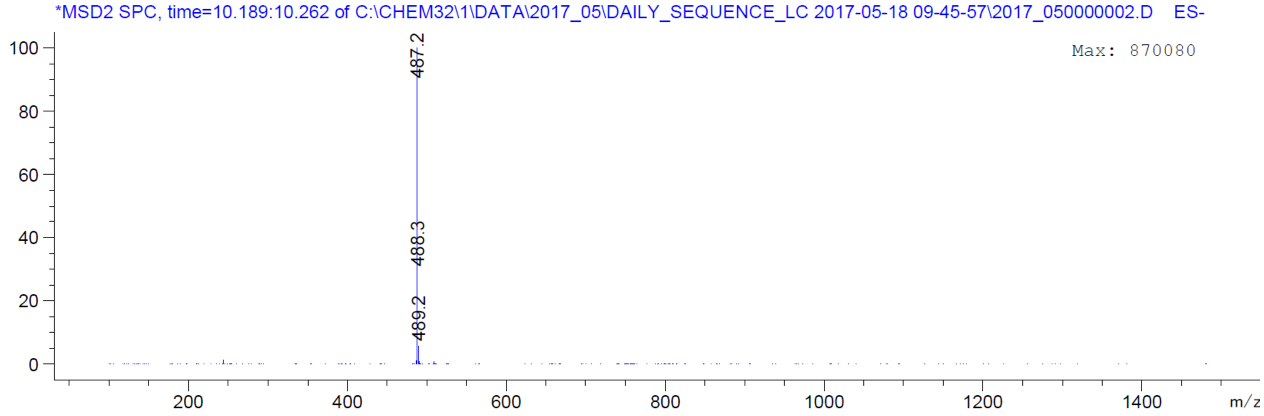

*MSD2 SPC, time=10.189:10.262 of C:\CHEM32\1\DATA\2017_05\DAILY_SEQUENCE_LC 2017-05-18 09-45-57\2017_050000002.D ES-
Max: 870080
100
80
60
40
20
0
200
400
600
800
1000
1200
1400
m/z
487.2
488.3
489.2

Origin	Bruker BioSpin GmbH
Solvent	CDCl3
Temperature	295.5
Pulse Sequence	zg30
Experiment	1D
Number of Scans	16
Acquisition Date	2017-10-13T12:19:00
Spectrometer Frequency	400.13
Spectral Width	8012.8
Lowest Frequency	-1544.9
Nucleus	1H
Acquired Size	32768
Spectral Size	65536

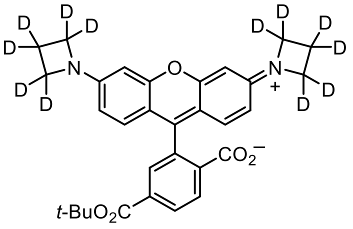

S2

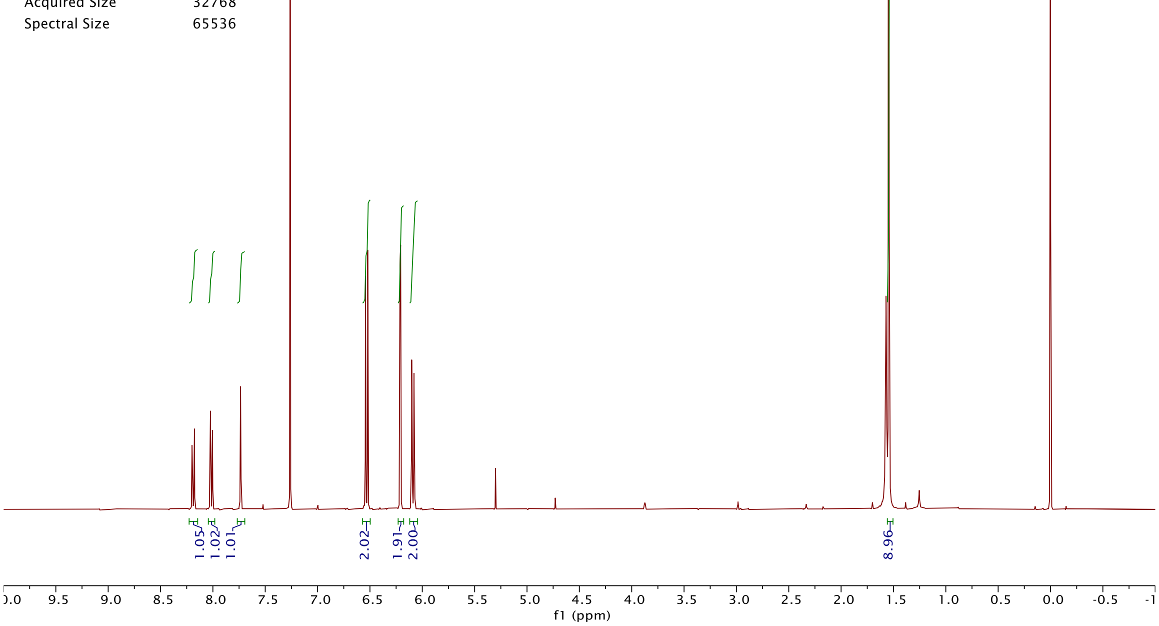

Origin	Bruker BioSpin GmbH
Solvent	CDCl3
Temperature	300.0
Pulse Sequence	zgpg30
Experiment	1D
Number of Scans	512
Acquisition Date	2019-05-15T01:58:00
Spectrometer Frequency	100.62
Spectral Width	24038.5
Lowest Frequency	-1946.1
Nucleus	13C
Acquired Size	32768
Spectral Size	65536

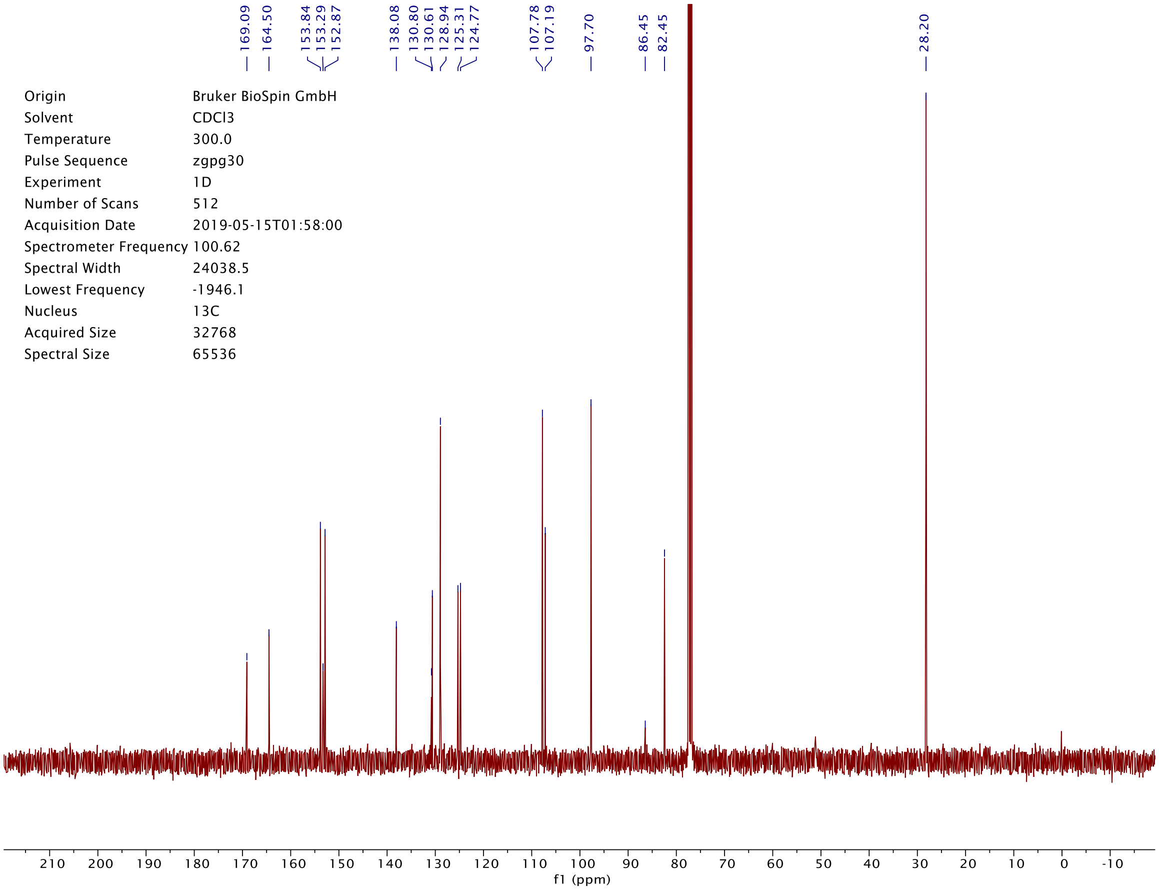

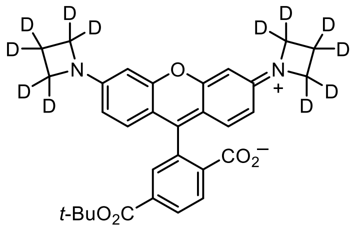

D
N
O
CO2
t-BuO2C

S2

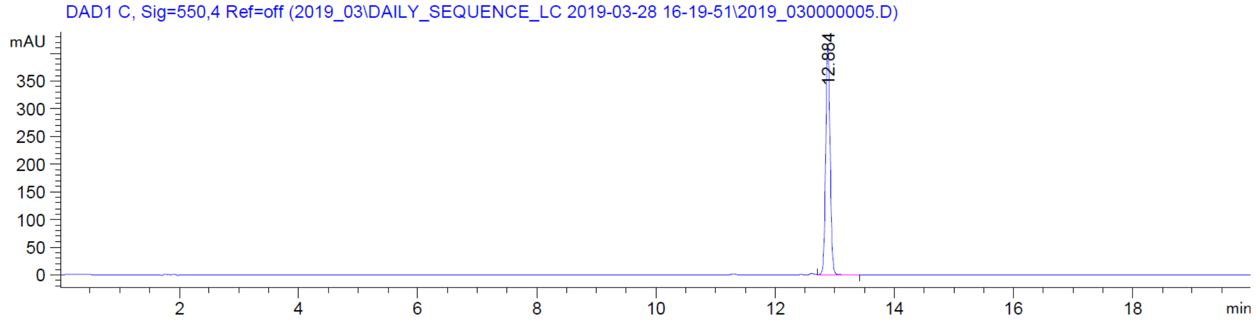

DAD1 C, Sig=550,4 Ref=off (2019_03\DAILY_SEQUENCE_LC 2019-03-28 16-19-51\2019_030000005.D)
mAU
12.884
min

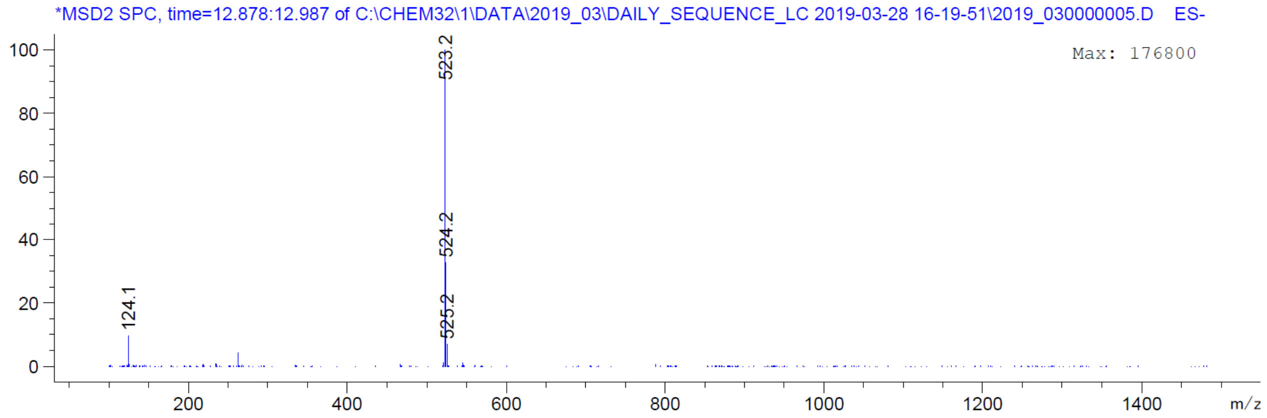

*MSD2 SPC, time=12.878:12.987 of C:\CHEM32\1\DATA\2019_03\DAILY_SEQUENCE_LC 2019-03-28 16-19-51\2019_030000005.D ES-
Max: 176800
523.2
524.2
525.2
124.1
m/z

Origin	Bruker BioSpin GmbH
Solvent	MeOD
Temperature	300.0
Pulse Sequence	zg30
Experiment	1D
Number of Scans	16
Acquisition Date	2017-04-20T09:40:00
Spectrometer Frequency	400.13
Spectral Width	8012.8
Lowest Frequency	-1543.2
Nucleus	1H
Acquired Size	32768
Spectral Size	65536

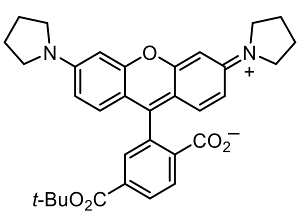

S3

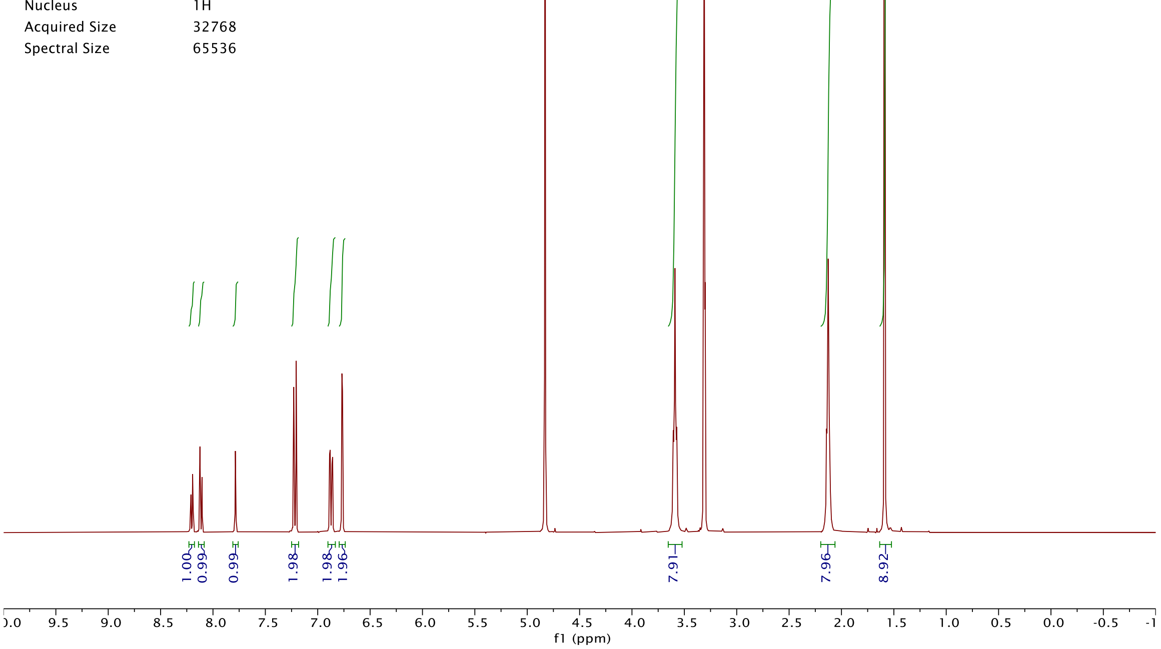

Origin	Bruker BioSpin GmbH
Solvent	MeOD
Temperature	300.0
Pulse Sequence	zgpg30
Experiment	1D
Number of Scans	4096
Acquisition Date	2019-03-26T23:55:00
Spectrometer Frequency	100.62
Spectral Width	24038.5
Lowest Frequency	-1817.9
Nucleus	13C
Acquired Size	32768
Spectral Size	65536

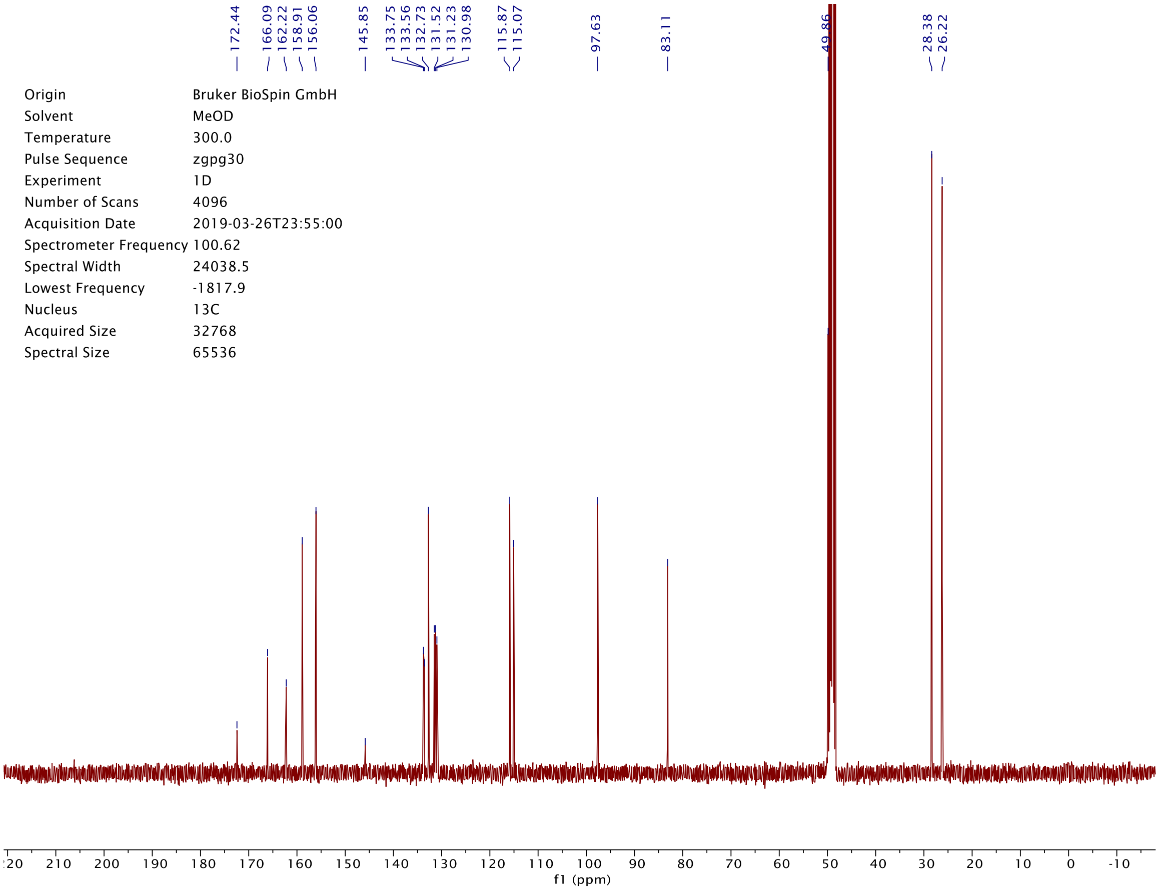

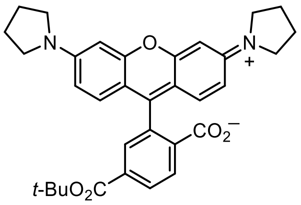
N
O
N
+
CO_2^-
t-BuO2C

S3

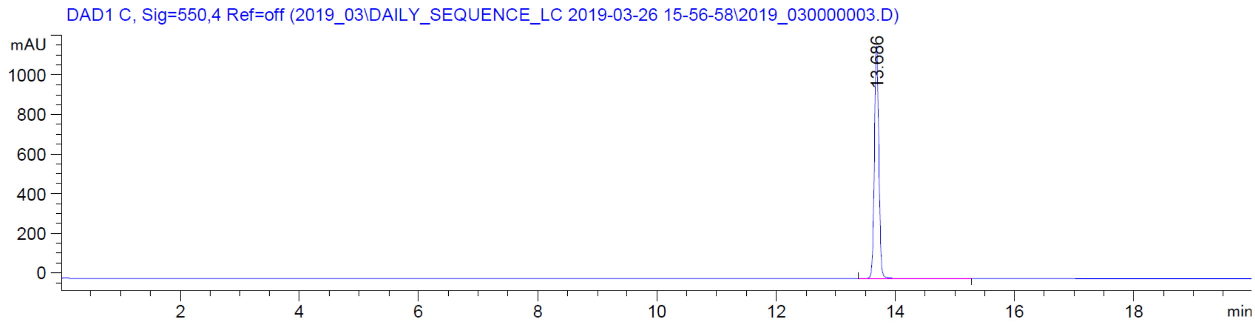
DAD1 C, Sig=550,4 Ref=off (2019_03\DAILY_SEQUENCE_LC 2019-03-26 15-56-58\2019_030000003.D)
mAU
1000
800
600
400
200
0
13.686
2
4
6
8
10
12
14
16
18
min

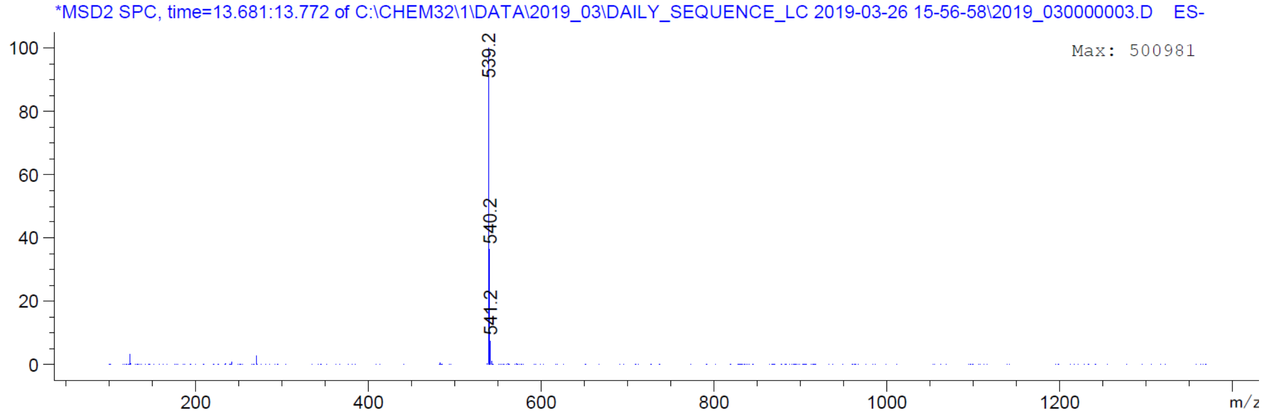
*MSD2 SPC, time=13.681:13.772 of C:\CHEM32\1\DATA\2019_03\DAILY_SEQUENCE_LC 2019-03-26 15-56-58\2019_030000003.D ES-
Max: 500981
100
80
60
40
20
0
539.2
540.2
541.2
200
400
600
800
1000
1200
m/z

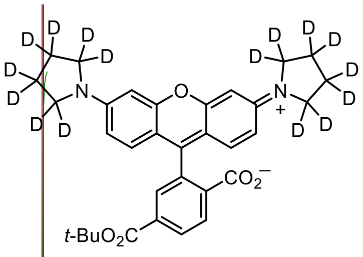

S4

Origin	Bruker BioSpin GmbH
Solvent	MeOD
Temperature	300.0
Pulse Sequence	zg30
Experiment	1D
Number of Scans	16
Acquisition Date	2017-04-20T09:45:00
Spectrometer Frequency	400.13
Spectral Width	8012.8
Lowest Frequency	-1543.2
Nucleus	1H
Acquired Size	32768
Spectral Size	65536

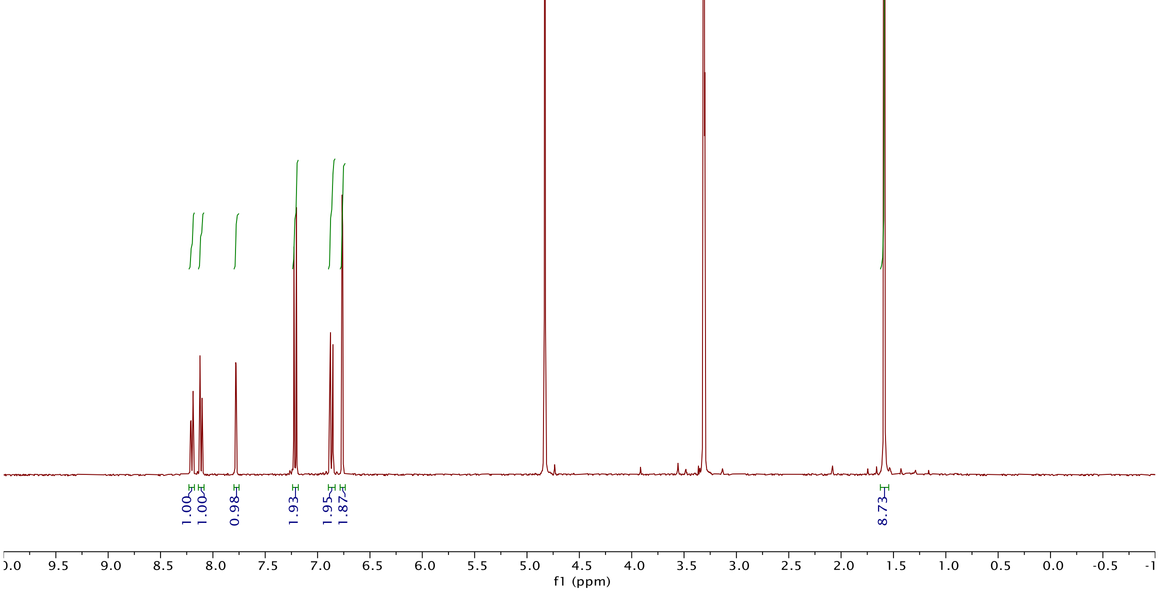

Origin	Bruker BioSpin GmbH
Solvent	MeOD
Temperature	300.0
Pulse Sequence	zgpg30
Experiment	1D
Number of Scans	4096
Acquisition Date	2019-05-15T00:11:00
Spectrometer Frequency	100.62
Spectral Width	24038.5
Lowest Frequency	-1817.7
Nucleus	13C
Acquired Size	32768
Spectral Size	65536

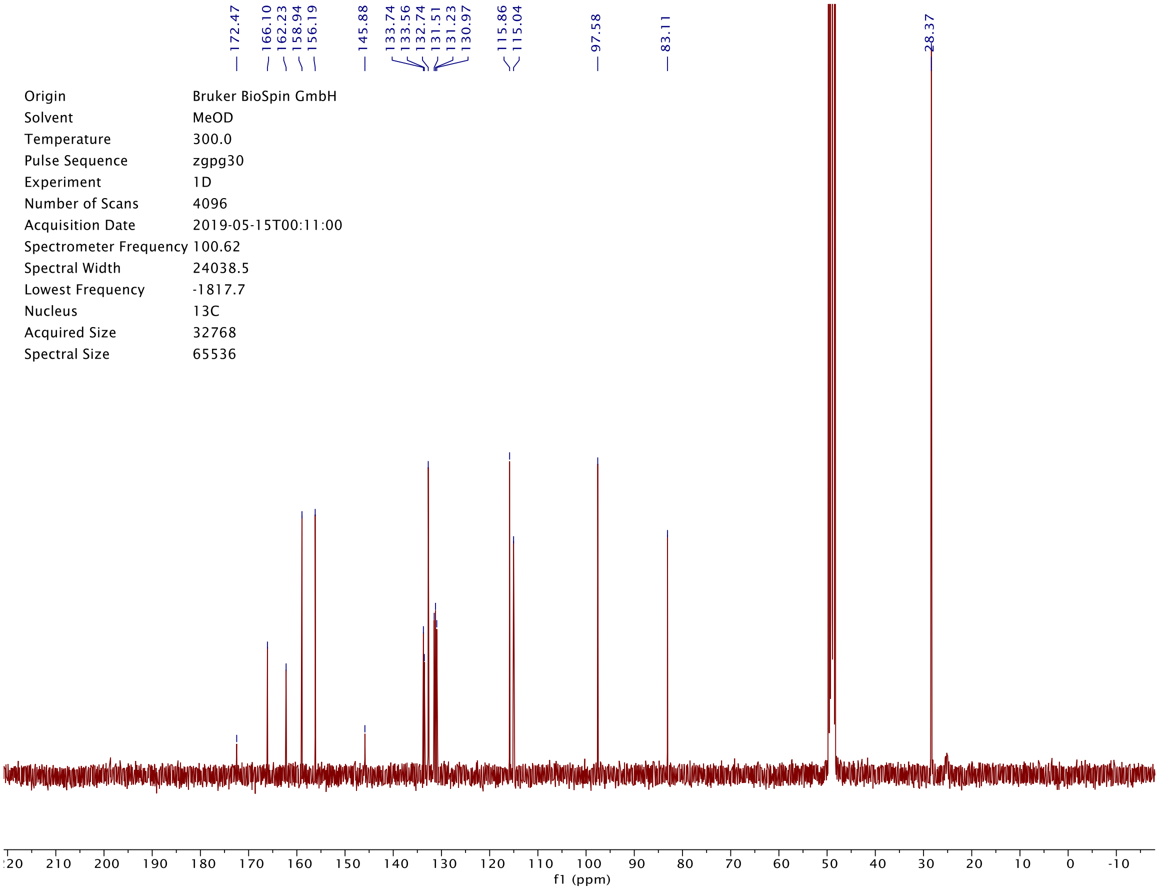

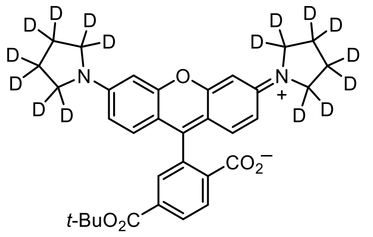

S4

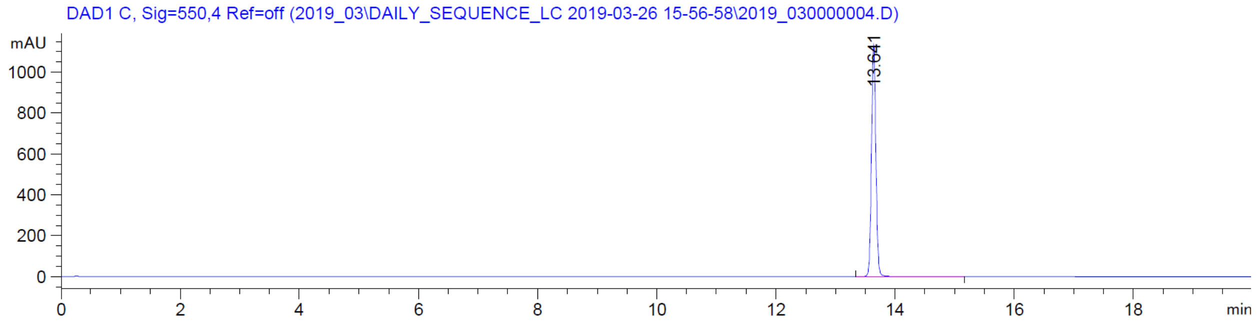

DAD1 C, Sig=550,4 Ref=off (2019_03\DAILY_SEQUENCE_LC 2019-03-26 15-56-58\2019_030000004.D)
mAU
13.641
min

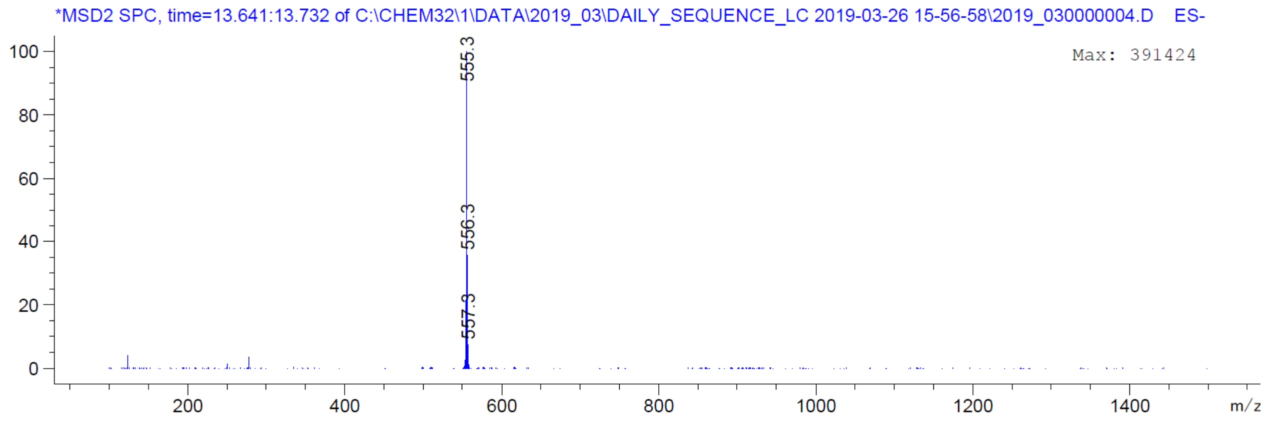

*MSD2 SPC, time=13.641:13.732 of C:\CHEM32\1\DATA\2019_03\DAILY_SEQUENCE_LC 2019-03-26 15-56-58\2019_030000004.D ES-
Max: 391424
555.3
556.3
557.3
m/z

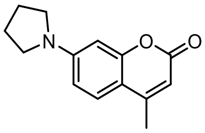

22

Origin	Bruker BioSpin GmbH
Solvent	CDCl3
Temperature	300.0
Pulse Sequence	zg30
Experiment	1D
Number of Scans	16
Acquisition Date	2019-04-05T10:14:00
Spectrometer Frequency	400.13
Spectral Width	8012.8
Lowest Frequency	-1545.3
Nucleus	1H
Acquired Size	32768
Spectral Size	65536

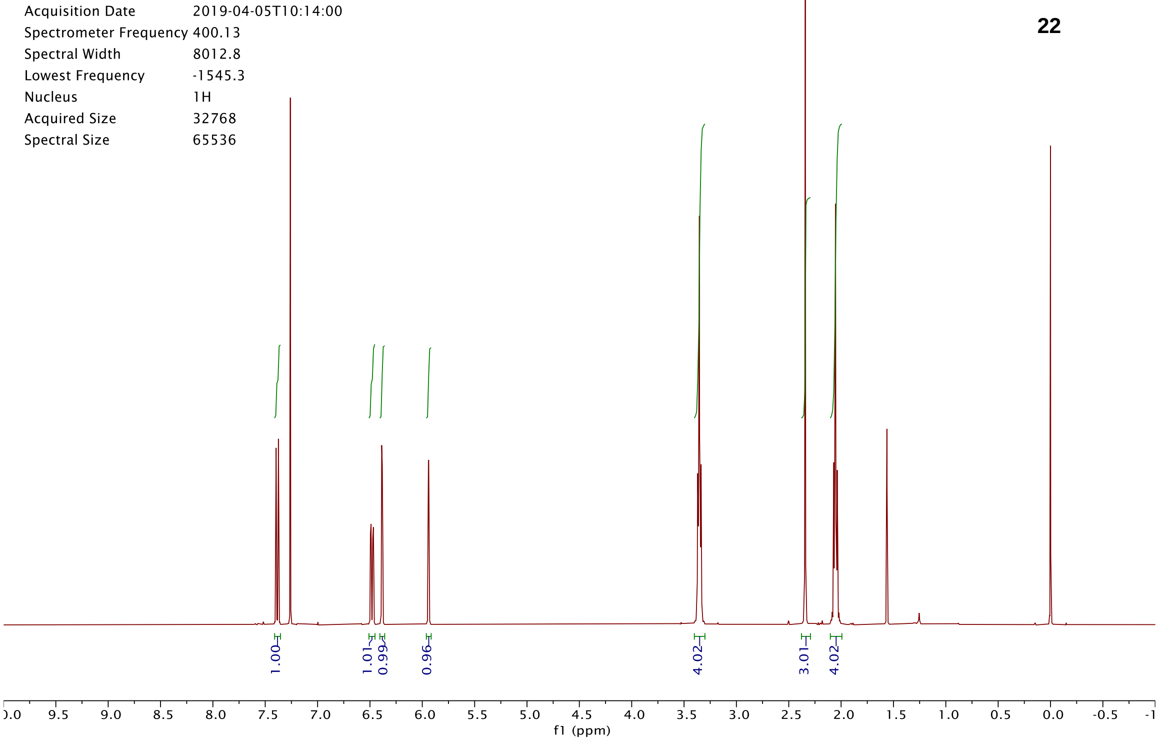

Origin	Bruker BioSpin GmbH
Solvent	CDCl3
Temperature	300.0
Pulse Sequence	zgpg30
Experiment	1D
Number of Scans	2048
Acquisition Date	2019-04-05T21:08:00
Spectrometer Frequency	100.62
Spectral Width	24038.5
Lowest Frequency	-1947.5
Nucleus	13C
Acquired Size	32768
Spectral Size	65536

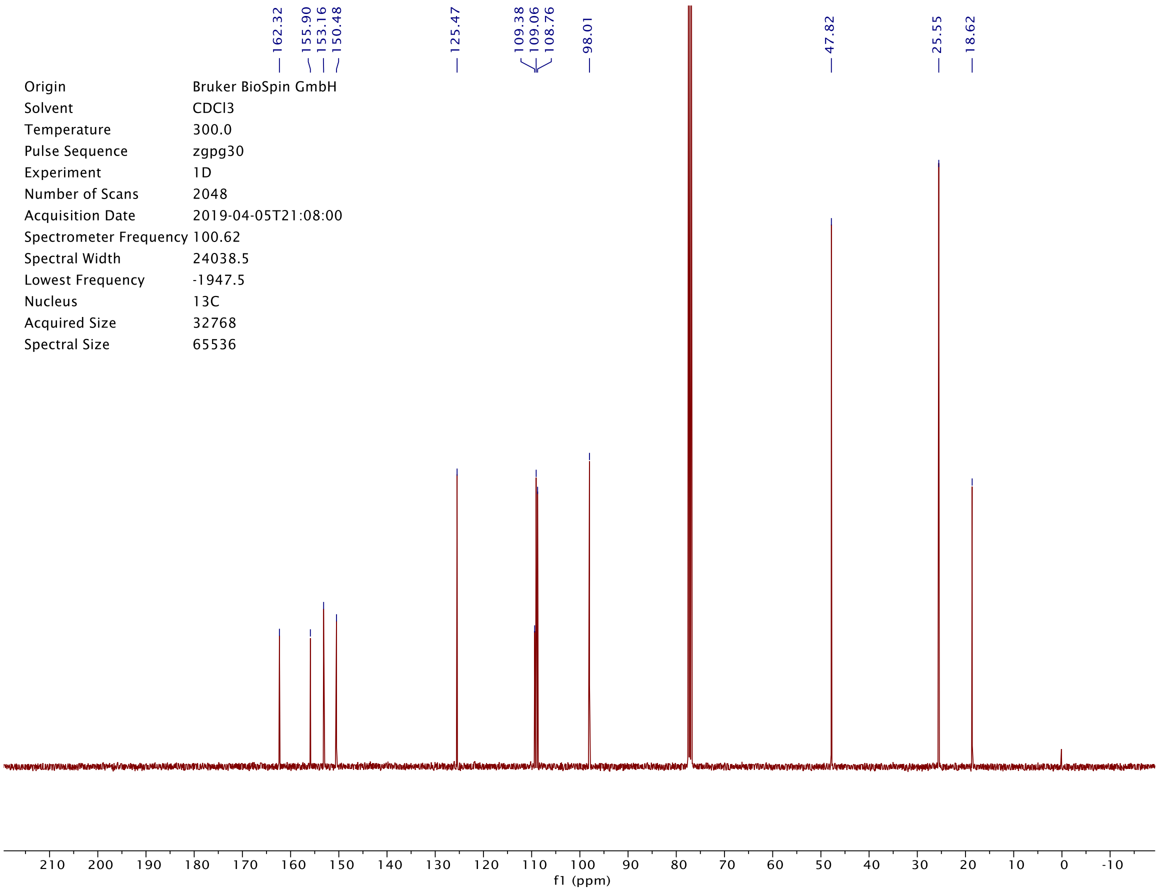

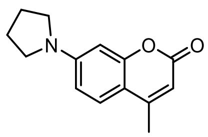

22

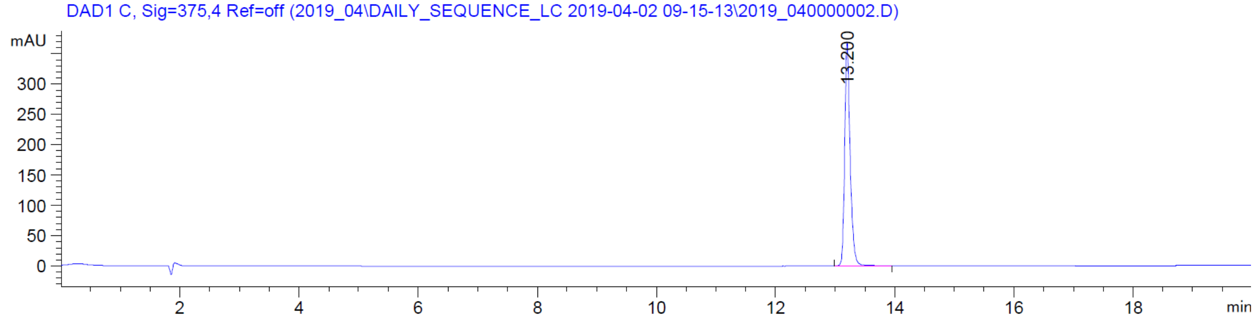

DAD1 C, Sig=375,4 Ref=off (2019_04\DAILY_SEQUENCE_LC 2019-04-02 09-15-13\2019_040000002.D)
mAU
13.200
min

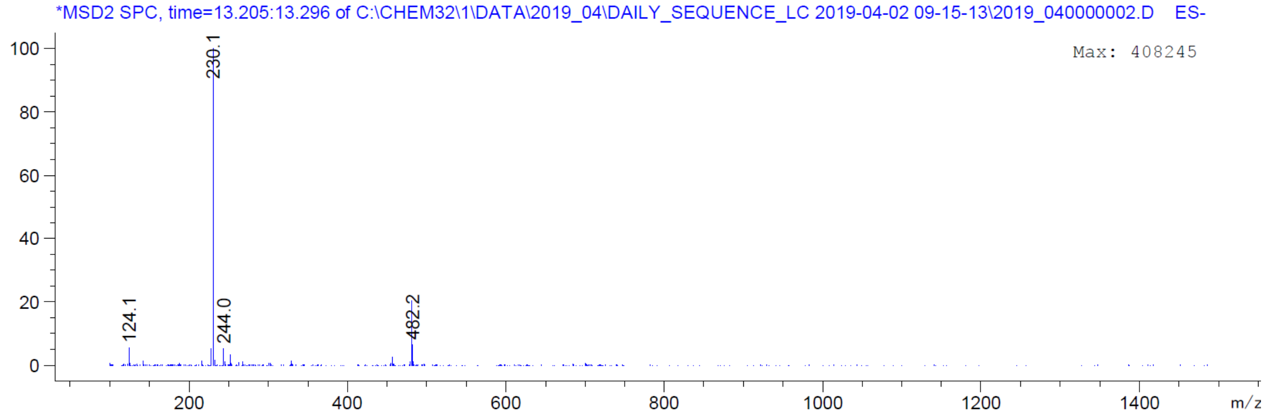

*MSD2 SPC, time=13.205:13.296 of C:\CHEM32\1\DATA\2019_04\DAILY_SEQUENCE_LC 2019-04-02 09-15-13\2019_040000002.D ES-
Max: 408245
230.1
124.1
244.0
482.2
m/z

Origin	Bruker BioSpin GmbH
Solvent	CDCl3
Temperature	300.0
Pulse Sequence	zg30
Experiment	1D
Number of Scans	16
Acquisition Date	2019-04-05T10:19:00
Spectrometer Frequency	400.13
Spectral Width	8012.8
Lowest Frequency	-1545.2
Nucleus	1H
Acquired Size	32768
Spectral Size	65536

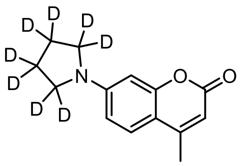

$\mathbf{22_D}$

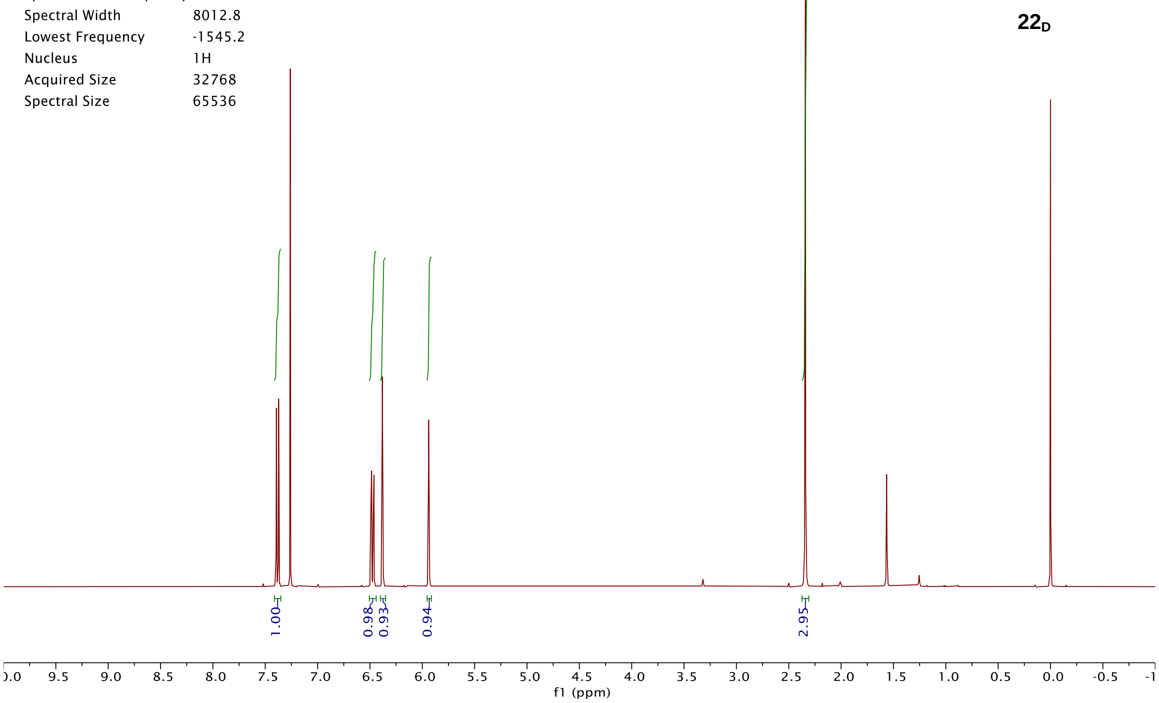

Origin	Bruker BioSpin GmbH
Solvent	CDCl3
Temperature	300.0
Pulse Sequence	zgpg30
Experiment	1D
Number of Scans	256
Acquisition Date	2019-05-10T14:56:00
Spectrometer Frequency	100.62
Spectral Width	24038.5
Lowest Frequency	-1947.2
Nucleus	13C
Acquired Size	32768
Spectral Size	65536

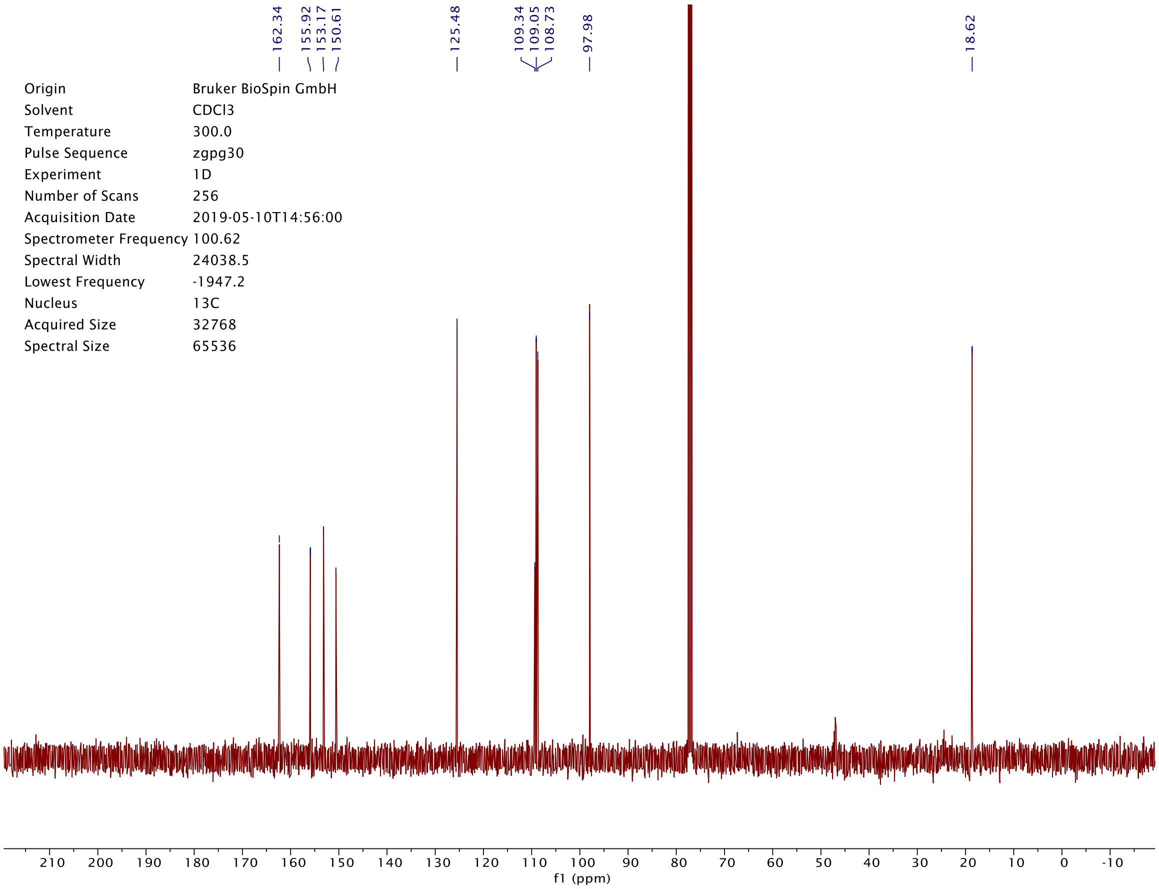

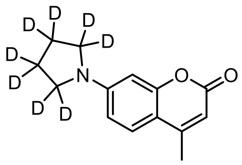
D
D
D
D
D
D
D
D
N
O
O

22$_D$

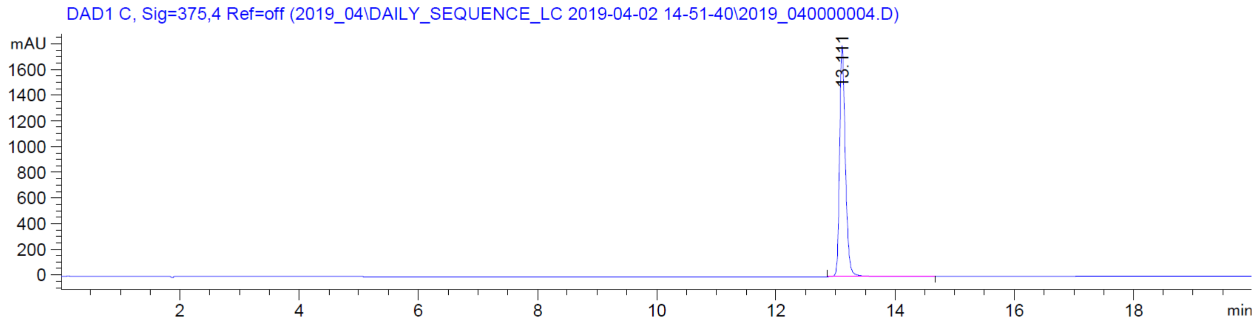
DAD1 C, Sig=375,4 Ref=off (2019_04\DAILY_SEQUENCE_LC 2019-04-02 14-51-40\2019_040000004.D)
mAU
13.111
min

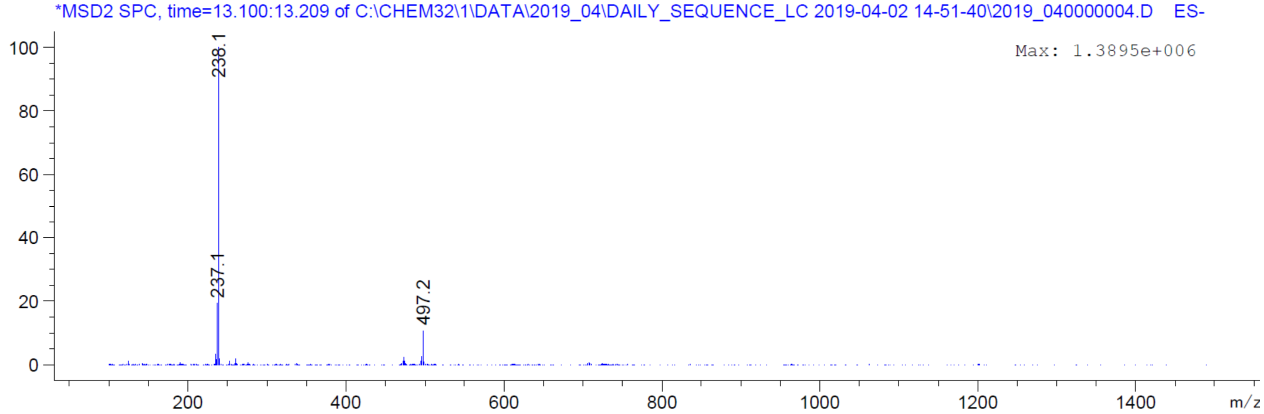
*MSD2 SPC, time=13.100:13.209 of C:\CHEM32\1\DATA\2019_04\DAILY_SEQUENCE_LC 2019-04-02 14-51-40\2019_040000004.D ES-
Max: 1.3895e+006
238.1
237.1
497.2
m/z

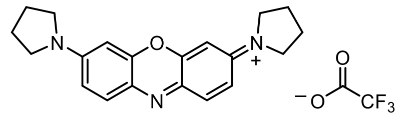

23

Origin	Bruker BioSpin GmbH
Solvent	MeOD
Temperature	300.0
Pulse Sequence	zg30
Experiment	1D
Number of Scans	16
Acquisition Date	2019-04-08T11:32:00
Spectrometer Frequency	400.13
Spectral Width	8012.8
Lowest Frequency	-1543.2
Nucleus	1H
Acquired Size	32768
Spectral Size	65536

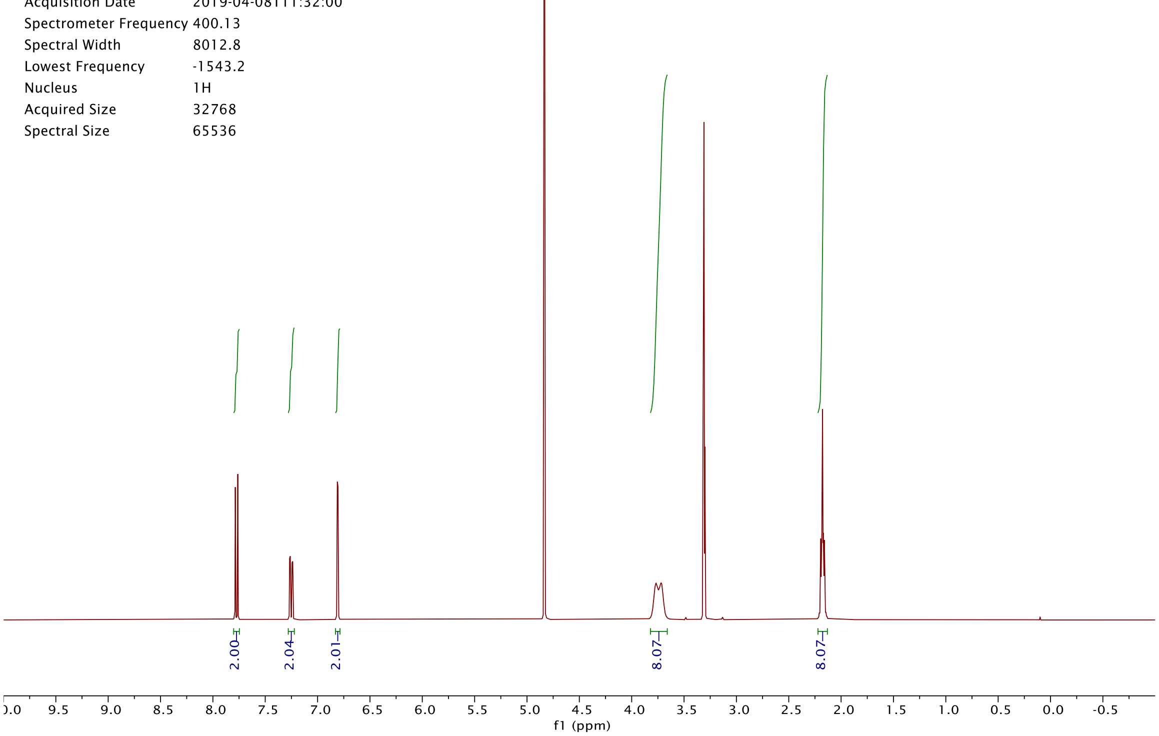

Origin	Bruker BioSpin GmbH
Solvent	MeOD
Temperature	300.0
Pulse Sequence	zgpg30
Experiment	1D
Number of Scans	2048
Acquisition Date	2019-04-09T21:08:00
Spectrometer Frequency	100.62
Spectral Width	24038.5
Lowest Frequency	-1818.0
Nucleus	13C
Acquired Size	32768
Spectral Size	65536

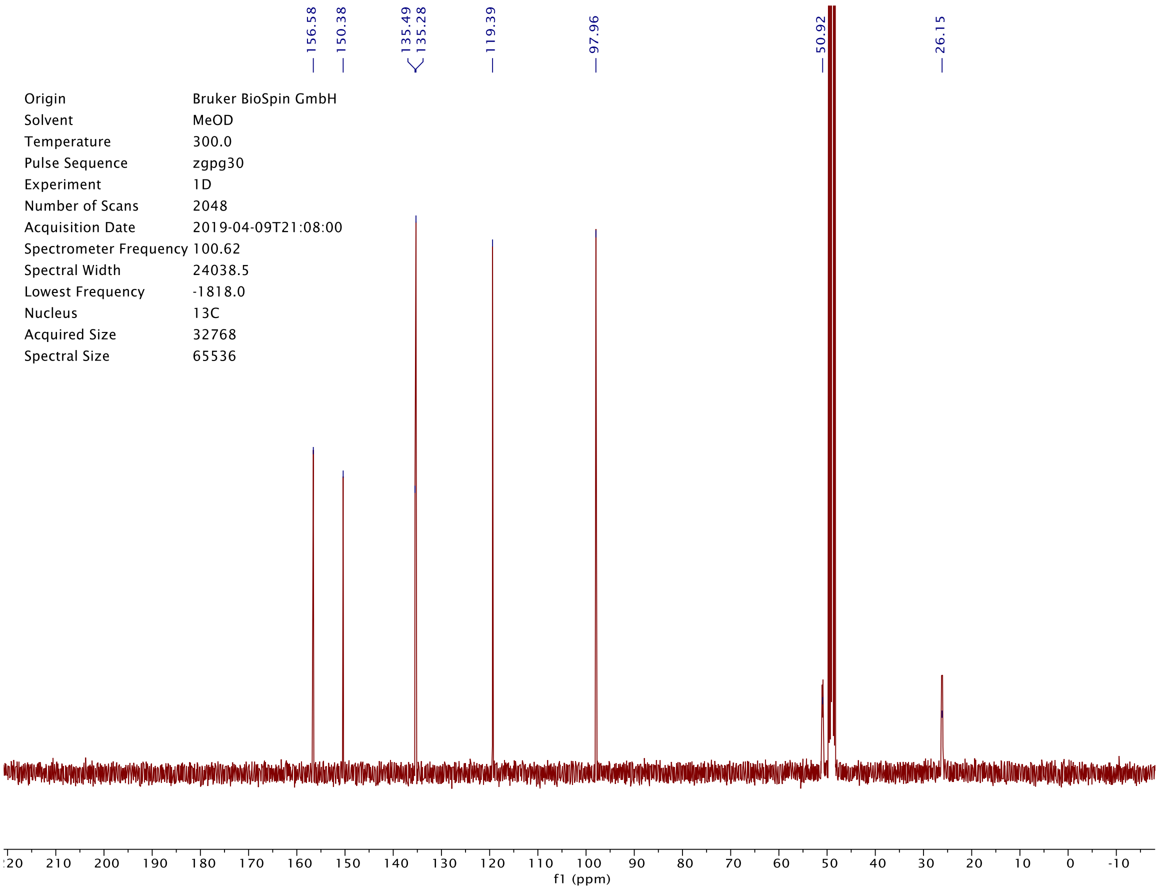

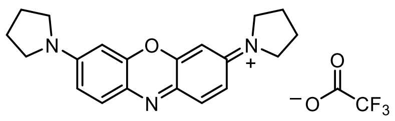

23

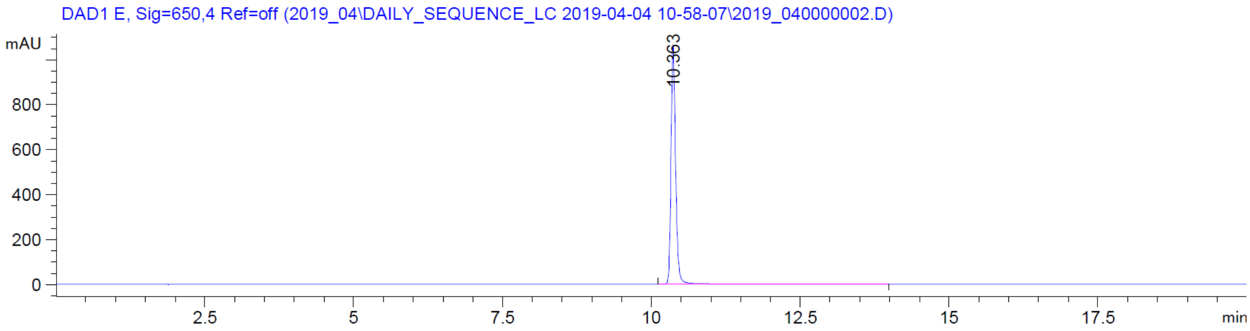

DAD1 E, Sig=650,4 Ref=off (2019_04\DAILY_SEQUENCE_LC 2019-04-04 10-58-07\2019_040000002.D)
mAU
10.363
min

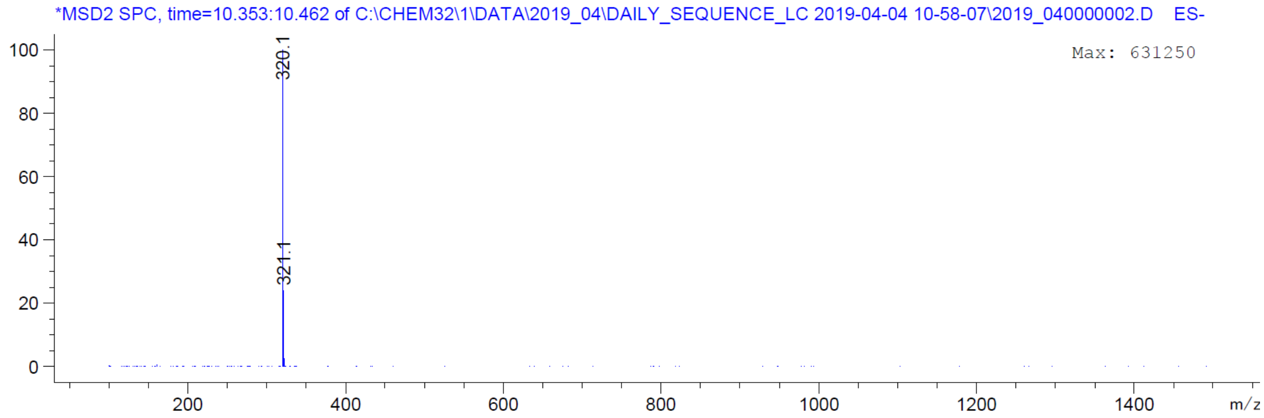

*MSD2 SPC, time=10.353:10.462 of C:\CHEM32\1\DATA\2019_04\DAILY_SEQUENCE_LC 2019-04-04 10-58-07\2019_040000002.D ES-
Max: 631250
320.1
321.1
m/z

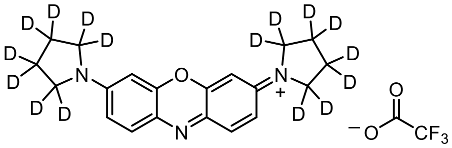

D D D D D D D D
N O N N
+
O
−O CF3

$\mathbf{23_D}$

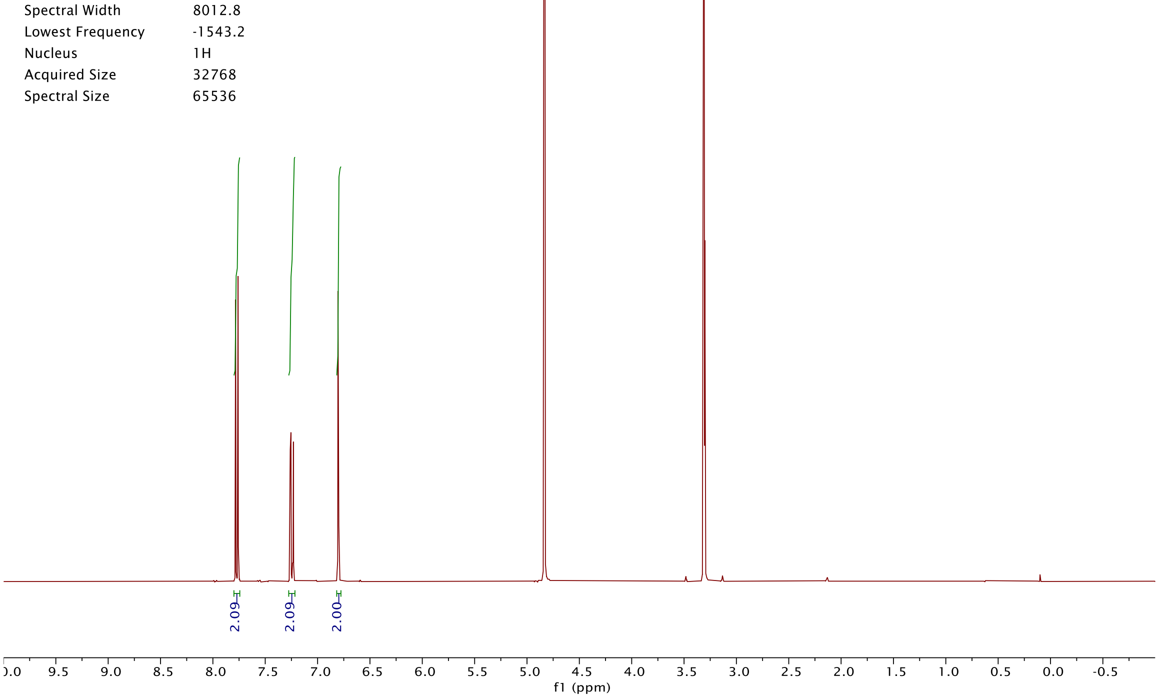

Origin Bruker BioSpin GmbH
Solvent MeOD
Temperature 300.0
Pulse Sequence zg30
Experiment 1D
Number of Scans 16
Acquisition Date 2019-04-08T11:40:00
Spectrometer Frequency 400.13
Spectral Width 8012.8
Lowest Frequency -1543.2
Nucleus 1H
Acquired Size 32768
Spectral Size 65536
2.09
2.09
2.00
f1 (ppm)

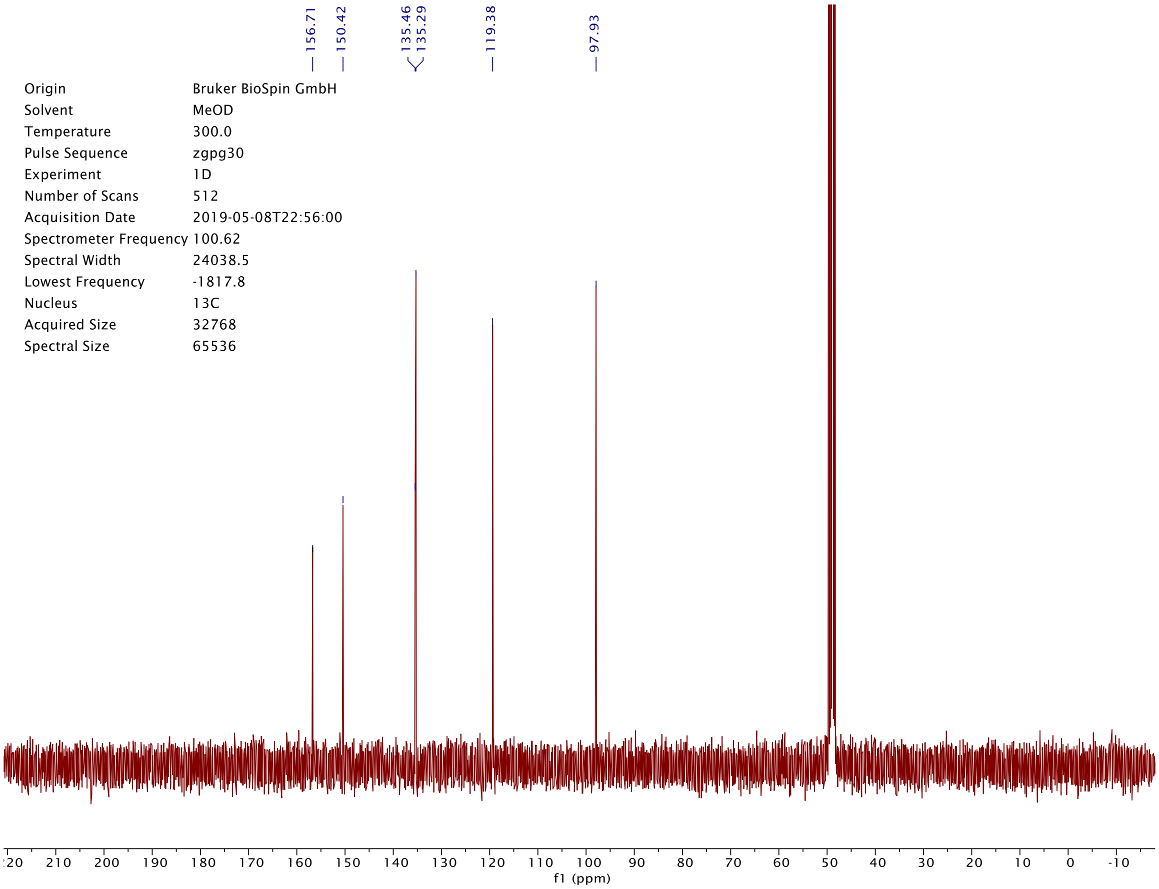

156.71
150.42
135.46
135.29
119.38
97.93
Origin Bruker BioSpin GmbH
Solvent MeOD
Temperature 300.0
Pulse Sequence zgpg30
Experiment 1D
Number of Scans 512
Acquisition Date 2019-05-08T22:56:00
Spectrometer Frequency 100.62
Spectral Width 24038.5
Lowest Frequency -1817.8
Nucleus 13C
Acquired Size 32768
Spectral Size 65536
f1 (ppm)

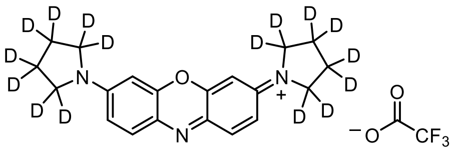

23_D

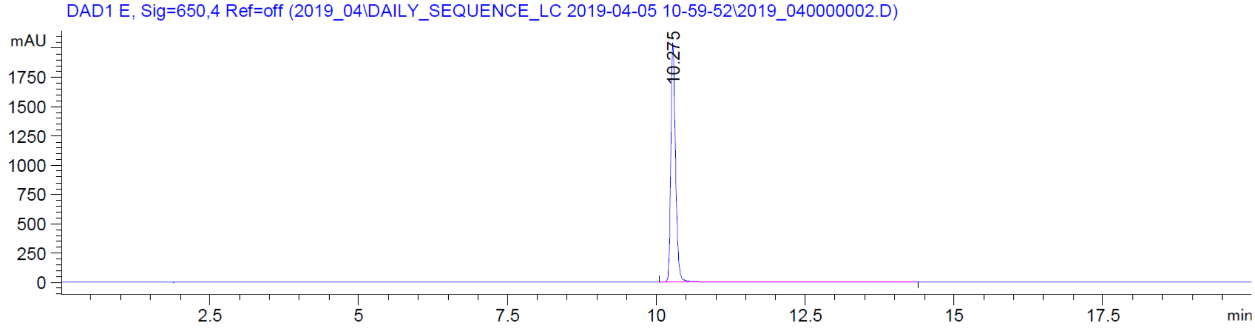

DAD1 E, Sig=650,4 Ref=off (2019_04\DAILY_SEQUENCE_LC 2019-04-05 10-59-52\2019_040000002.D)
mAU
10.275
min

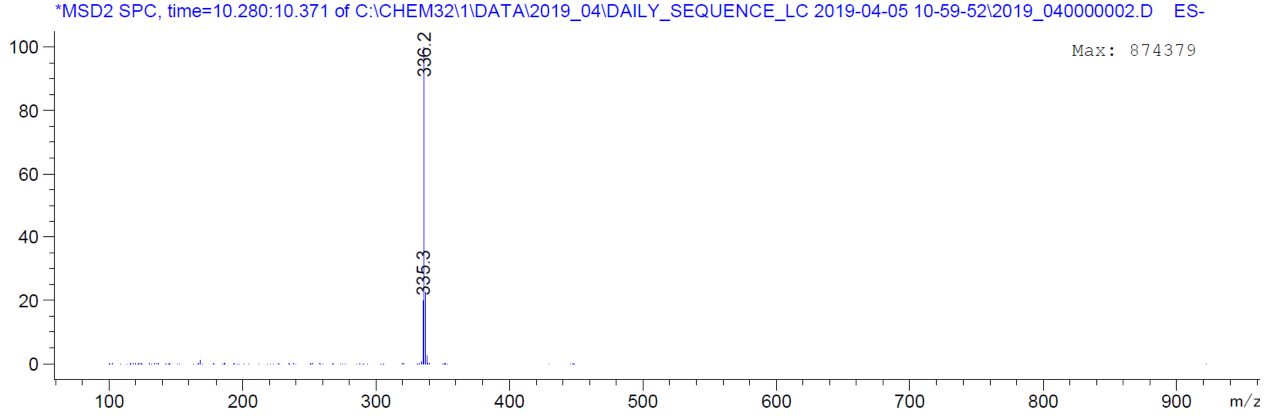

*MSD2 SPC, time=10.280:10.371 of C:\CHEM32\1\DATA\2019_04\DAILY_SEQUENCE_LC 2019-04-05 10-59-52\2019_040000002.D ES-
Max: 874379
336.2
335.3
m/z

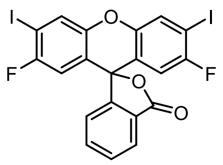
I
I
O
F
F
O
O

S12

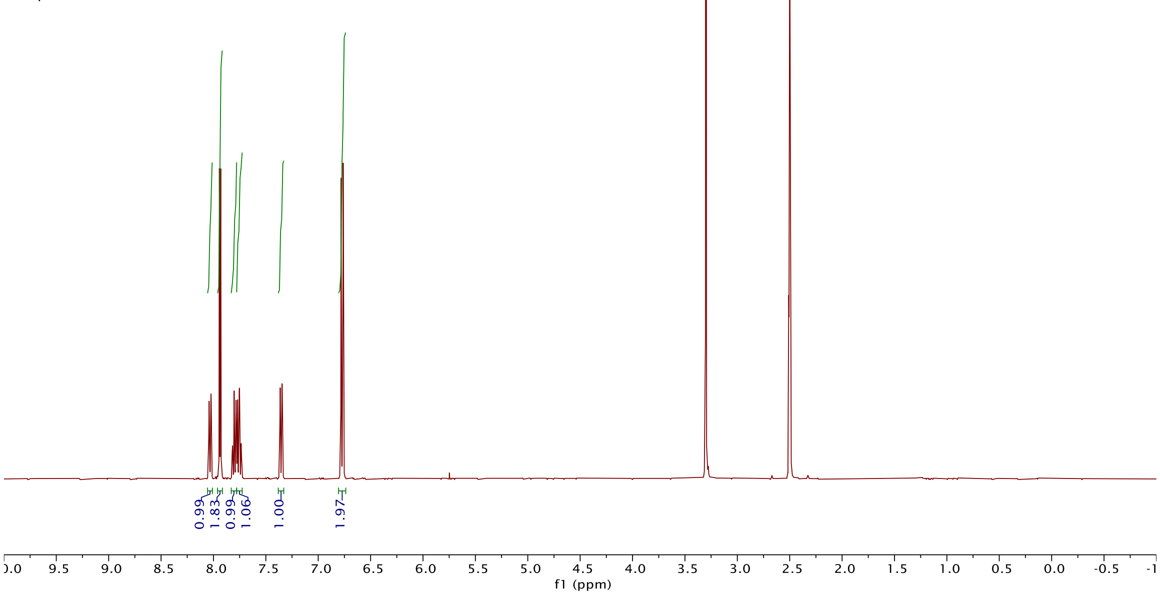
Origin Bruker BioSpin GmbH
Solvent DMSO
Temperature 300.0
Pulse Sequence zg30
Experiment 1D
Number of Scans 16
Acquisition Date 2020-08-04T15:24:00
Spectrometer Frequency 400.13
Spectral Width 8012.8
Lowest Frequency -1539.2
Nucleus 1H
Acquired Size 32768
Spectral Size 65536
0.99
1.83
0.99
1.06
1.00
1.97
9.5 9.0 8.5 8.0 7.5 7.0 6.5 6.0 5.5 5.0 4.5 4.0 3.5 3.0 2.5 2.0 1.5 1.0 0.5 0.0 -0.5
f1 (ppm)

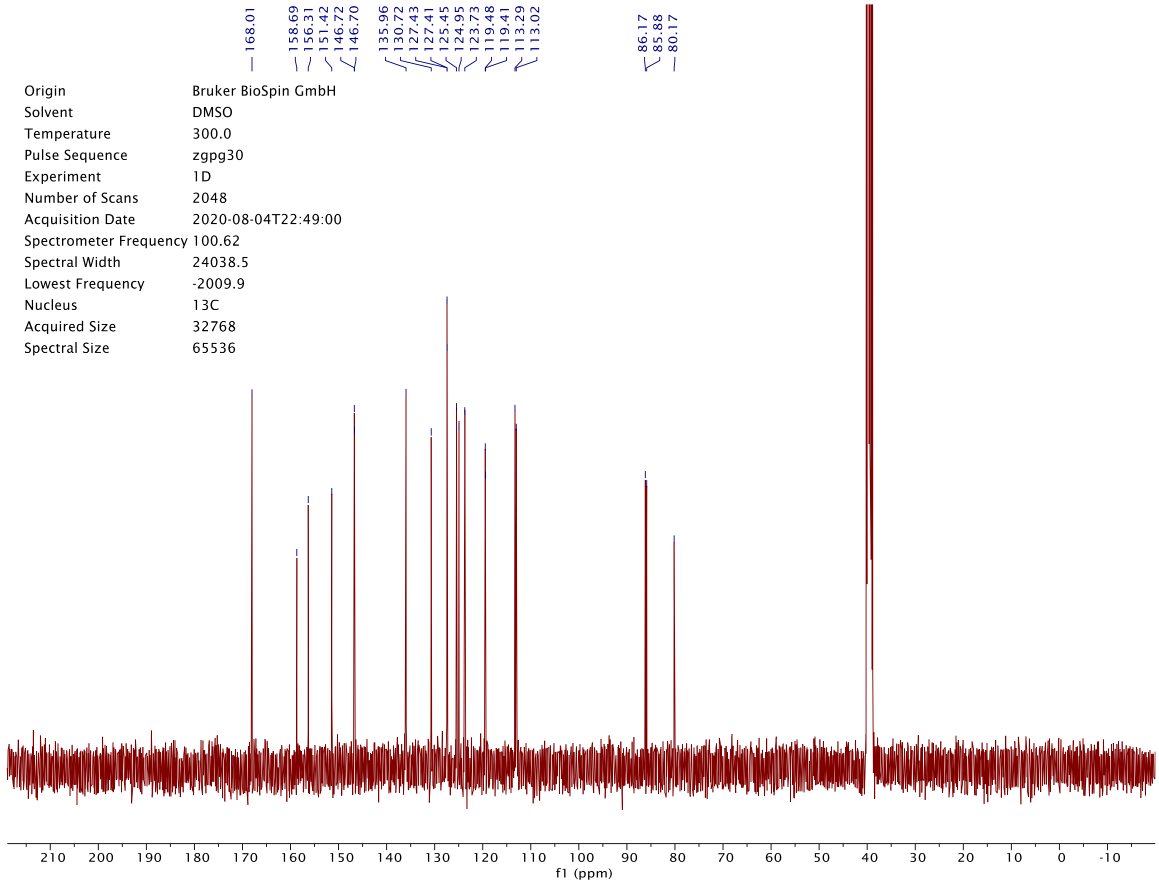
168.01
158.69
156.31
151.42
146.72
146.70
135.96
130.72
127.43
127.41
125.45
124.95
123.73
119.48
119.41
113.29
113.02
86.17
85.88
80.17
Origin Bruker BioSpin GmbH
Solvent DMSO
Temperature 300.0
Pulse Sequence zgpg30
Experiment 1D
Number of Scans 2048
Acquisition Date 2020-08-04T22:49:00
Spectrometer Frequency 100.62
Spectral Width 24038.5
Lowest Frequency -2009.9
Nucleus 13C
Acquired Size 32768
Spectral Size 65536
210 200 190 180 170 160 150 140 130 120 110 100 90 80 70 60 50 40 30 20 10 0 -10
f1 (ppm)

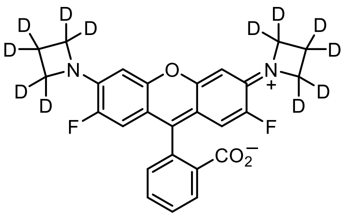

24_D

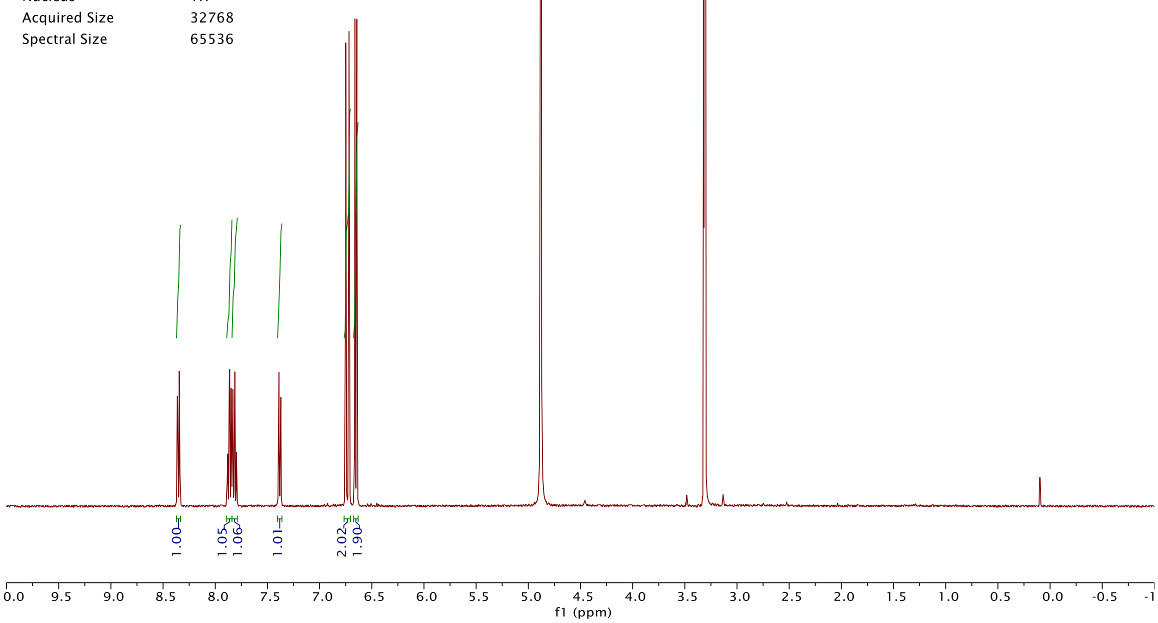

Origin	Bruker BioSpin GmbH
Solvent	MeOD
Temperature	295.3
Pulse Sequence	zg30
Experiment	1D
Number of Scans	16
Acquisition Date	2020-04-24T10:48:00
Spectrometer Frequency	400.13
Spectral Width	8012.8
Lowest Frequency	-1543.3
Nucleus	1H
Acquired Size	32768
Spectral Size	65536

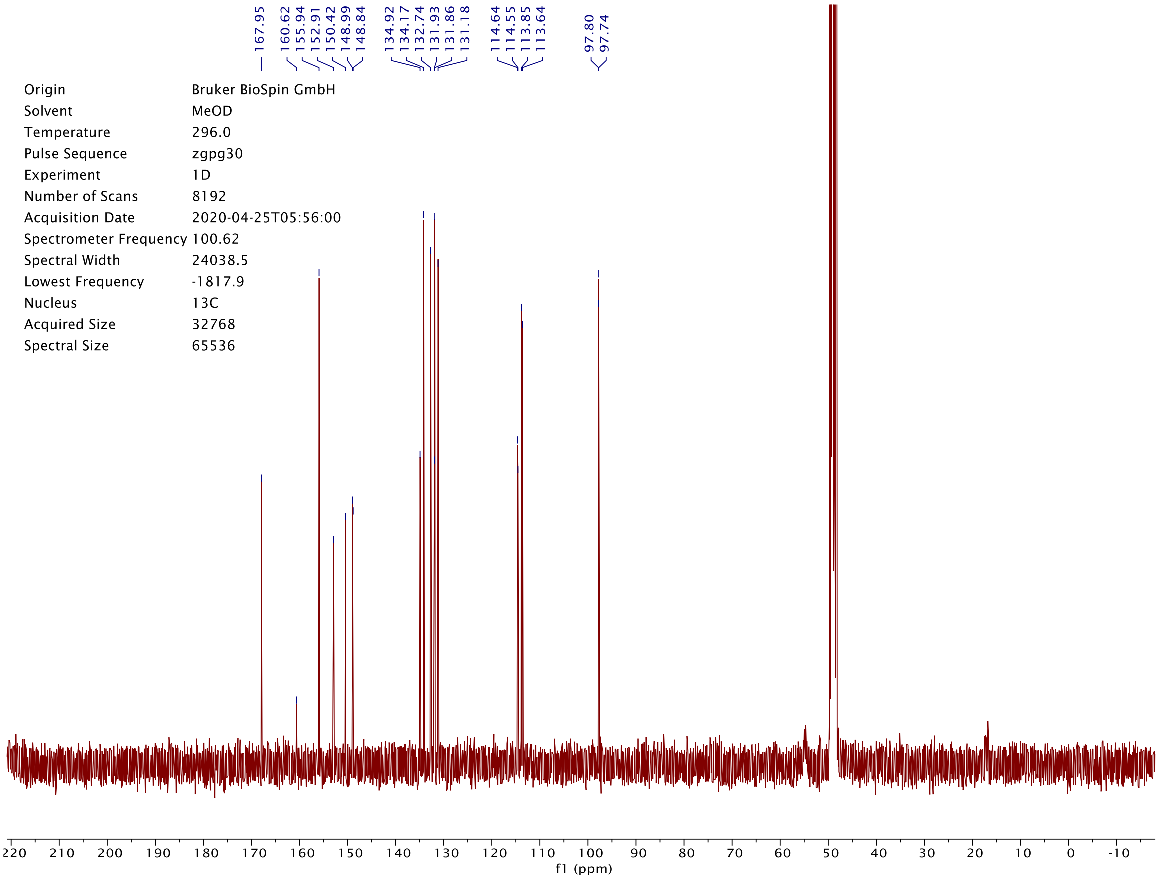

Origin	Bruker BioSpin GmbH
Solvent	MeOD
Temperature	296.0
Pulse Sequence	zgpg30
Experiment	1D
Number of Scans	8192
Acquisition Date	2020-04-25T05:56:00
Spectrometer Frequency	100.62
Spectral Width	24038.5
Lowest Frequency	-1817.9
Nucleus	13C
Acquired Size	32768
Spectral Size	65536

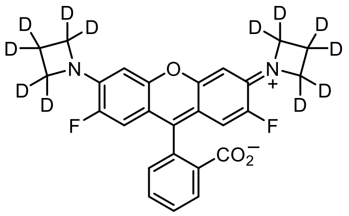

24_D

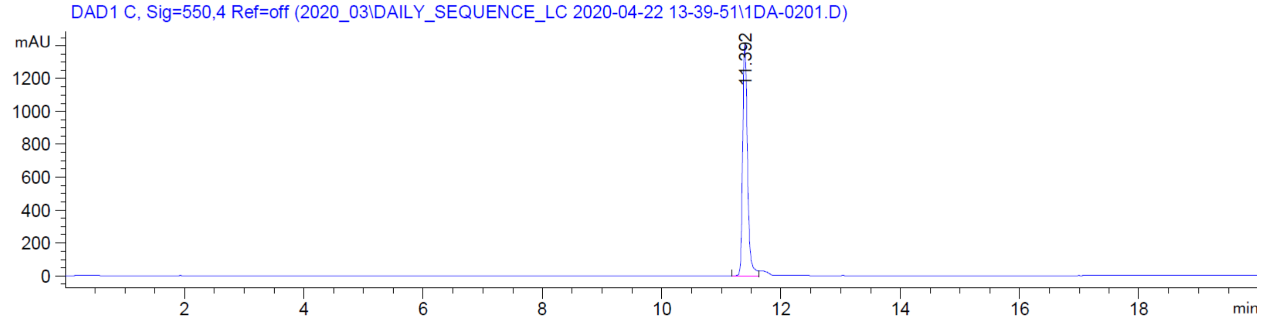
DAD1 C, Sig=550,4 Ref=off (2020_03\DAILY_SEQUENCE_LC 2020-04-22 13-39-51\1DA-0201.D)
mAU
1200
1000
800
600
400
200
0
11.392
2
4
6
8
10
12
14
16
18
min

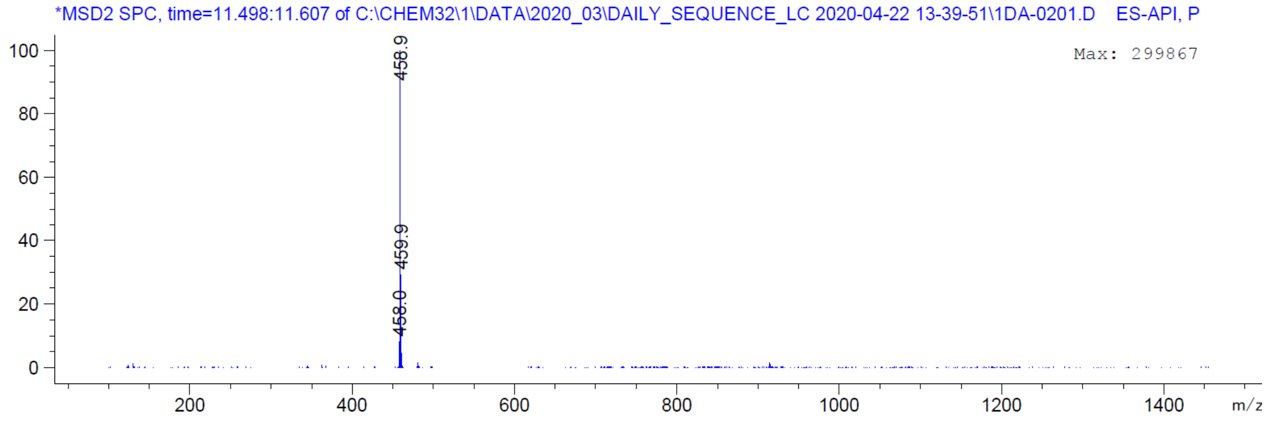
*MSD2 SPC, time=11.498:11.607 of C:\CHEM32\1\DATA\2020_03\DAILY_SEQUENCE_LC 2020-04-22 13-39-51\1DA-0201.D ES-API, P
Max: 299867
100
80
60
40
20
0
458.9
459.9
458.0
200
400
600
800
1000
1200
1400
m/z

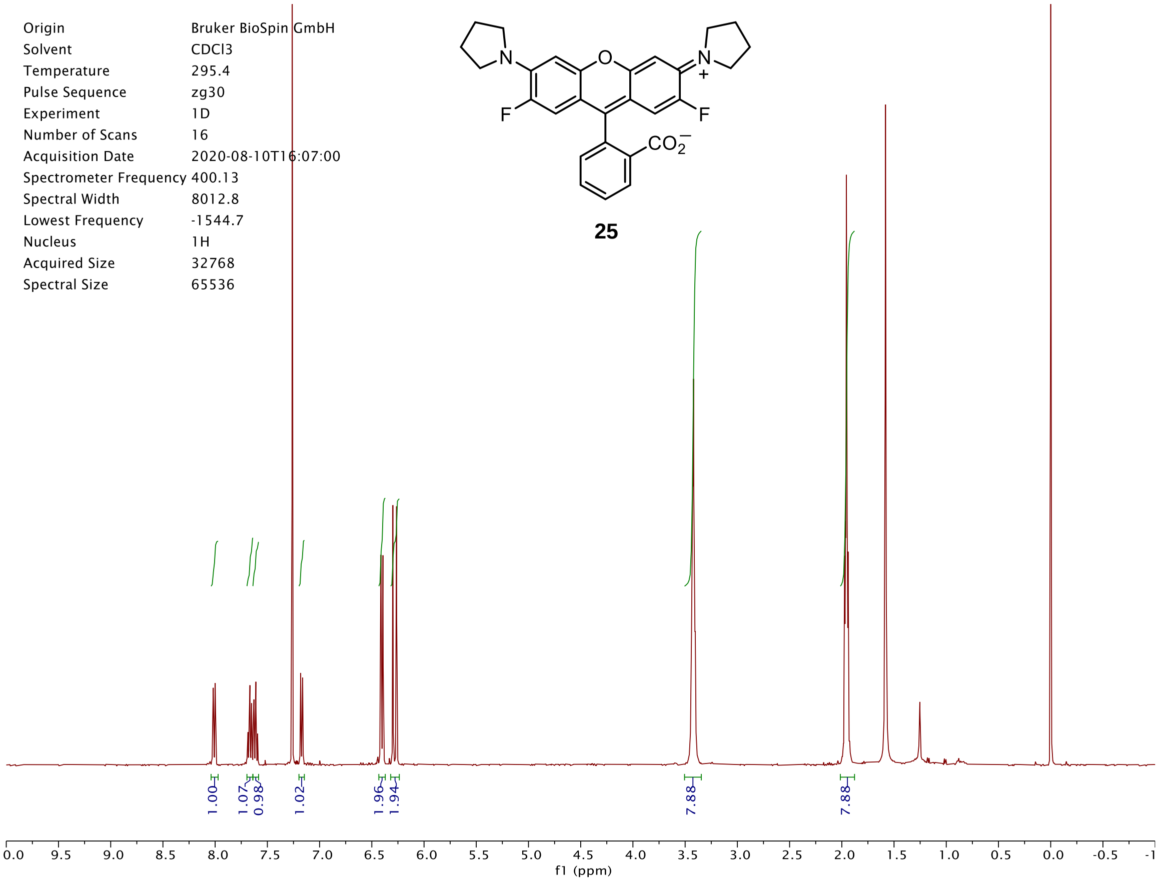

25
Origin Bruker BioSpin GmbH
Solvent CDCl3
Temperature 295.4
Pulse Sequence zg30
Experiment 1D
Number of Scans 16
Acquisition Date 2020-08-10T16:07:00
Spectrometer Frequency 400.13
Spectral Width 8012.8
Lowest Frequency -1544.7
Nucleus 1H
Acquired Size 32768
Spectral Size 65536
1.00
1.07
0.98
1.02
1.96
1.94
7.88
7.88
f1 (ppm)

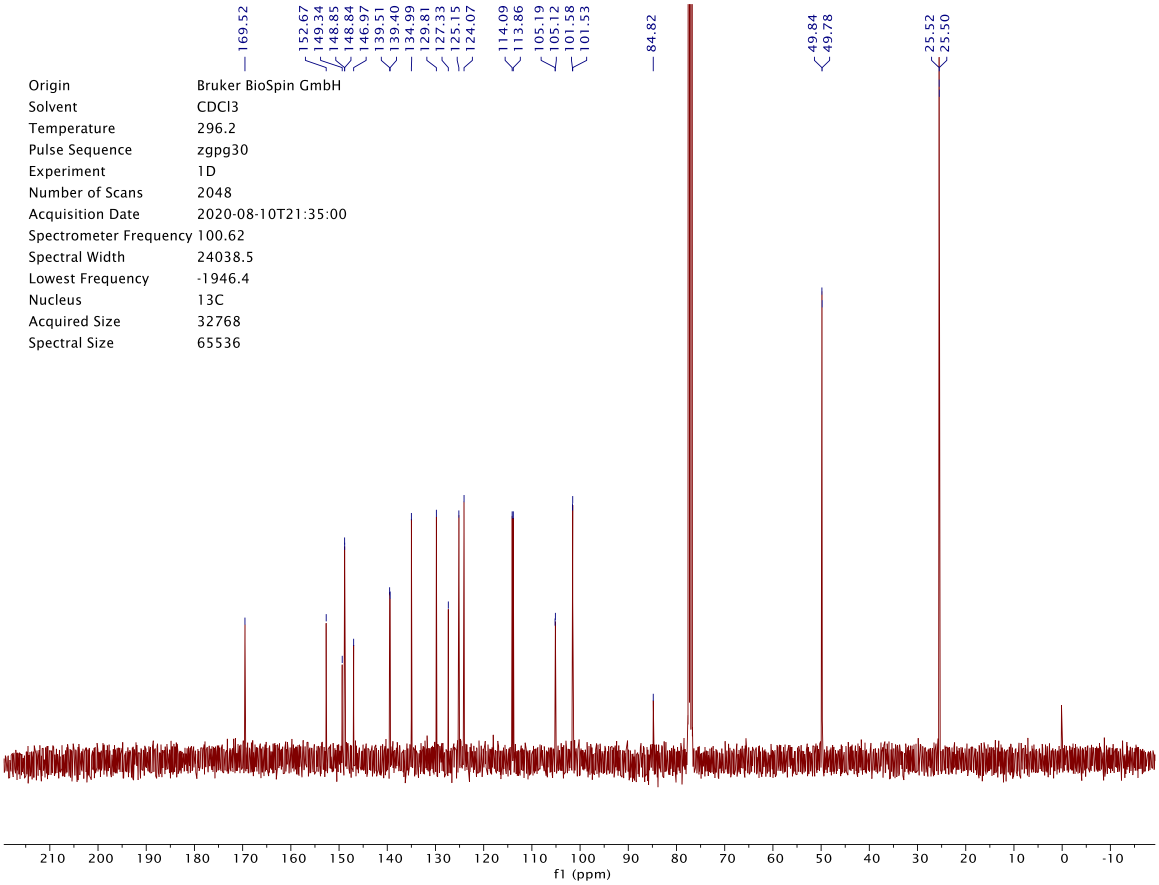

169.52
152.67
149.34
148.85
148.84
146.97
139.51
139.40
134.99
129.81
127.33
125.15
124.07
114.09
113.86
105.19
105.12
101.58
101.53
84.82
49.84
49.78
25.52
25.50
Origin Bruker BioSpin GmbH
Solvent CDCl3
Temperature 296.2
Pulse Sequence zgpg30
Experiment 1D
Number of Scans 2048
Acquisition Date 2020-08-10T21:35:00
Spectrometer Frequency 100.62
Spectral Width 24038.5
Lowest Frequency -1946.4
Nucleus 13C
Acquired Size 32768
Spectral Size 65536
f1 (ppm)

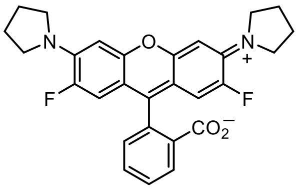
N
O
N
+
F
F
CO2
−

25

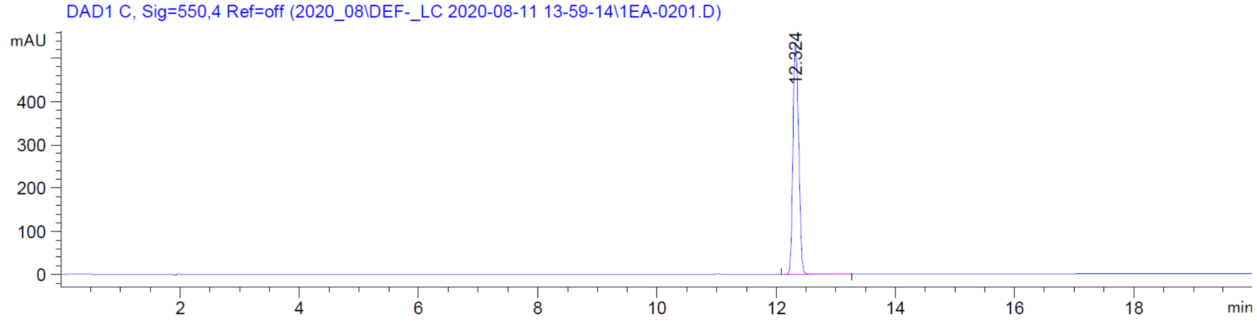
DAD1 C, Sig=550,4 Ref=off (2020_08\DEF-_LC 2020-08-11 13-59-14\1EA-0201.D)
mAU
400
300
200
100
0
12.324
2
4
6
8
10
12
14
16
18
min

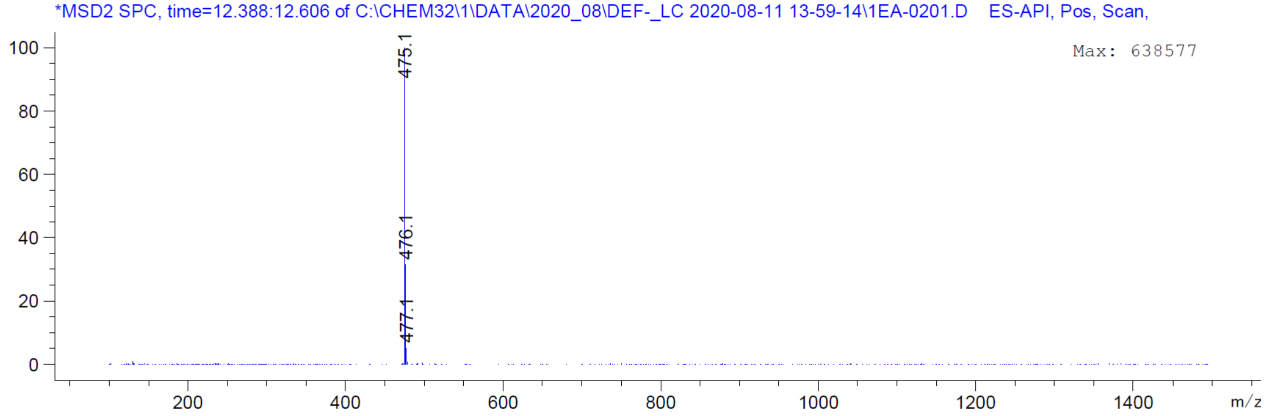
*MSD2 SPC, time=12.388:12.606 of C:\CHEM32\1\DATA\2020_08\DEF-_LC 2020-08-11 13-59-14\1EA-0201.D ES-API, Pos, Scan,
Max: 638577
100
80
60
40
20
0
475.1
476.1
477.1
200
400
600
800
1000
1200
1400
m/z

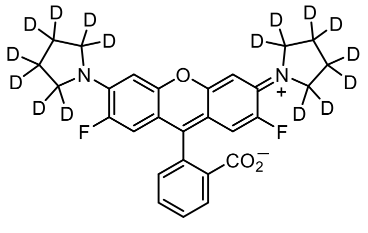

$\mathbf{25_D}$

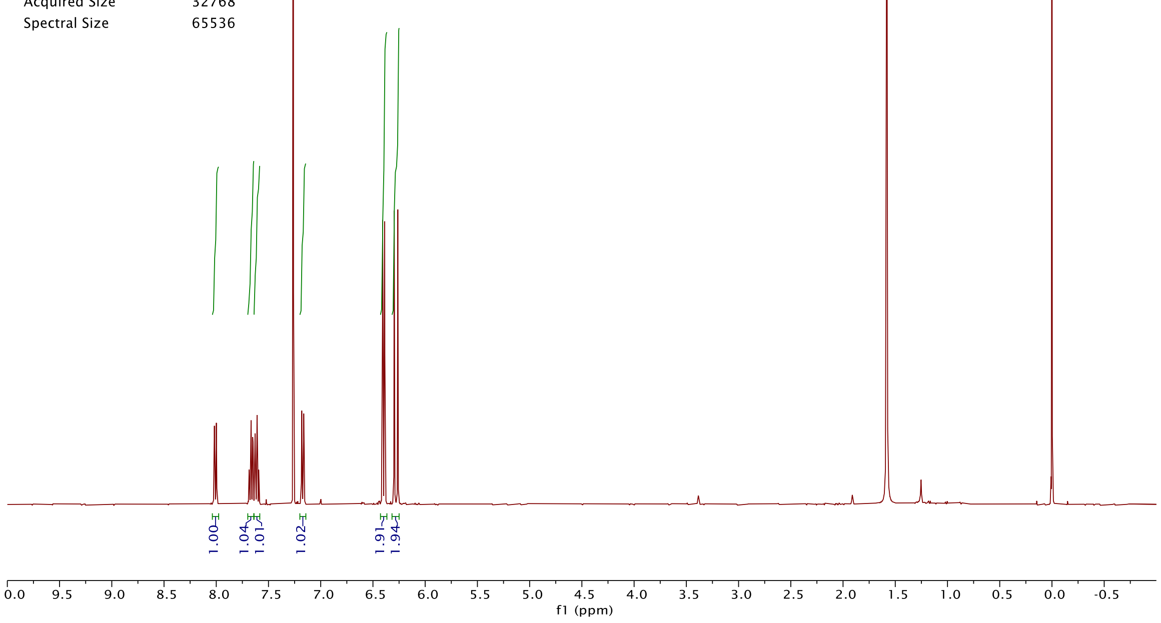

Origin Bruker BioSpin GmbH
Solvent CDCl3
Temperature 295.4
Pulse Sequence zg30
Experiment 1D
Number of Scans 16
Acquisition Date 2020-08-10T16:12:00
Spectrometer Frequency 400.13
Spectral Width 8012.8
Lowest Frequency -1544.8
Nucleus 1H
Acquired Size 32768
Spectral Size 65536
1.00
1.04
1.01
1.02
1.91
1.94
f1 (ppm)

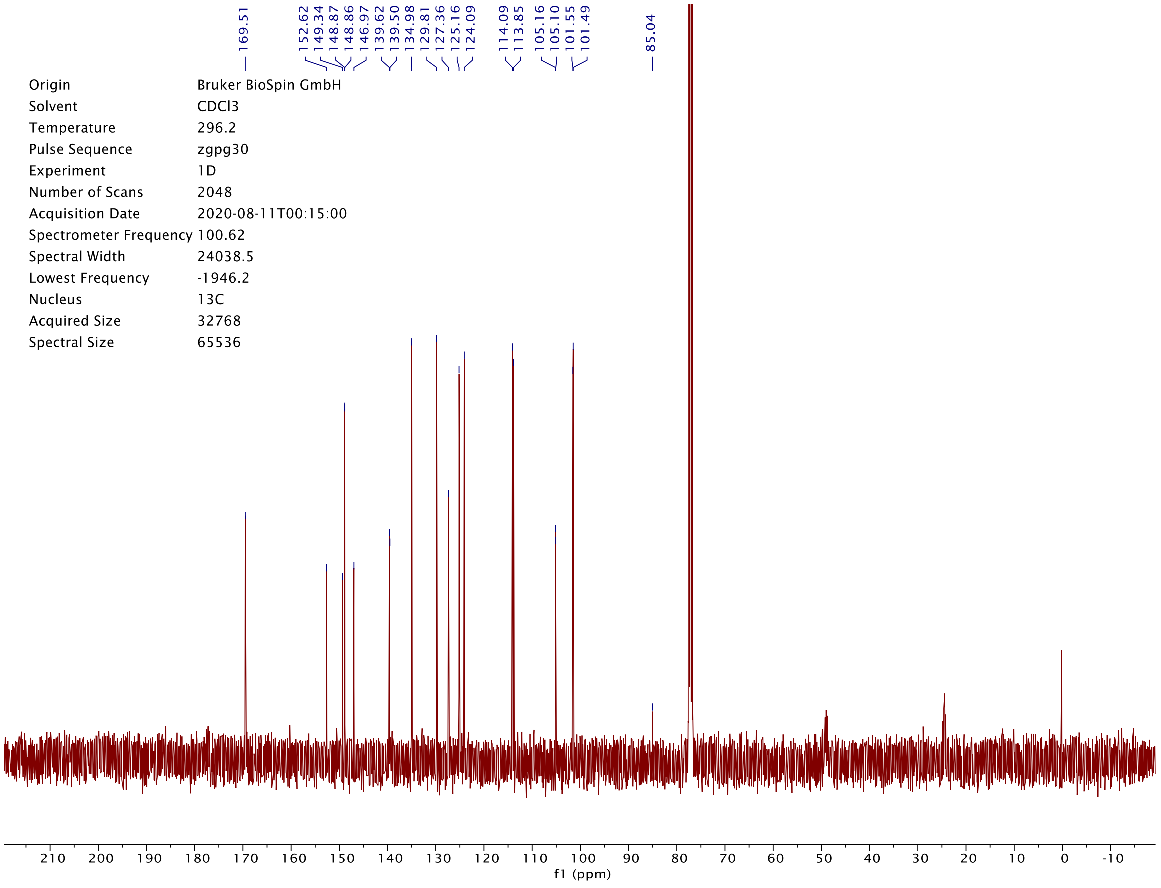

169.51
152.62
149.34
148.87
148.86
146.97
139.62
139.50
134.98
129.81
127.36
125.16
124.09
114.09
113.85
105.16
105.10
101.55
101.49
85.04
Origin Bruker BioSpin GmbH
Solvent CDCl3
Temperature 296.2
Pulse Sequence zgpg30
Experiment 1D
Number of Scans 2048
Acquisition Date 2020-08-11T00:15:00
Spectrometer Frequency 100.62
Spectral Width 24038.5
Lowest Frequency -1946.2
Nucleus 13C
Acquired Size 32768
Spectral Size 65536
f1 (ppm)

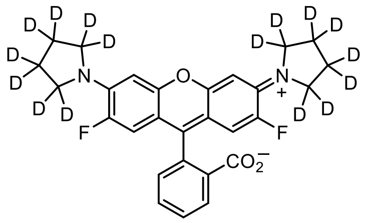

25_D

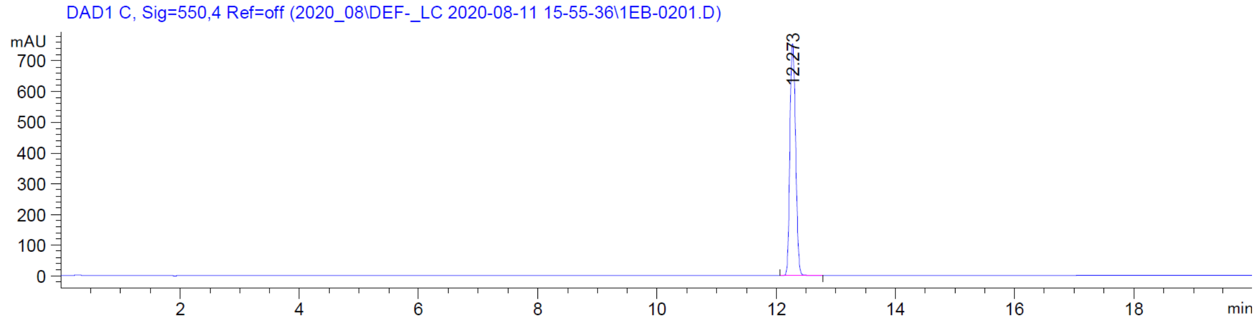
DAD1 C, Sig=550,4 Ref=off (2020_08\DEF-_LC 2020-08-11 15-55-36\1EB-0201.D)
mAU
12.273
min

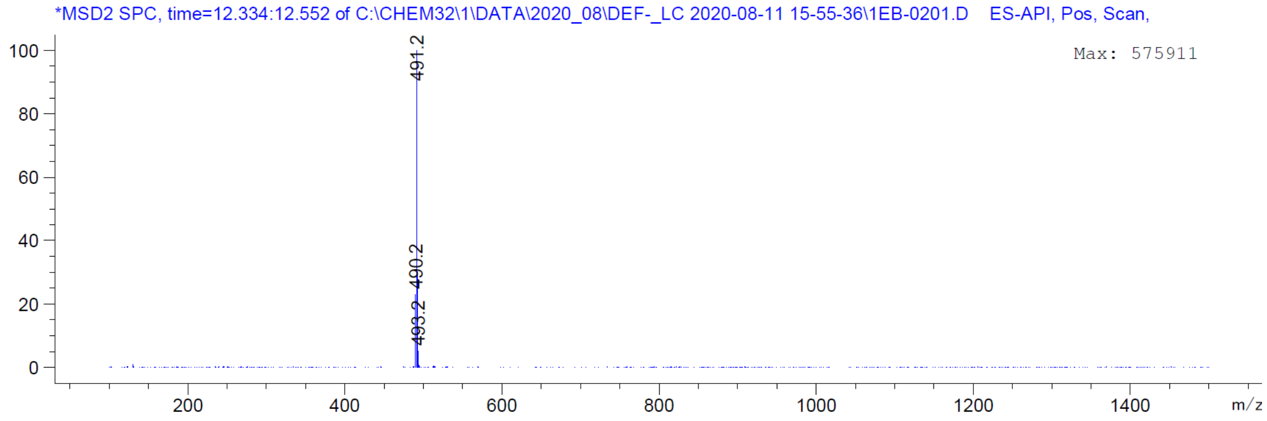
*MSD2 SPC, time=12.334:12.552 of C:\CHEM32\1\DATA\2020_08\DEF-_LC 2020-08-11 15-55-36\1EB-0201.D ES-API, Pos, Scan,
Max: 575911
491.2
490.2
493.2
m/z

Origin	Bruker BioSpin GmbH
Solvent	CDCl3
Temperature	300.0
Pulse Sequence	zg30
Experiment	1D
Number of Scans	16
Acquisition Date	2019-03-06T14:37:00
Spectrometer Frequency	400.13
Spectral Width	8012.8
Lowest Frequency	-1545.8
Nucleus	1H
Acquired Size	32768
Spectral Size	65536

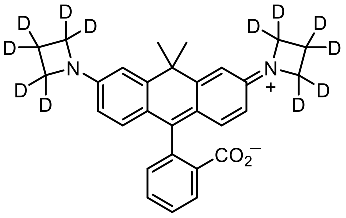

26_D

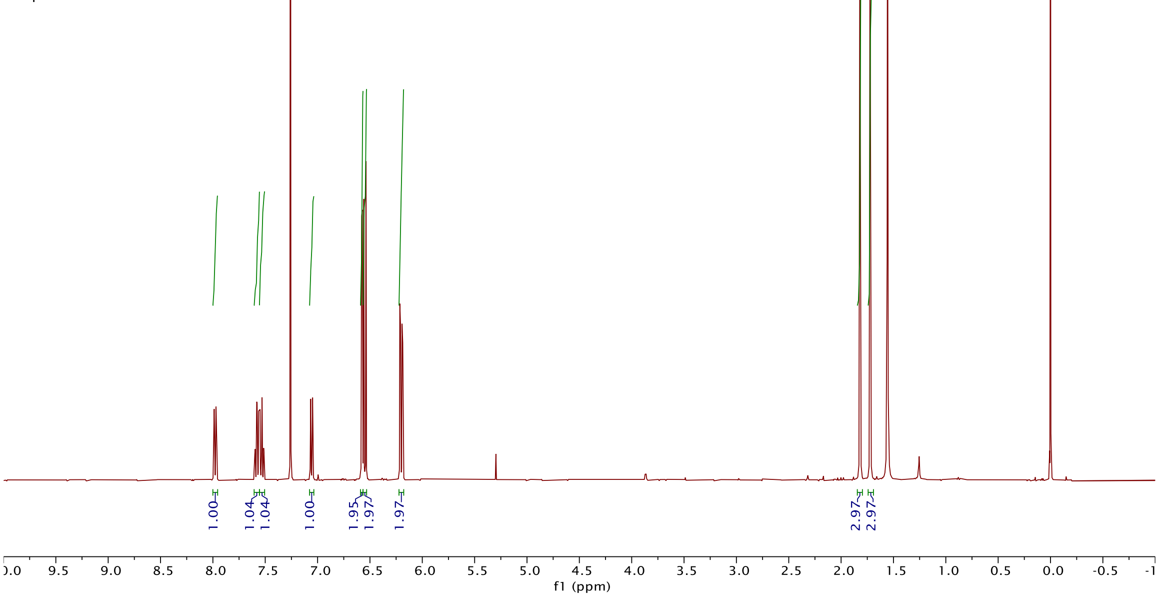

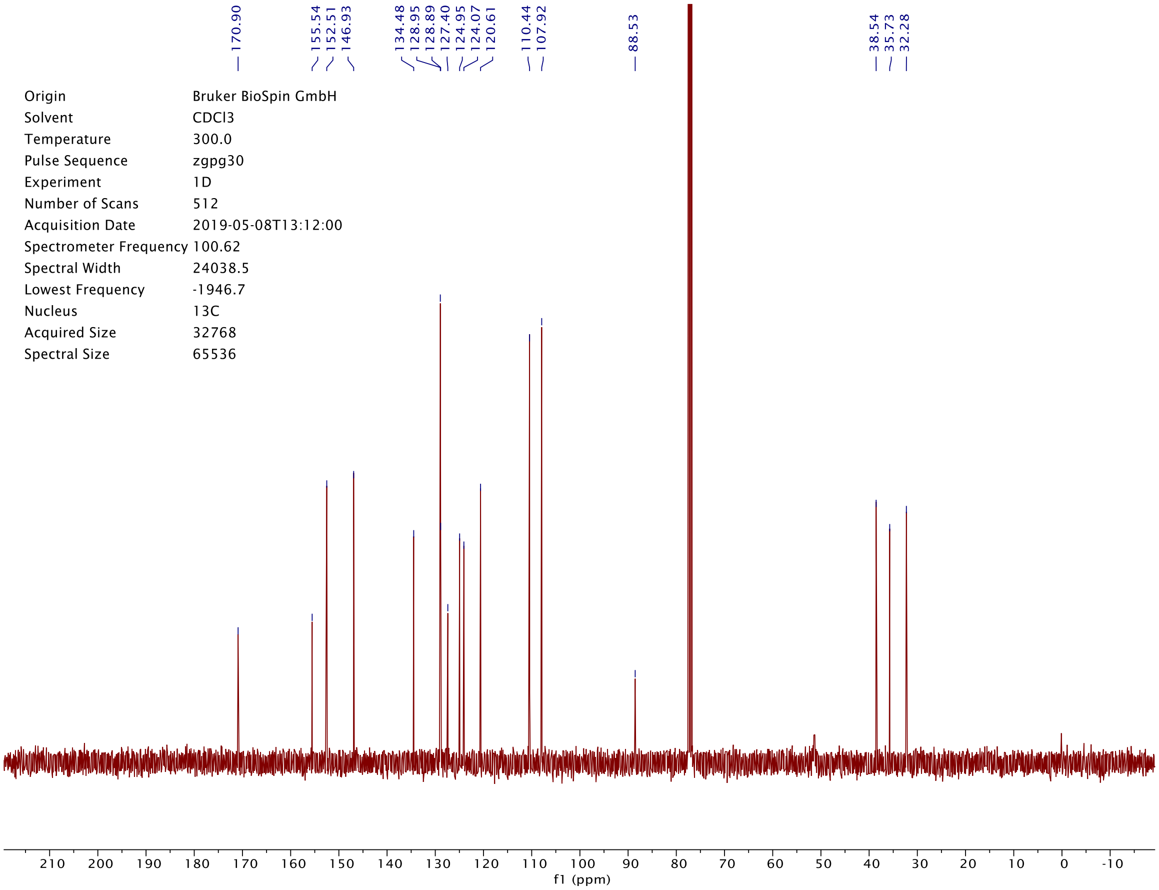

Origin	Bruker BioSpin GmbH
Solvent	CDCl3
Temperature	300.0
Pulse Sequence	zgpg30
Experiment	1D
Number of Scans	512
Acquisition Date	2019-05-08T13:12:00
Spectrometer Frequency	100.62
Spectral Width	24038.5
Lowest Frequency	-1946.7
Nucleus	13C
Acquired Size	32768
Spectral Size	65536

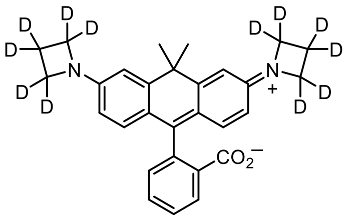

26_D

DAD1 D, Sig=600,4 Ref=off (2019_03\DAILY_SEQUENCE_LC 2019-03-05 13-54-35\2019_030000002.D)

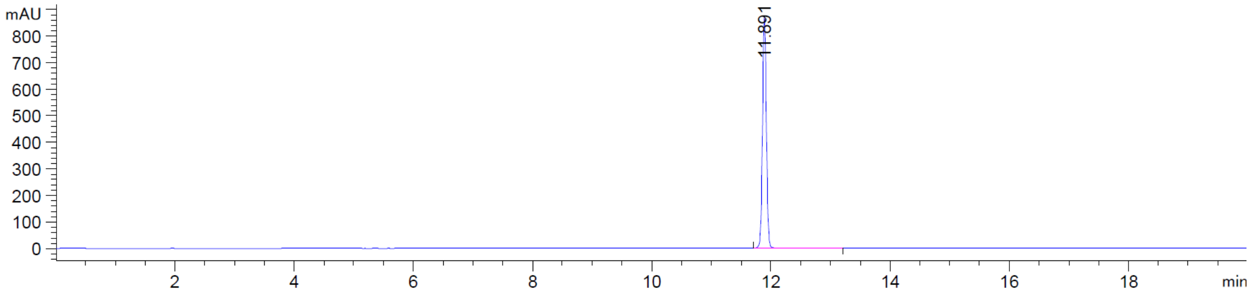

*MSD2 SPC, time=11.879:12.006 of C:\CHEM32\1\DATA\2019_03\DAILY_SEQUENCE_LC 2019-03-05 13-54-35\2019_030000002.D ES-

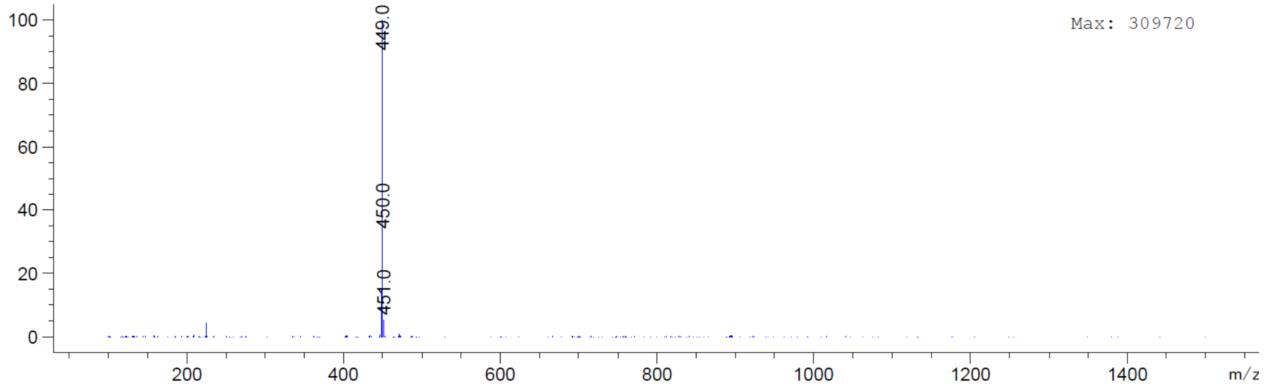

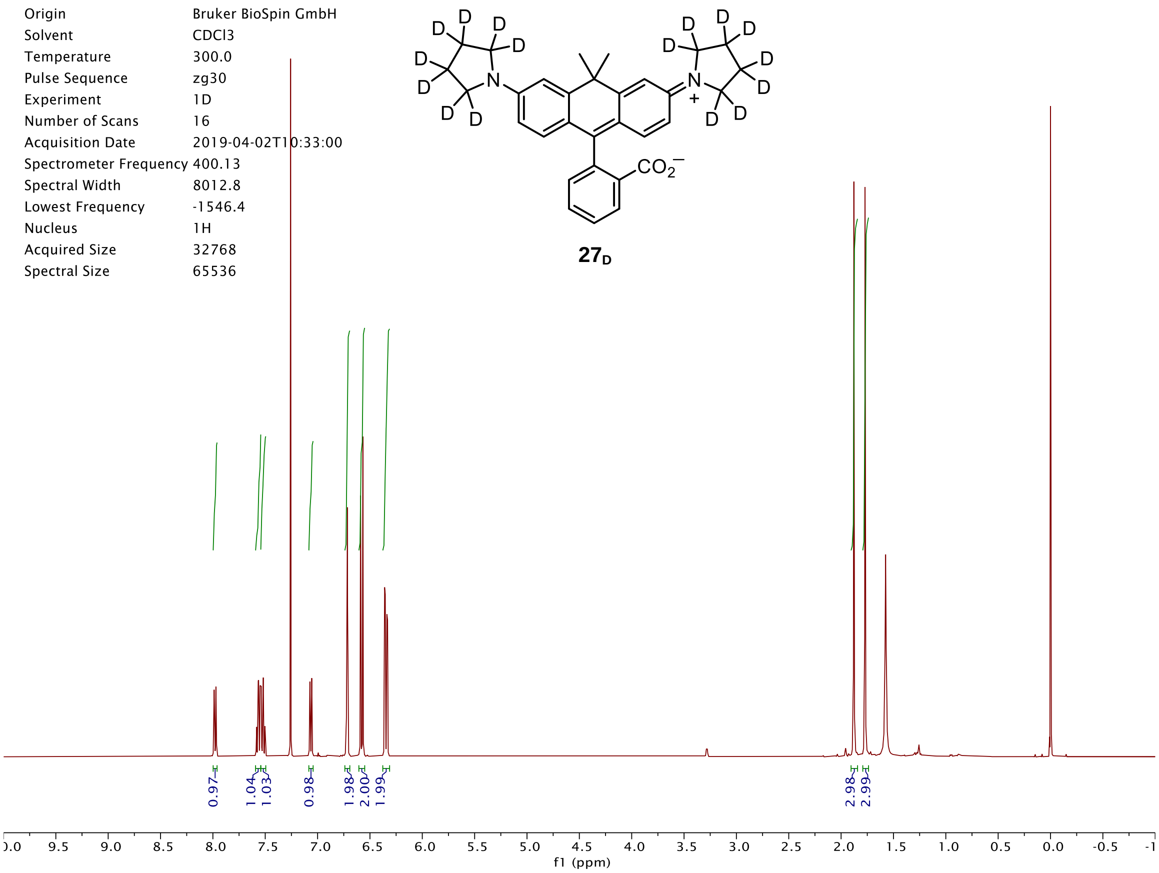
Origin Bruker BioSpin GmbH
Solvent CDCl3
Temperature 300.0
Pulse Sequence zg30
Experiment 1D
Number of Scans 16
Acquisition Date 2019-04-02T10:33:00
Spectrometer Frequency 400.13
Spectral Width 8012.8
Lowest Frequency -1546.4
Nucleus 1H
Acquired Size 32768
Spectral Size 65536
D
N
+
CO_2^-
0.97
1.04
1.03
0.98
1.98
2.00
1.99
2.98
2.99
f1 (ppm)

27_D

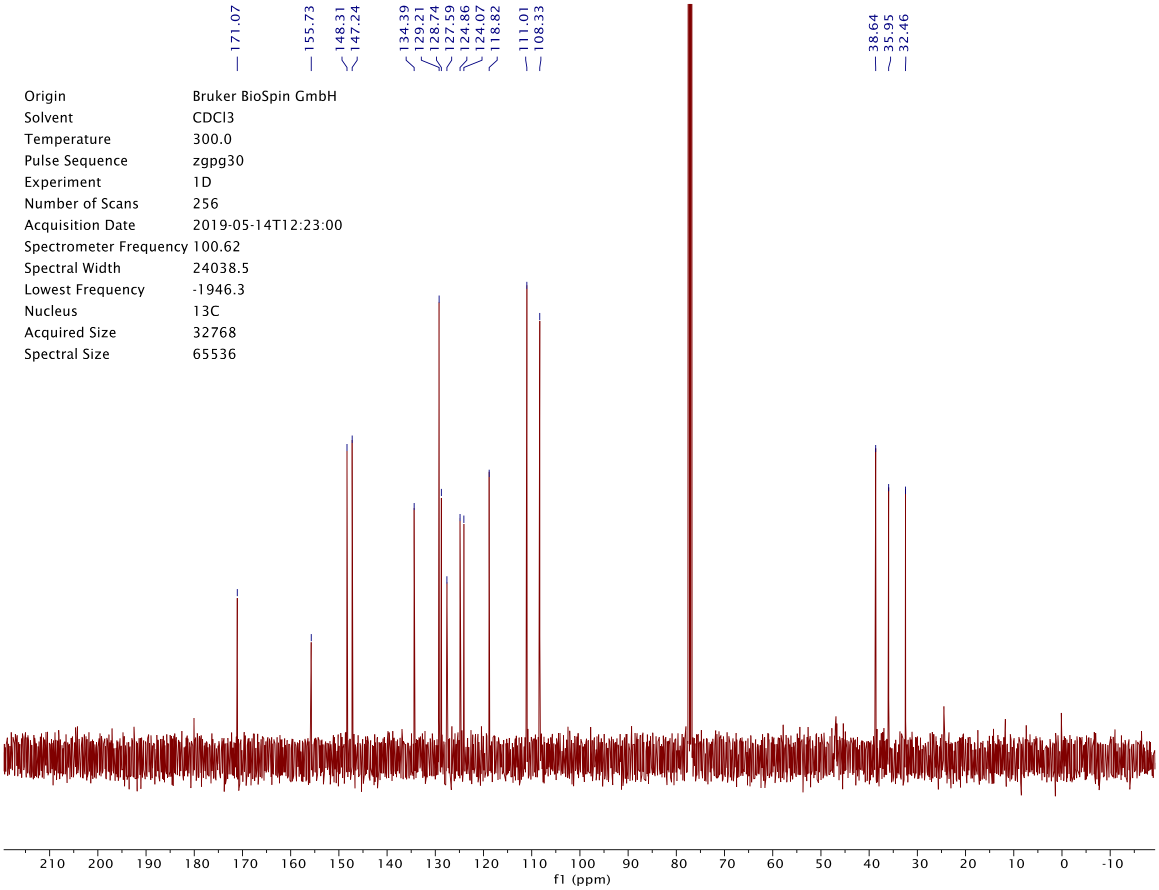
171.07
155.73
148.31
147.24
134.39
129.21
128.74
127.59
124.86
124.07
118.82
111.01
108.33
38.64
35.95
32.46
Origin Bruker BioSpin GmbH
Solvent CDCl3
Temperature 300.0
Pulse Sequence zgpg30
Experiment 1D
Number of Scans 256
Acquisition Date 2019-05-14T12:23:00
Spectrometer Frequency 100.62
Spectral Width 24038.5
Lowest Frequency -1946.3
Nucleus 13C
Acquired Size 32768
Spectral Size 65536
f1 (ppm)

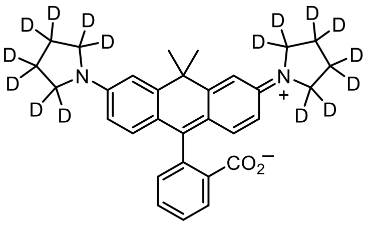

27_D

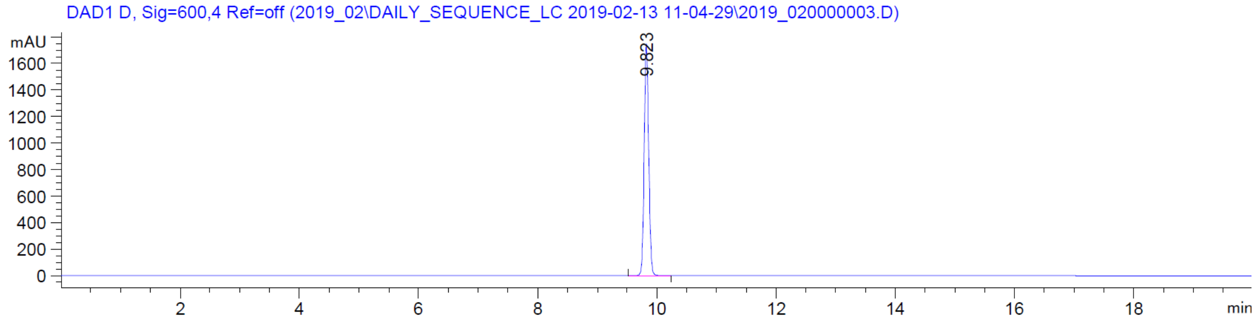
DAD1 D, Sig=600,4 Ref=off (2019_02\DAILY_SEQUENCE_LC 2019-02-13 11-04-29\2019_020000003.D)
mAU
9.823
min

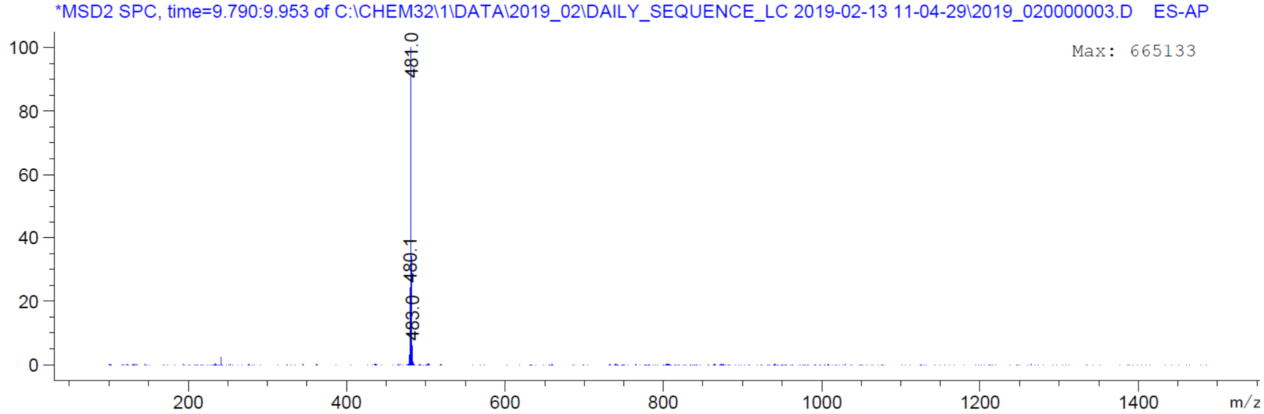
*MSD2 SPC, time=9.790:9.953 of C:\CHEM32\1\DATA\2019_02\DAILY_SEQUENCE_LC 2019-02-13 11-04-29\2019_020000003.D ES-AP
Max: 665133
481.0
480.1
483.0
m/z

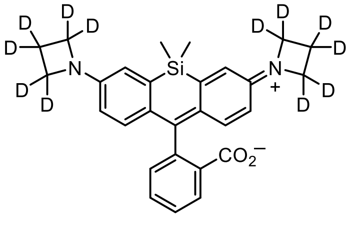

D
Si
N
CO_2^-

$\mathbf{28_D}$

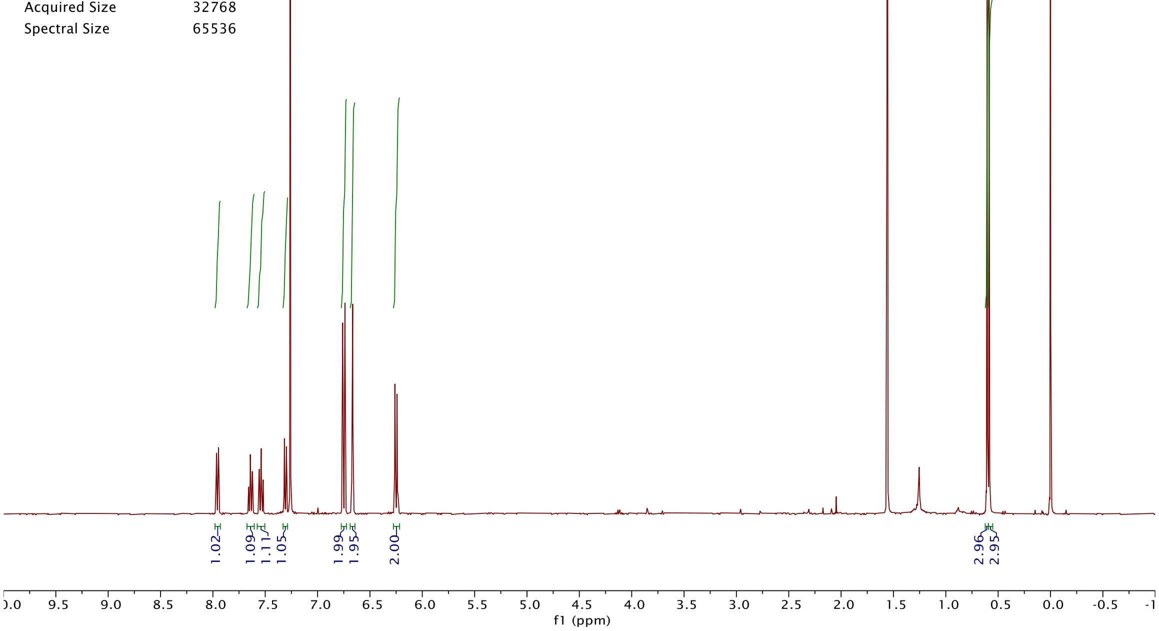

Origin Bruker BioSpin GmbH
Solvent CDCl3
Temperature 295.6
Pulse Sequence zg30
Experiment 1D
Number of Scans 16
Acquisition Date 2017-10-13T12:10:00
Spectrometer Frequency 400.13
Spectral Width 8012.8
Lowest Frequency -1545.2
Nucleus 1H
Acquired Size 32768
Spectral Size 65536
1.02
1.09
1.11
1.05
1.99
1.95
2.00
2.96
2.95
9.5 9.0 8.5 8.0 7.5 7.0 6.5 6.0 5.5 5.0 4.5 4.0 3.5 3.0 2.5 2.0 1.5 1.0 0.5 0.0 -0.5
f1 (ppm)

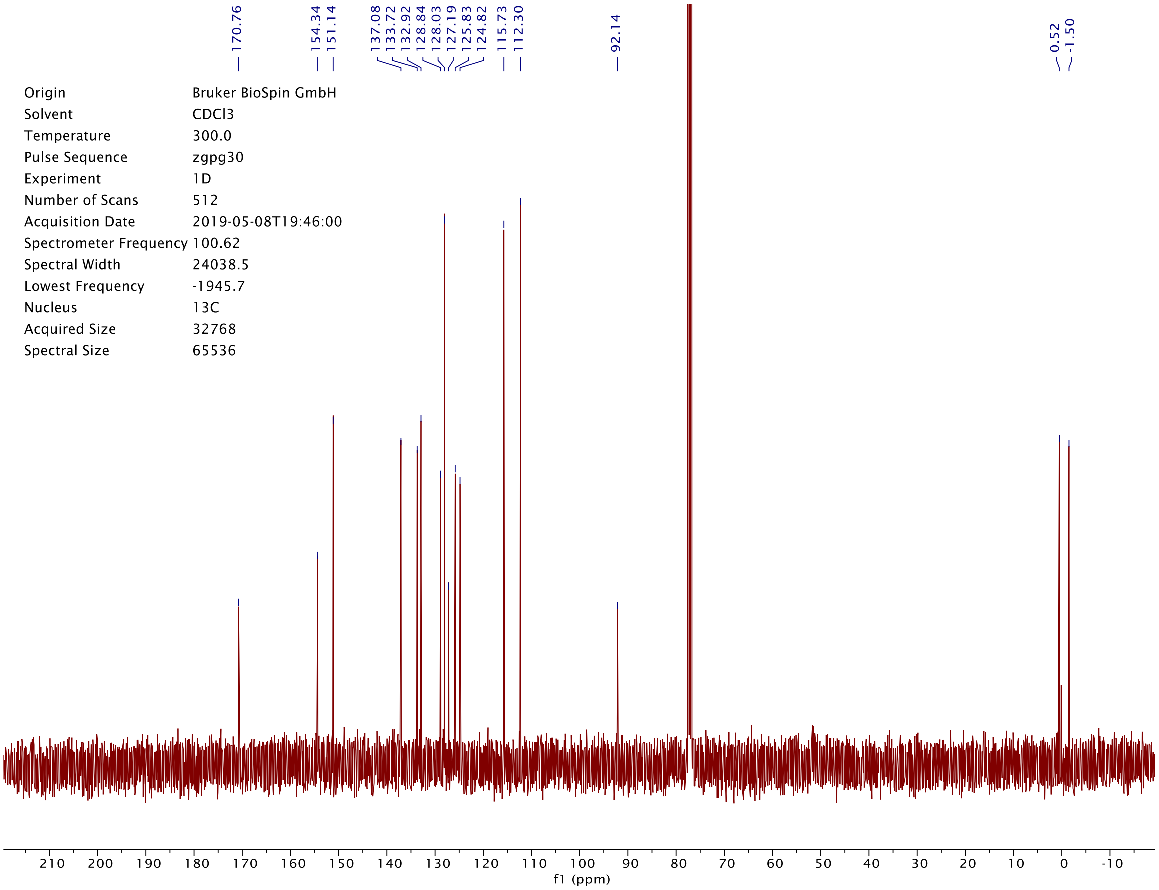

170.76
154.34
151.14
137.08
133.72
132.92
128.84
128.03
127.19
125.83
124.82
115.73
112.30
92.14
0.52
-1.50
Origin Bruker BioSpin GmbH
Solvent CDCl3
Temperature 300.0
Pulse Sequence zgpg30
Experiment 1D
Number of Scans 512
Acquisition Date 2019-05-08T19:46:00
Spectrometer Frequency 100.62
Spectral Width 24038.5
Lowest Frequency -1945.7
Nucleus 13C
Acquired Size 32768
Spectral Size 65536
210 200 190 180 170 160 150 140 130 120 110 100 90 80 70 60 50 40 30 20 10 0 -10
f1 (ppm)

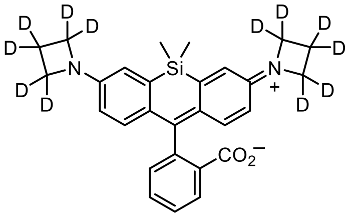

28_D

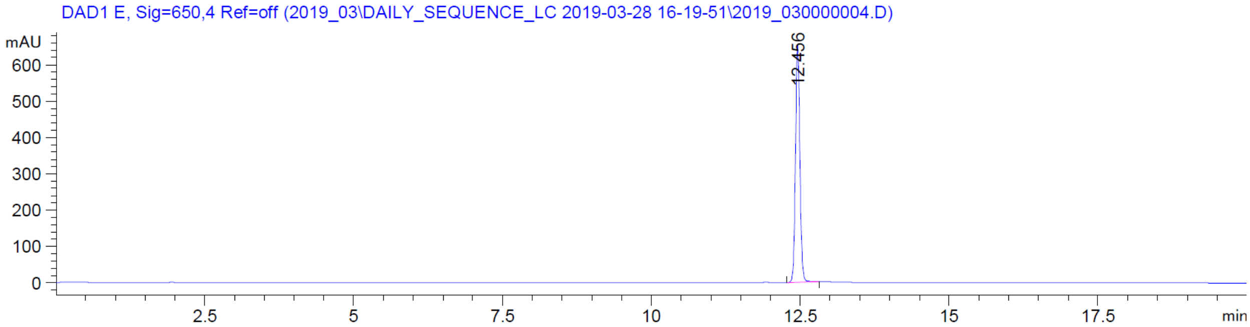
DAD1 E, Sig=650,4 Ref=off (2019_03\DAILY_SEQUENCE_LC 2019-03-28 16-19-51\2019_030000004.D)
mAU
12.456
min

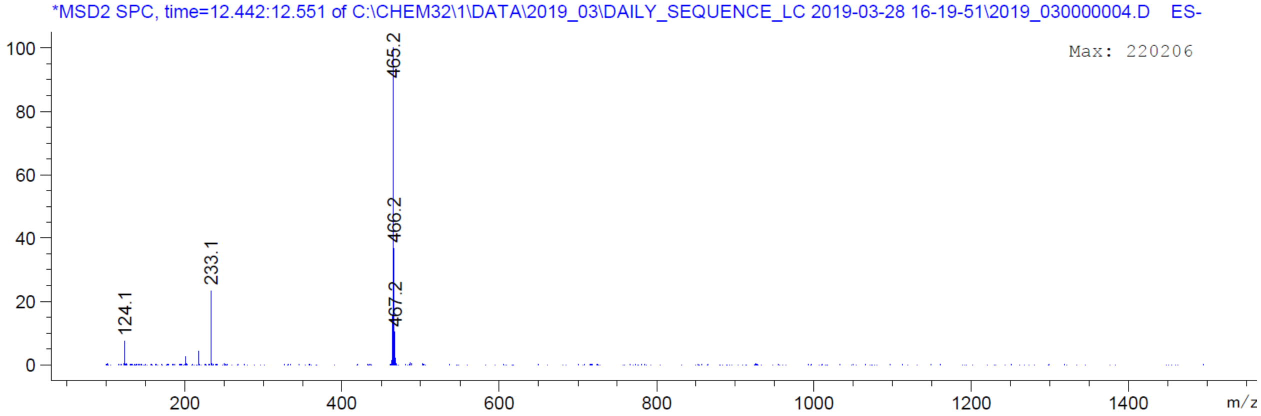
*MSD2 SPC, time=12.442:12.551 of C:\CHEM32\1\DATA\2019_03\DAILY_SEQUENCE_LC 2019-03-28 16-19-51\2019_030000004.D ES-
Max: 220206
465.2
466.2
467.2
233.1
124.1
m/z

Origin	Bruker BioSpin GmbH
Solvent	CDCl3
Temperature	295.5
Pulse Sequence	zg30
Experiment	1D
Number of Scans	16
Acquisition Date	2013-05-24T09:18:00
Spectrometer Frequency	400.13
Spectral Width	8223.7
Lowest Frequency	-1647.3
Nucleus	1H
Acquired Size	32768
Spectral Size	65536

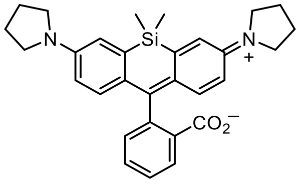

29

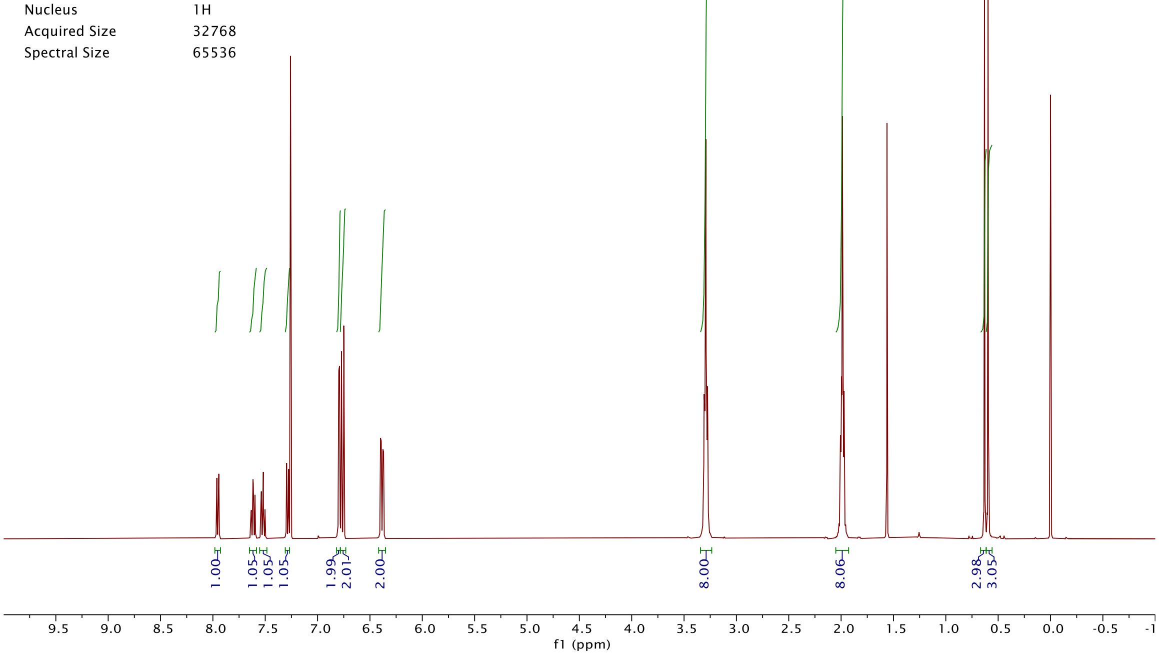

Origin	Bruker BioSpin GmbH
Solvent	CDCl3
Temperature	296.8
Pulse Sequence	zgpg30
Experiment	1D
Number of Scans	2048
Acquisition Date	2013-05-25T00:01:00
Spectrometer Frequency	100.62
Spectral Width	24038.5
Lowest Frequency	-1946.3
Nucleus	13C
Acquired Size	32768
Spectral Size	65536

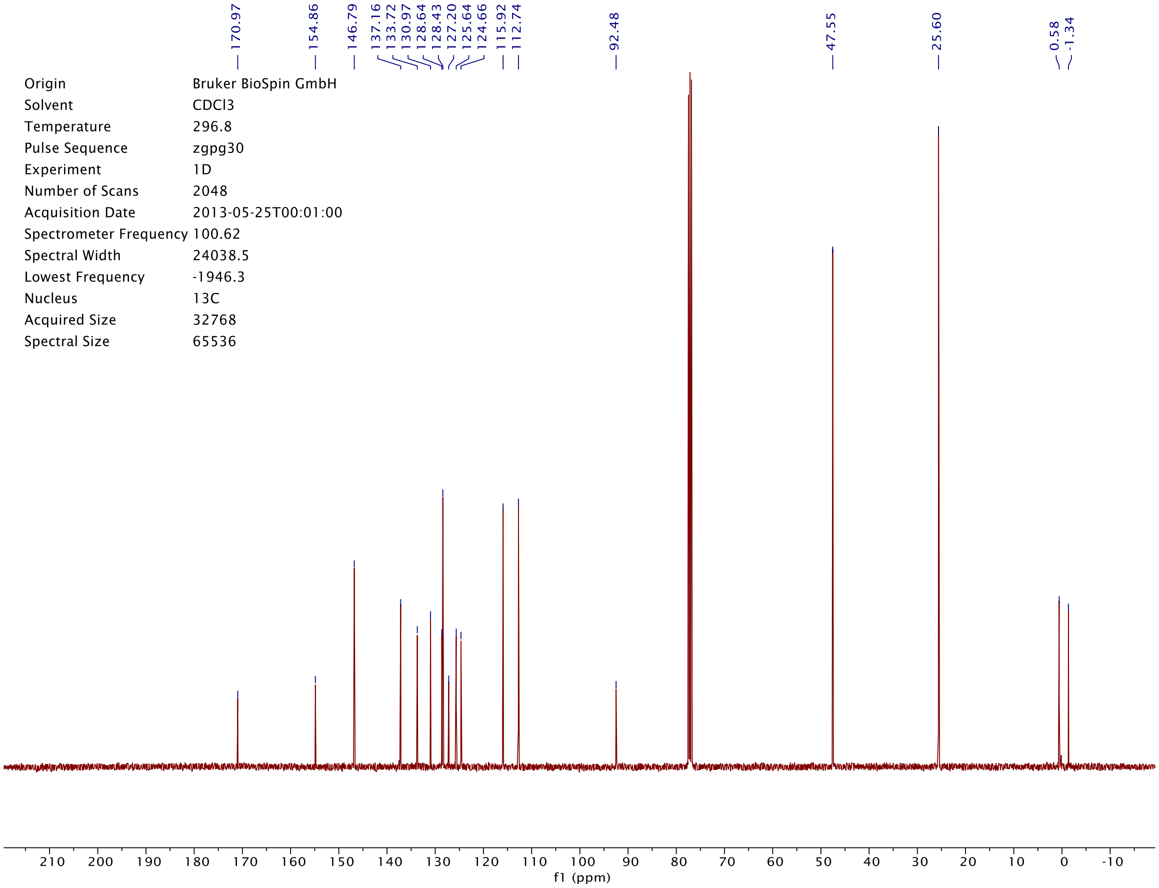

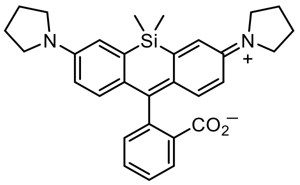

N
Si
N
+
CO_2^-

29

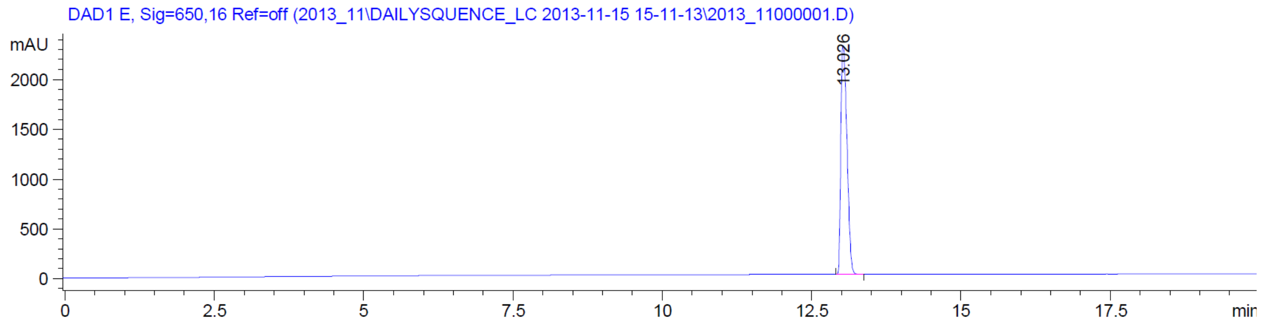

DAD1 E, Sig=650,16 Ref=off (2013_11\DAILYSQUENCE_LC 2013-11-15 15-11-13\2013_11000001.D)
mAU
2000
1500
1000
500
0
13.026
0
2.5
5
7.5
10
12.5
15
17.5
min

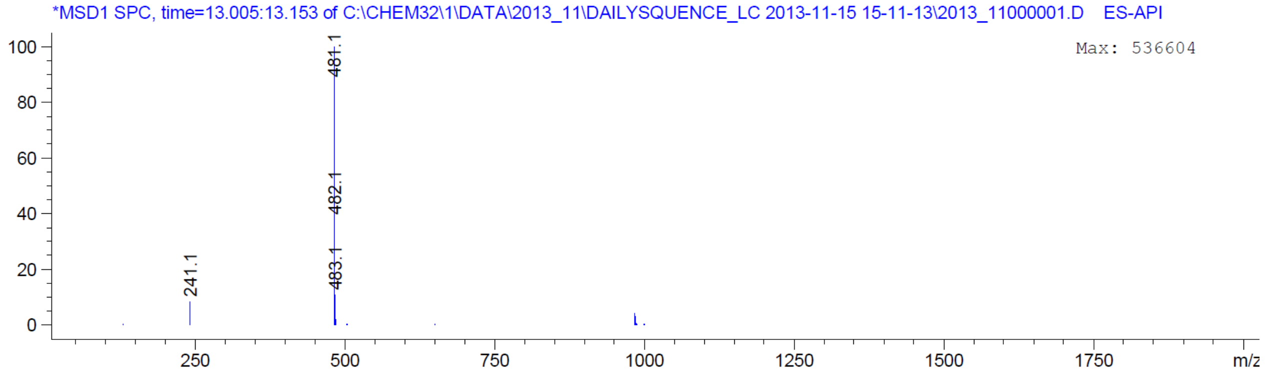

*MSD1 SPC, time=13.005:13.153 of C:\CHEM32\1\DATA\2013_11\DAILYSQUENCE_LC 2013-11-15 15-11-13\2013_11000001.D ES-API
Max: 536604
100
80
60
40
20
0
481.1
482.1
483.1
241.1
250
500
750
1000
1250
1500
1750
m/z

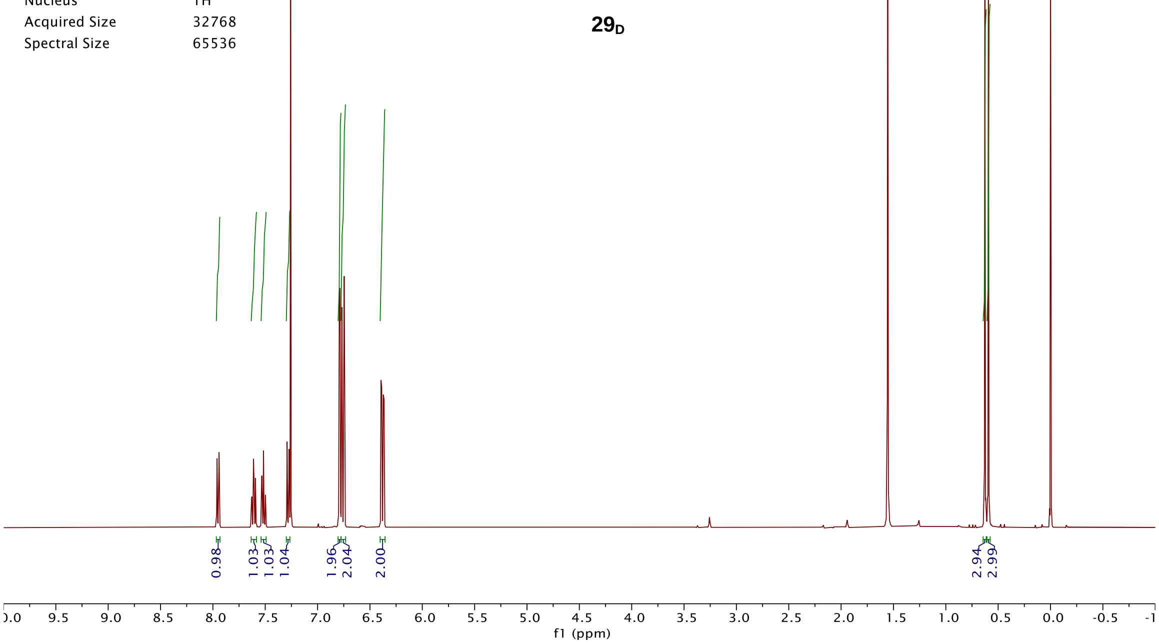
Origin Bruker BioSpin GmbH
Solvent CDCl3
Temperature 300.0
Pulse Sequence zg30
Experiment 1D
Number of Scans 16
Acquisition Date 2019-04-02T10:28:00
Spectrometer Frequency 400.13
Spectral Width 8012.8
Lowest Frequency -1546.6
Nucleus 1H
Acquired Size 32768
Spectral Size 65536
0.98
1.03
1.03
1.04
1.96
2.04
2.00
2.94
2.99
f1 (ppm)

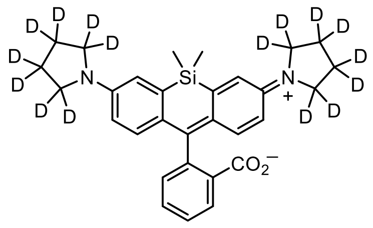
Si
N
N
+
D
CO_2^-

$\mathbf{29_D}$

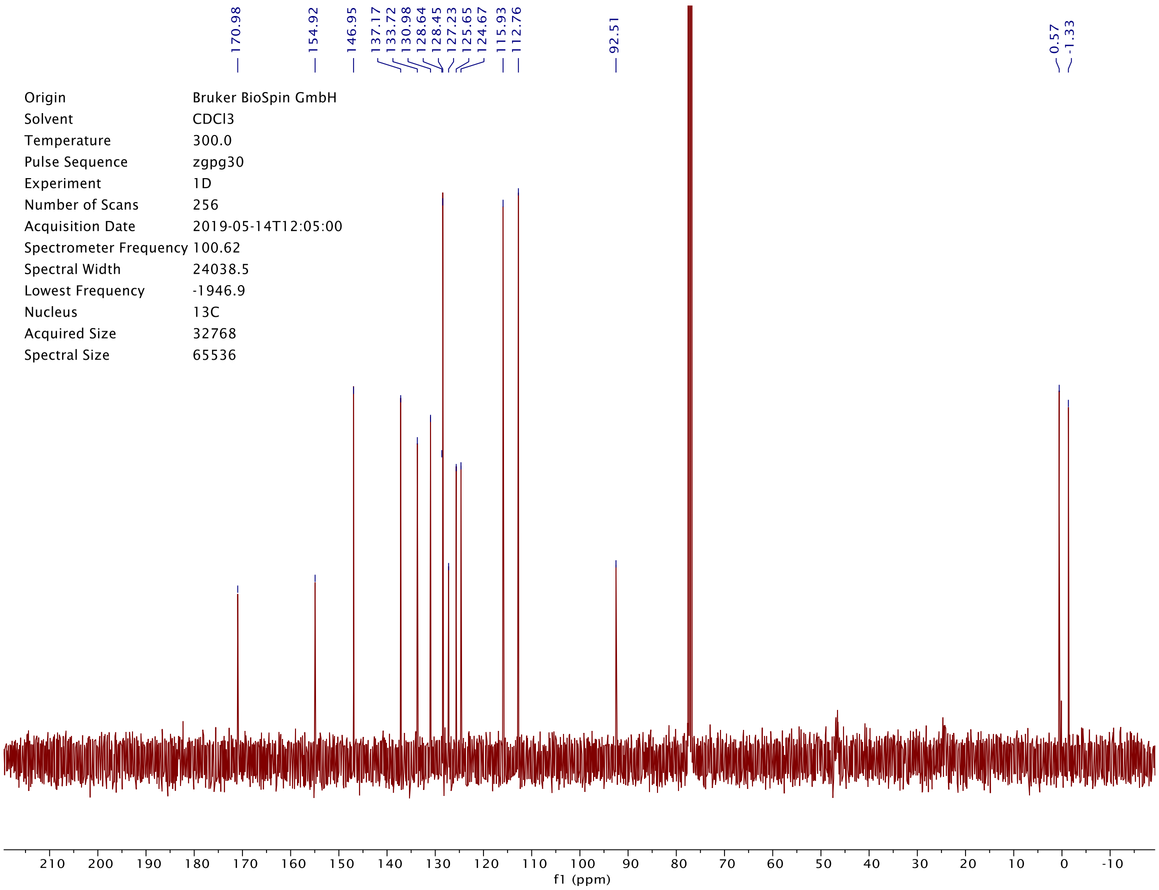
170.98
154.92
146.95
137.17
133.72
130.98
128.64
128.45
127.23
125.65
124.67
115.93
112.76
92.51
0.57
-1.33
Origin Bruker BioSpin GmbH
Solvent CDCl3
Temperature 300.0
Pulse Sequence zgpg30
Experiment 1D
Number of Scans 256
Acquisition Date 2019-05-14T12:05:00
Spectrometer Frequency 100.62
Spectral Width 24038.5
Lowest Frequency -1946.9
Nucleus 13C
Acquired Size 32768
Spectral Size 65536
f1 (ppm)

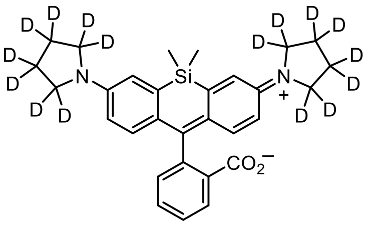

29_D

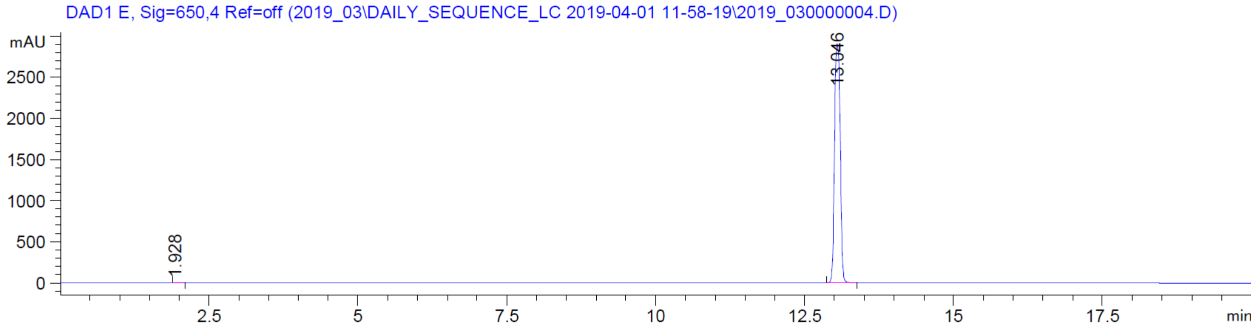

DAD1 E, Sig=650,4 Ref=off (2019_03\DAILY_SEQUENCE_LC 2019-04-01 11-58-19\2019_030000004.D)
mAU
2500
2000
1500
1000
500
0
1.928
13.046
2.5
5
7.5
10
12.5
15
17.5
min

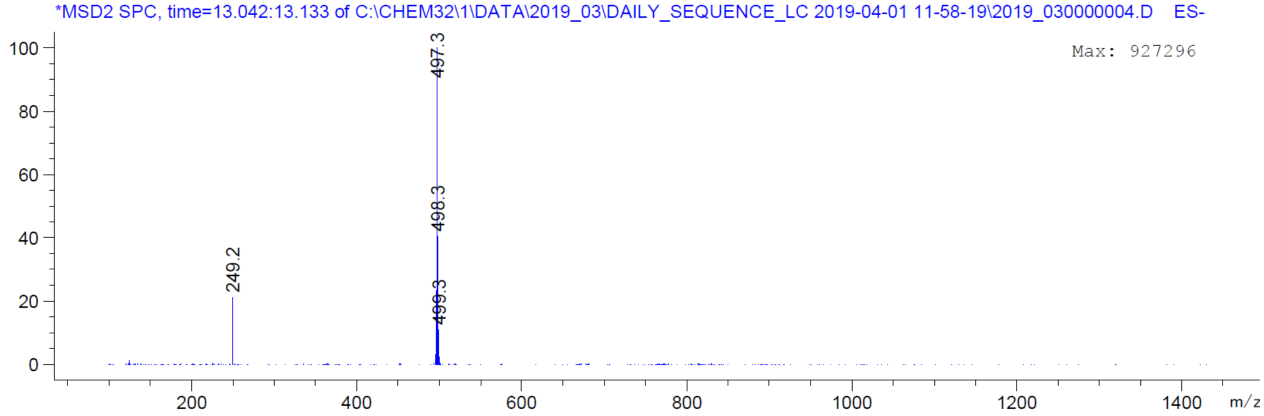

*MSD2 SPC, time=13.042:13.133 of C:\CHEM32\1\DATA\2019_03\DAILY_SEQUENCE_LC 2019-04-01 11-58-19\2019_030000004.D ES-
Max: 927296
100
80
60
40
20
0
497.3
498.3
499.3
249.2
200
400
600
800
1000
1200
1400
m/z

Origin	Bruker BioSpin GmbH
Solvent	CDCl3
Temperature	300.0
Pulse Sequence	zg30
Experiment	1D
Number of Scans	16
Acquisition Date	2019-04-02T10:14:00
Spectrometer Frequency	400.13
Spectral Width	8012.8
Lowest Frequency	-1545.9
Nucleus	1H
Acquired Size	32768
Spectral Size	65536

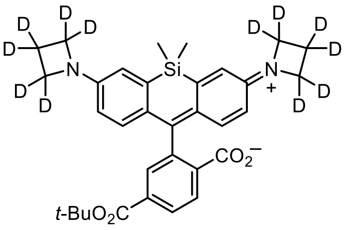

S16

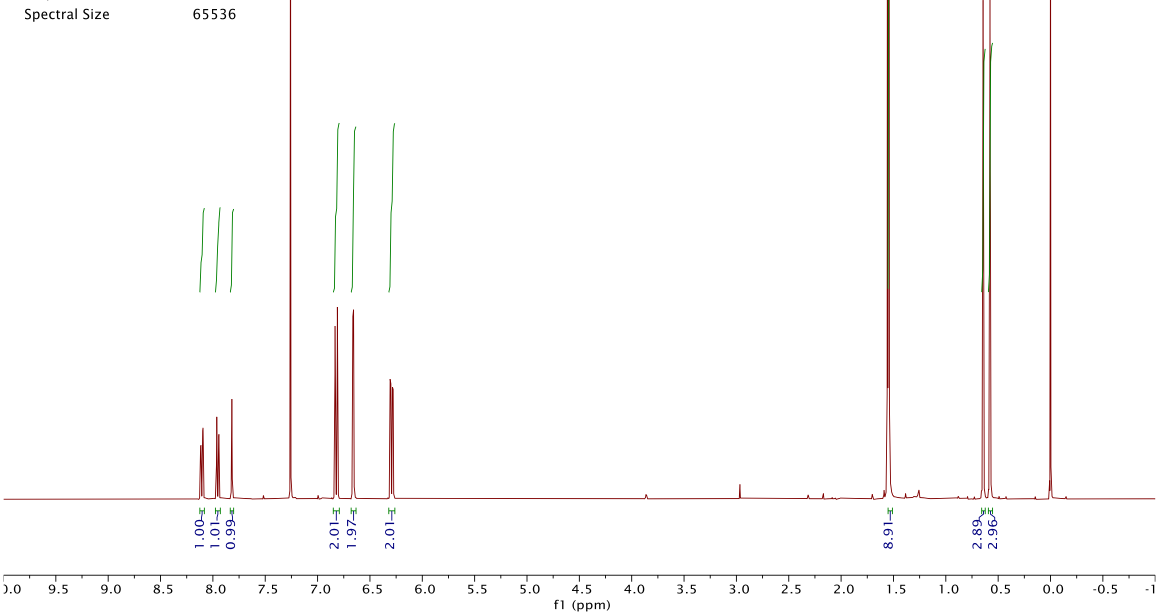

Origin	Bruker BioSpin GmbH
Solvent	CDCl3
Temperature	300.0
Pulse Sequence	zgpg30
Experiment	1D
Number of Scans	512
Acquisition Date	2019-11-27T12:19:00
Spectrometer Frequency	100.62
Spectral Width	24038.5
Lowest Frequency	-1945.8
Nucleus	13C
Acquired Size	32768
Spectral Size	65536

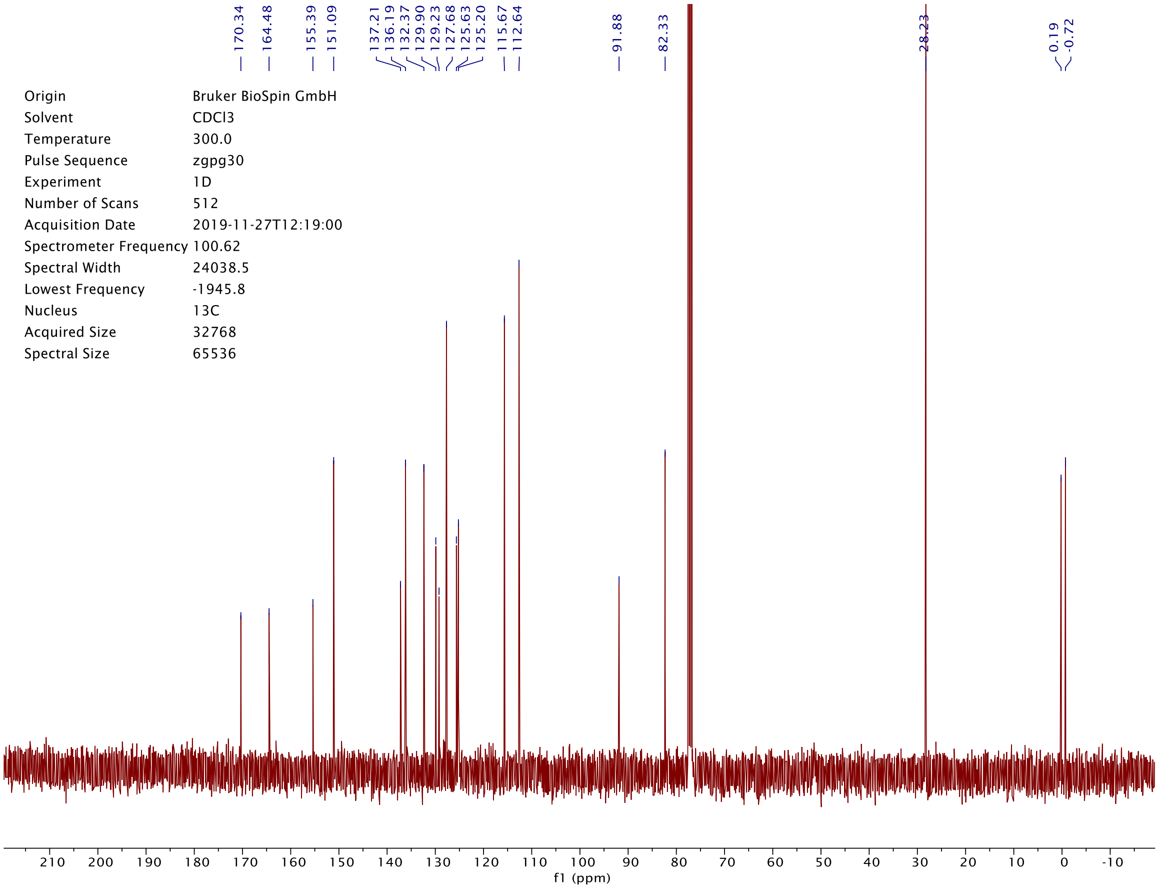

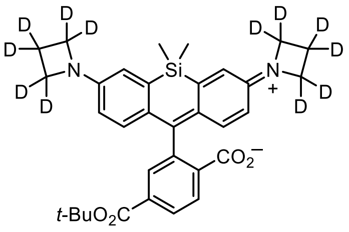

S16

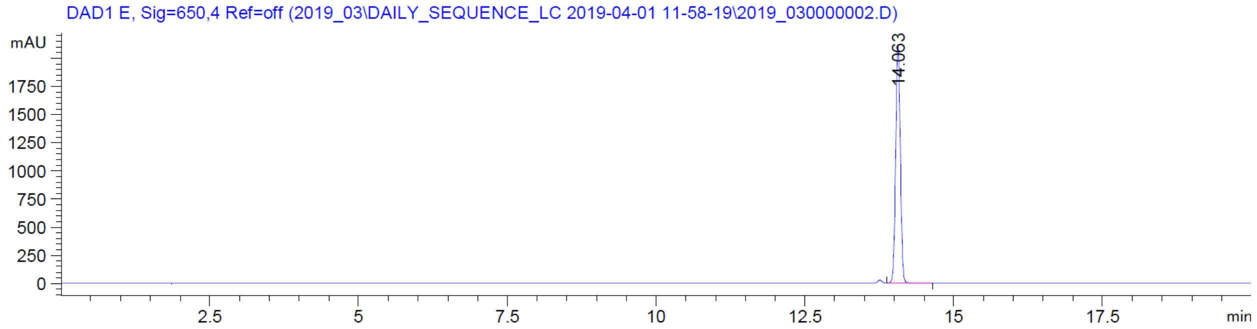

DAD1 E, Sig=650,4 Ref=off (2019_03\DAILY_SEQUENCE_LC 2019-04-01 11-58-19\2019_030000002.D)
mAU
14.063
min

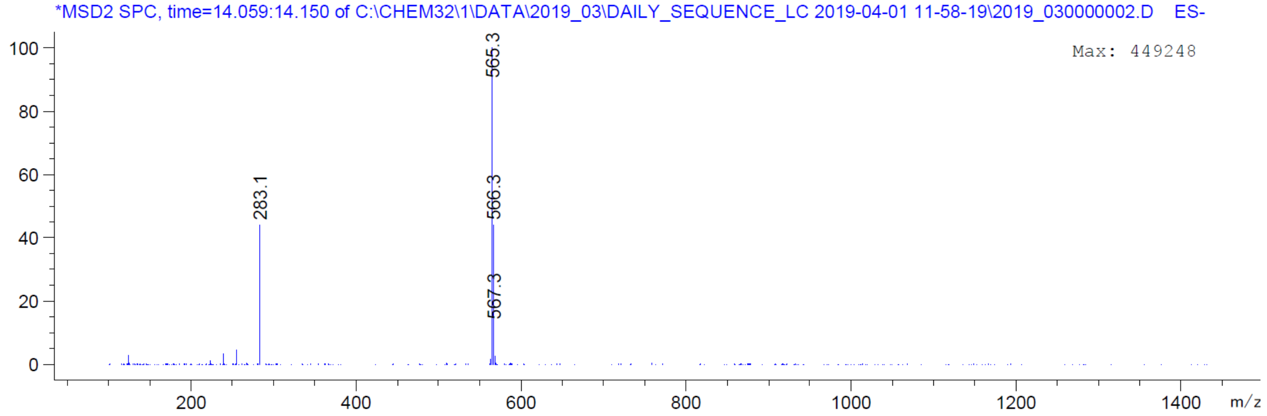

*MSD2 SPC, time=14.059:14.150 of C:\CHEM32\1\DATA\2019_03\DAILY_SEQUENCE_LC 2019-04-01 11-58-19\2019_030000002.D ES-
Max: 449248
565.3
283.1
566.3
567.3
m/z

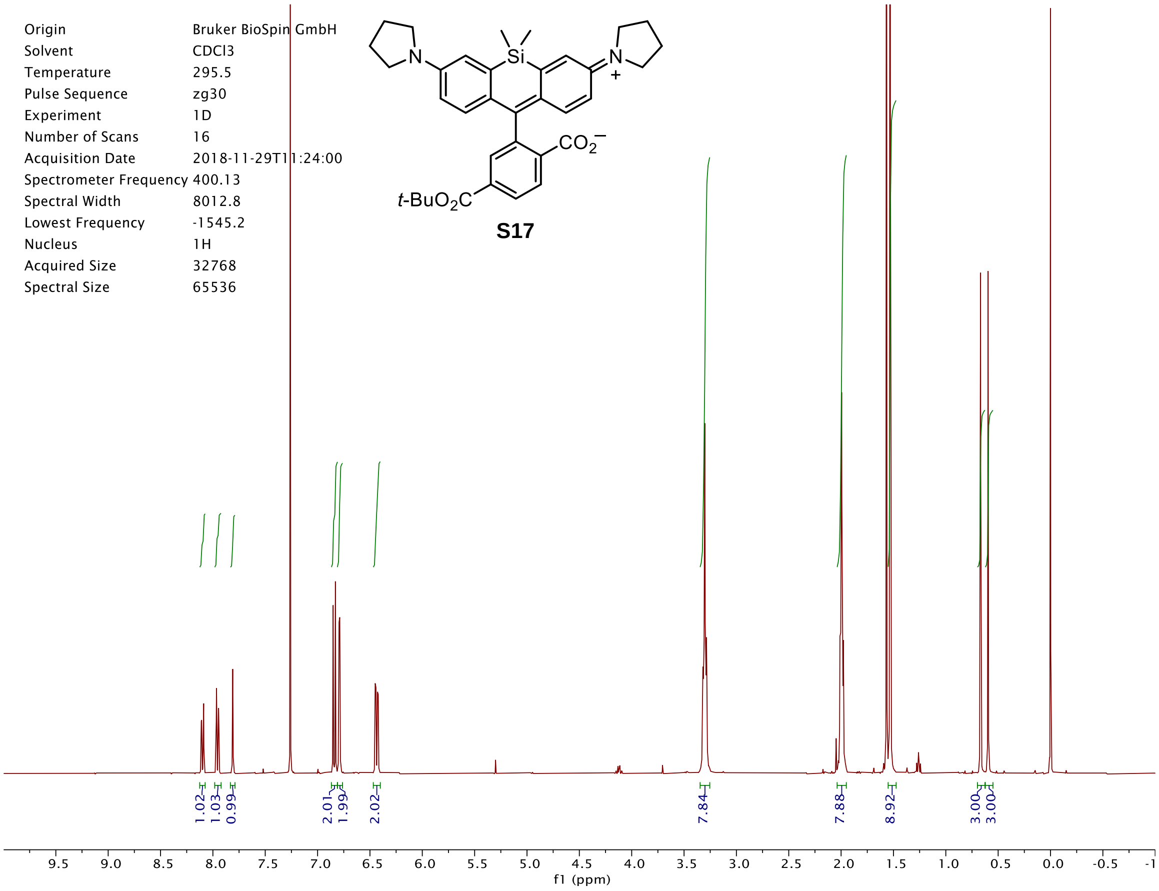
Origin Bruker BioSpin GmbH
Solvent CDCl3
Temperature 295.5
Pulse Sequence zg30
Experiment 1D
Number of Scans 16
Acquisition Date 2018-11-29T11:24:00
Spectrometer Frequency 400.13
Spectral Width 8012.8
Lowest Frequency -1545.2
Nucleus 1H
Acquired Size 32768
Spectral Size 65536
Si
N
N
+
CO_2^-
t-BuO_2C
S17
1.02
1.03
0.99
2.01
1.99
2.02
7.84
7.88
8.92
3.00
3.00
9.5 9.0 8.5 8.0 7.5 7.0 6.5 6.0 5.5 5.0 4.5 4.0 3.5 3.0 2.5 2.0 1.5 1.0 0.5 0.0 -0.5 -1
f1 (ppm)

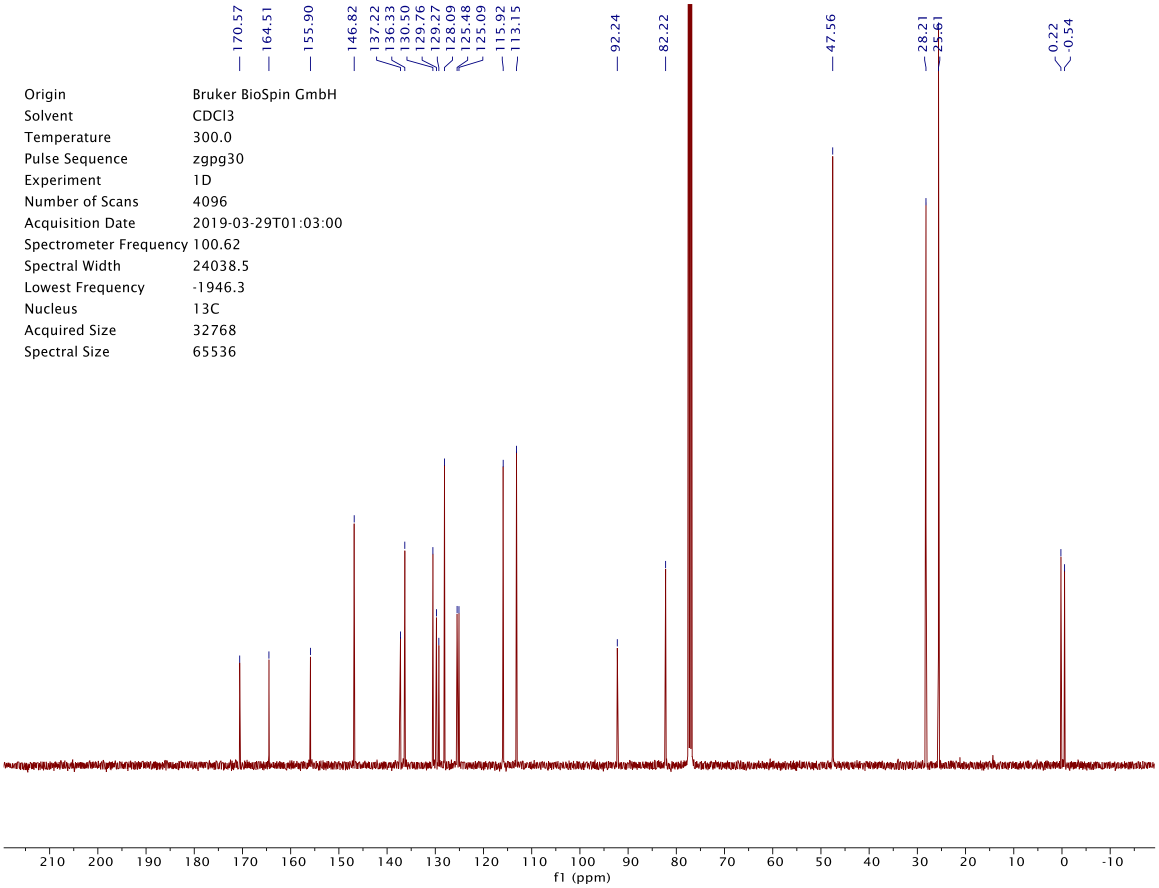
170.57
164.51
155.90
146.82
137.22
136.33
130.50
129.76
129.27
128.09
125.48
125.09
115.92
113.15
92.24
82.22
47.56
28.21
25.61
0.22
-0.54
Origin Bruker BioSpin GmbH
Solvent CDCl3
Temperature 300.0
Pulse Sequence zgpg30
Experiment 1D
Number of Scans 4096
Acquisition Date 2019-03-29T01:03:00
Spectrometer Frequency 100.62
Spectral Width 24038.5
Lowest Frequency -1946.3
Nucleus 13C
Acquired Size 32768
Spectral Size 65536
210 200 190 180 170 160 150 140 130 120 110 100 90 80 70 60 50 40 30 20 10 0 -10
f1 (ppm)

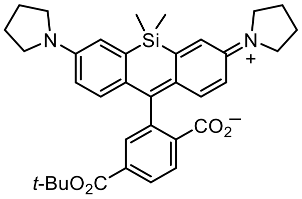
Si
N
N
+
CO2−
t-BuO2C

S17

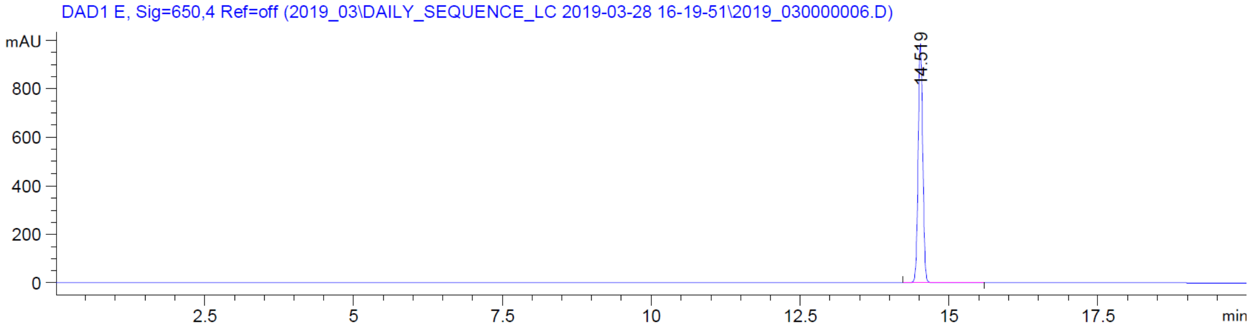
DAD1 E, Sig=650,4 Ref=off (2019_03\DAILY_SEQUENCE_LC 2019-03-28 16-19-51\2019_030000006.D)
mAU
800
600
400
200
0
14.519
2.5
5
7.5
10
12.5
15
17.5
min

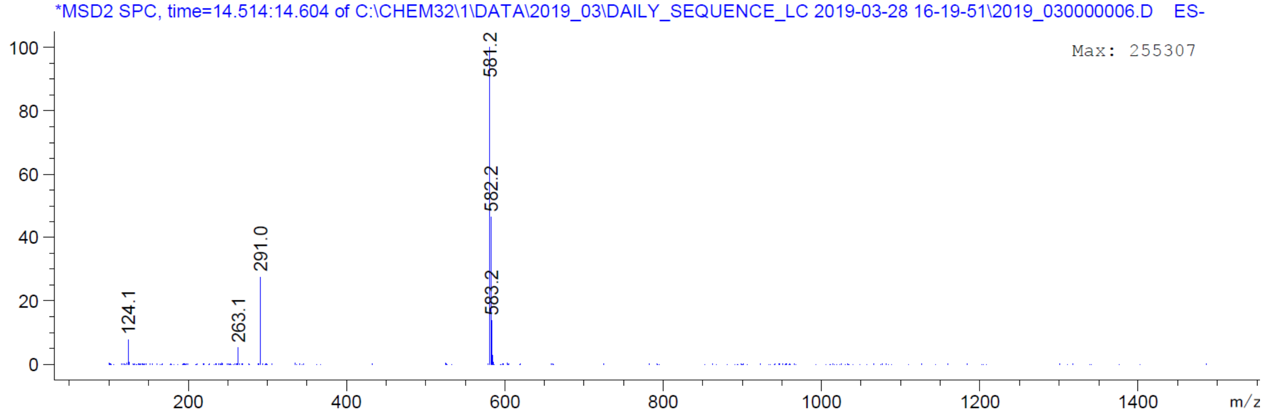
*MSD2 SPC, time=14.514:14.604 of C:\CHEM32\1\DATA\2019_03\DAILY_SEQUENCE_LC 2019-03-28 16-19-51\2019_030000006.D ES-
Max: 255307
100
80
60
40
20
0
581.2
582.2
583.2
291.0
263.1
124.1
200
400
600
800
1000
1200
1400
m/z

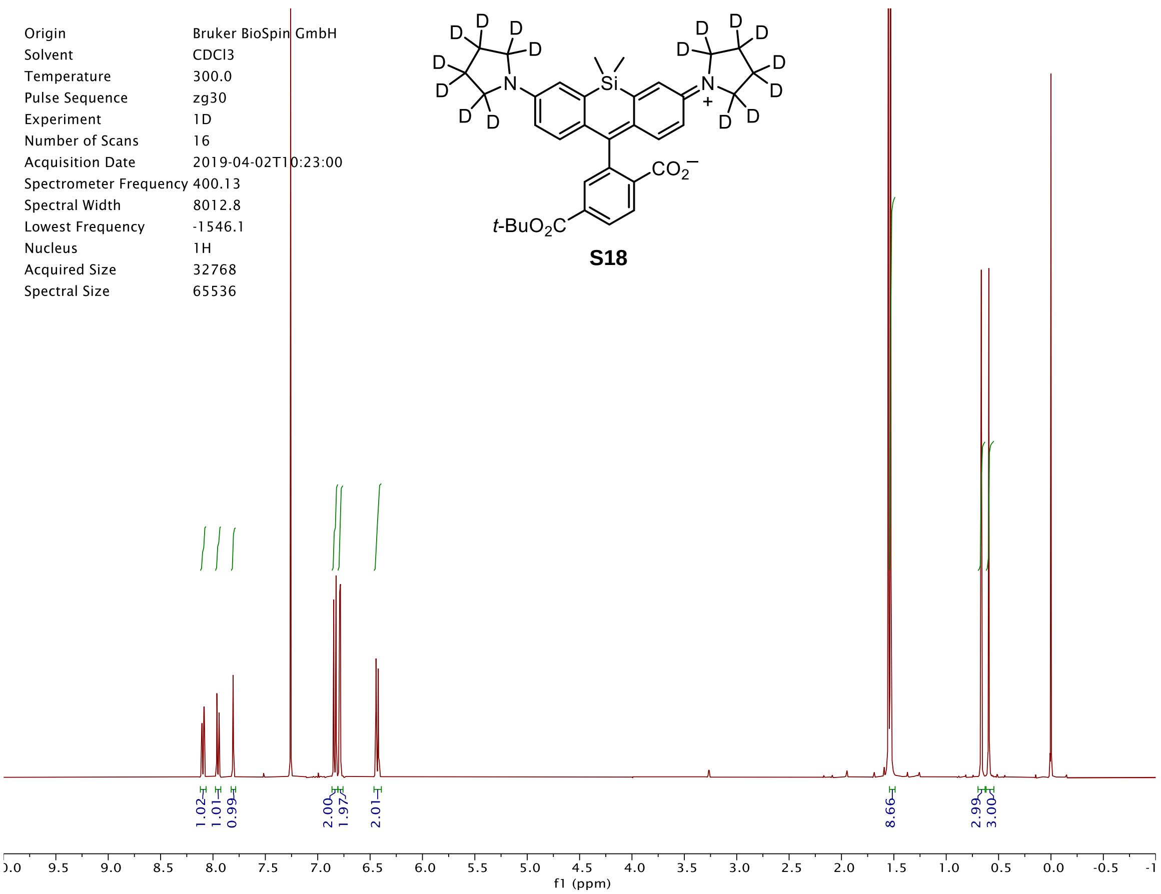

Origin	Bruker BioSpin GmbH
Solvent	CDCl3
Temperature	300.0
Pulse Sequence	zg30
Experiment	1D
Number of Scans	16
Acquisition Date	2019-04-02T10:23:00
Spectrometer Frequency	400.13
Spectral Width	8012.8
Lowest Frequency	-1546.1
Nucleus	1H
Acquired Size	32768
Spectral Size	65536

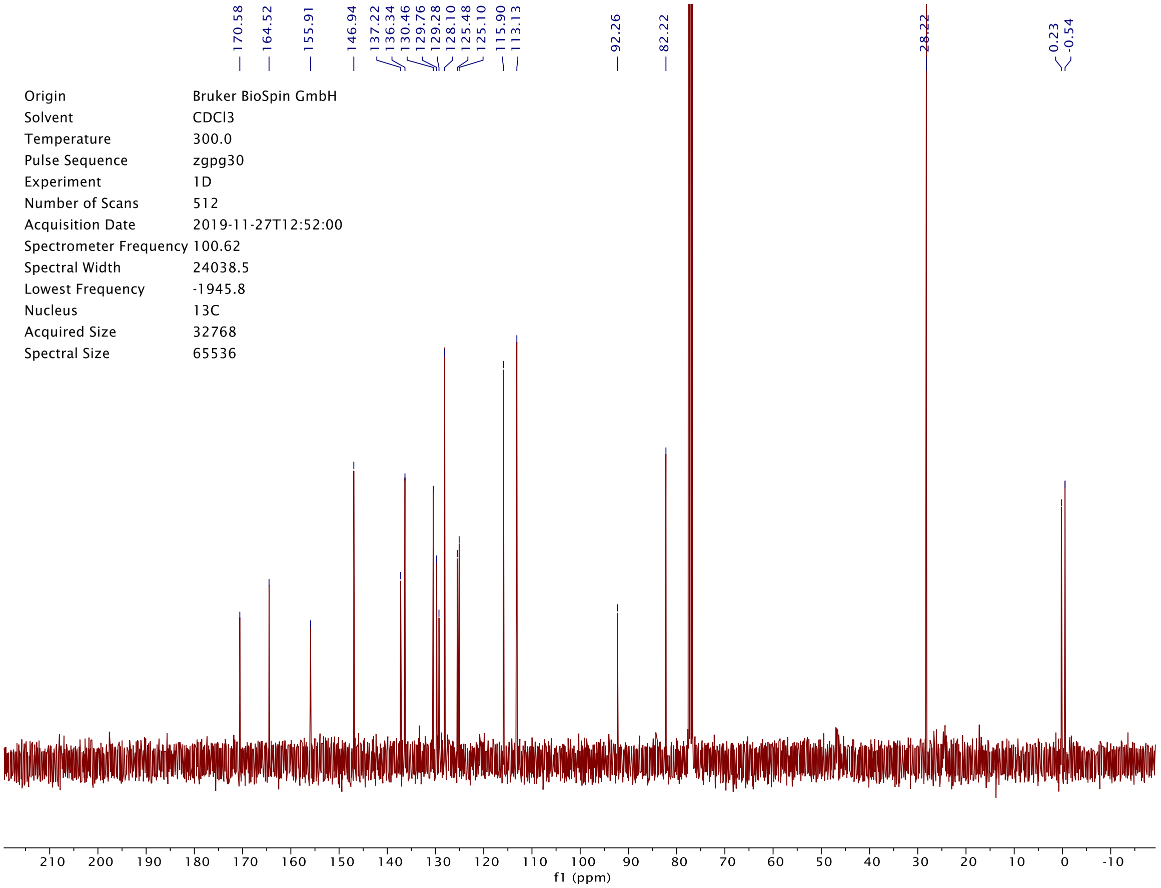

Origin	Bruker BioSpin GmbH
Solvent	CDCl3
Temperature	300.0
Pulse Sequence	zgpg30
Experiment	1D
Number of Scans	512
Acquisition Date	2019-11-27T12:52:00
Spectrometer Frequency	100.62
Spectral Width	24038.5
Lowest Frequency	-1945.8
Nucleus	13C
Acquired Size	32768
Spectral Size	65536

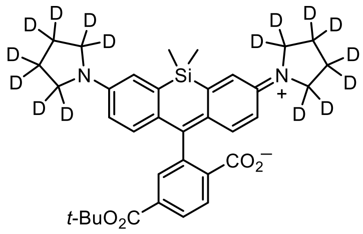

D
N
Si
CO2−
t-BuO2C

S18

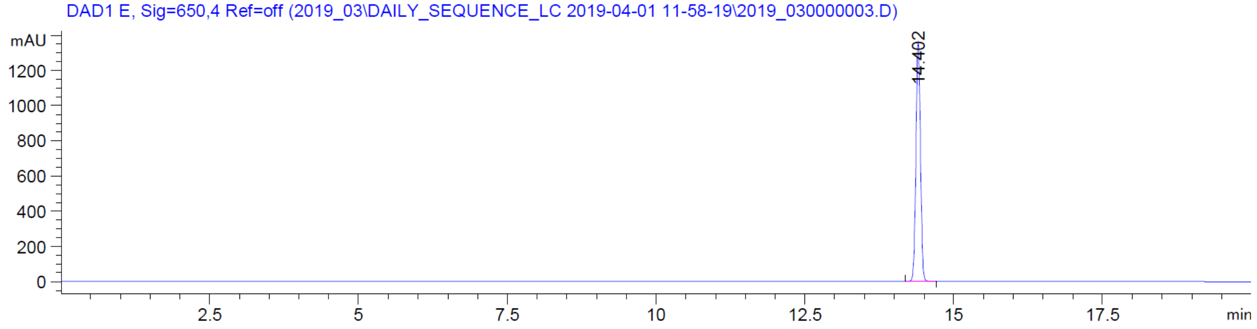

DAD1 E, Sig=650,4 Ref=off (2019_03\DAILY_SEQUENCE_LC 2019-04-01 11-58-19\2019_030000003.D)
mAU
1200
1000
800
600
400
200
0
14.402
2.5
5
7.5
10
12.5
15
17.5
min

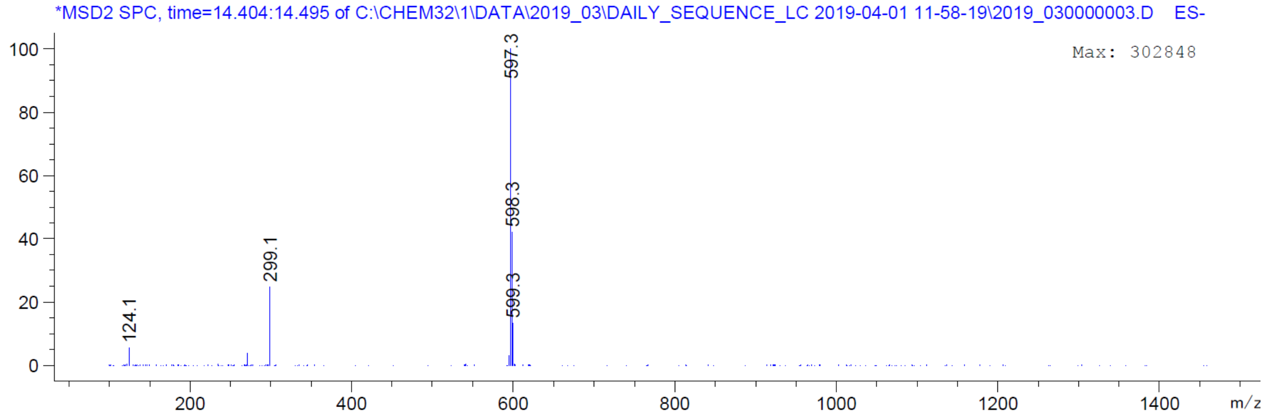

*MSD2 SPC, time=14.404:14.495 of C:\CHEM32\1\DATA\2019_03\DAILY_SEQUENCE_LC 2019-04-01 11-58-19\2019_030000003.D ES-
Max: 302848
100
80
60
40
20
0
597.3
598.3
599.3
299.1
124.1
200
400
600
800
1000
1200
1400
m/z

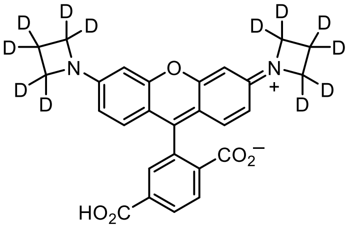

S5

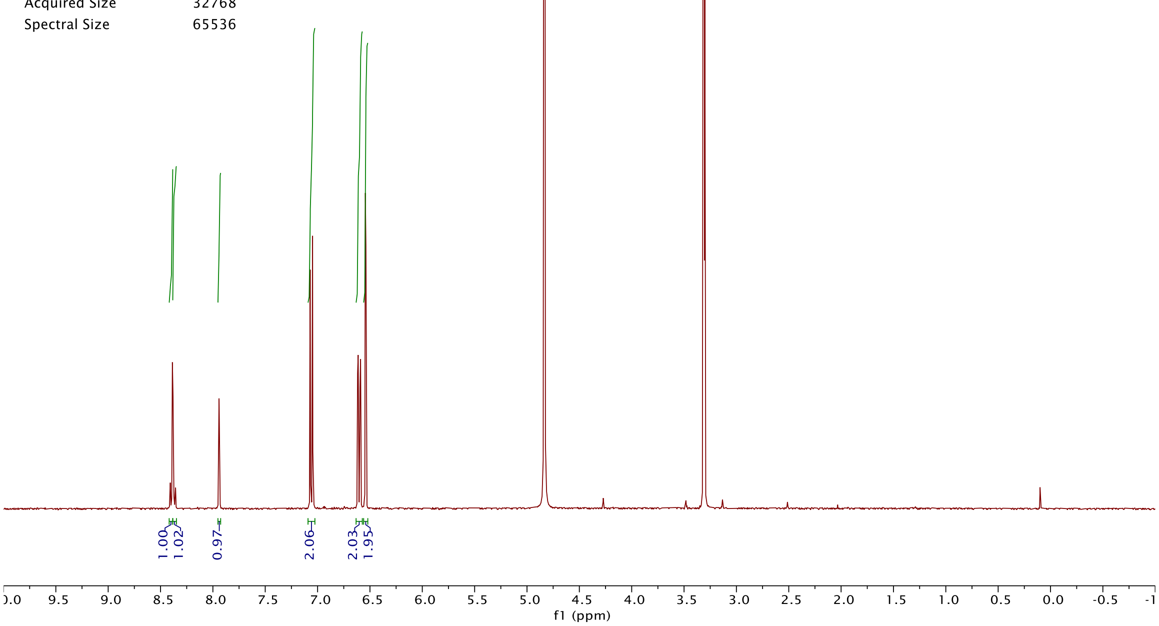

Origin Bruker BioSpin GmbH
Solvent MeOD
Temperature 300.0
Pulse Sequence zg30
Experiment 1D
Number of Scans 16
Acquisition Date 2019-03-27T11:07:00
Spectrometer Frequency 400.13
Spectral Width 8012.8
Lowest Frequency -1543.2
Nucleus 1H
Acquired Size 32768
Spectral Size 65536
1.00
1.02
0.97
2.06
2.03
1.95
f1 (ppm)

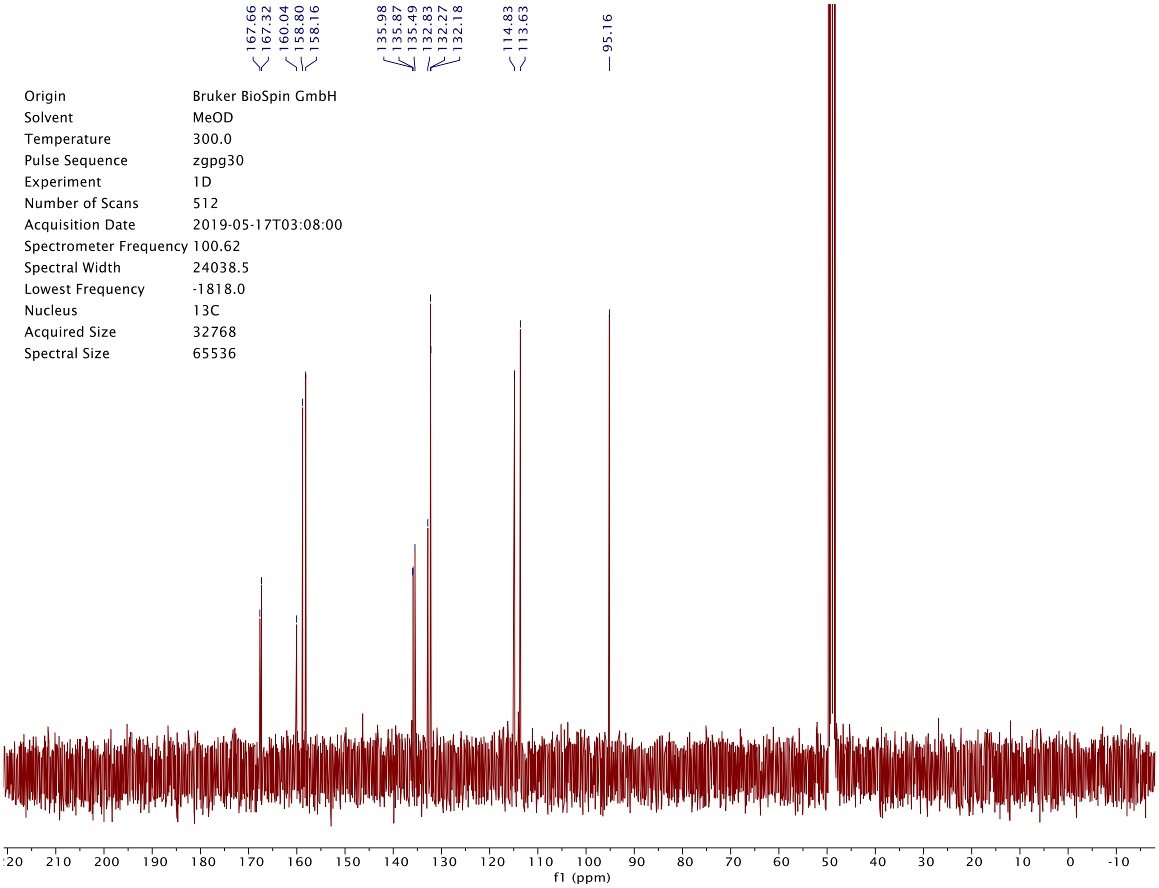

167.66
167.32
160.04
158.80
158.16
135.98
135.87
135.49
132.83
132.27
132.18
114.83
113.63
95.16
Origin Bruker BioSpin GmbH
Solvent MeOD
Temperature 300.0
Pulse Sequence zgpg30
Experiment 1D
Number of Scans 512
Acquisition Date 2019-05-17T03:08:00
Spectrometer Frequency 100.62
Spectral Width 24038.5
Lowest Frequency -1818.0
Nucleus 13C
Acquired Size 32768
Spectral Size 65536
f1 (ppm)

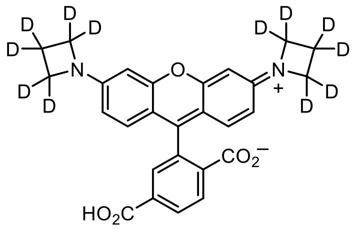

S5

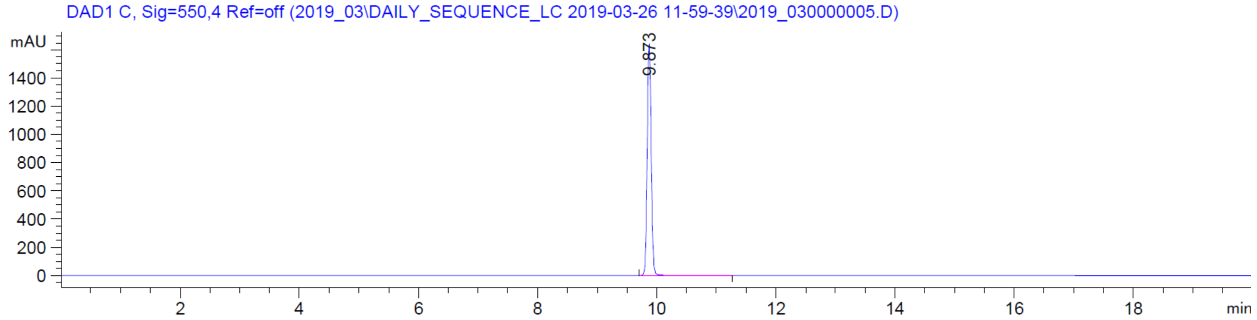
DAD1 C, Sig=550,4 Ref=off (2019_03\DAILY_SEQUENCE_LC 2019-03-26 11-59-39\2019_030000005.D)
mAU
1400
1200
1000
800
600
400
200
0
9.873
2
4
6
8
10
12
14
16
18
min

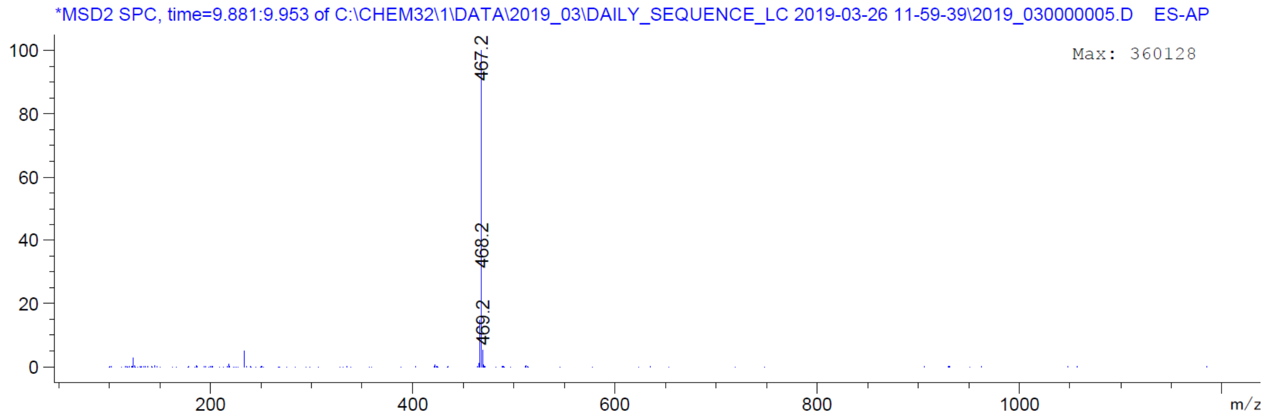
*MSD2 SPC, time=9.881:9.953 of C:\CHEM32\1\DATA\2019_03\DAILY_SEQUENCE_LC 2019-03-26 11-59-39\2019_030000005.D ES-AP
Max: 360128
100
80
60
40
20
0
467.2
468.2
469.2
200
400
600
800
1000
m/z

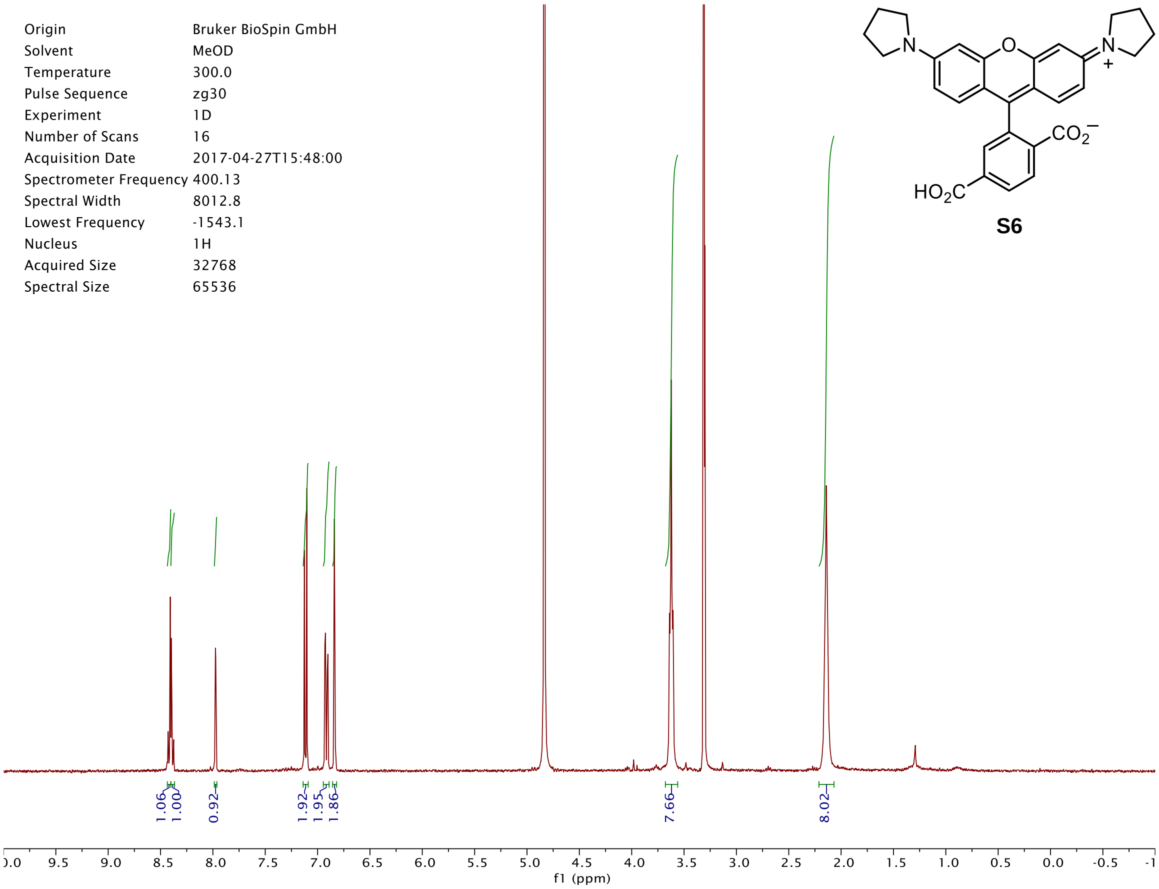

N
O
N
+
CO_2^-
HO_2C
S6
Origin Bruker BioSpin GmbH
Solvent MeOD
Temperature 300.0
Pulse Sequence zg30
Experiment 1D
Number of Scans 16
Acquisition Date 2017-04-27T15:48:00
Spectrometer Frequency 400.13
Spectral Width 8012.8
Lowest Frequency -1543.1
Nucleus 1H
Acquired Size 32768
Spectral Size 65536
1.06
1.00
0.92
1.92
1.95
1.86
7.66
8.02
f1 (ppm)

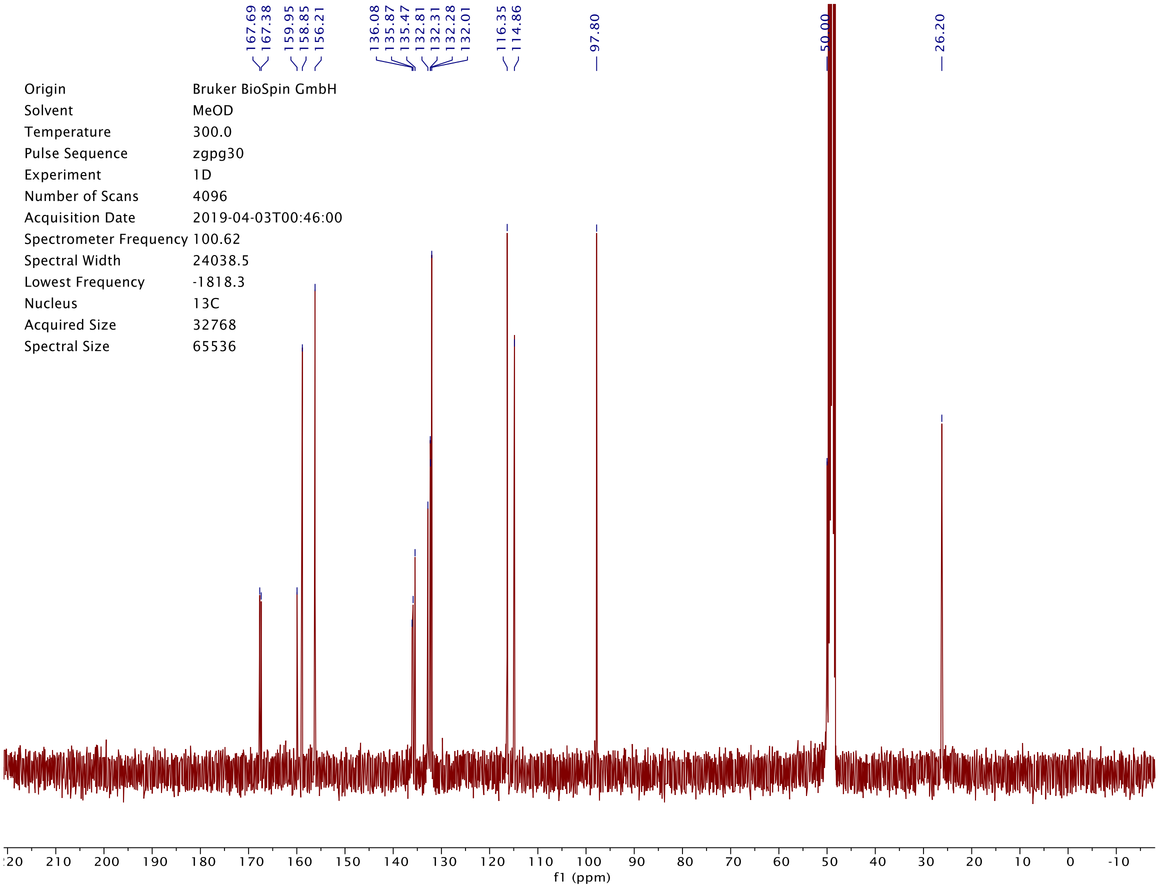

167.69
167.38
159.95
158.85
156.21
136.08
135.87
135.47
132.81
132.31
132.28
132.01
116.35
114.86
97.80
50.00
26.20
Origin Bruker BioSpin GmbH
Solvent MeOD
Temperature 300.0
Pulse Sequence zgpg30
Experiment 1D
Number of Scans 4096
Acquisition Date 2019-04-03T00:46:00
Spectrometer Frequency 100.62
Spectral Width 24038.5
Lowest Frequency -1818.3
Nucleus 13C
Acquired Size 32768
Spectral Size 65536
f1 (ppm)

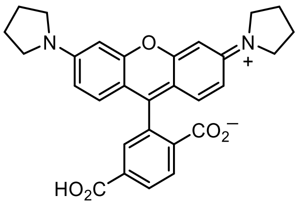

S6

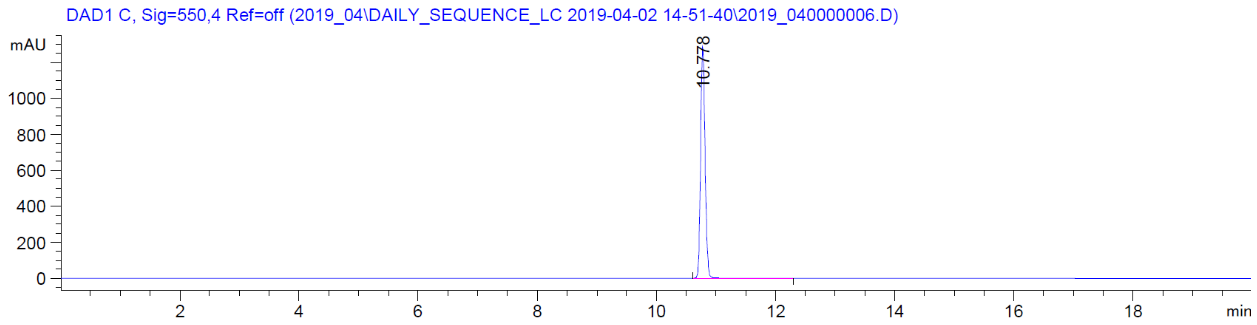
DAD1 C, Sig=550,4 Ref=off (2019_04\DAILY_SEQUENCE_LC 2019-04-02 14-51-40\2019_040000006.D)
mAU
1000
800
600
400
200
0
10.778
2
4
6
8
10
12
14
16
18
min

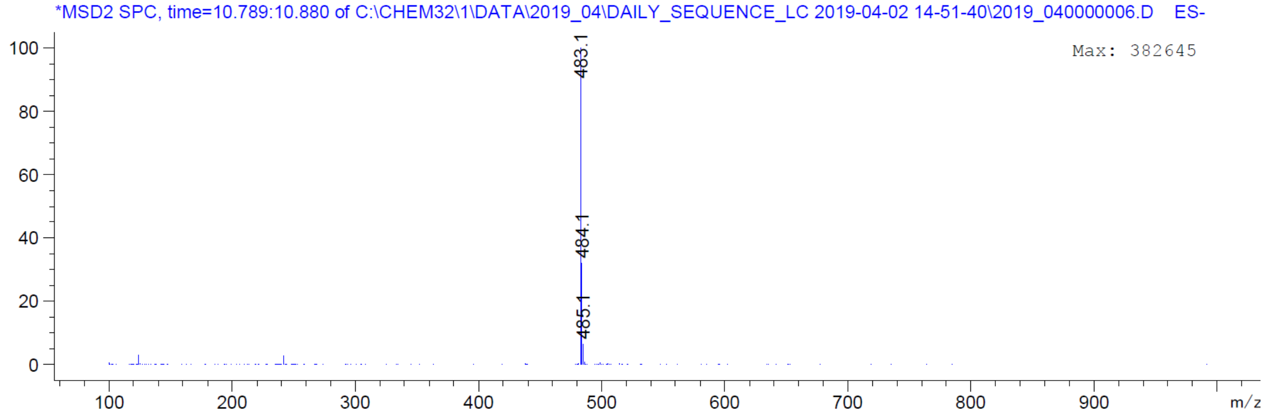
*MSD2 SPC, time=10.789:10.880 of C:\CHEM32\1\DATA\2019_04\DAILY_SEQUENCE_LC 2019-04-02 14-51-40\2019_040000006.D ES-
Max: 382645
100
80
60
40
20
0
483.1
484.1
485.1
100
200
300
400
500
600
700
800
900
m/z

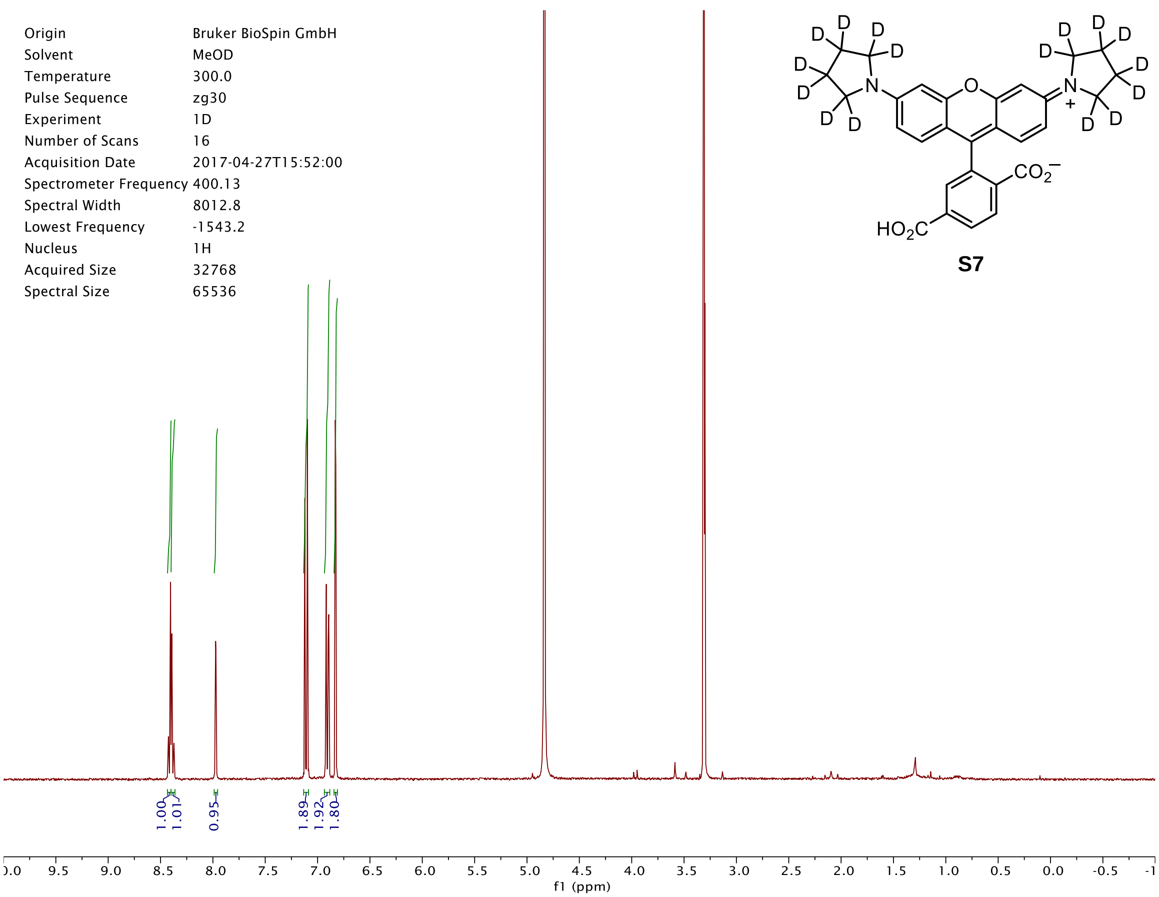
Origin Bruker BioSpin GmbH
Solvent MeOD
Temperature 300.0
Pulse Sequence zg30
Experiment 1D
Number of Scans 16
Acquisition Date 2017-04-27T15:52:00
Spectrometer Frequency 400.13
Spectral Width 8012.8
Lowest Frequency -1543.2
Nucleus 1H
Acquired Size 32768
Spectral Size 65536
HO2C
CO2−
N
O
D
S7
1.00
1.01
0.95
1.89
1.92
1.80
f1 (ppm)

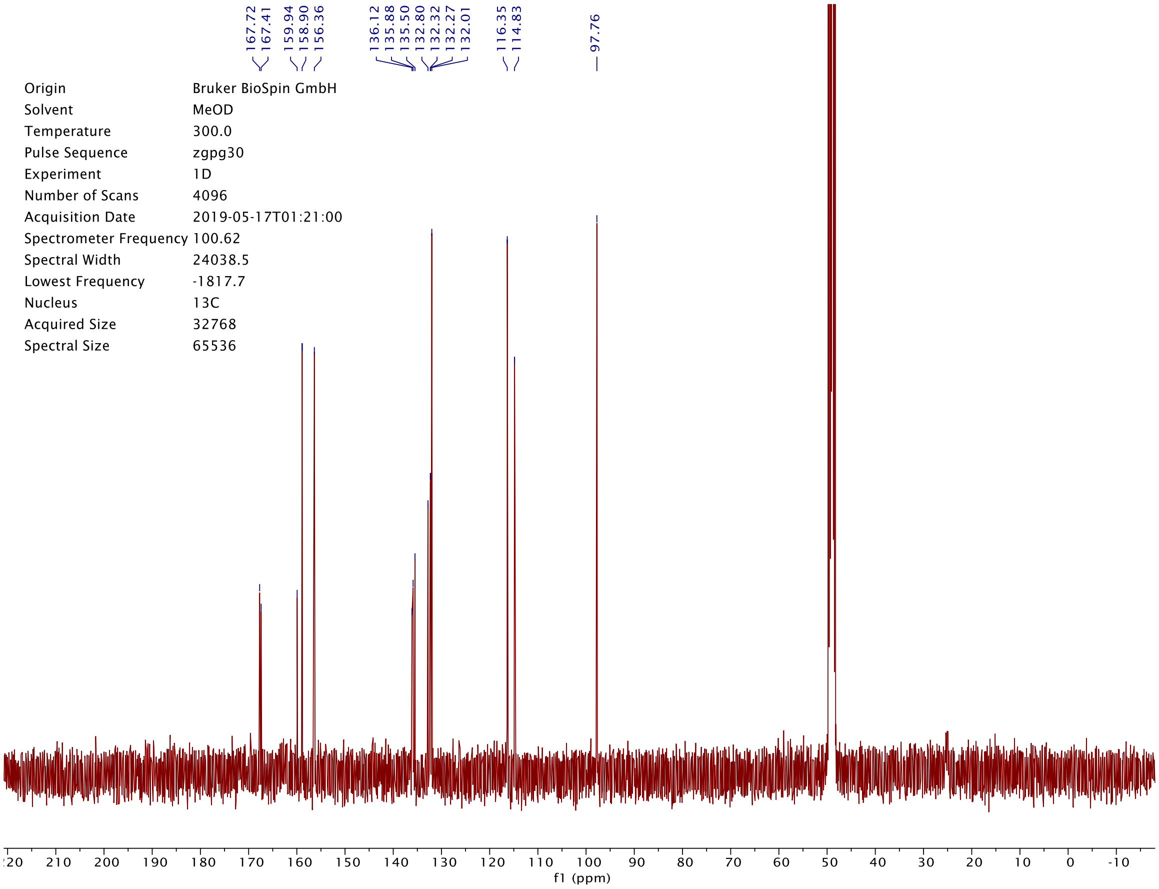
167.72
167.41
159.94
158.90
156.36
136.12
135.88
135.50
132.80
132.32
132.27
132.01
116.35
114.83
97.76
Origin Bruker BioSpin GmbH
Solvent MeOD
Temperature 300.0
Pulse Sequence zgpg30
Experiment 1D
Number of Scans 4096
Acquisition Date 2019-05-17T01:21:00
Spectrometer Frequency 100.62
Spectral Width 24038.5
Lowest Frequency -1817.7
Nucleus 13C
Acquired Size 32768
Spectral Size 65536
f1 (ppm)

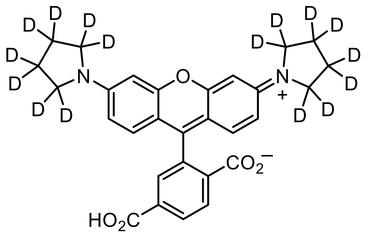

S7

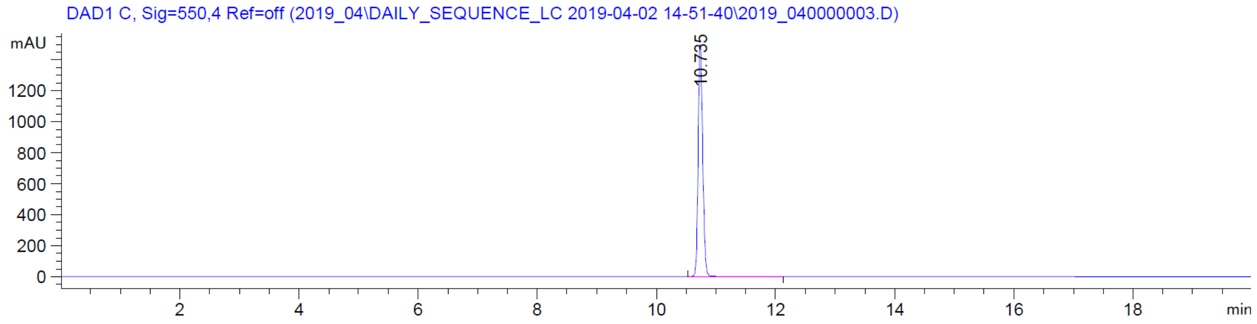

DAD1 C, Sig=550,4 Ref=off (2019_04\DAILY_SEQUENCE_LC 2019-04-02 14-51-40\2019_040000003.D)
mAU
10.735
min

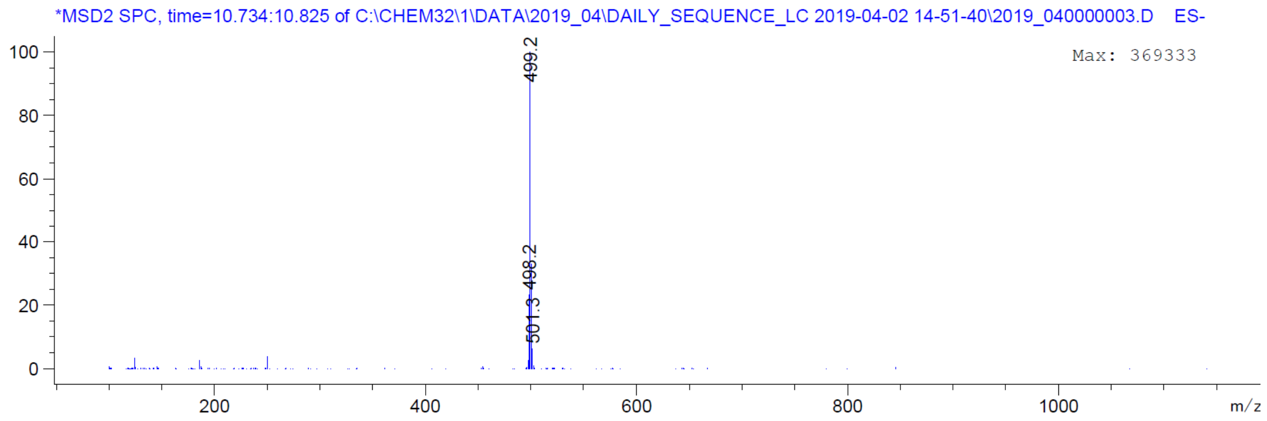

*MSD2 SPC, time=10.734:10.825 of C:\CHEM32\1\DATA\2019_04\DAILY_SEQUENCE_LC 2019-04-02 14-51-40\2019_040000003.D ES-
Max: 369333
499.2
498.2
501.3
m/z

Origin	Bruker BioSpin GmbH
Solvent	MeOD
Temperature	300.0
Pulse Sequence	zg30
Experiment	1D
Number of Scans	16
Acquisition Date	2019-03-25T15:36:00
Spectrometer Frequency	400.13
Spectral Width	8012.8
Lowest Frequency	-1543.2
Nucleus	1H
Acquired Size	32768
Spectral Size	65536

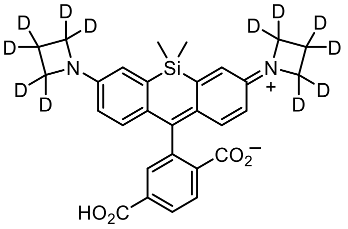

S19

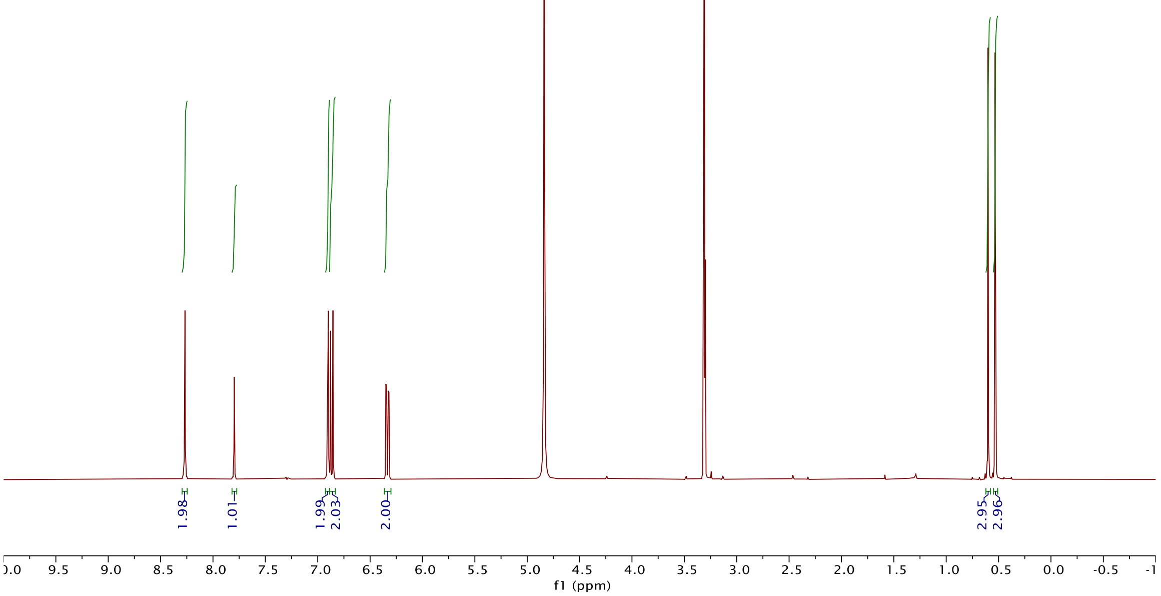

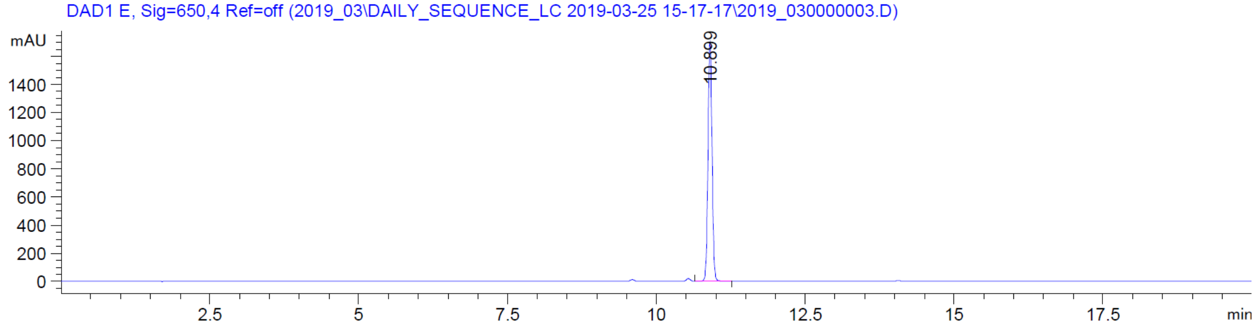

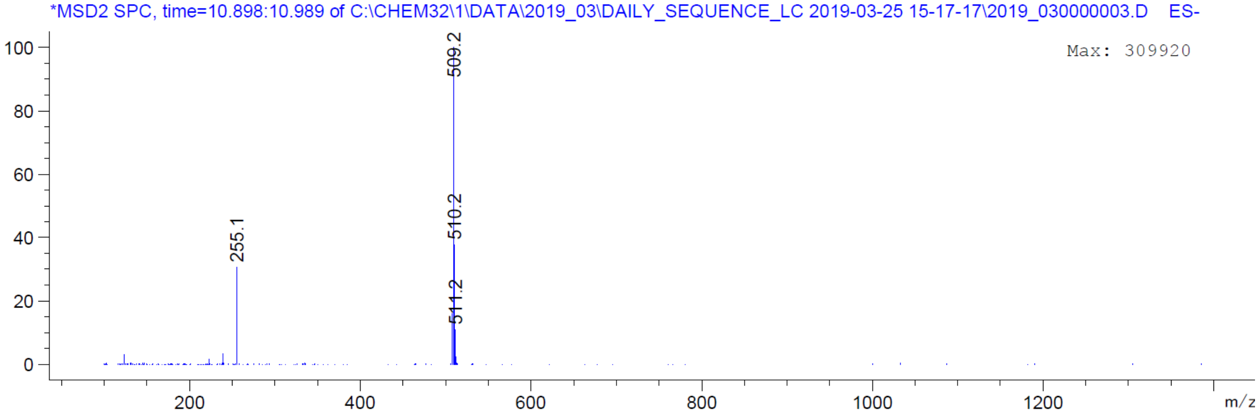

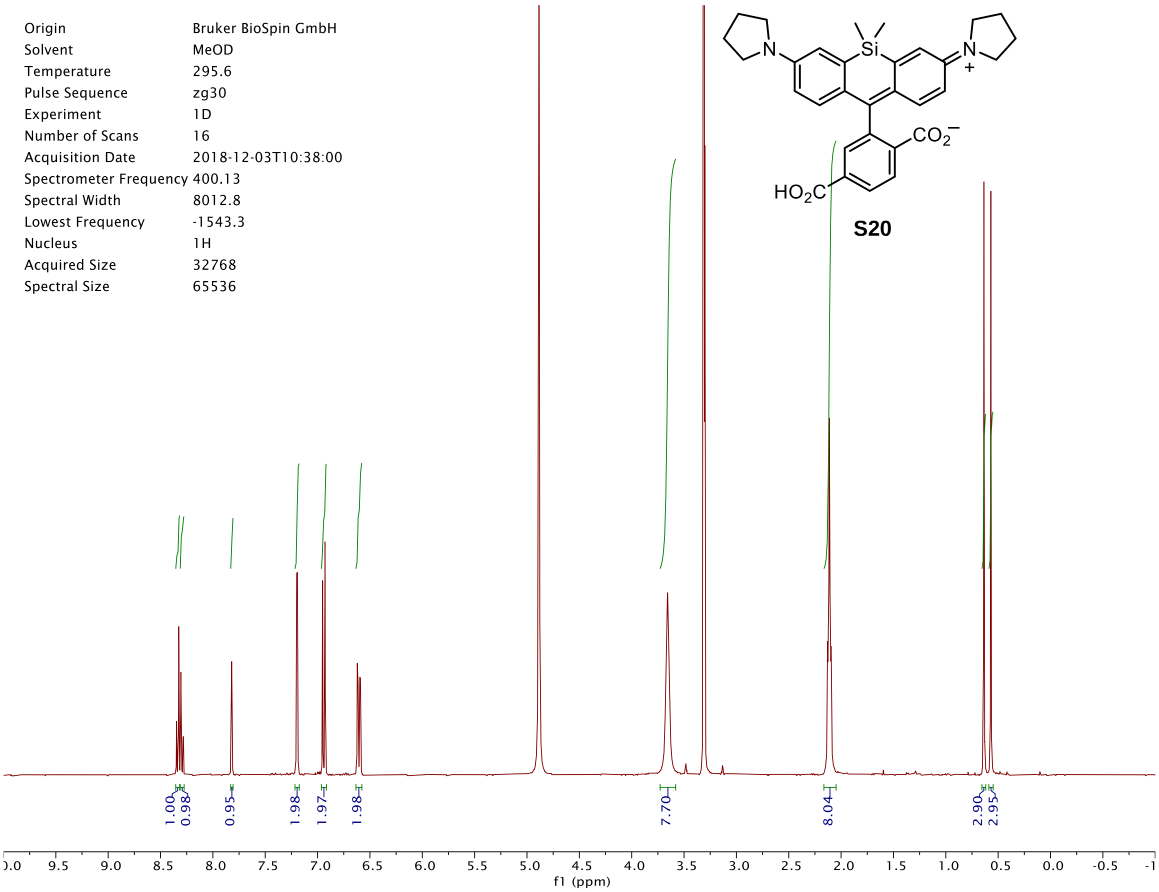
Origin Bruker BioSpin GmbH
Solvent MeOD
Temperature 295.6
Pulse Sequence zg30
Experiment 1D
Number of Scans 16
Acquisition Date 2018-12-03T10:38:00
Spectrometer Frequency 400.13
Spectral Width 8012.8
Lowest Frequency -1543.3
Nucleus 1H
Acquired Size 32768
Spectral Size 65536
S20
1.00
0.98
0.95
1.98
1.97
1.98
7.70
8.04
2.90
2.95
f1 (ppm)

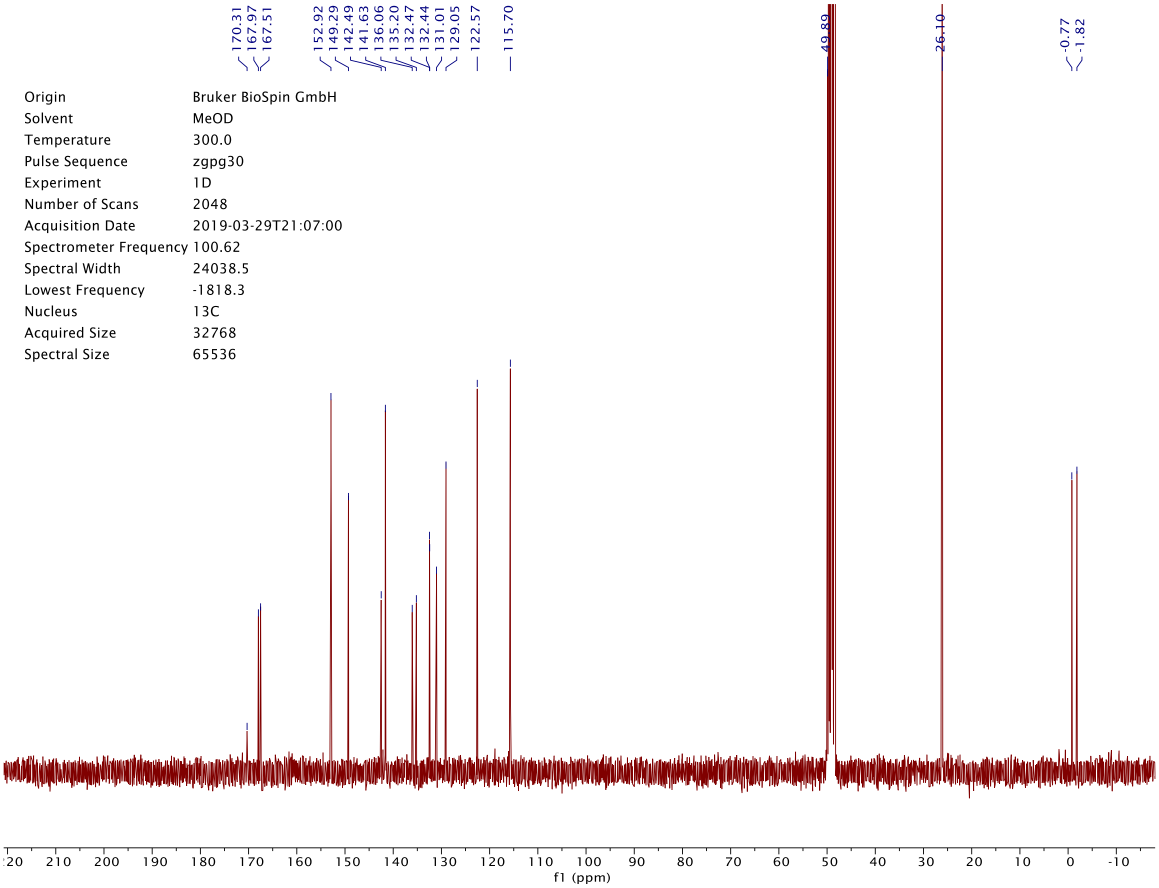
170.31
167.97
167.51
152.92
149.29
142.49
141.63
136.06
135.20
132.47
132.44
131.01
129.05
122.57
115.70
49.89
26.10
-0.77
-1.82
Origin Bruker BioSpin GmbH
Solvent MeOD
Temperature 300.0
Pulse Sequence zgpg30
Experiment 1D
Number of Scans 2048
Acquisition Date 2019-03-29T21:07:00
Spectrometer Frequency 100.62
Spectral Width 24038.5
Lowest Frequency -1818.3
Nucleus 13C
Acquired Size 32768
Spectral Size 65536
f1 (ppm)

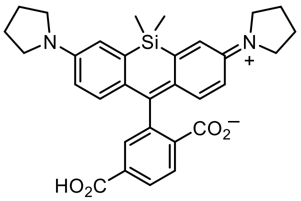

S20

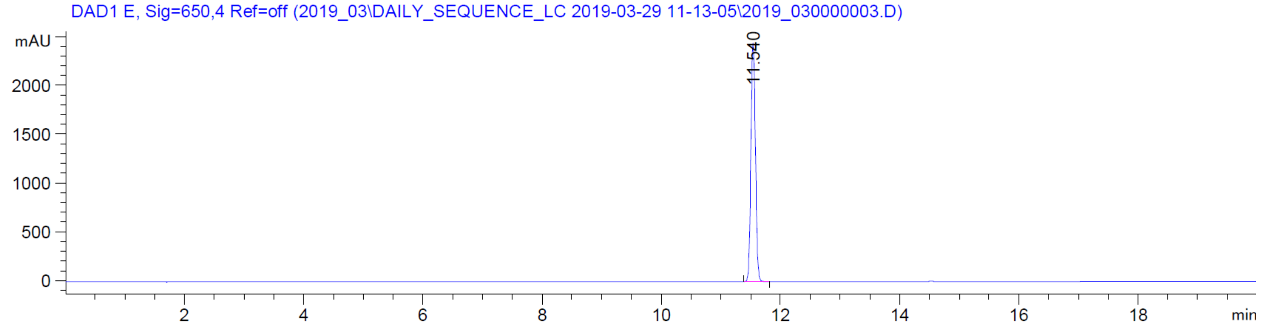

DAD1 E, Sig=650,4 Ref=off (2019_03\DAILY_SEQUENCE_LC 2019-03-29 11-13-05\2019_030000003.D)
mAU
11.540
min

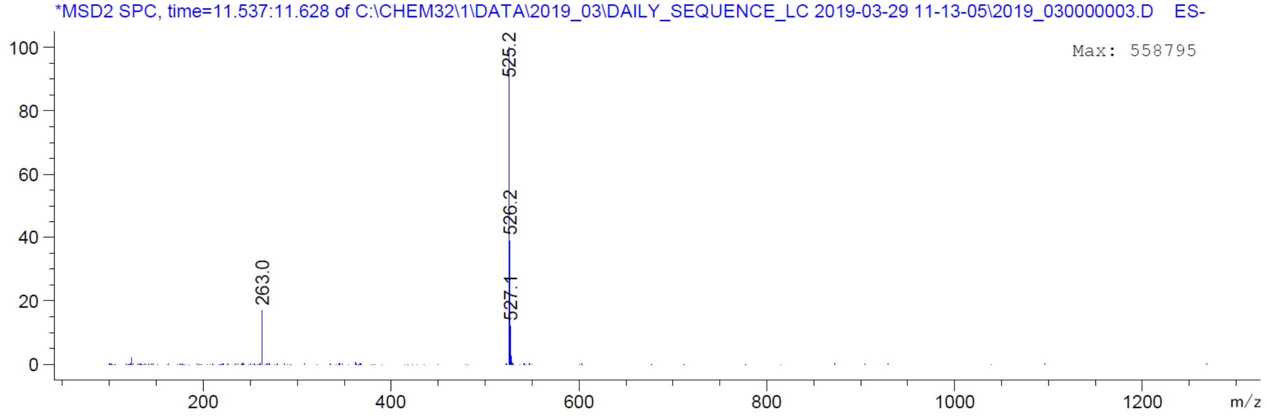

*MSD2 SPC, time=11.537:11.628 of C:\CHEM32\1\DATA\2019_03\DAILY_SEQUENCE_LC 2019-03-29 11-13-05\2019_030000003.D ES-
Max: 558795
525.2
526.2
527.1
263.0
m/z

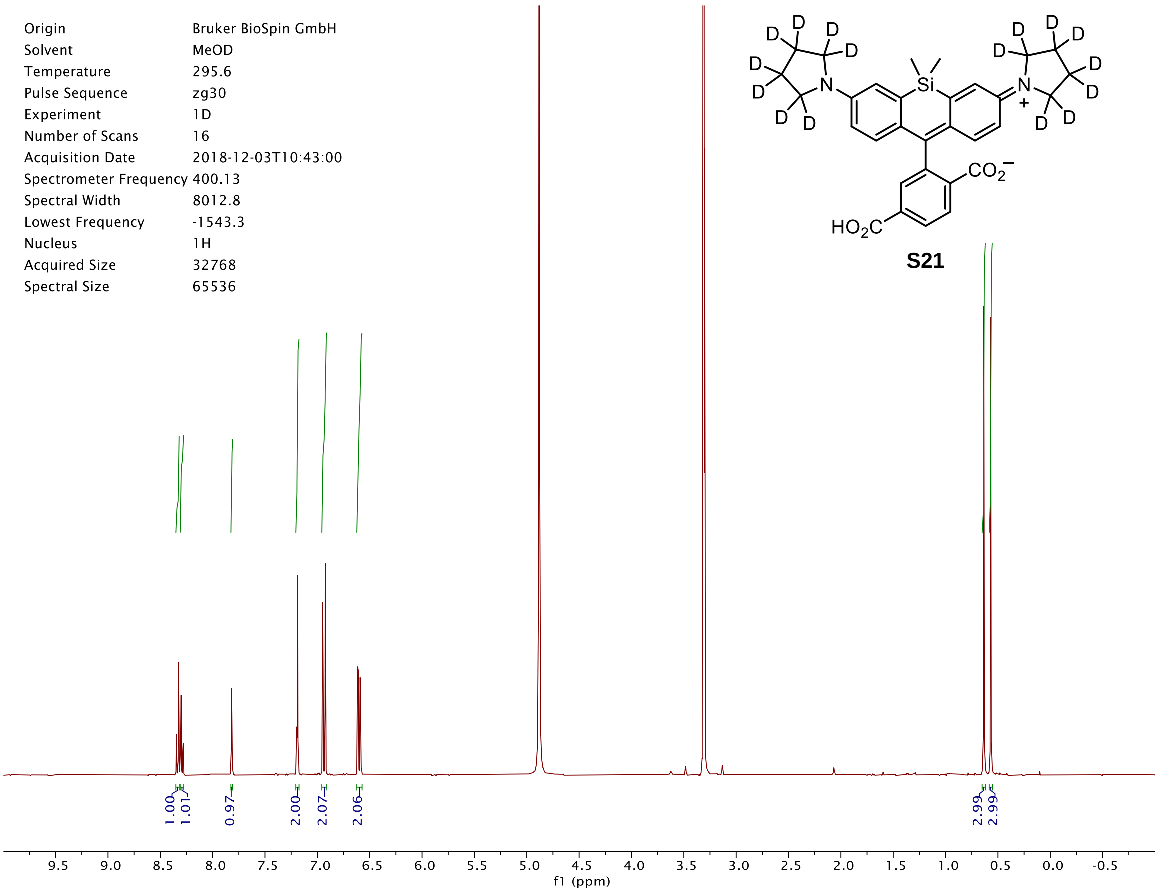
Origin Bruker BioSpin GmbH
Solvent MeOD
Temperature 295.6
Pulse Sequence zg30
Experiment 1D
Number of Scans 16
Acquisition Date 2018-12-03T10:43:00
Spectrometer Frequency 400.13
Spectral Width 8012.8
Lowest Frequency -1543.3
Nucleus 1H
Acquired Size 32768
Spectral Size 65536
1.00
1.01
0.97
2.00
2.07
2.06
2.99
2.99
f1 (ppm)

S21

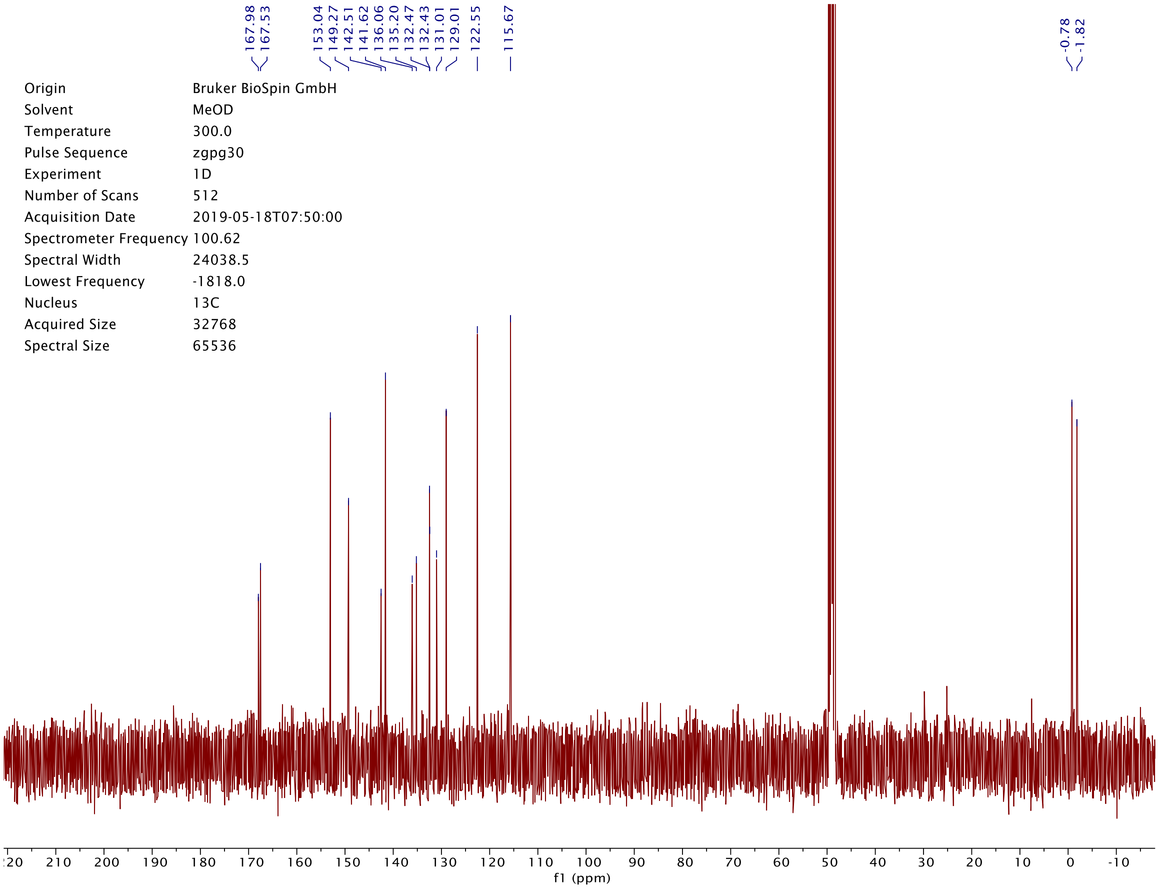
167.98
167.53
153.04
149.27
142.51
141.62
136.06
135.20
132.47
132.43
131.01
129.01
122.55
115.67
-0.78
-1.82
Origin Bruker BioSpin GmbH
Solvent MeOD
Temperature 300.0
Pulse Sequence zgpg30
Experiment 1D
Number of Scans 512
Acquisition Date 2019-05-18T07:50:00
Spectrometer Frequency 100.62
Spectral Width 24038.5
Lowest Frequency -1818.0
Nucleus 13C
Acquired Size 32768
Spectral Size 65536
f1 (ppm)

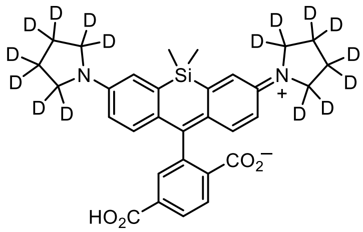
D
N
Si
N
+
CO_2^-
HO_2C

S21

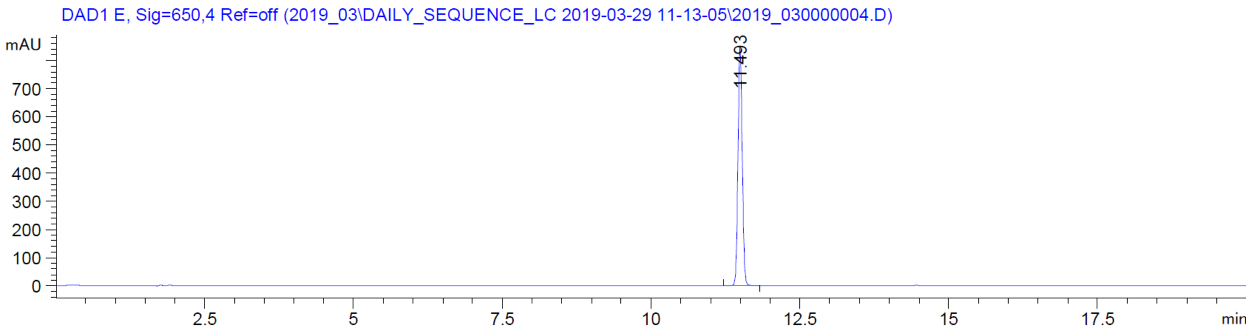
DAD1 E, Sig=650,4 Ref=off (2019_03\DAILY_SEQUENCE_LC 2019-03-29 11-13-05\2019_030000004.D)
mAU
700
600
500
400
300
200
100
0
11.493
2.5
5
7.5
10
12.5
15
17.5
min

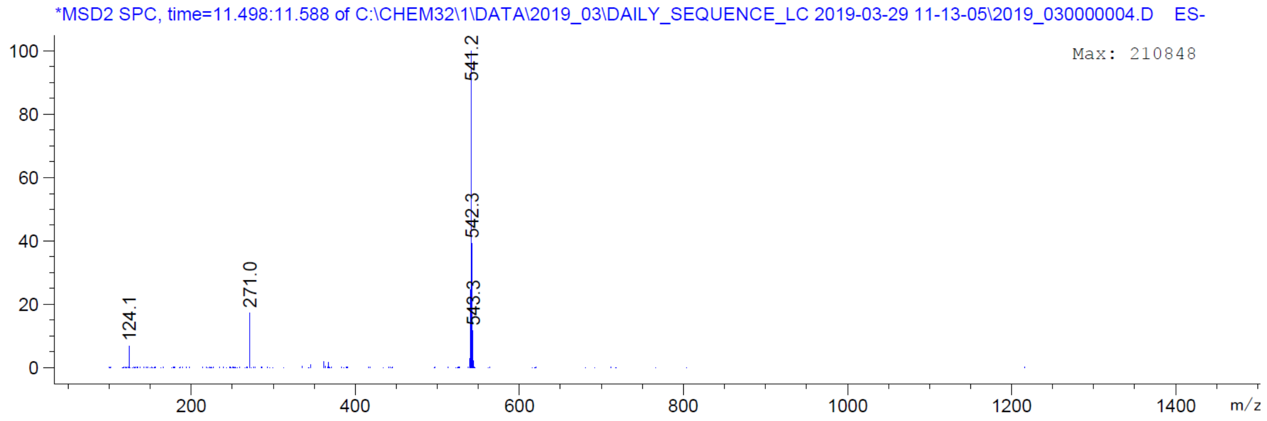
*MSD2 SPC, time=11.498:11.588 of C:\CHEM32\1\DATA\2019_03\DAILY_SEQUENCE_LC 2019-03-29 11-13-05\2019_030000004.D ES-
Max: 210848
100
80
60
40
20
0
541.2
542.3
543.3
271.0
124.1
200
400
600
800
1000
1200
1400
m/z

Origin	Bruker BioSpin GmbH
Solvent	CDCl3
Temperature	300.0
Pulse Sequence	zg30
Experiment	1D
Number of Scans	16
Acquisition Date	2019-04-16T14:57:00
Spectrometer Frequency	400.13
Spectral Width	8012.8
Lowest Frequency	-1545.8
Nucleus	1H
Acquired Size	32768
Spectral Size	65536

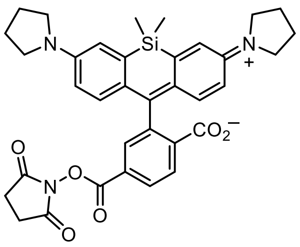

S22

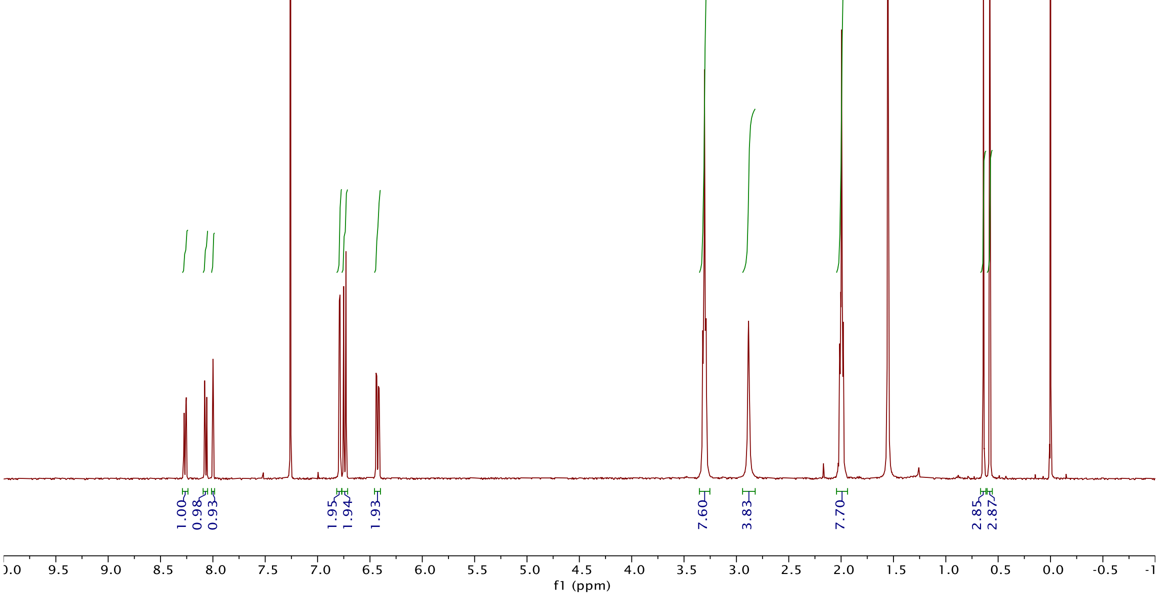

Origin	Bruker BioSpin GmbH
Solvent	CDCl3
Temperature	300.0
Pulse Sequence	zgpg30
Experiment	1D
Number of Scans	2048
Acquisition Date	2019-04-16T21:10:00
Spectrometer Frequency	100.62
Spectral Width	24038.5
Lowest Frequency	-1946.9
Nucleus	13C
Acquired Size	32768
Spectral Size	65536

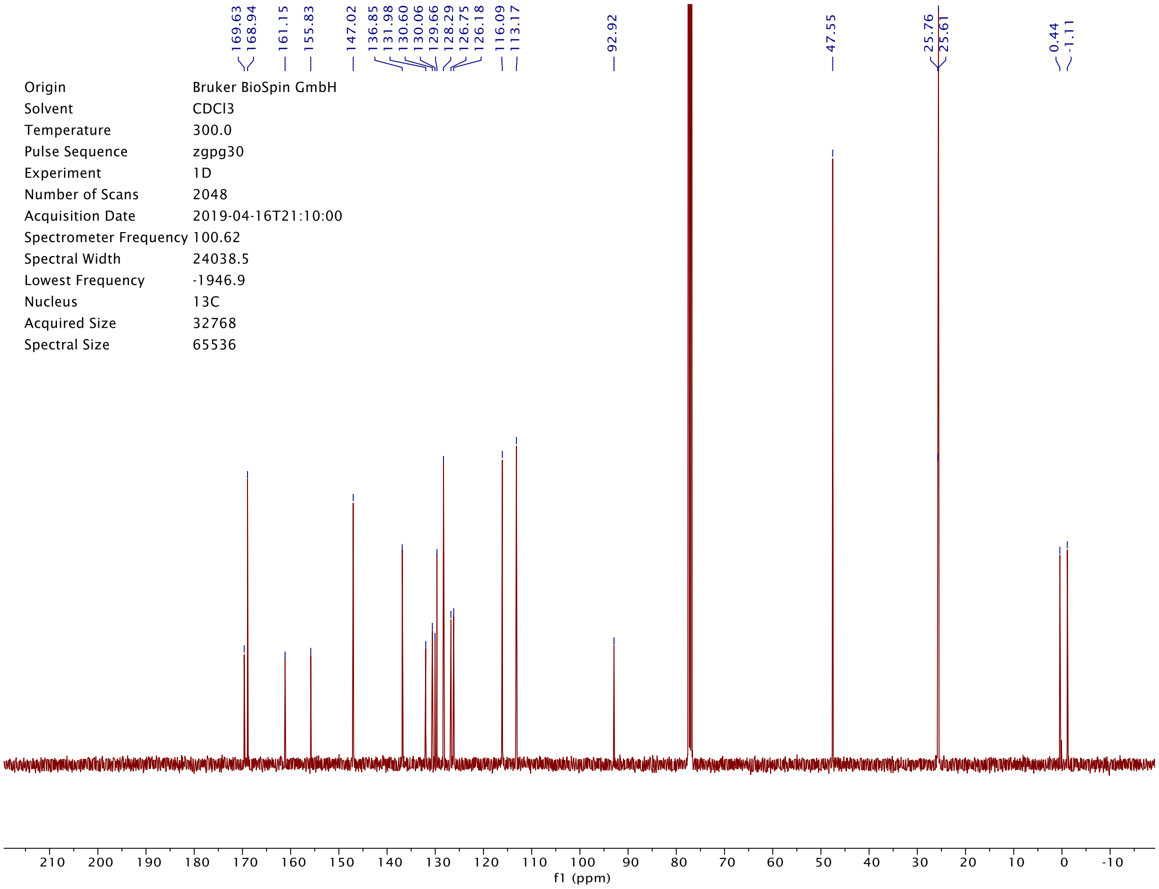

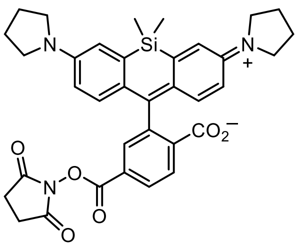

S22

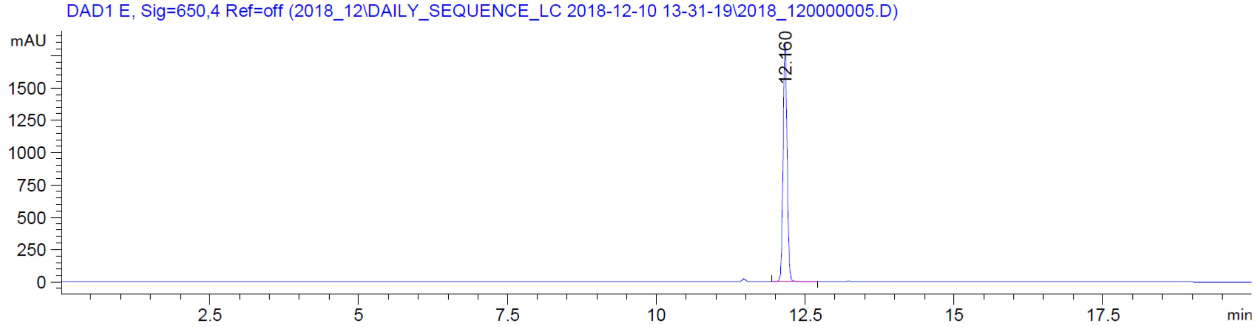
DAD1 E, Sig=650,4 Ref=off (2018_12\DAILY_SEQUENCE_LC 2018-12-10 13-31-19\2018_120000005.D)
mAU
1500
1250
1000
750
500
250
0
12.160
2.5
5
7.5
10
12.5
15
17.5
min

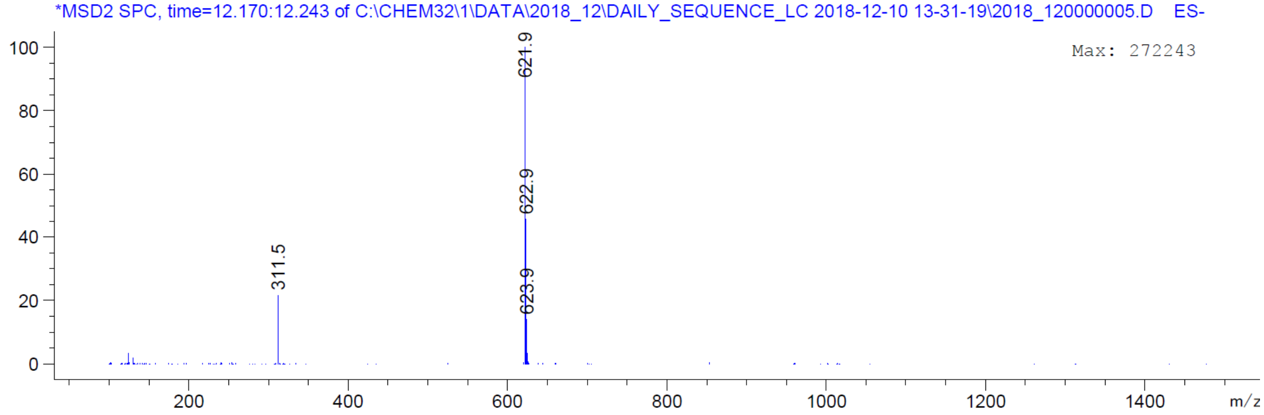
*MSD2 SPC, time=12.170:12.243 of C:\CHEM32\1\DATA\2018_12\DAILY_SEQUENCE_LC 2018-12-10 13-31-19\2018_120000005.D ES-
Max: 272243
100
80
60
40
20
0
621.9
622.9
623.9
311.5
200
400
600
800
1000
1200
1400
m/z

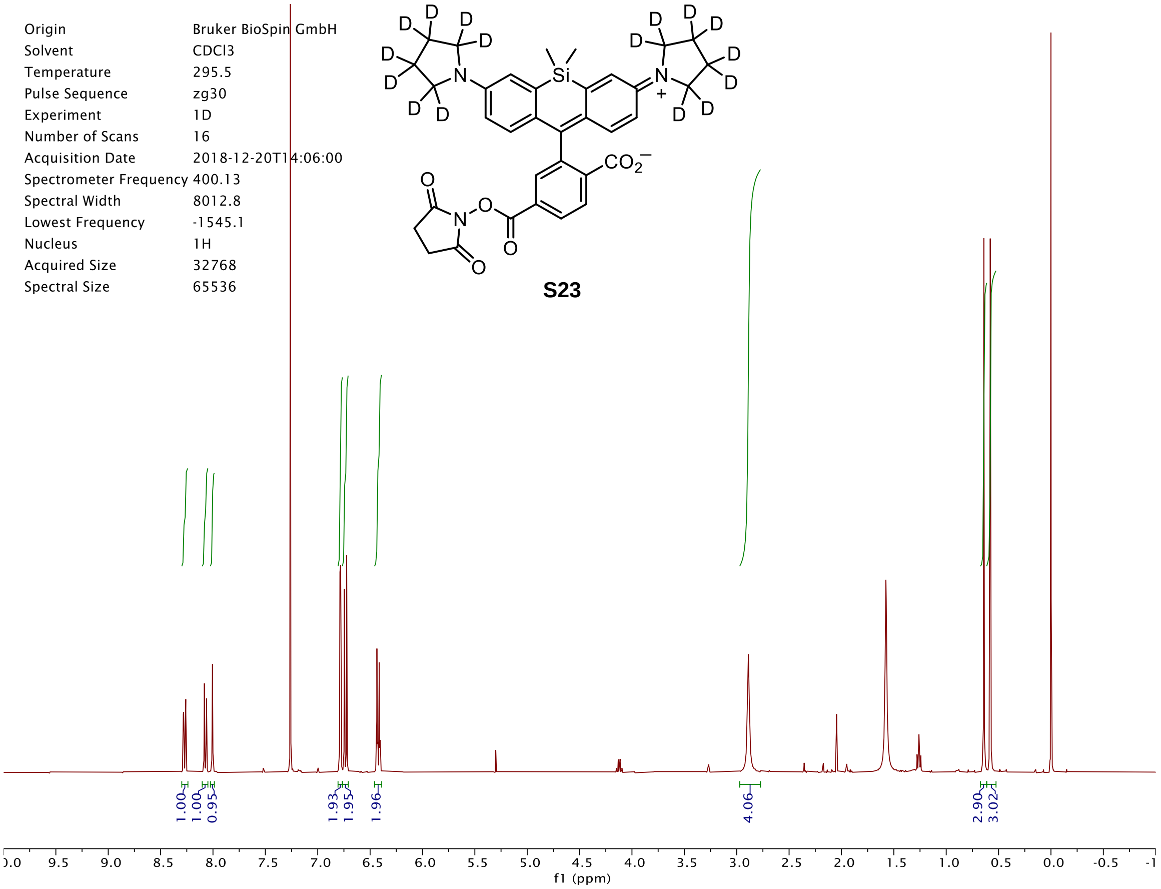
Origin Bruker BioSpin GmbH
Solvent CDCl3
Temperature 295.5
Pulse Sequence zg30
Experiment 1D
Number of Scans 16
Acquisition Date 2018-12-20T14:06:00
Spectrometer Frequency 400.13
Spectral Width 8012.8
Lowest Frequency -1545.1
Nucleus 1H
Acquired Size 32768
Spectral Size 65536
S23
1.00
1.00
0.95
1.93
1.95
1.96
4.06
2.90
3.02
f1 (ppm)

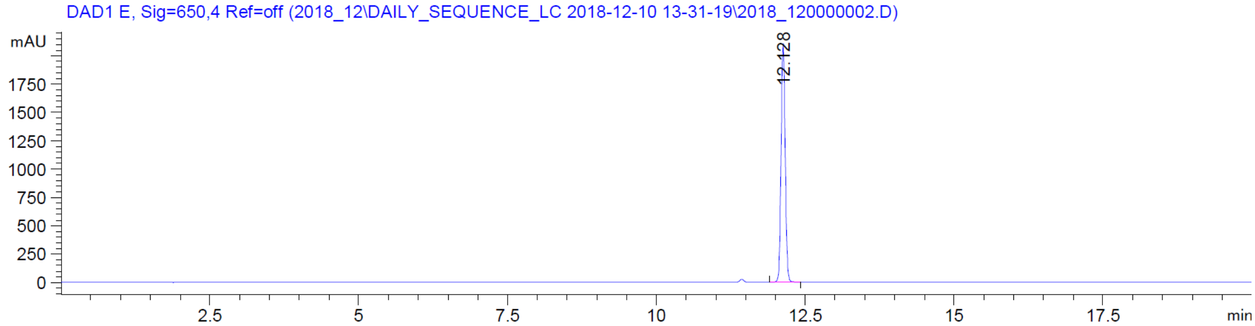
DAD1 E, Sig=650,4 Ref=off (2018_12\DAILY_SEQUENCE_LC 2018-12-10 13-31-19\2018_120000002.D)
mAU
12.128
min

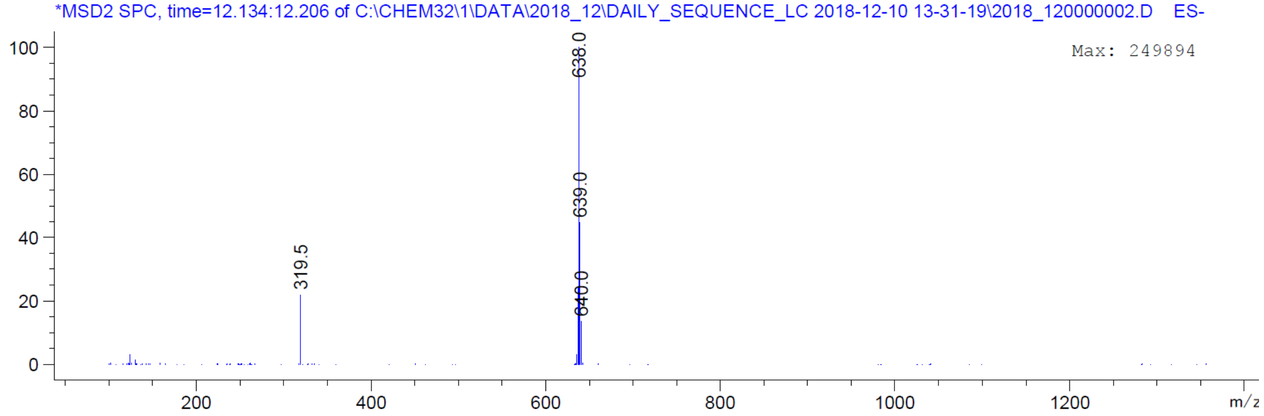
*MSD2 SPC, time=12.134:12.206 of C:\CHEM32\1\DATA\2018_12\DAILY_SEQUENCE_LC 2018-12-10 13-31-19\2018_120000002.D ES-
Max: 249894
638.0
639.0
640.0
319.5
m/z

Origin	Bruker BioSpin GmbH
Solvent	MeOD
Temperature	295.6
Pulse Sequence	zg30
Experiment	1D
Number of Scans	16
Acquisition Date	2017-10-18T11:20:00
Spectrometer Frequency	400.13
Spectral Width	8012.8
Lowest Frequency	-1543.2
Nucleus	1H
Acquired Size	32768
Spectral Size	65536

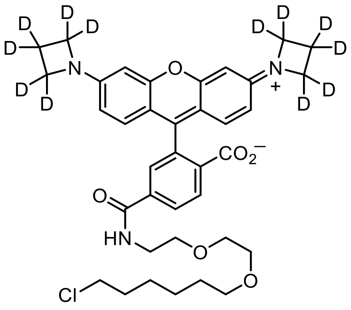

20_D

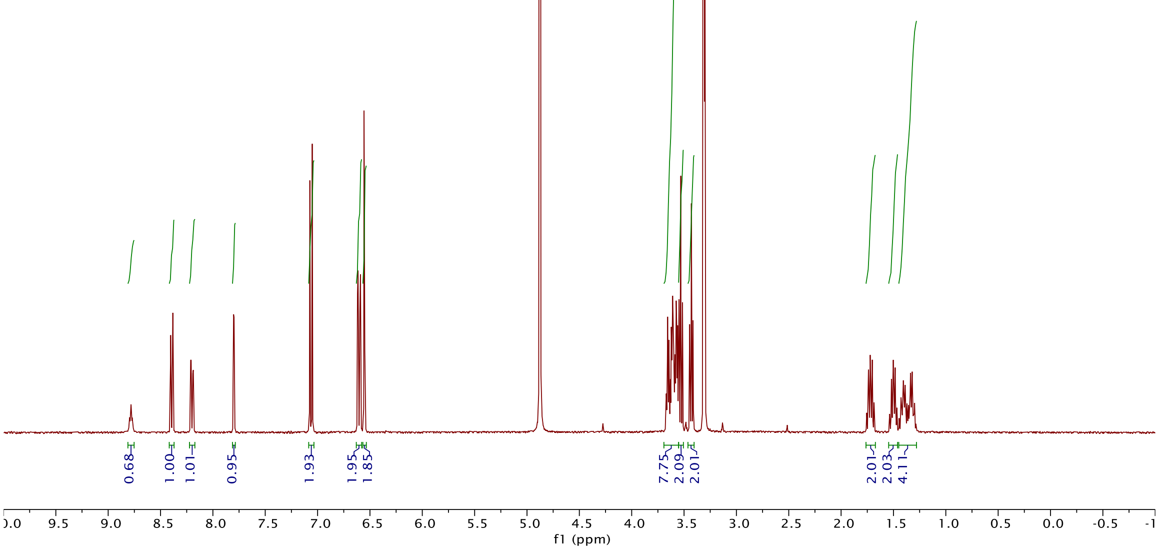

DAD1 C, Sig=550,4 Ref=off (2017_10\DAILY_SEQUENCE_LC 2017-10-17 15-38-37\2017_100000002.D)

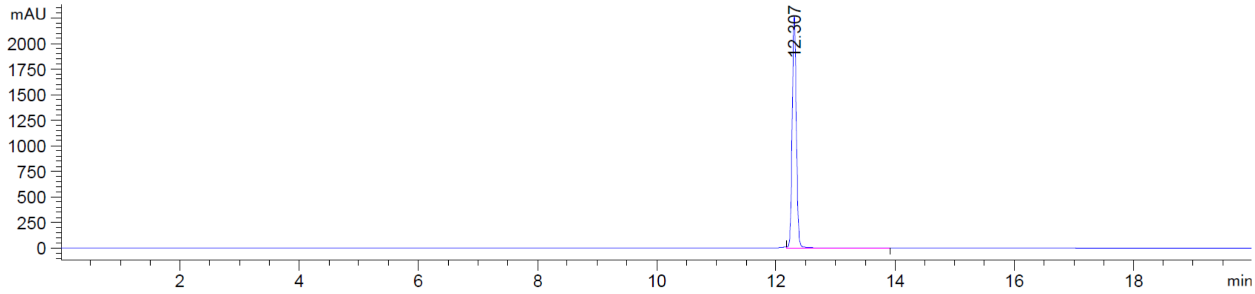

*MSD2 SPC, time=12.300:12.373 of C:\CHEM32\1\DATA\2017_10\DAILY_SEQUENCE_LC 2017-10-17 15-38-37\2017_100000002.D ES-

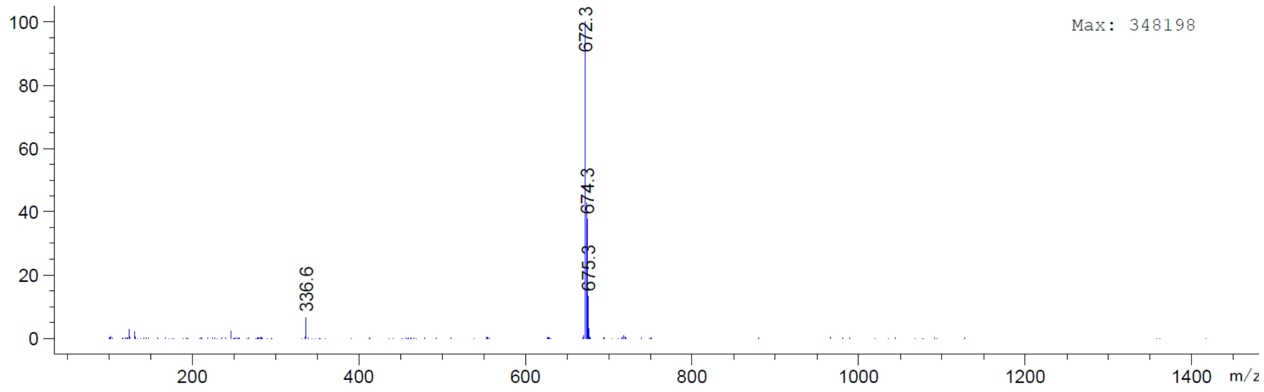

Origin	Bruker BioSpin GmbH
Solvent	MeOD
Temperature	300.0
Pulse Sequence	zg30
Experiment	1D
Number of Scans	16
Acquisition Date	2017-05-04T11:15:00
Spectrometer Frequency	400.13
Spectral Width	8012.8
Lowest Frequency	-1543.1
Nucleus	1H
Acquired Size	32768
Spectral Size	65536

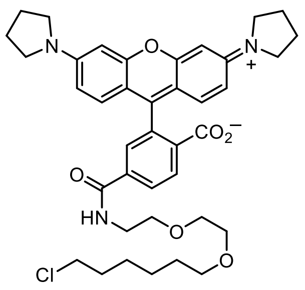

21

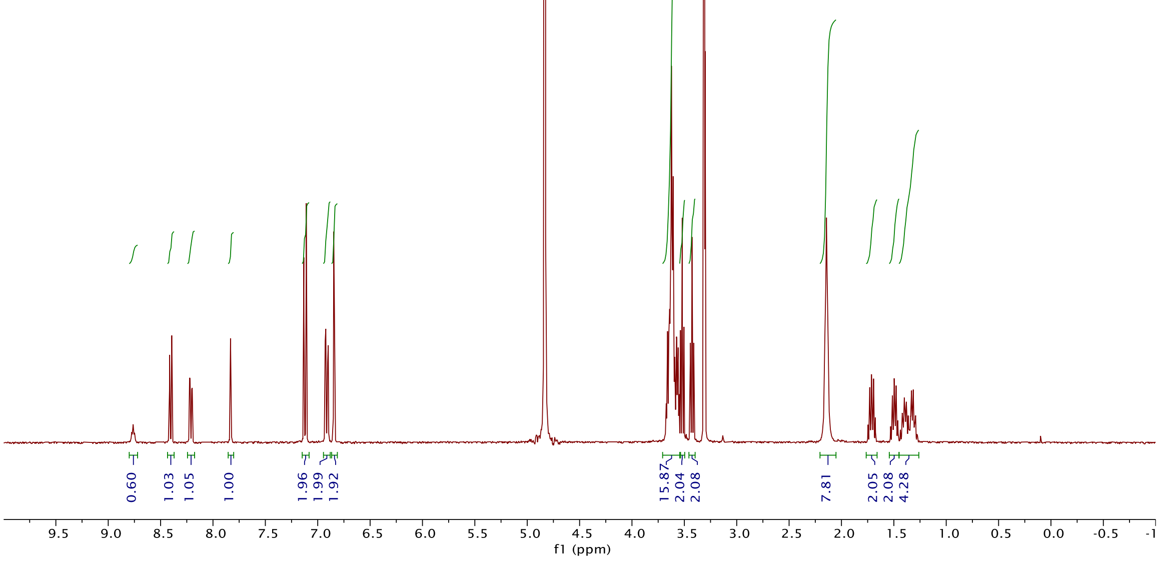

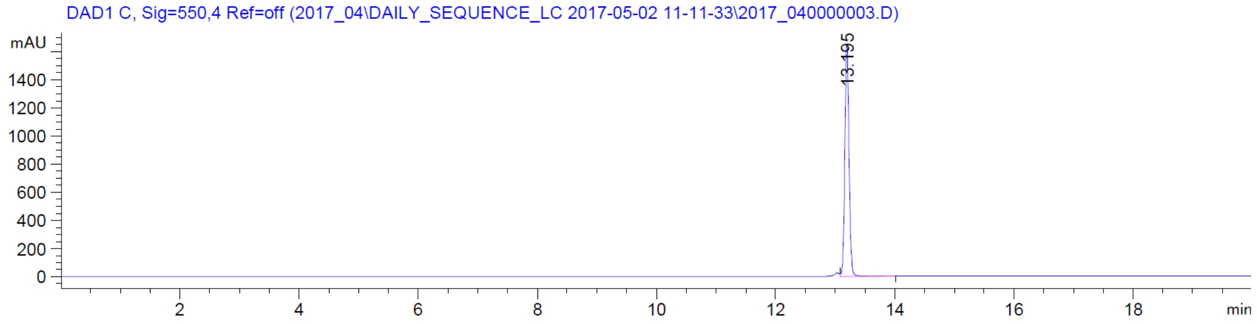

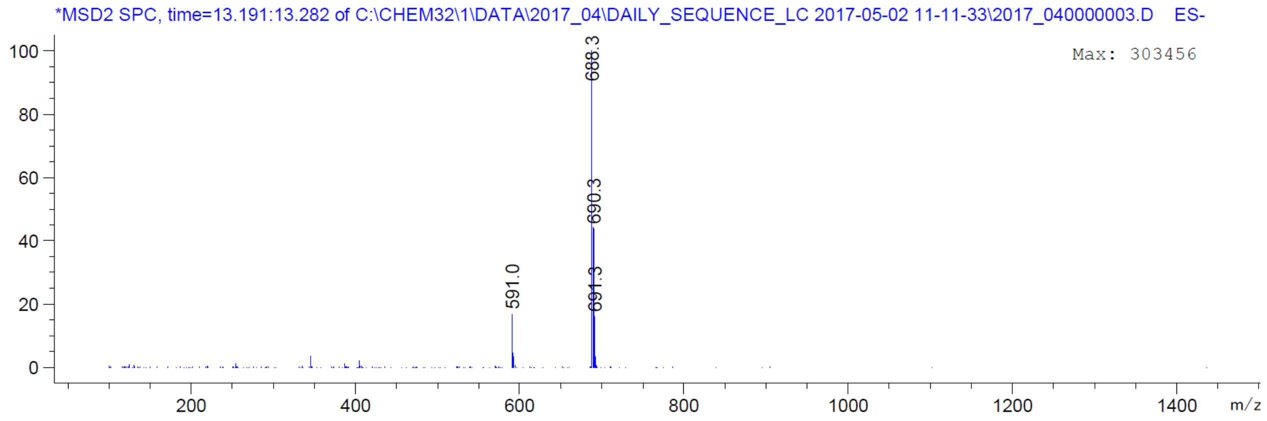

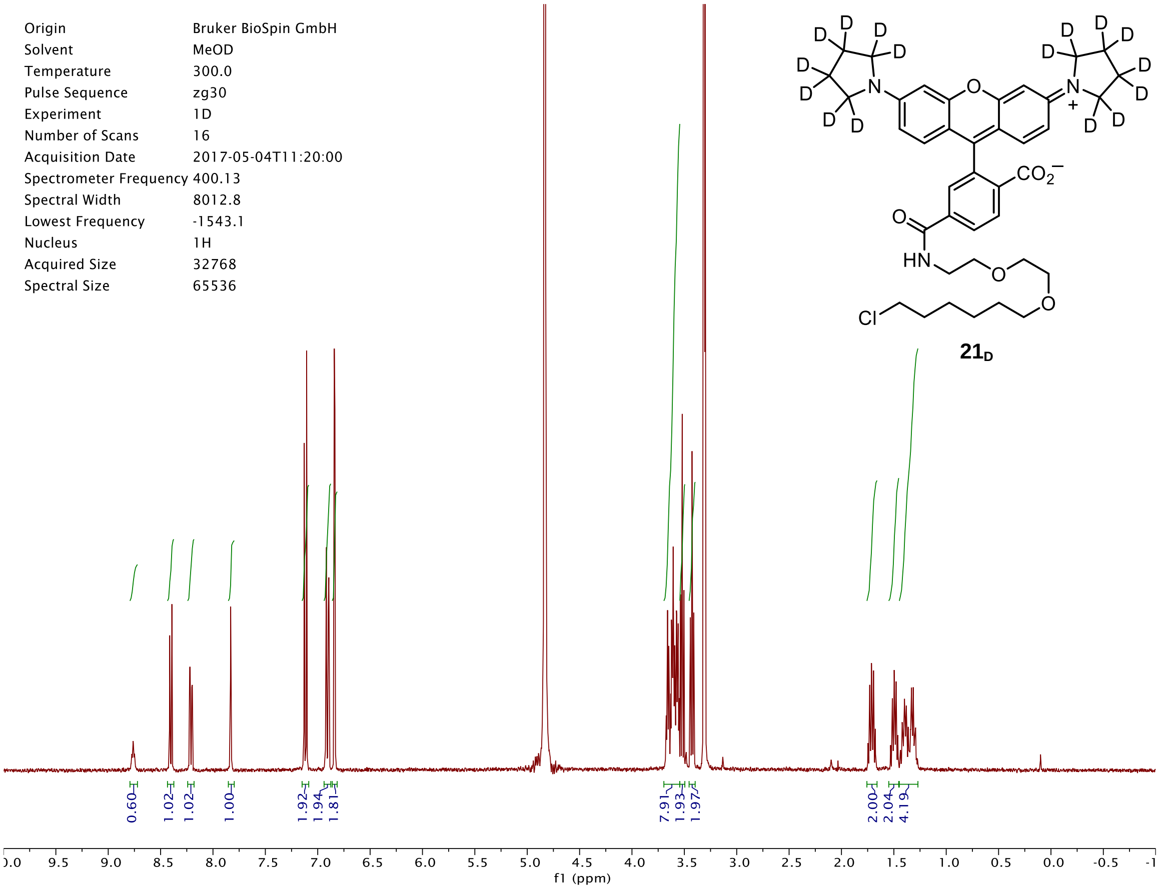
Origin Bruker BioSpin GmbH
Solvent MeOD
Temperature 300.0
Pulse Sequence zg30
Experiment 1D
Number of Scans 16
Acquisition Date 2017-05-04T11:20:00
Spectrometer Frequency 400.13
Spectral Width 8012.8
Lowest Frequency -1543.1
Nucleus 1H
Acquired Size 32768
Spectral Size 65536
21_D
0.60 1.02 1.02 1.00 1.92 1.94 1.81 7.91 1.93 1.97 2.00 2.04 4.19
f1 (ppm)

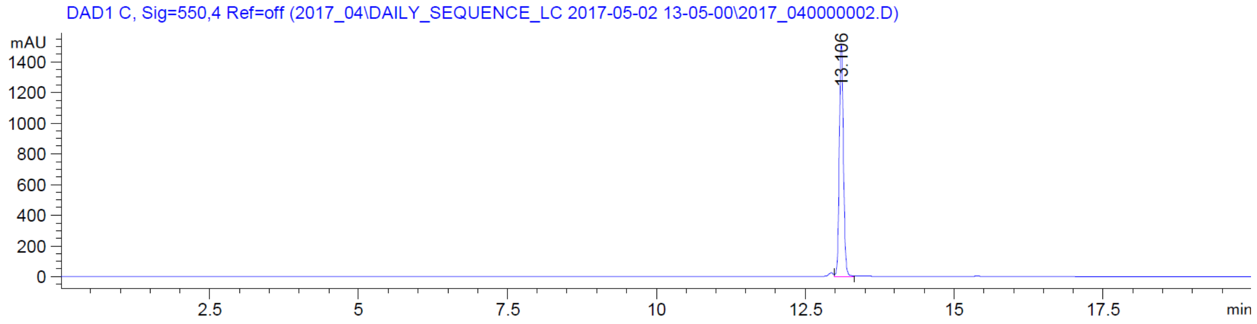
DAD1 C, Sig=550,4 Ref=off (2017_04\DAILY_SEQUENCE_LC 2017-05-02 13-05-00\2017_040000002.D)
mAU
13.106
min

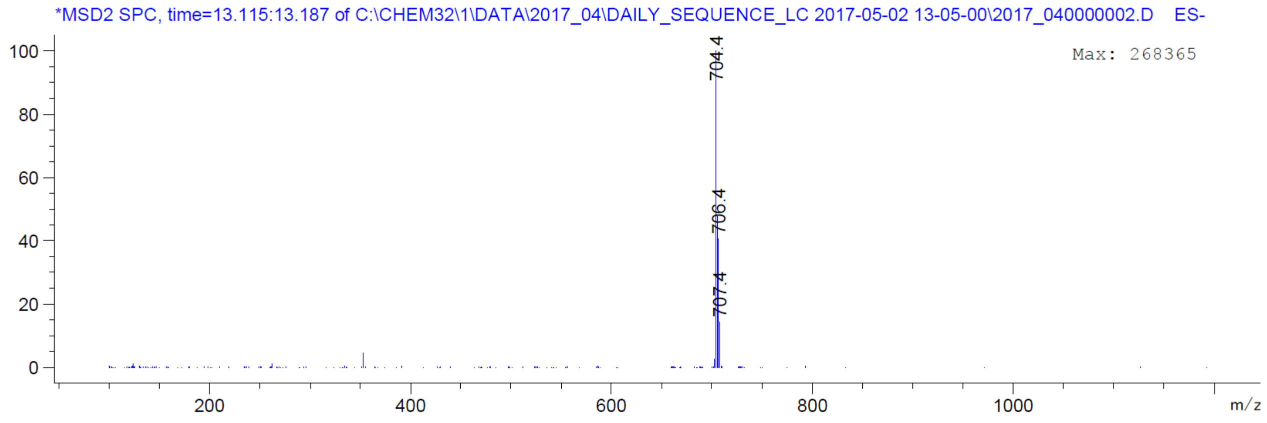
*MSD2 SPC, time=13.115:13.187 of C:\CHEM32\1\DATA\2017_04\DAILY_SEQUENCE_LC 2017-05-02 13-05-00\2017_040000002.D ES-
Max: 268365
704.4
706.4
707.4
m/z

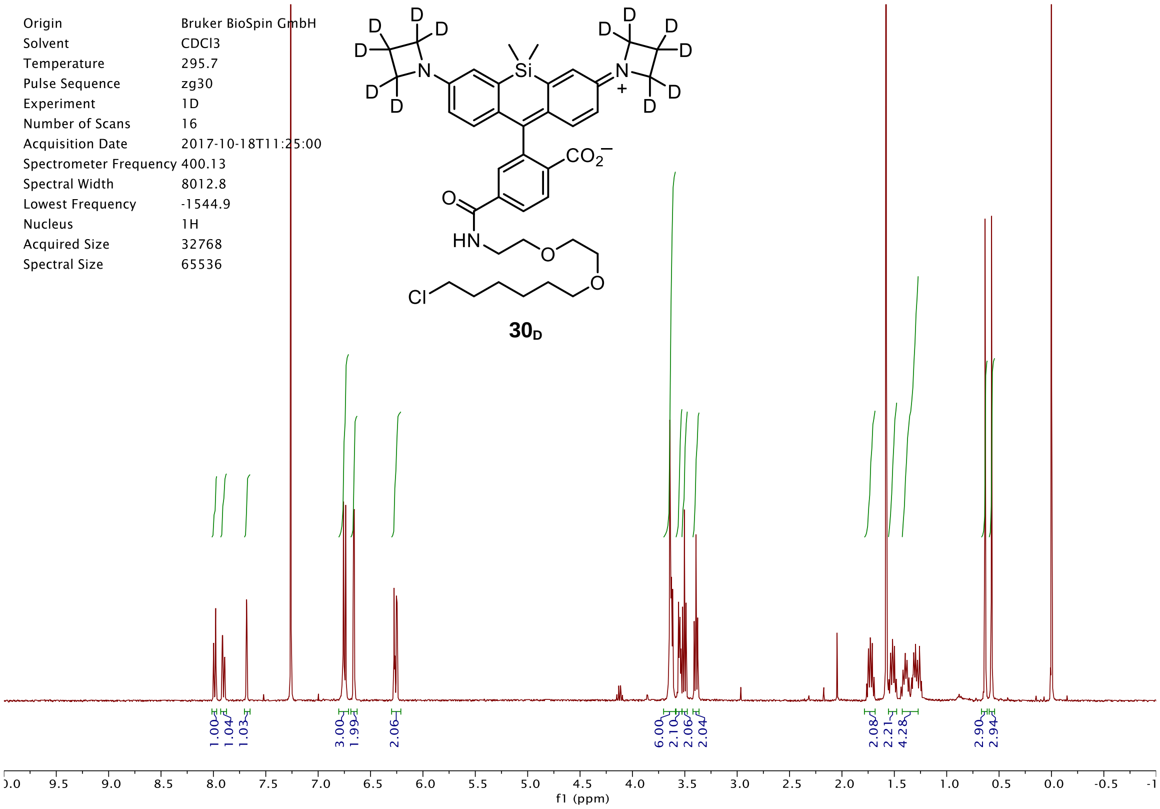

DAD1 E, Sig=650,4 Ref=off (2017\2017_10\DAILY_SEQUENCE_LC 2017-10-18 12-18-10\2017_100000003.D)

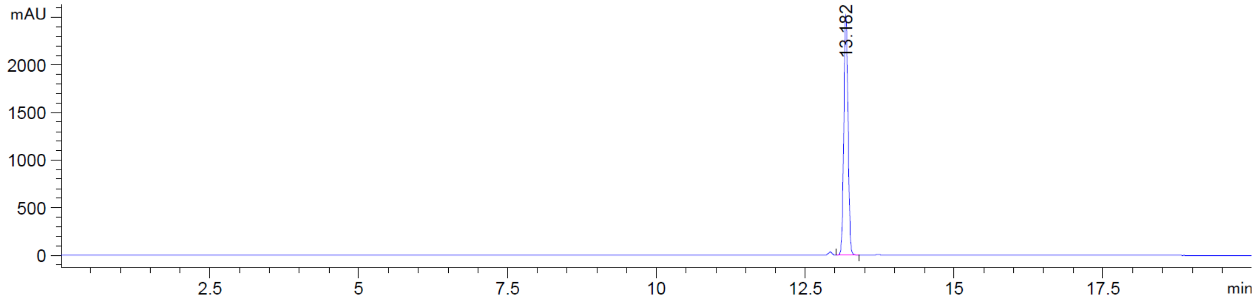

*MSD2 SPC, time=13.187:13.260 of C:\CHEM32\1\DATA\2017\2017_10\DAILY_SEQUENCE_LC 2017-10-18 12-18-10\2017_100000003.D

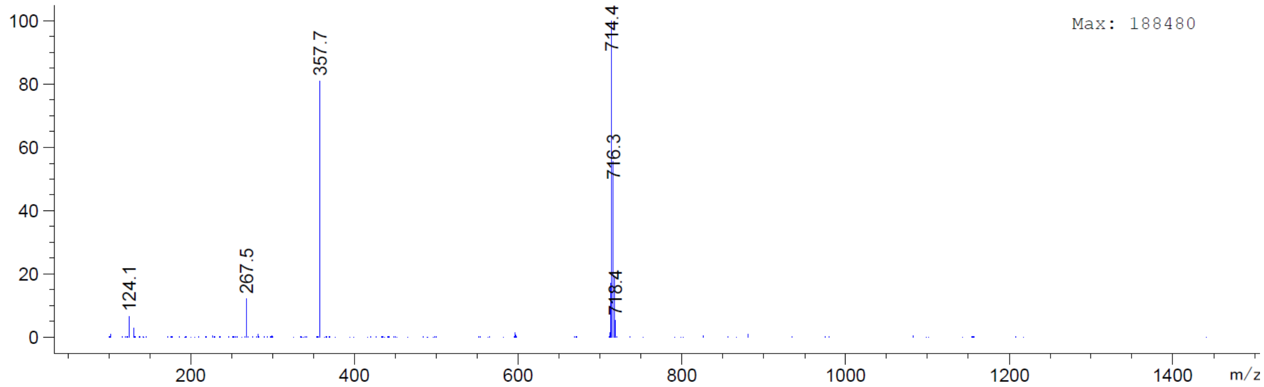

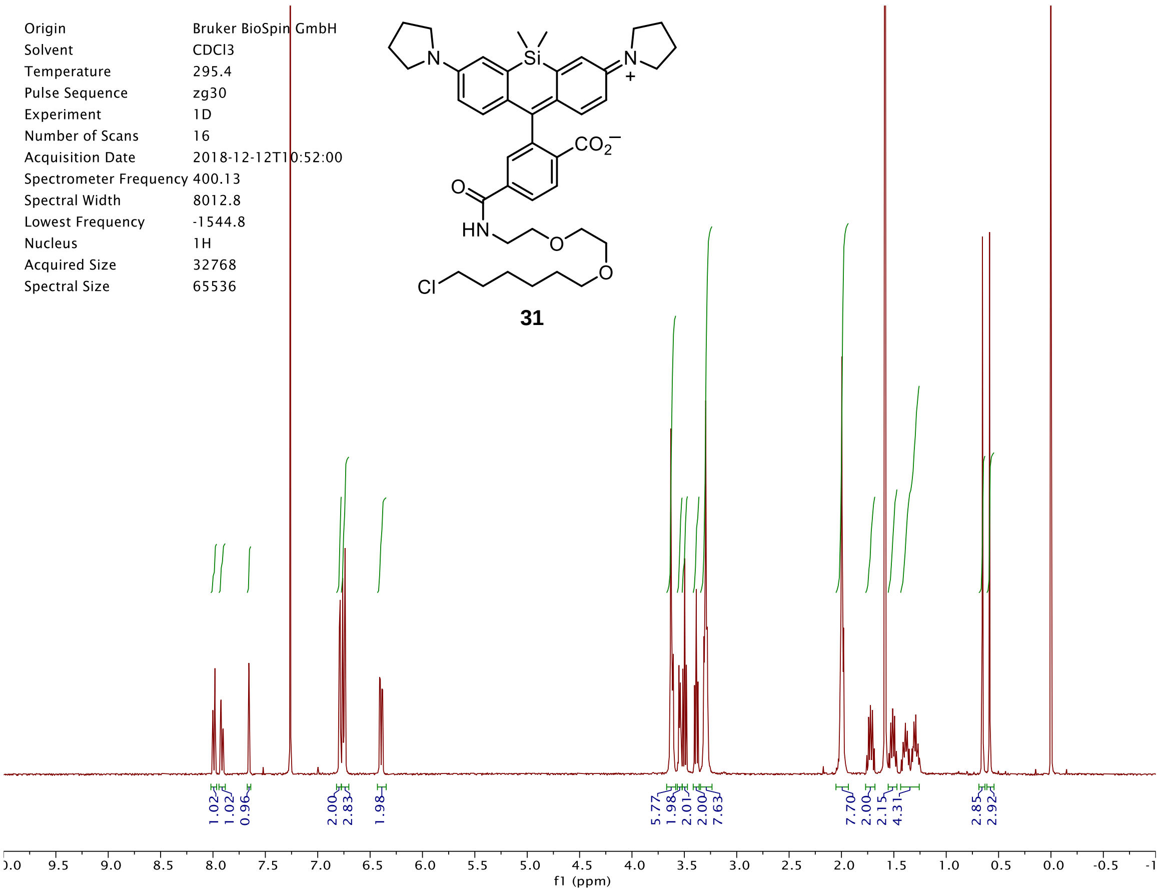
Origin Bruker BioSpin GmbH
Solvent CDCl3
Temperature 295.4
Pulse Sequence zg30
Experiment 1D
Number of Scans 16
Acquisition Date 2018-12-12T10:52:00
Spectrometer Frequency 400.13
Spectral Width 8012.8
Lowest Frequency -1544.8
Nucleus 1H
Acquired Size 32768
Spectral Size 65536
31
1.02 1.02 0.96 2.00 2.83 1.98 5.77 1.98 2.01 2.00 7.63 7.70 2.00 2.15 4.31 2.85 2.92
f1 (ppm)

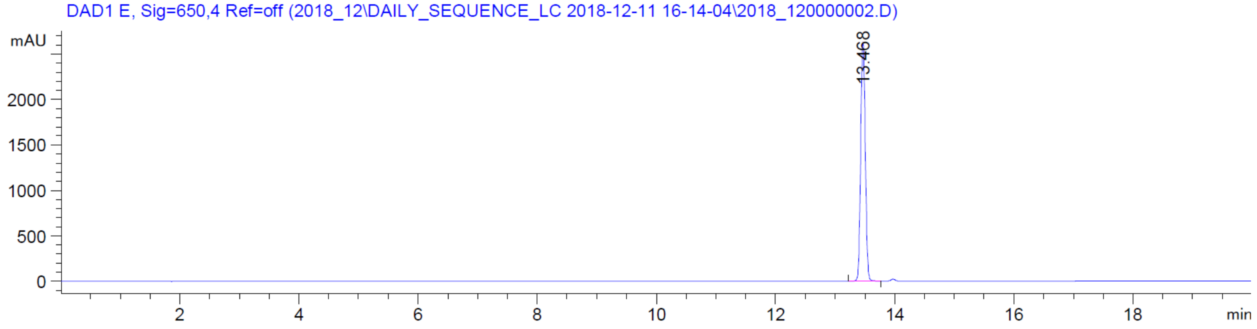
DAD1 E, Sig=650,4 Ref=off (2018_12\DAILY_SEQUENCE_LC 2018-12-11 16-14-04\2018_120000002.D)
mAU
13.468
min

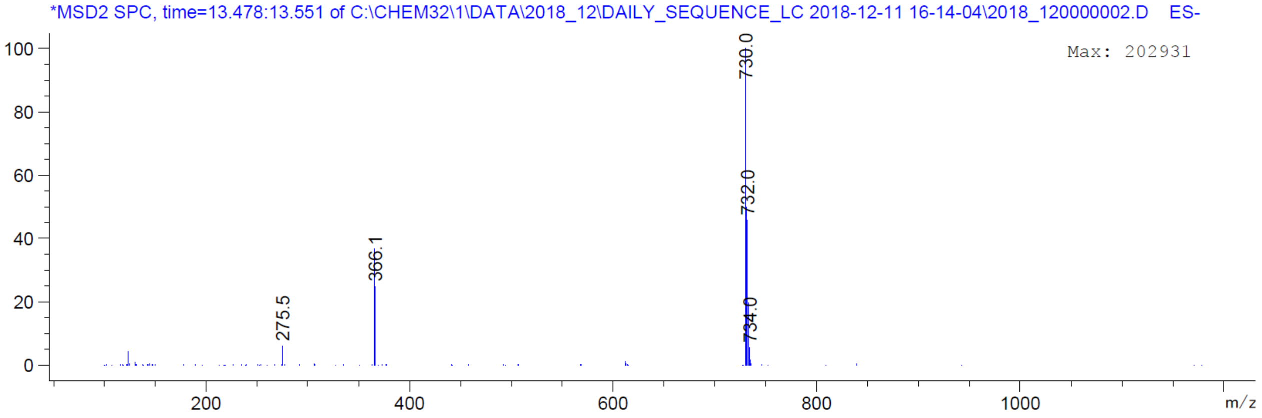
*MSD2 SPC, time=13.478:13.551 of C:\CHEM32\1\DATA\2018_12\DAILY_SEQUENCE_LC 2018-12-11 16-14-04\2018_120000002.D ES-
Max: 202931
730.0
732.0
734.0
366.1
275.5
m/z

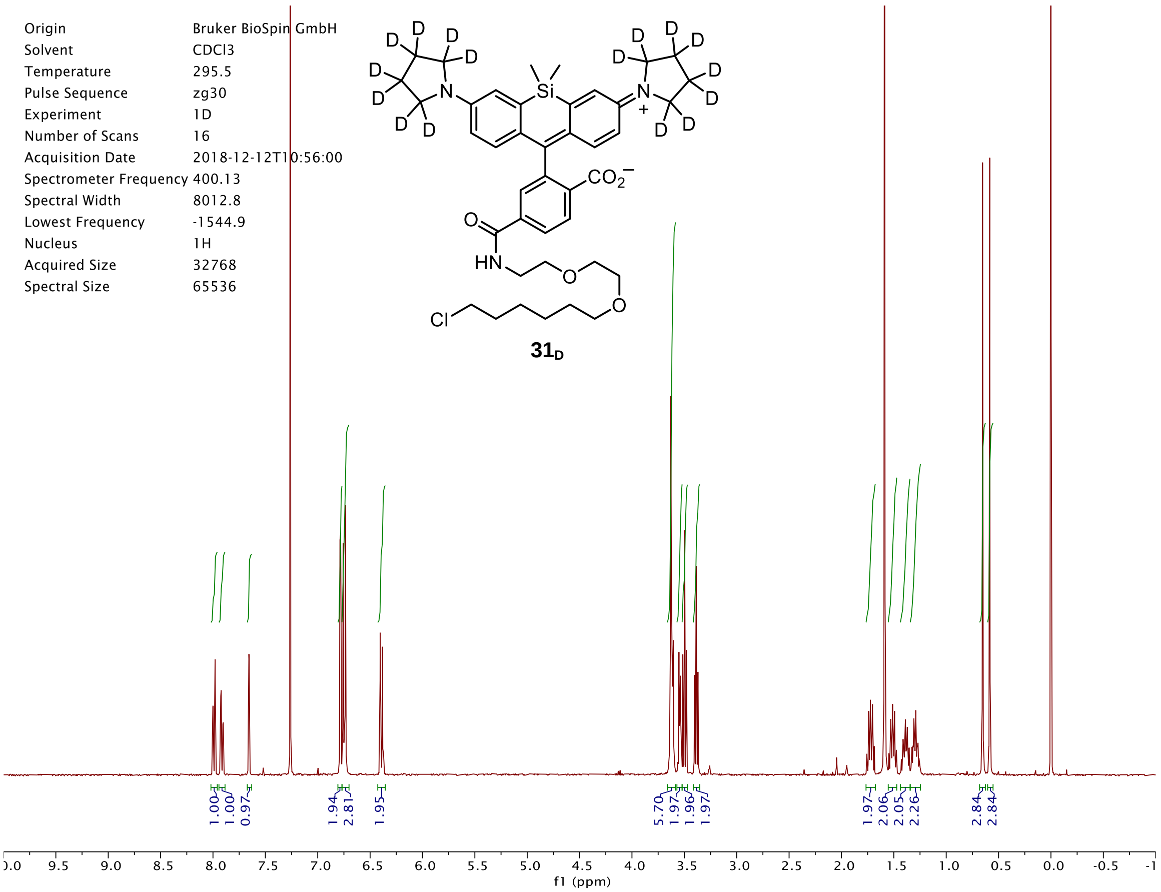
Origin Bruker BioSpin GmbH
Solvent CDCl3
Temperature 295.5
Pulse Sequence zg30
Experiment 1D
Number of Scans 16
Acquisition Date 2018-12-12T10:56:00
Spectrometer Frequency 400.13
Spectral Width 8012.8
Lowest Frequency -1544.9
Nucleus 1H
Acquired Size 32768
Spectral Size 65536
31D
f1 (ppm)

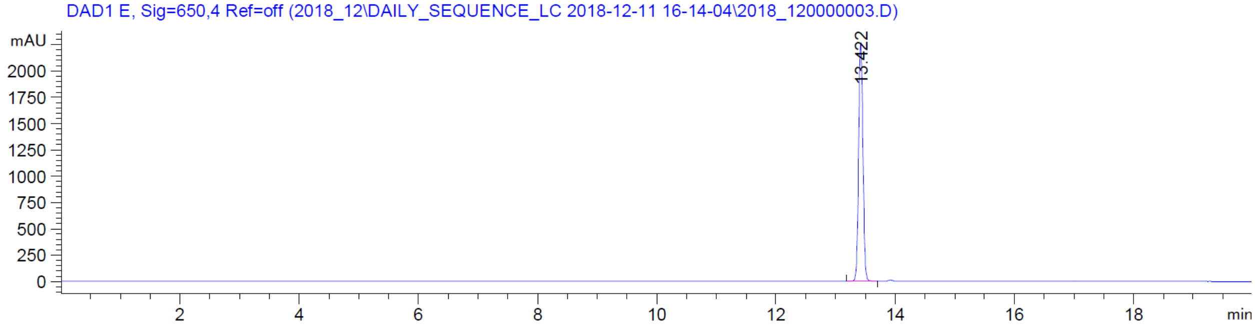
DAD1 E, Sig=650,4 Ref=off (2018_12\DAILY_SEQUENCE_LC 2018-12-11 16-14-04\2018_120000003.D)
mAU
13.422
min

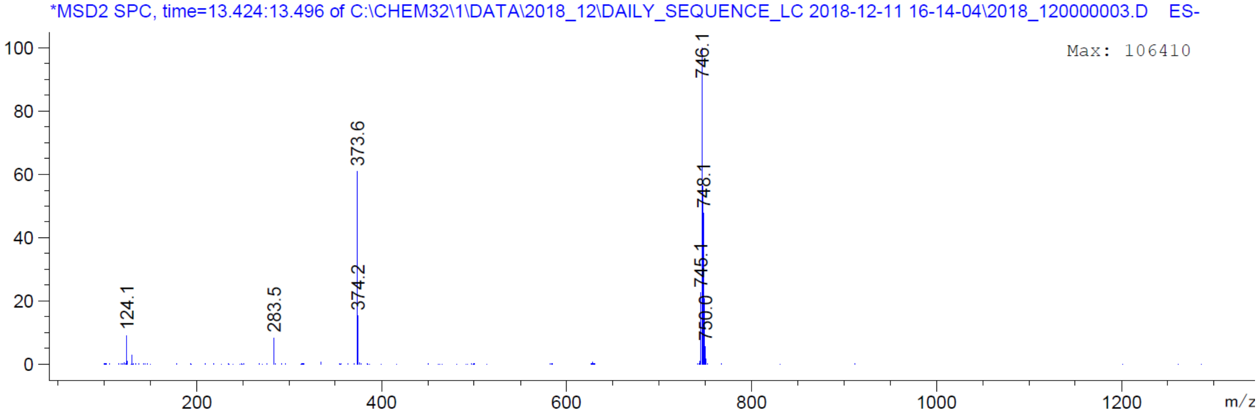
*MSD2 SPC, time=13.424:13.496 of C:\CHEM32\1\DATA\2018_12\DAILY_SEQUENCE_LC 2018-12-11 16-14-04\2018_120000003.D ES-
Max: 106410
746.1
748.1
745.1
750.0
373.6
374.2
283.5
124.1
m/z
